# Chemoenzymatic Total Synthesis of Deoxy‐, *epi*‐, and Podophyllotoxin and a Biocatalytic Kinetic Resolution of Dibenzylbutyrolactones

**DOI:** 10.1002/anie.201900926

**Published:** 2019-05-08

**Authors:** Mattia Lazzarotto, Lucas Hammerer, Michael Hetmann, Annika Borg, Luca Schmermund, Lorenz Steiner, Peter Hartmann, Ferdinand Belaj, Wolfgang Kroutil, Karl Gruber, Michael Fuchs

**Affiliations:** ^1^ Institute of Chemistry, Organic and Bioorganic Chemistry University of Graz Heinrichstrasse 28/II 8010 Graz Austria; ^2^ Austrian Centre of Industrial Biotechnology c/o University of Graz Graz Austria; ^3^ Institute of Molecular Biosciences University of Graz Humboldtstraße 50/III 8010 Graz Austria; ^4^ Institute of Chemistry Inorganic Chemistry University of Graz Schubertstraße 1/III 8010 Graz Austria

**Keywords:** 2-oxoglutarate-dependent dioxygenases, biocatalysis, lignans, podophyllotoxin, total synthesis

## Abstract

Podophyllotoxin is probably the most prominent representative of lignan natural products. Deoxy‐, *epi*‐, and podophyllotoxin, which are all precursors to frequently used chemotherapeutic agents, were prepared by a stereodivergent biotransformation and a biocatalytic kinetic resolution of the corresponding dibenzylbutyrolactones with the same 2‐oxoglutarate‐dependent dioxygenase. The reaction can be conducted on 2 g scale, and the enzyme allows tailoring of the initial, “natural” structure and thus transforms various non‐natural derivatives. Depending on the substitution pattern, the enzyme performs an oxidative C−C bond formation by C−H activation or hydroxylation at the benzylic position prone to ring closure.

The selective activation of inert C−H bonds represents one of the most remarkable reactions in modern organic chemistry.^1^ At the same time, it can be considered as one of the biggest challenges as well. Organometallic chemistry has provided an impressive assembly of examples, requiring in most cases adjacent directing groups to achieve selectivity.^1^ In contrast to these endeavors, nature provides selectivity for C−H activation through the enzyme's active site, which properly aligns the substrate(s) towards the active center. The most prominent representatives are cytochrome P450 monooxygenases (CYPs), which activate the C−H bond at an iron heme center with oxygen as a co‐substrate.^2^ While significant efforts have been dedicated to CYPs,^3^ 2‐oxoglutarate‐dependent dioxygenases (2‐ODDs) have gained rather moderate attention thus far.^4^ Predominantly, the hydroxylation of aminoacids has been studied in a biocatalytic context (e.g., hydroxylation of proline^5^ and leucine/isoleucine^6^). Nevertheless, recent reports demonstrate the high biocatalytic potenital of these proteins (even on gram scale).^7^ Besides these studies, the portfolio of reactivity is far beyond “simple” hydroxylation reactions: The biosyntheses of many natural products rely on 2‐ODDs, and reactions such as selective oxidation of sugar moieties,^8^ *endo*‐peroxide formation,^9^ ring expansion^9^ and contraction,^10^ selective halogenations,^11^ and multistep oxidations^12^ have been attributed to 2‐ODDs. Recently, the reactivity has been extended to the ring closure of the C‐ring in podophyllotoxin (**1**) biosynthesis by *Podophyllum hexandrum* 13 and *Sinopodophyllum hexandrum*.^14^ The 2‐ODD enzyme—further referred to as 2‐ODD‐PH—catalyzes the cyclization of yatein (**2 a**) and yields deoxypodophyllotoxin (**12 a**) at the expense of 2‐oxoglutaric acid and oxygen (Figure 1).

**Figure 1:**
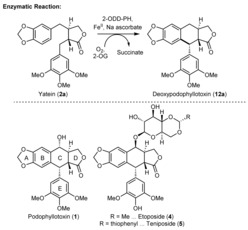
Reaction of 2‐ODD‐PH in nature and structures of pharmaceutical compounds.

Podophyllotoxin (**1**) is the precursor of etoposide (**4**) and teniposide (**5**), two chemotherapeutic agents used in several clinical therapies. Both compounds inhibit topoisomerase II, a key enzyme in cell mitosis, by forming a ternary complex with the DNA and isomerase enzyme. This prevents religation of the DNA strands and results in cell apoptosis. As cancer cells proliferate more rapidly, their cell death occurs preferentially.^15^ Etoposide (**5**) therefore became a member of the WHO's list of essential medicines.^16^ As other closely related compounds exhibit interesting bioactivities, too (e.g., cytotoxic, insecticidal, antifungal, antiviral, anti‐inflammatory, neurotoxic, immunosuppressive, antirheumatic, antispasmogenic, or hypolipidemic properties),^17^ this structural motif has been the subject of several synthetic research endeavors. Nevertheless, the asymmetric construction of the yatein precursor (**2 a**) and the stereoselective ring closure of ring C still represent major bottlenecks in the synthesis. Especially the construction of the southern stereocenter with the appropriate configuration has been remarkably difficult through the Friedel–Crafts approach, both chemically^18^ and by biotechnological methods.^19^ Some of these challenges have recently been resolved, but these approaches require expensive reagents or catalysts.^20^ Therefore, a chemoenzymatic, scalable route to the target compound podophyllotoxin (**1**) and the substrate scope of the biocatalyst are extremely tempting scientific targets.

Aiming for an efficient synthesis by integrating biocatalysis^21^ towards podophyllotoxin, the racemic yatein precursor (*rac*‐**2 a**) was prepared following a slightly modified literature procedure (Scheme 1 a).^22^ A simple switch to less polar solvents for the allylation step gave better diastereomeric ratios in our hands compared to the literature, and the target compound was obtained in good yields (82 % overall yield).

**Scheme 1:**
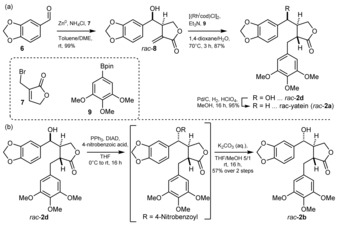
a) Preparation of *rac*‐yatein (*rac*‐**2 a**). b) Preparation of substrate *rac*‐**2 b**: installation of the “natural” stereoconfiguration. Bold and dashed lines refer to relative stereochemistry.

To access sufficient amounts of the enzyme 2‐ODD‐PH, the gene was expressed in *E. coli* BL21(DE3) using a pET21(a)+ vector system. As about 80 % of the catalyst first remained insoluble and inactive, the expression was improved by late induction (at OD_600_=1.2–1.4), yielding about 50 % of the enzyme in the soluble fraction at a high expression level (33 mg of soluble enzyme per 1.0 g of cells).

When performing the reaction with 2‐ODD‐PH (see the Supporting Information for details), substrate *rac*‐**2 a** was recovered with an *ee* of 10 % (26 % recovered material; see Table 1, entry 1), while two enantiopure products were observed. We identified these two products as the diastereomers deoxypodophyllotoxin (**12 a**; isolated in 19 % yield) and isodeoxypodophyllotoxin (**3**; isolated in 20 % yield). Thus, the biocatalyst is non‐stereoselective concerning the two chiral centers present in the substrate **2 a** and forms the new C−C bond within both substrate enantiomers. However, the new chiral center formed during C−C bond formation possesses the same configuration for both substrate enantiomers, leading to an enantiodivergent reaction (two diastereomers are formed). Consequently, the enzyme overrides the substrate‐controlled stereopreference for the ring formation.18a The diastereomeric mixture complicated the purification process, and pure products were obtained only by preparative HPLC.

**Table 1:** Biotransformations with 2‐ODD‐PH. 
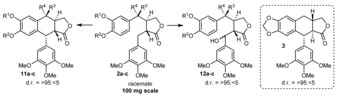

Entry	Substrate			HPLC area		Yield		*ee* of
		R^1^=	R^3^=	[%]^[a]^		[%]^[b]^		**2 a**–**c**
		R^2^=	R^4^=	**11 a**–**c**	**12 a**–**c**	**11 a**–**c**	**12 a**–**c**	[%]^[c]^
1	*rac*‐**2 a**	‐CH_2_‐	H H	27^[d]^	–	19^[e]^	–	10
								
2^[f]^	*rac*‐**2 b**	‐CH_2_‐	H OH	–	29	–	15	46
								
3	*rac*‐**2 c**	Me Me	OH H	12	5	7	4	26

Reaction conditions: Cell‐free extract (CFE, 44 v %), 20 mm substrate, 2‐oxoglutarate (1.75 equiv), sodium ascorbate (3 equiv), 23 % DMSO as cosolvent. [a] Determined by peak area integration of the HPLC‐UV chromatogram (at 215 nm). [b] Yields of isolated, chromatographically pure, and fully characterized products are reported; [c] *ee* was determined via HPLC‐UV on a chiral stationary phase (for details see the Supporting Information). [d] With 37 % of diastereoisomer **3**. [e] 20 % of compound **3** were isolated from the same batch; [*α*]_D_^20^ values for **11 a** and **3** are in full consistency with literature values.^24^ [f] The experiment was conducted on 50 mg substrate scale. Bold and dashed lines refer to relative stereochemistry; bold and dashed wedges refer to absolute stereochemistry.

These results encouraged us to challenge the biocatalyst with a substrate congener with an additional chiral center, such as the hydroxy group present in podophyllotoxin's northern benzylic position (Figure 1). In order to obtain the “natural” relative configuration of the OH group as present in podophyllotoxin (**1**), we performed a Mitsunobu inversion on intermediate *rac*‐**2 d**, and cleaved off the ester moiety in a two‐step process, yielding substrate *rac*‐**2 b** (Scheme 1 b).

The transformation of substrate *rac*‐**2 b** with the biocatalyst turned out to be enantioselective, leading to a kinetic resolution (see Table 1, entry 2). Only one enantiomer was transformed, but instead of the expected ring‐closed aryl tetralin structure, product **12 b** was obtained with the benzylic position—formerly prone to cyclization—hydroxylated. Substrate **2 b** may not be positioned properly in the enzyme, preventing the C−C bond formation and favoring hydroxylation instead. Interestingly, substrate **2 c** (Table 1, entry 3), which bears two methoxy groups on the western aromatic ring system, was converted into a mixture of products generated by ring closure (**11 c**) and hydroxylation (**12 c**). These findings are in consistency with a recent mechanistic study, where related hydroxylated products were observed.^23^ In agreement with this report, a cationic mechanism involving a Friedel–Crafts alkylation is the most likely mechanistic pathway of the reaction. Nevertheless, a radical pathway cannot be ruled out completely (for a detailed outline of the catalytic mechanism, see the Supporting Information), and structural information on the enzyme is required to provide further arguments for either pathway.

As substrate *rac*‐**2 b** was transformed via a kinetic resolution, the hydroxy function at the northern benzylic position seems to play a fundamental role in recognition. Substrate *rac*‐**2 d** possesses the opposite relative configuration for this hydroxy group, and therefore, we tested this compound, too (see Table 2, top scheme). The results clearly show that with this stereoconfiguration, the aromatic moiety seems to be aligned in the correct way, and the ring formation was observed exclusively in a kinetic resolution of substrate *rac*‐**2 d**. Only the enantiomer with the same absolute configuration for the northern hydroxy group as in etoposide (**4**) was transformed. Consequently, product **11 d** bears the same stereoconfiguration as the APIs etoposide (**4**) and teniposide (**5**; see Figure 1), which may facilitate the synthesis of these compounds.

**Table 2:** Biocatalytic transformation of substrate **2 d** by 2‐ODD‐PH. 
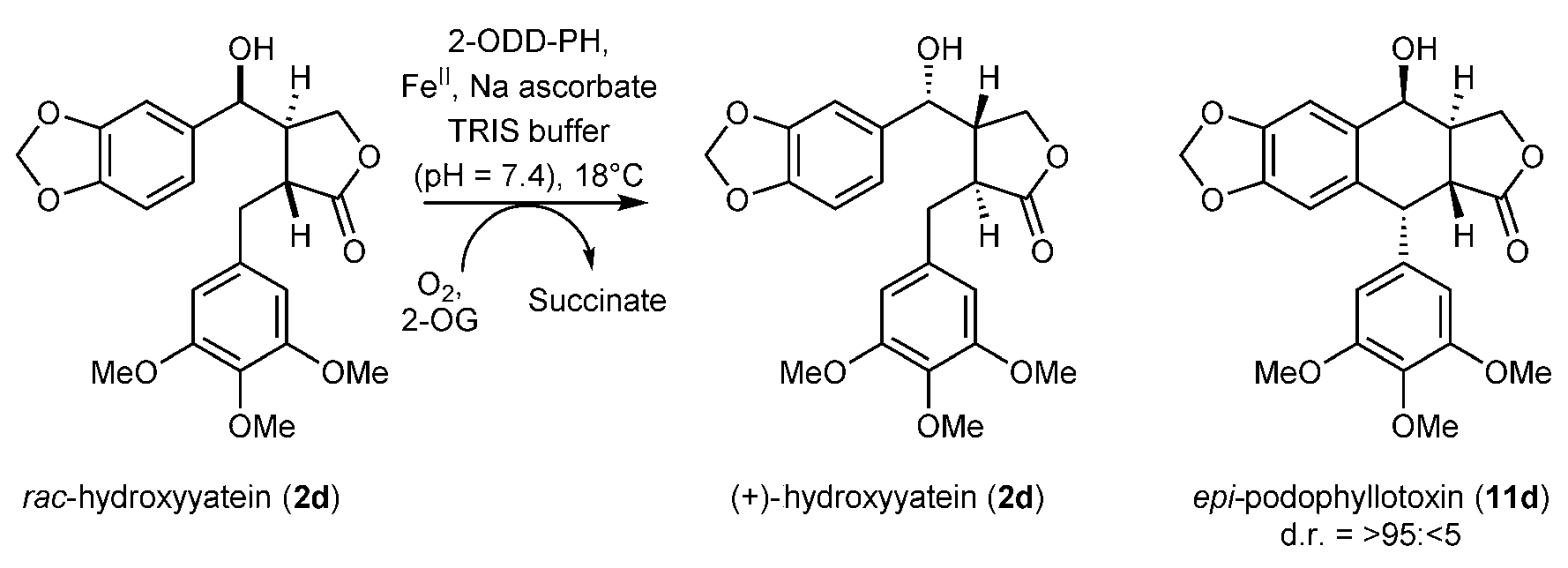

Entry	Scale	Conv.	**11 d** [%]		(+)‐**2 d** [%]	
		[%]^[a]^	Yield^[b]^	*ee* ^[d]^	Yield^[b]^	*ee* ^[c]^
1	1 mg	50	–^[e]^	99	–^[e]^	99
2	100 mg	43	38	95	50	72
3	2.0 g	41	39	95 (>99^[f]^)	45	66

Reaction conditions: CFE (44 v %), 20 mm substrate, 2‐oxoglutarate (1.75 equiv), sodium ascorbate (3 equiv), 23 % DMSO as cosolvent, 18 h; the absolute configurations were determined by comparison to literature values.^24^ [a] The conversion was determined from calibrated HPLC‐UV spectra. [b] Yields of isolated, chromatographically pure, and fully characterized products are reported. [c] The *ee* values were determined by HPLC‐UV analysis on a chiral stationary phase (for details see the Supporting Information). [d] The *ee* value was determined from the conversion and the *ee* of the remaining substrate.^25^ [e] Yield of isolated product not determined. [f] The *ee* was determined by HPLC‐UV analysis of the follow‐up product **1** (see Scheme 2).

The measured conversion was confirmed by the isolated yield of preparative reactions. We obtained 770 mg (39 % yield of isolated product) of the target compound **11 d** from a 235 mL reaction mixture within 18 h. This corresponds to a space–time yield of 200 mg L^−1^ h^−1^ and an overall yield of 32 % over four steps (note that a late‐stage kinetic resolution was performed, which limits the yield to a maximum of 50 %). The discrepancy in conversion between the small‐scale and larger‐scale experiments (see Table 2) results most likely from different surface‐to‐volume ratios, leading to a lower oxygen input, as well as from deactivation of the enzyme over time. Additionally, kinetic measurements imply that the reaction actually occurs over two to four hours only (see Tables S13 a and S13 b in the Supporting Information; the upscale experiment was performed for 16 h). This leads to a space–time yield of 1.6–0.8 g L^−1^ h^−1^ for the analytical scale, and we assume that similar numbers can be obtained in upscale experiments.

Consequently, the actual natural product podophyllotoxin (**1**) was prepared from **11 d** according to a literature procedure in two additional steps (17 % overall yield over five steps, see Scheme 2).

**Scheme 2:**
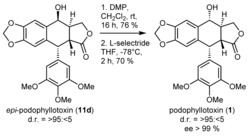
Preparation of podophyllotoxin (**1**) from its *epi*‐congener **10 b**.

Next, we focused on the substrate scope of the 2‐ODD‐PH enzyme; the results are depicted in Figure 2. Whereas the southern aromatic ring requires the trimethoxy motif for high enzyme activity, the substituents on the western aromatic system can be altered. Nevertheless, a phenyl substituent in the southern position was recently shown to be accepted by the enzyme at high enzyme concentrations (enzyme/substrate 1:1.2).^23^

**Figure 2:**
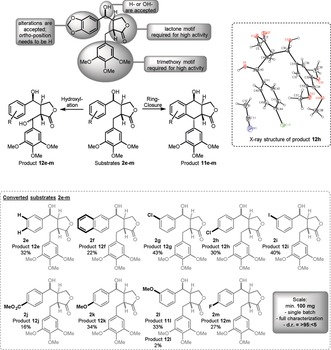
Substrate scope of the 2‐ODD‐PH enzyme. Schematic overview of the variable positions (top), two product structures (middle), and substrate scope (bottom). Alterations to substrate **2 d** are indicated with bold black lines and letters.

Despite the suitable configuration of the northern hydroxy function (see above), almost all substrates tested yielded the hydroxylated product type **12 e**–**m** (Figure 2). Only substrate **2 l**, which contains the methoxy substituent in *para* position towards the ring‐closing site, provided the aryl tetralin product **11 l** as the major product. It should be mentioned that the hydroxylated product **12 l** does not spontaneously cyclize under these reaction conditions. A schematic overview of the substrate alterations and their consequences can be found in Figure 2 (for a list of substrates that were not accepted, see the Supporting Information).

The lactone moiety and the southern aromatic fragment are required for high enzyme activity. The northern hydroxy function can be removed, resulting in alterations of the stereorecognition and the product outcome (see above). Substrates with larger substituents—namely acetoxy moieties—gave no conversion (see the Supporting Information). The most flexible part of the structural motif is the western aromatic moiety, for which several substituents in *meta* and *para* position are accepted. Substitution in the *ortho* position reduces the catalytic activity below the detection limit. For the *meta* and *para* positions, electron‐withdrawing as well as electron‐donating substituents are accepted, with similar yields for the corresponding products. Even bulkier substrates (e.g., naphthyl substrate **2 f**) are accepted, whereas heteroaromatic residues (e.g., a furyl substituent) led to no catalytic activity (see the Supporting Information).

The crystal structure that we obtained from product **12 h** (isolated from the biotransformation of compound **2 h**) provides information on the accepted enantiomer and the stereoselectivity of the hydroxylation (see Figure 2).

In conclusion, the chemoenzymatic, target‐oriented synthesis of podophyllotoxin (**1**) and its *epi*‐congener **11 d** was achieved by employing a stereoselective biocatalytic C−C bond formation as the key step. This enzymatic transformation lacks analogies in conventional organic chemistry as it overrides the stereopreference of the ring closure and provides a short way to the precursor of etoposide (**4**) and teniposide (**5**). The latter are key compounds in chemotherapy. The substrate screening delivered predominantly products **12 e**–**m**, and thus dibenzylbutyrolactones—another subclass of lignan natural products—in enantiopure form. The biocatalytic C−C bond formation was incorporated into a target‐oriented synthesis of *epi*‐podophyllotoxin (**11 d**), and the kinetic resolution was performed on a 2 g substrate scale. The study opens a new avenue into the synthesis of key therapeutic agents and homologues thereof using a biocatalytic C−C bond formation, and represents a rare example of the target‐oriented application of this class of enzymes.^26^

## Conflict of interest

The authors declare no conflict of interest.

## Supporting information

As a service to our authors and readers, this journal provides supporting information supplied by the authors. Such materials are peer reviewed and may be re‐organized for online delivery, but are not copy‐edited or typeset. Technical support issues arising from supporting information (other than missing files) should be addressed to the authors.

Supplementary
