# Supplementary material for: Chemoenzymatic Total Synthesis of Deoxy‐, epi‐, and Podophyllotoxin and a Biocatalytic Kinetic Resolution of Dibenzylbutyrolactones
Source: Angew Chem Int Ed Engl. 2019 May 8;58(24):8226–30. doi: 10.1002/anie.201900926 (PMC6563474; doi:10.1002/anie.201900926)
Supplement: Supplementary file 1 — Supplementary [file ANIE-58-8226-s001.pdf]

Angewandte Chemie
Eine Zeitschrift der Gesellschaft Deutscher Chemiker

Supporting Information

Chemoenzymatic Total Synthesis of Deoxy-, *epi*-, and Podophyllotoxin and a Biocatalytic Kinetic Resolution of Dibenzylbutyrolactones

Mattia Lazzarotto, Lucas Hammerer, Michael Hetmann, Annika Borg, Luca Schmermund, Lorenz Steiner, Peter Hartmann, Ferdinand Belaj, Wolfgang Kroutil, Karl Gruber, and Michael Fuchs**

anie_201900926_sm_miscellaneous_information.pdf

Supporting Information

Chemoenzymatic Totalsynthesis of deoxy- and epi-Podophyllotoxin via Biocatalytic C-H Activation by 2-Oxoglutarate Dependent Dioxygenases

Mattia Lazzarotto, Lucas Hammerer, Michael Hetmann, Annika Borg, Luca Schmermund, Lorenz Steiner, Peter Hartmann, Ferdinand Belaj, Wolfgang Kroutil, Karl Gruber and Michael Fuchs

Table of Contents

Table of Contents 2

Previous synthetic approaches towards podophyllotoxin 6

Enzymatic Mechanism 7

Optimization of Enzymatic Transformation 8

Control Experiments and Kinetic Study 12

Substrate Conversions 15

Substrates Showing No Conversion 19

Experimental Procedures 20

General Information 20

DNA sequence of the biocatalyst 20

Preparation of the biocatalyst 21

Cell lysis 21

Enzyme purification 21

General procedure for the allylation of aldehydes with bromolactone **7.** 22

rac-4-[benzo[d][1,3]dioxol-5-yl(hydroxy)methyl]-3-methylenedihydrofuran-2(3*H*)-one (**8**). 22

rac-4-[(3,4-dimethoxyphenyl)(hydroxy)methyl]-3-methylenedihydrofuran-2(3*H*)-one (SI-**12**). 23

rac-4-[hydroxy(phenyl)methyl]-3-methylenedihydrofuran-2(3*H*)-one (**SI-13**). 23

rac-4-[hydroxy(naphthalen-2-yl)methyl]-3-methylenedihydrofuran-2(3*H*)-one (**SI-14**). 23

rac-4-[(3-chlorophenyl)(hydroxy)methyl]-3-methylenedihydrofuran-2(3*H*)-one (**SI-15**). 24

rac-4-[(4-chlorophenyl)(hydroxy)methyl]-3-methylenedihydrofuran-2(3*H*)-one (**SI-16**). 24

rac-4-[(3-Iodophenyl)(hydroxy)methyl]-3-methylenedihydrofuran-2(3H)-one (**SI-17**). 25

rac-Methyl 4-[hydroxy(4-methylene-5-oxotetrahydrofuran-3-yl)methyl]benzoate (**SI-18**)... 25

rac-4-[hydroxy(4-methoxyphenyl)methyl]-3-methylenedihydrofuran-2(3*H*)-one (**SI-19**). 25

rac-4-[hydroxy(3-methoxyphenyl)methyl]-3-methylenedihydrofuran-2(3*H*)-one (**SI-20**). 26

rac-4-[(4-fluorophenyl)(hydroxy)methyl]-3-methylenedihydrofuran-2(3*H*)-one (**SI-21**). 26

rac-4-[(2-chlorophenyl)(hydroxy)methyl]-3-methylenedihydrofuran-2(3*H*)-one (**SI-22**). 27

rac-4-[furan-2-yl(hydroxy)methyl]-3-methylenedihydrofuran-2(3*H*)-one (**SI-23**). 27

rac-4-[Hydroxy(4-hydroxyphenyl)methyl]-3-methylenedihydrofuran-2(3H)-one (**SI-25**). 27

rac-4-[(4-Iodophenyl)(hydroxy)methyl]-3-methylenedihydrofuran-2(3H)-one (**SI-26**). 28

General procedure for the 1,4-addition of boronic acid ester to a unsaturated lactone....... 28

rac-4-{Benzo[d][1,3]dioxol-5-yl(hydroxy)methyl}-3-(3,4,5-trimethoxybenzyl)dihydrofuran-2(3H)-one (**2d**). 29

rac-4-[(3,4-dimethoxyphenyl)(hydroxy)methyl]-3-(3,4,5-trimethoxybenzyl)dihydrofuran-2(3H)-one (**2c**). 29

rac-4-[hydroxy(phenyl)methyl)-3-(3,4,5-trimethoxybenzyl)dihydrofuran-2(3H)-one (**2e**). ... 30

rac-4-[hydroxy(naphthalen-2-yl)methyl]-3-(3,4,5-trimethoxybenzyl)dihydrofuran-2(3H)-one (**2f**). 30

rac-4-[(3-chlorophenyl)(hydroxy)methyl]-3-(3,4,5-trimethoxybenzyl)dihydrofuran-2(3H)-one (**2g**). 31

rac-4-[(4-chlorophenyl)(hydroxy)methyl]-3-(3,4,5-trimethoxybenzyl)dihydrofuran-2(3*H*)-one (**2h**). 31

rac-4-[(3-Iodophenyl)(hydroxy)methyl]-3-(3,4,5-trimethoxybenz-yl)dihydrofuran-2(3*H*)-one (**2i**): 32

rac-Methyl 4-{hydroxy[5-oxo-4-(3,4,5-trimethoxybenzyl)tetrahydrofuran-3-yl]methyl}benzoate (**2j**). 32

rac-4-[hydroxy(4-methoxyphenyl)methyl]-3-(3,4,5-trimethoxybenzyl)dihydrofuran-2(3*H*)-one (**2k**). 33

rac-4-[hydroxy(3-methoxyphenyl)methyl]-3-(3,4,5-trimethoxybenzyl)dihydrofuran-2(3*H*)-one (**2l**). 33

rac-4-[hydroxy(4-fluorophenyl)methyl]-3-(3,4,5-trimethoxybenzyl)dihydrofuran-2(3*H*)-one (**2m**). 34

rac-4-[(4-iodophenyl)(hydroxy)methyl]-3-(3,4,5-trimethoxybenzyl)dihydrofuran-2(3*H*)-one (**SI-1**). 34

rac-4-[Benzo[d][1,3]dioxol-5-yl(hydroxy)methyl]-3-(3,4-dimethoxybenzyl)dihydrofuran-2(3*H*)-one (**SI-2**). 35

rac-4-[Benzo[d][1,3]dioxol-5-yl(hydroxy)methyl]-3-(4-methoxybenzyl)dihydrofuran-2(3*H*)-one (**SI-3**). 35

rac-4-[(2-chlorophenyl)(hydroxy)methyl]-3-(3,4,5-trimethoxybenzyl)dihydrofuran-2(3*H*)-one (**SI-5**). 36

rac-4-[furan-2-yl(hydroxy)methyl]-3-(3,4,5-trimethoxybenzyl)dihydrofuran-2(3H)-one (**SI-6**). 36

rac-4-[hydroxy(4-hydroxyphenyl)methyl]-3-(3,4,5-trimethoxybenzyl)dihydrofuran-2(3*H*)-one (**SI-10**). 37

rac-Yatein (**2a**). 37

rac-4-(benzo[d][1,3]dioxole-5-carbonyl)-3-(3,4,5-trimethoxybenzyl)dihydrofuran-2(3*H*)-one (**SI-7**). 38

rac-benzo[d][1,3]dioxol-5-yl[5-oxo-4-(3,4,5-trimethoxybenzyl)tetrahydrofuran-3-yl]methyl acetate (**SI-8**). 38

rac-4-[(-Benzo[d][1,3]dioxol-5-yl(hydroxy)methyl]-3-(3,4,5-trimethoxybenz-yl)dihydrofuran-2(3*H*)-one (**2b**). 39

Preparation of substrate **SI-4**. 39

General procedure for screening of the 2-ODD enzyme on analytical scale 41

General procedure for biotransformation with the 2-ODD enzyme on preparative scale..... 43

epi-Podophyllotoxine (**11d**). 44

Upscale of yatein (**2a**). 45

Upscale of substrate **2c**. 47

(3*S*,4*R*)-3-[Hydroxy(3,4,5-trimethoxyphenyl)methyl]-4-[(*S*)-hydroxy(phenyl)methyl]dihydrofuran-2(3*H*)-one (**12e**). 48

(3*S*,4*R*)-3-[Hydroxy(3,4,5-trimethoxyphenyl)methyl]-4-[(*S*)-hydroxy(naphthalen-2-yl)methyl]dihydrofuran-2(3*H*)-one (**2f**). 49

(3*S*,4*R*)-4-[(*S*)-(3-chlorophenyl)(hydroxy)methyl]-3-[hydroxy(3,4,5-trimethoxyphenyl)methyl]dihydrofuran-2(3*H*)-one (**12g**). 49

(3*S*,4*R*)-4-[(*S*)-(4-chlorophenyl)(hydroxy)methyl]-3-[hydroxy(3,4,5-trimethoxyphenyl)methyl]dihydrofuran-2(3*H*)-one (**12h**). 50

(3*S*,4*R*)-4-[(*S*)-(3-iodophenyl)(hydroxy)methyl]-3-[hydroxy(3,4,5-trimethoxyphenyl)methyl]dihydrofuran-2(3*H*)-one (**12i**). 51

Methyl 4-((1*S*)-hydroxy{(3*R*,4*S*)-4-[hydroxy(3,4,5-trimethoxyphenyl)methyl]-5-oxotetrahydrofuran-3-yl}methyl)benzoate (**12j**). 51

(3*S*,4*R*)-3-[hydroxy(3,4,5-trimethoxyphenyl)methyl]-4-[(*S*)-hydroxy(4-methoxyphenyl)methyl]dihydrofuran-2(3*H*)-one (**12k**). 52

Upscale of substrate **2l**. 52

(3*S*,4*R*)-4-[(*S*)-(4-fluorophenyl)(hydroxy)methyl]-3-[hydroxy(3,4,5-trimethoxyphenyl)methyl]dihydrofuran-2(3*H*)-one (**12m**). 53

Two gram upscale for the preparation of *epi*-podophyllotoxin (**11d**). 54

(3*S*,4*R*)-4-[(R)-(-Benzo[d][1,3]dioxol-5-yl(hydroxy)methyl]-3-[hydroxy(3,4,5-trimethoxyphenyl)methyl]dihydrofuran-2(3*H*)-one (**12b**). 54

Podophyllotoxone (**SI-42**) 55

Podophyllotoxin (**1**). 55

Control experiment for an enzyme-catalyzed ring closure (see page 12 in this supporting information) – experimental procedure. 57

Crystal Structure Determination of Compound **12h** 57

References 63

NMR-Data 64

HPLC Chromatograms 122

epi-Podophyllotoxin (**11d**, 2 gram upscale experiment). 123

Upscale of yatein (**2**). 125

Upscale of substrate **2c**. 127

(3*S*,4*R*)-3-[Hydroxy(3,4,5-trimethoxyphenyl)methyl]-4-[(*S*)-hydroxy(phenyl)methyl]dihydrofuran-2(3*H*)-one (**12e**). 129

(3*S*,4*R*)-3-[Hydroxy(3,4,5-trimethoxyphenyl)methyl]-4-[(*S*)-hydroxy(naphthalen-2-yl)methyl]dihydrofuran-2(3*H*)-one (**12f**). 131

(3*S*,4*R*)-4-[(*S*)-(3-chlorophenyl)(hydroxy)methyl]-3-[hydroxy(3,4,5-trimethoxyphenyl)methyl]dihydrofuran-2(3*H*)-one (**12g**). 133

(3*S*,4*R*)-4-[(*S*)-(4-chlorophenyl)(hydroxy)methyl]-3-[hydroxy(3,4,5-trimethoxyphenyl)methyl]dihydrofuran-2(3*H*)-one (**12h**). 135

(3S,4R)-4-[(S)-(3-iodophenyl)(hydroxy)methyl]-3-[hydroxy(3,4,5-trimethoxyphenyl)methyl]dihydrofuran-2(3*H*)-one (**12i**). 137

Methyl 4-((1*S*)-hydroxy{(3*R*,4*S*)-4-[hydroxy(3,4,5-trimethoxyphenyl)methyl]-5-oxotetrahydrofuran-3-yl}methyl)benzoate (**12j**). 139

(3*S*,4*R*)-3-[hydroxy(3,4,5-trimethoxyphenyl)methyl]-4-[(*S*)-hydroxy(4-methoxyphenyl)methyl]dihydrofuran-2(3*H*)-one (**12k**).. 141

(3*S*,4*R*)-3-[hydroxy(3,4,5-trimethoxyphenyl)methyl]-4-[(*S*)-hydroxy(3-methoxyphenyl)methyl]dihydrofuran-2(3*H*)-one (**11l**)... 143

(3*S*,4*R*)-4-[(*S*)-(4-fluorophenyl)(hydroxy)methyl]-3-[hydroxy(3,4,5-trimethoxyphenyl)methyl]dihydrofuran-2(3*H*)-one (**12m**)... 145

(3*S*,4*R*)-4-[(*R*)-(-Benzo[d][1,3]dioxol-5-yl(hydroxy)methyl]-3-[hydroxy(3,4,5-trimethoxyphenyl)methyl]dihydrofuran-2(3*H*)-one (Upscale Substrate **2b**)....................... 147

Biotransformation of (3*R*,4*R*)-4-[(*S*)-hydroxy(4-iodophenyl)methyl]-3-(3,4,5-trimethoxybenzyl)dihydrofuran-2(3*H*)-one (**SI-1**)... 149

Control experiment for enzyme catalysis during the ring closure (see page 12 in this supporting information)... 150

Podophyllotoxin (**1**)... 152

Calibration Curves for Table SI14b (see p. 18) 153

Previous synthetic approaches towards podophyllotoxin

Table SI01. Previous synthetic routes

Entry	Year[ref.]	steps	Overall yield [%]	Notes
1	1966[1]	17	0.3	Racemate
2	1986[2]	12	4	Racemate
3	1987[3]	7	6	Racemate; starting from literature known intermediate
4	1988[4]	16	< 3	Racemate; starting from literature known intermediate
5	1988[5]	24	5	First asymmetric synthesis of (-)-podophyllotoxin
6	1989 [6]	7	18	Racemate; starting from literature known intermediate
7	1991 [7]	14	20	starting from literature known intermediate; chiral auxiliary was used; (-)-*epi*-podophyllotoxin (**11d**) was obtained
8	1993[8]	8	15	starting from literature known intermediate; chiral auxiliary was used; (-)-*epi*-podophyllotoxin (**11d**) was obtained
9	1996[9]	5	-[a]	starting from literature known intermediate; chiral auxiliary was used
10	1996[10]	6	< 15	starting from literature known intermediate; chiral auxiliary was used
11	2000[11]	> 6	35	Starting from Taniguchi lactone[12]; keystep: alcohol dehydrogenase catalyzed desymmetrization
12	2003 [13]	12	30	Chiral auxiliary was used; (-)-*epi*-podophyllotoxin (**11d**) was obtained
13	2007[14]	9	29	Racemate
14	2008[15]	> 6	35	Starting from Taniguchi lactone[12]
15	2009[16]	7	35	Enantioselective variant of ref. [[14]]; chiral auxiliary was used; (+)-*ent*-podophyllotoxin was prepared
16	2014 [17]	4	8	Keystep: Pd catalyzed, diastereoselective C-H activation; mixture of podophyllotoxin and *epi*-podophyllotoxin (**11d**) (1.3 : 1) was obtained
17	2016[18]	11	6[b]	Keystep: photocyclization; use of chiral auxiliary; formal totalsynthesis
18	2017[19]	7	27	Highly catalytic route; starting material for initial asymmetric step (methyl 4-oxobutanoate) is expensive and required in high excess (5 eq.)
19	2018[20]	11	7	Target oriented (gram scale); diastereoisomers (1:1.2) are obtained in Ni catalyzed key step

[a] isolated yields were only reported for key steps; [b] overall yield of literature known intermediate in the podophyllotoxin synthesis is given.

Enzymatic Mechanism

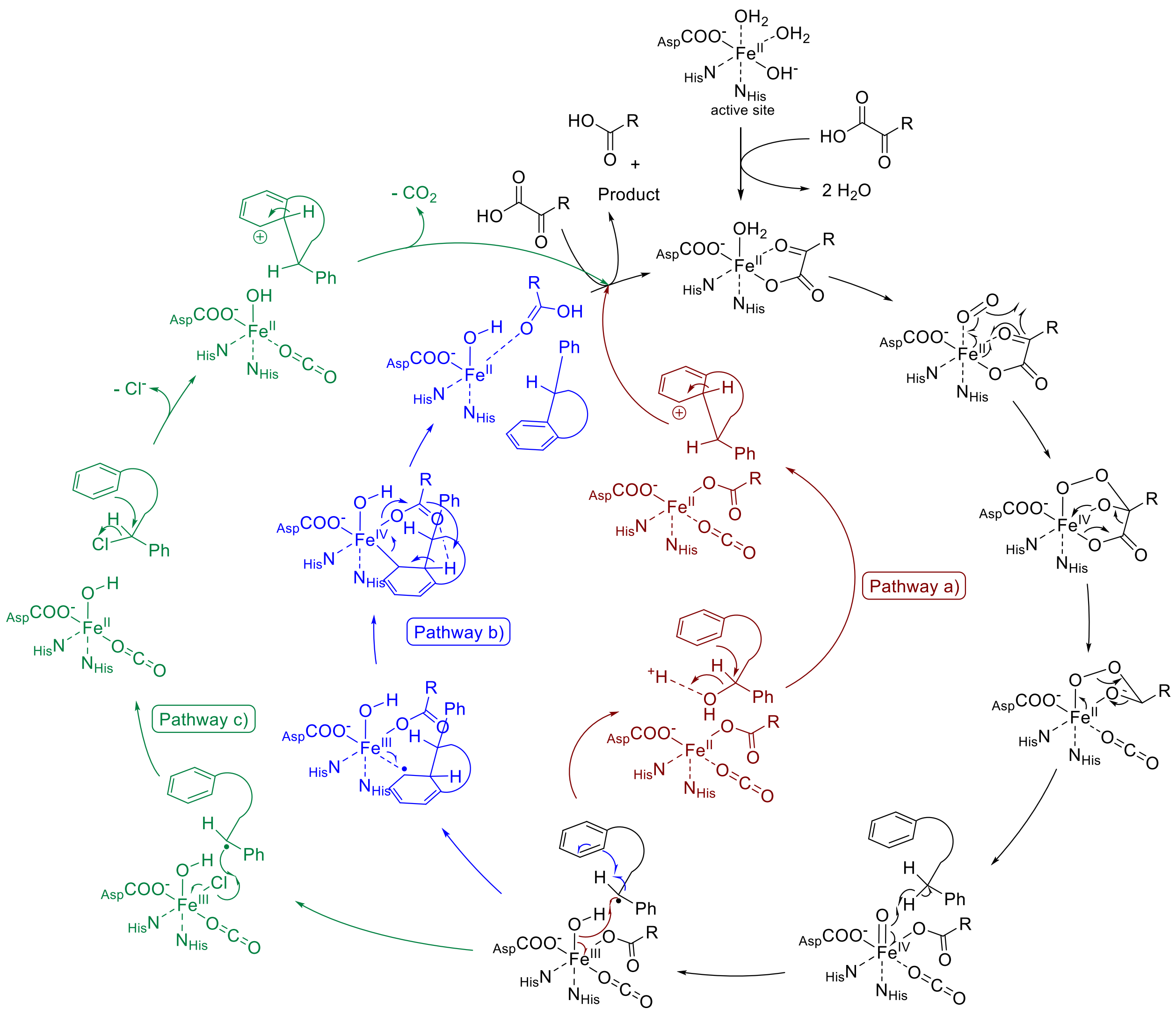

Figure SI01. Potential mechanisms for the biocatalytic cyclization step; pathway a): hydroxylation of benzylic position and subsequent Friedel-Crafts alkylation;[21] pathway b): radical cyclization;[21] pathway c): biocatalytic halogenation followed by a Friedel-Crafts alkylation.[22]

Optimization of Enzymatic Transformation

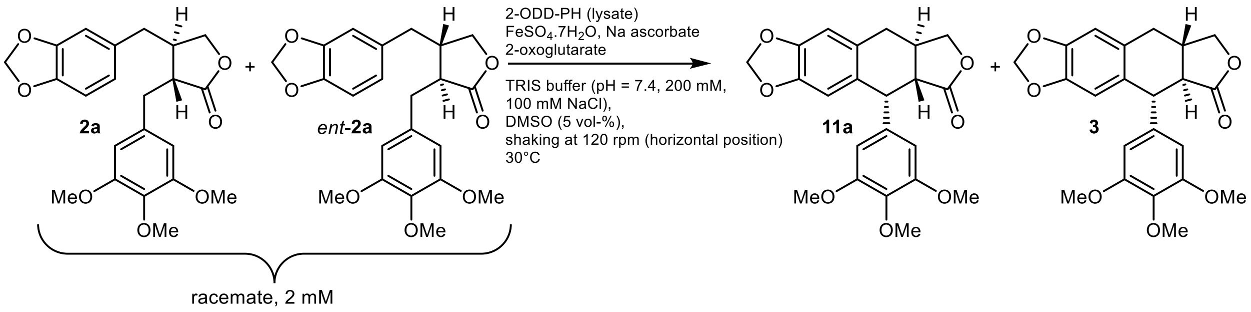

Scheme SI01. Reaction for the buffer optimization

Table SI02. Buffer systems

Entry	Buffer System (50 mM, 100 mM NaCl, pH =7.4)	Conv. [%][a]
1	P_i buffer (sodium salts)	75
2	MOPS / HCl	92
3	TRIS / HCl	91

Reaction conditions: 2 mM substrate *rac*-**2a**, 60 mM 2-oxoglutarate, 20 mM sodium ascorbate, $FeSO_4.7H_2O$ (0.1 eq.); [a] conversion was determined after 16 h of reaction time via peak area integration of the HPLC-UV chromatogram at 215 nm wavelength and refers to the sum of conversion towards **11a** and **3**.

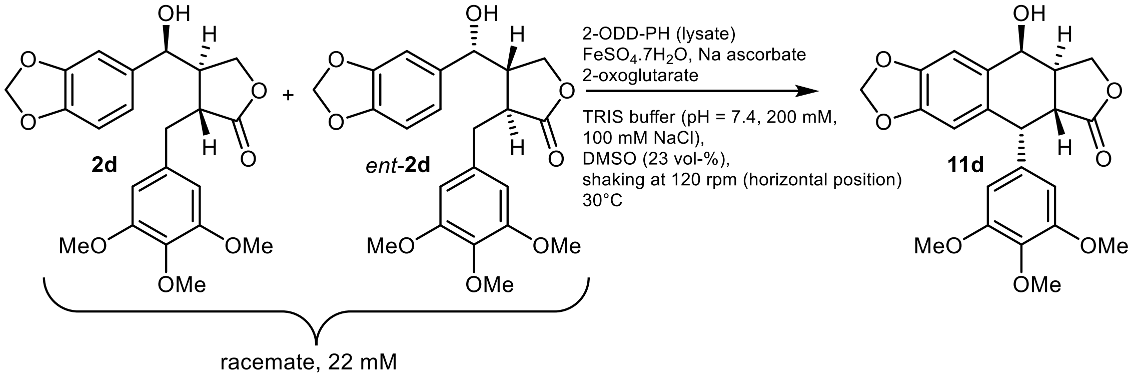

Scheme SI02. Reaction for all other enzyme optimization studies; standard conditions are given; alterations from standard conditions are noted in the tables below; lysate was prepared from 100 mg whole cells / mL buffer.

Table SI03. pH study

Entry	Buffer system (50 mM, 100 mM NaCl)	pH	HPLC peak areas [%][a]		
			**2d**	*ent*-**2d**	**11d**
1	Bis-Tris / HCl	6.0	14	43	43
2	Tris / HCl	**7.0**	**9**	**42**	**49**
3	Tris / HCl	8.0	12	42	46
4	Tris / HCl	8.5	22	45	34
5	Tris / HCl	9.0	38	48	14

Reaction conditions: 22 mM substrate *rac*-**2d**, CFE (44 vol-%), 2-oxoglutarate (12 eq.), sodium ascorbate (2 eq.), $FeSO_4.7H_2O$ (0.1 eq.); [a] determined after 16 h of reaction time at 215 nm wavelength at a chiral stationary phase (for details see experimental procedures).

Table SI04. Buffer strength

Entry	Tris / HCl buffer(100 mM NaCl) [mM]	HPLC peak areas [%][a]		
		**2d**	*ent*-**2d**	**11d**
1	100	7	41	52
2	**200**	**5**	**40**	**55**
3	500	11	42	47

Reaction conditions: 22 mM substrate *rac*-**2d**, CFE (44 vol-%), 2-oxoglutarate (12 eq.), sodium ascorbate (2 eq.), $FeSO_4.7H_2O$ (0.1 eq.); [a] determined after 16 h of reaction time at 215 nm wavelength at a chiral stationary phase (for details see experimental procedures).

Table SI05. DMSO concentration studies

Entry	DMSO [vol-%]	HPLC peak areas [%][a]		
		**2d**	*ent*-**2d**	**11d**
1	10	10	41	49
2	20	8	41	51
3	**23**	**3**	**39**	**58**
4	30	6	40	54
5	40	11	42	47

Reaction conditions: 22 mM substrate *rac*-**2d**, CFE (44 vol-%), 2-oxoglutarate (12 eq.), sodium ascorbate (2 eq.), $FeSO_4.7H_2O$ (0.1 eq.); [a] determined after 16 h of reaction time at 215 nm wavelength at a chiral stationary phase (for details see experimental procedures).

Table SI06. DMSO concentration studies

Entry	Cosolvent (23 %)	HPLC peak areas [%][a]		
		**2d**	*ent*-**2d**	**11d**
1	cyclohexane	47	50	3
2	1-butanol	34	48	18
3	diethylether	36	42	22
4	1-pentanol	26	46	28
5	1-octanol	21	40	39
6	toluene	13	41	46
7	Methyl-*tert*-butylether	9	39	52
8	**DMSO**	**5**	**38**	**57**

Reaction conditions: 22 mM substrate *rac*-**2d**, CFE (44 vol-%), 2-oxoglutarate (1.5 eq.), sodium ascorbate (1.6 eq.), $FeSO_4.7H_2O$ (0.1 eq.); [a] determined after 16 h of reaction time at 215 nm wavelength at a chiral stationary phase (for details see experimental procedures).

Table SI07. 2-Oxoglutarate concentration studies

Entry	2-oxolgutarate		HPLC peak areas [%][a]		
	[mM]	[Eq.]	**2d**	*ent*-**2d**	**11d**
1	**33**	**1.5**	**< 1**	**37**	**63**
2	66	3.0	2	38	60
3	132	6.0	3	39	58
4	264	12.0	8	41	51

Reaction conditions: 22 mM substrate *rac*-**2d**, sodium ascorbate (1.6 eq.), CFE (44 vol-%), $FeSO_4.7H_2O$ (0.1 eq.); [a] determined after 16 h of reaction time at 215 nm wavelength at a chiral stationary phase (for details see experimental procedures).

Table SI08. Sodium ascorbate concentration studies

Entry	Sodium ascorbate		HPLC peak areas [%][a]		
	[mM]	[Eq.]	**2d**	*ent*-**2d**	**11d**
1	0	0	51	49	< 1
2	8.8	0.4	14	42	44
3	17.6	0.8	3	39	58
4	**35.2**	**1.6**	**2**	**39**	**59**
5	70.4	3.2	15	42	43

Reaction conditions: 22 mM substrate *rac*-**2d**, CFE (44 vol-%), 2-oxoglutarate (1.5 eq.), $FeSO_4.7H_2O$ (0.1 eq.); [a] determined after 16 h of reaction time at 215 nm wavelength at a chiral stationary phase (for details see experimental procedures).

Table SI09. Temperature studies

Entry	Temperature [°C]	HPLC peak areas [%][a]		
		**2d**	*ent*-**2d**	**11d**
1	38	49	49	2
2	20	12	42	47
3	25	9	41	50
4	**18**	**7**	**38**	**55**
5	10	18	39	43

Reaction conditions: 22 mM substrate *rac*-**2d**, CFE (44 vol-%), 2-oxoglutarate (1.5 eq.), sodium ascorbate (1.6 eq.), $FeSO_4.7H_2O$ (0.1 eq.); [a] determined after 16 h of reaction time at 215 nm wavelength at a chiral stationary phase (for details see experimental procedures).

Table SI10. Studies on the influence of Fe^{II} concentration

Entry	$FeSO_4$		HPLC peak areas [%][a]		
	[mM]	[Eq.]	**2d**	*ent*-**2d**	**11d**
1	0	0	25	45	30
2	2.2	0.1	9	42	50
3	4.4	0.2	10	42	48
4	6.6	0.3	7	41	52
**5**	**11**	**0.5**	**6**	**41**	**54**

Reaction conditions: 22 mM substrate *rac*-**2d**, 2-oxoglutarate (1.5 eq.), sodium ascorbate (1.6 eq.), $FeSO_4.7H_2O$ (0.1 eq.); [a] determined after 16 h of reaction time at 215 nm wavelength at a chiral stationary phase (for details see experimental procedures).

Table SI11. Substrate concentration

Entry	Substrate Concentration		HPLC peak areas [%][a]		
	[mM]	[g/L]	**2d**	*ent*-**2d**	**11d**
1	22	9.2	< 1	37	63
2	30	12.3	5	40	65
3	37	15.4	12	42	46

Reaction conditions: 22 mM substrate *rac*-**2d**, CFE (44 vol-%), 2-oxoglutarate (1.5 eq.), sodium ascorbate (1.6 eq.), $FeSO_4.7H_2O$ (0.1 eq.); [a] determined after 16 h of reaction time at 215 nm wavelength at a chiral stationary phase (for details see experimental procedures).

Control Experiments and Kinetic Study

Table SI12a. Control experiments

Entry	Control	HPLC peak areas [%][a]		
		**2d**	*ent*-**2d**	**11d**
1	Standard conditions	< 1	37	63
2	Buffer + $Fe^{II}SO_4$ instead of lysate	50	50	< 1
3	Lysate from empty expression host [*E. coli* BL21(DE3)]	50	50	< 1
4	Standard conditions but lysate was heat shocked (80°C, 30 min)	50	50	< 1
5	Purified enzyme	14	43	43

Standard reaction conditions: 22 mM substrate *rac*-**2d**, CFE (44 vol-%), 2-oxoglutarate (1.5 eq.), sodium ascorbate (1.6 eq.), $FeSO_4.7H_2O$ (0.1 eq.); [a] determined after 16 h of reaction time at 215 nm wavelength at a chiral stationary phase (for details see experimental procedures).

Table SI12b. Control experiments for enzyme catalyzed ring closure

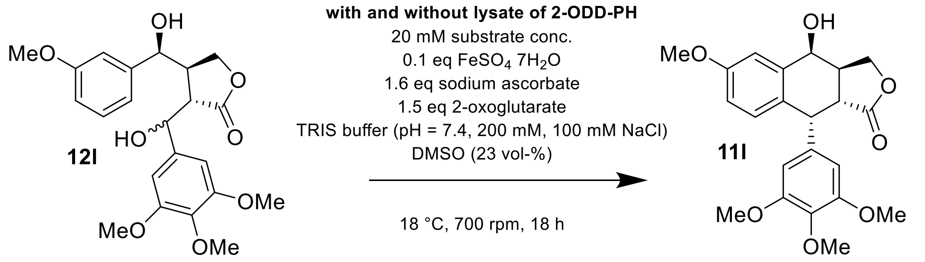

Entry	Conditions	HPLC area [%][a]	
		**12l**	**11l**
1	With lysate of 2-ODD-PH (44 vol-%)	>99	not detected
2	Buffer instead of lysate of 2-ODD-PH	>99	not detected

Reaction conditions: 20 mM concentration of **12l**, $FeSO_4.7H_2O$ (0.1 eq.), sodium ascorbate (1.6 eq.), 2-oxoglutarate (1.5 eq.), TRIS buffer (pH = 7.4, 200 mM, 100 mM NaCl), 23 vol-% DMSO, incubation at 18°C and 700 rpm for 18 h; [a] determined via peak area integration of the HPLC chromatograms; **11l** was not detected in any of these experiments (for details see HPLC chromatogram section).

Table SI13a. Time study on analytical scale (1 mg substrate)

Entry	Reaction time [min (h)]	HPLC peak areas [%][a]			ee of 2d [%]
		**2d**	*ent*-**2d**	**11d**	
1	2	47	49	4	3
2	5	44	49	7	5
3	11	33	47	20	17
4	15	28	46	27	25
5	30 (0.5)	12	43	45	27
6	60 (1.0)	7	41	52	73
7	120 (2.0)	4	40	56	82
8	210 (3.5)	3	40	57	84
**9**	**300 (5.0)**	**2**	**40**	**58**	**89**
10	1260 (21)	5	40	55	79

Reaction conditions: 22 mM substrate *rac*-**2d** (1.0 mg batch size), CFE (44 vol-%), 2-oxoglutarate (1.5 eq.), sodium ascorbate (1.6 eq.), $FeSO_4.7H_2O$ (0.1 eq.); [a] determined at 215 nm wavelength at a chiral stationary phase (for details see experimental procedures).

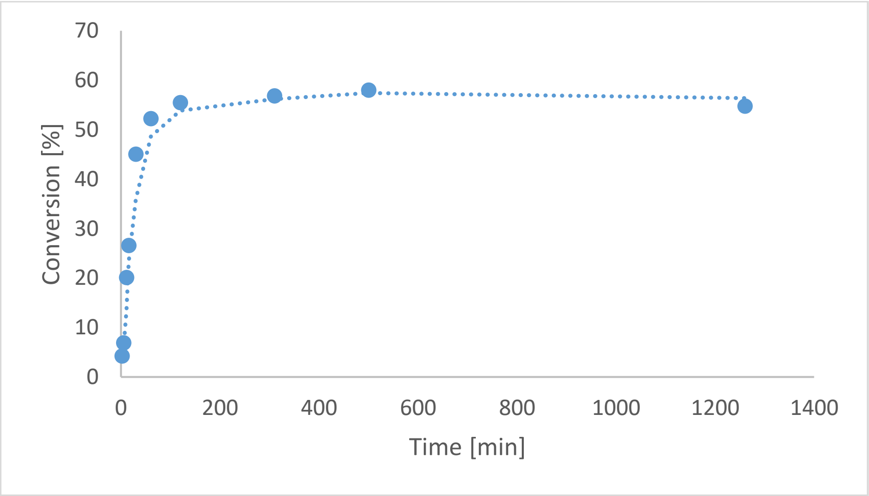

Figure SI02a. Time study of the 2-ODD-PH catalyzed reaction with substrate *rac*-**2d** on analytical scale (1 mg substrate).

Table SI13b. Time study on preparative scale (100 mg substrate)

Entry	Reaction time [min (h)]	HPLC peak areas [%][a]			ee of 2d [%]
		**2d**	*ent*-**2d**	**11d**	
1	30 (0.5)	39	48	13	10
2	60 (1.0)	30	46	23	21
3	120 (2.0)	17	43	40	44
4	180 (3.0)	10	41	49	62
5	240 (4.0)	6	40	54	72
6	300 (5.0)	5	38	57	78
7	360 (6.0)	4	38	58	80
8	1080 (18.0)	2	35	62	87

Reaction conditions: 22 mM substrate *rac*-**2d** (100 mg batch size), CFE (44 vol-%), 2-oxoglutarate (1.5 eq.), sodium ascorbate (1.6 eq.), $FeSO_4.7H_2O$ (0.1 eq.); [a] determined at 215 nm wavelength at a chiral stationary phase (for details see experimental procedures).

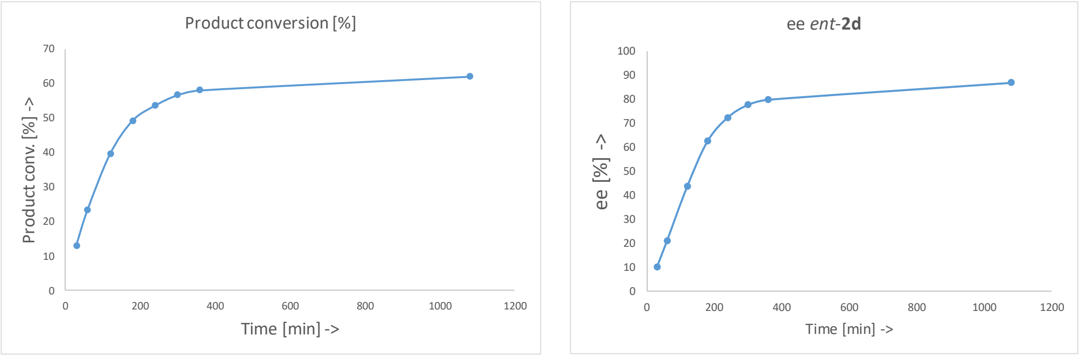

Figure SI02b. Time study: conversion of **2d** and the ee of *ent*-**2d** of the 2-ODD-PH catalyzed reaction on preparative scale (100 mg)

Substrate Conversions

Table SI14a. Conversions towards dihydroxylated substrates on analytical scale

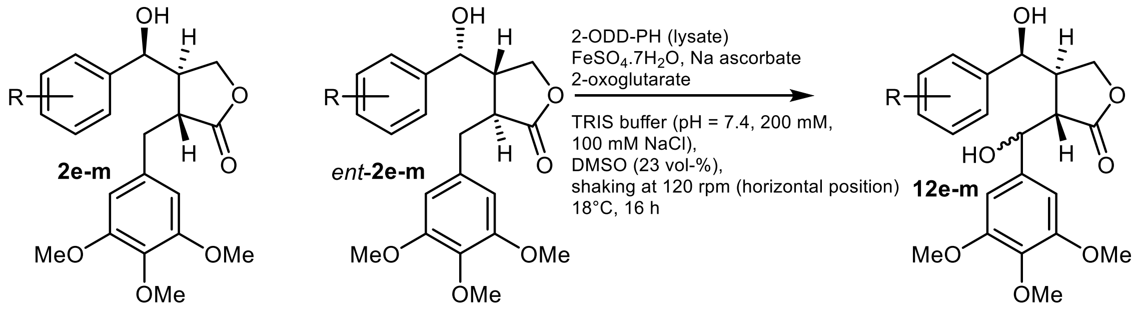

Entry	Substrate	HPLC peak areas [%][a]			ee of remaining 2e-m
		**2e-m**	*ent*-**2e-m**	**12e-m**	[%]
1	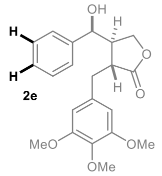	3	53	44	87
2	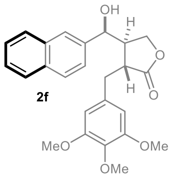	< 1	50	50	> 99
3	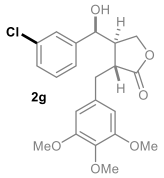	< 1	57	43	> 99
4	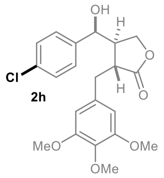	3	56	43	90
5	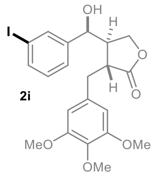	2	47	51	92
6	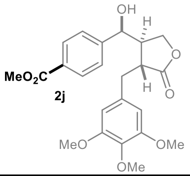	31	51	18	24

Reaction conditions: 22 mM racemic substrates (**2e-m** and **SI-1**), 2-oxoglutarate (1.5 eq.), sodium ascorbate (1.6 eq.), $FeSO_4.7H_2O$ (0.1 eq.); [a] determined at 215 nm wavelength at a chiral stationary phase (for details see experimental procedures); [b] two products were observed; [c] no upscale reaction has been performed for substrate **SI-1**; [c] **11l** was isolated as main product.

Table SI14a – continued.

Entry	Substrate	HPLC peak areas [%][a]			ee of remaining 2e-m
		**2e-m**	*ent*-**2e-m**	**12e-m**	[%]
7	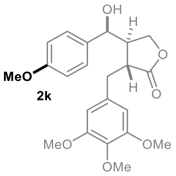 **2k**	12	54	34	64
8[c]	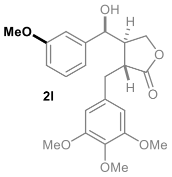 **2l**	No data given due to overlap of peaks, see HPLC section			
9	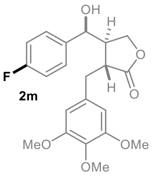 **2m**	2	52	46	93
10[c]	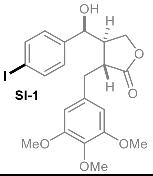 **SI-1**	14	52	34	57

Reaction conditions: 22 mM racemic substrates (**2e-m** and **SI-1**), 2-oxoglutarate (1.5 eq.), sodium ascorbate (1.6 eq.), $FeSO_4.7H_2O$ (0.1 eq.); [a] determined at 215 nm wavelength at a chiral stationary phase (for details see experimental procedures); [b] two products were observed; [c] no upscale reaction has been performed for substrate **SI-1**; [c] **11l** was isolated as main product.

Table SI14b. Detailed data for all upscale experiments

*Upscale of yatein **2a***

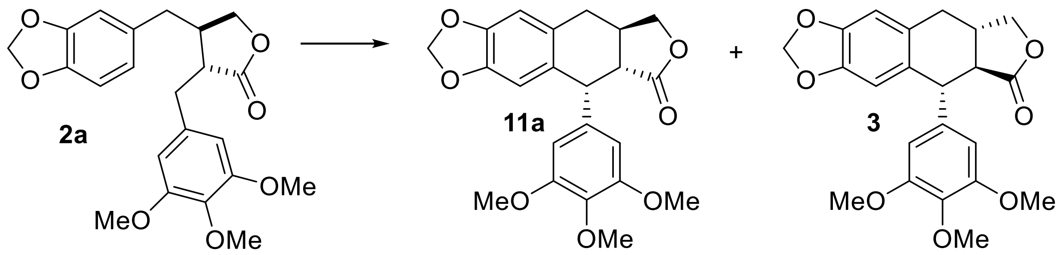

Entry	Comp. #	HPLC area [%][a]			Conv.[b] [%]	Isolated yield [%]			ee of 2a [%]	d.r.[e] of	
		**2a**	**11a**	**3**		**2a**	**11a**	**3**		**11a**	**3**
1	**2a**	37	26	37	63	26	19	20	9	>95:<5	>95:<5

[a] peak area integration values of the HPLC-UV chromatogram at 215 nm; [b] conversion determined based on peak area integration of the HPLC-UV chromatogram at 215 nm; [c] isolated yields of chromatographically pure material; [d] the ee was determined via HPLC-UV on a chiral stationarly phase (for details see Table SI15); [e] determined via NMR spectra.

Table SI14b – continued.

*Upscale of substrates **2b-m***

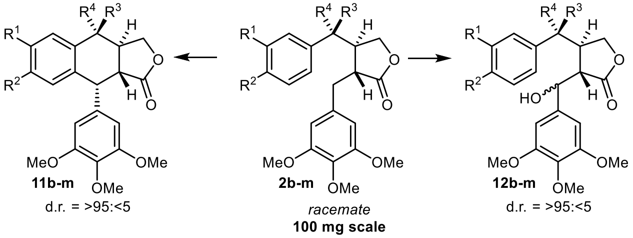

Entry	Comp. #	R^1 =	R^2 =	R^3 =	R^4 =	HPLC area [%][a]			Conv.[b] [%]	Conv.[i] [%]	Isolated yield [%]			ee of 2b-m [%]	d.r.[e] of	
						2b-m	11b-m	12b-m			2b-m	11b-m	12b-m		11b-m	12b-m
1	**2b**	OCH_2O		H	OH	71	-	29	30	n.d.	40	-	15	42	-	>95:<5
2	**2d**	OCH_2O		OH	H	47	53	-	40	43	45	39	-	72	>95:<5	
3	**2c**	OMe	OMe	OH	H	85	12	3	[h]	[h]	57	7	4	23	>95:<5	>95:<5
4	**2e**	H	H	OH	H	55	-	45	48	53	44	-	32	93	-	>95:<5
5	**2f**	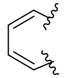		OH	H		[f]		47	[f]	22	-	22	89	-	>95:<5
6	**2g**	Cl	H	OH	H	55	-	45	49	50	37	-	43	95	-	>95:<5
7	**2h**	H	Cl	OH	H	60	-	40	39	42	50	-	30	63	-	>95:<5
8	**2i**	I	H	OH	H	49	-	51	45	48	41	-	40	82	-	>95:<5
9	**2j**	H	CO_2Me	OH	H	79	-	21	18	19	61	-	16	21	-	>95:<5
10	**2k**	H	OMe	OH	H	64	-	34	39	45	42	-	34	63	-	>95:<5
11	**2l**	OMe	H	OH	H		[g]		[h]	[g]	39	33	2	92	>95:<5	>95:<5
12	**2m**	H	F	OH	H	50	-	50	49	53	39	-	27	97	-	>95:<5

n.d. = not determined; [a] peak area integration values of the HPLC-UV chromatogram at 215 nm; [b] conversion determined based on the ee of a and the assumption of a kinetic resolution with E > 200; [c] isolated yields of chromatographically pure material; [d] the ee was determined via HPLC-UV on a chiral stationary phase (for details see Table SI15); [e] determined via NMR spectra; [f] no conversion could be determined due to overlap of substrate **2f** with the DMSO cosolvent; [g] not determined due to overlap of one enantiomer of substrate **2l** with **11l**; [h] not determined due to the formation of the hydroxylated (**12c** and **12l**) and the ring closed products (**11c** and **11l**); [i] conversion was determined via HPLC-UV calibration.

Substrates Showing No Conversion

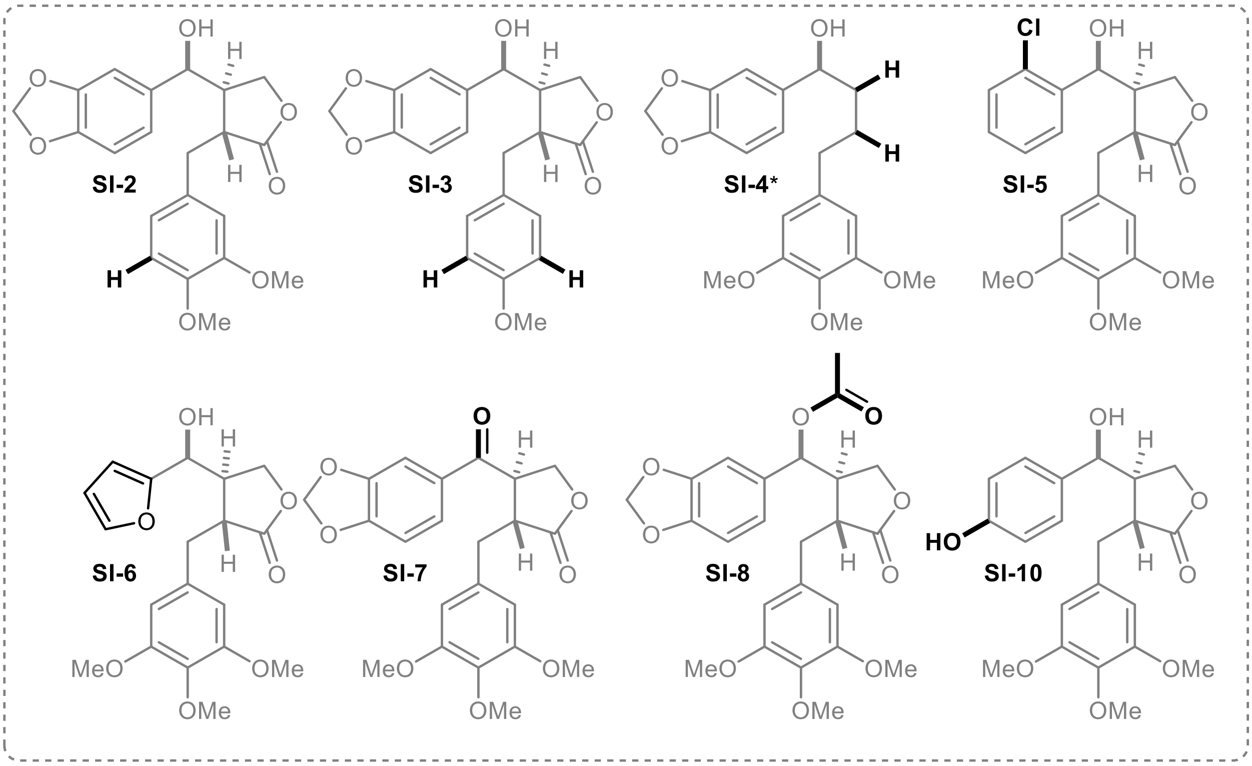

Figure SI03. Substrates showing no conversion under 2-OOD-PH catalysis; *contains ca. 10% of regioisomer **SI-29** (see experimental section).

Experimental Procedures

General Information.

All chemicals were purchased from Sigma Aldrich or Acros Organics and were used as received. All solvents were purchased from Roth. Dry THF was freshly distilled from sodium/benzophenone. All moisture sensitive reactions were operated using standard Schlenk techniques with dry argon. Biocatalytic reactions and rehydration of enzymes were accomplished in a HT Infors Unitron AJ 260 shaker at 120 rpm and 30 °C (horizontal position). Centrifugation was done at 13000 rpm in a Heraeus Biofuge pico or at 4000 rpm in a Heraeus Biofuge primo. NMR spectra were recorded on a Bruker NMR unit at 300 (^{1}H) and 75 (^{13}C) MHz, shifts are given in ppm and coupling constants (*J*) are given in Hz. High resolution mass spectra were recorded on a Agilent 6230 TOF LC/MS using ESI (positive mode, capillary voltage 3.5 kV) or APCI (negative mode, 5.0 kV) methods. Chiral HPLC analysis was performed on a Shimadzu HPLC system *n*-heptane/2-PrOH as eluent and Daicel columns (indicated below) as chiral stationary phase.

The gene encoding the 2-ODD enzyme was synthesized by GeneArt (Invitrogen) and transformed into the *E. coli* BL21 (DE3) expression host according to the cells' manual. Cell lysis via sonication was achieved using a Sonics & Materials Vibra Cell CV26 (13 mm tip).

High resolution mass spectra were recorded on an Agilent 6230 TOF LC/MS using ESI (positive mode, capillary voltage 1.6 kV) or APCI (negative mode, 5.0 kV) methods. Chiral HPLC analysis was performed on a Shimadzu HPLC system with n-heptane/2-PrOH as eluent. Optical rotation values were measured on a Perkin Elmer Polarimeter 341 or an Anton Paar MCP5100 unit.

Bromolactone **7** was prepared according to literature.[23]

DNA sequence of the biocatalyst

ATG GGT AGC ACA GCT CCA CTT AGA TTA CCT GTG ATT GAT CTG AGT ATG AAG AAC TTA AAG CCG GGT ACA ACC AGC TGG AAC TCG GTG AGG ACT CAA GTG CGA GAG GCT CTC GAA GAA TAC GGT TGC TTT GAA GCA GTA ATC GAT GCA GTA AGC CCT GAG CTC CAA AAG GCT GTG TGT AAC AAA GGT CAC GAG CTC TTA AAT TTA CCT CTG GAA ACT AAG ATG TTA AAC GGT AAC AAA CCT GAA TAT GAT GGT TTT ACT AGC ATT CCT AAC CTA AAC GAA GGG ATG GGT GTA GGG CGT ATT ACA GAT TTA GAA AAA GTG GAG AGA TTT ACC AAT TTA ATG TGG CCA GAG GGG AAT AAG GAT TTT TGT GAA ACC GTA TAT AGC TAT GGG AAA CGC ATG GCT GAG GTA GAC CAC ATT TTA AAA ATG ATG GTG TTT GAG AGC TTT GGT ATG GAG AAG CAC TTT GAC TCG TTT TGC GAA AGC ACA AAT TAC TTA CTA CAT TTT ATG CGT TAC CAG CAG CCT GGG AAG GAT GGT CGC AGC CCG GCA TTA TCG TTA CAT AAG GAC AAG TCG ATC TTA ACT ATT GTG AAC CAG AAT GAT GTA AAG GGT TTA GAA TTT GAA ACT AAG GAT GGT GAA TGG ATT CTG CCG ACA GCA GAC AAC CAT ATT GTG TTA CTT GGT GAC TGC TTT ATG GCT TGG TCG AAT GGT CGT CTG CAT AGC CCG TTA CAC CGA GTA ACT TTA GTA GCT AAC CAA GCT AGA CTG AGC ACA AGC TCG TTT TCG TTT CCT AAG GAC ATT ATT GAG ACT CCG GCT GAG CTC GTA GAT GAA GAG CAT CCG TTA CTT TTT AAT CCA TTT GAG ATT

ACT GAG TTA TTA GCA TAC TGT TTT ACA AAA GAG GGT GCT AAG GCT GTA TGT GAC CTA AAG CAG TAC AAG GCT TAC ACA GGT GCT TGA

Preparation of the biocatalyst

The obtained plasmid of the 2-ODD enzyme encoding the enzyme and a C-terminal His_6-tag was transformed into *E. coli* BL21(DE3) cells according to the cell's manufacturer's manual and grown on LB agar plates containing 100 µg/mL ampicillin. A single colony was picked to inoculate 10-20 mL of LB medium (100 µg/mL ampicillin) and this over night culture (ONC) was shaken at 120 rpm and 37°C in horizontal position overnight. LB medium (100 µg/mL ampicillin) was inoculated with this ONC culture (9 mL ONC per 1 L of medium) and the bacteria were grown at 37°C and shaking at 120 rpm until an OD_{600} of 1.6 was reached. IPTG was added to a final concentration of 1.0 mM to induce expression and the cells were cultivated at 20°C and 120 rpm of shaking overnight. The bacteria were harvested by centrifugation (8000 rpm, 4°C, 10 min), resuspended and washed with TRIS buffer (pH = 7.4, 200 mM, 100 mM NaCl, ca. 5 mL per L cells culture) and centrifuged again (8000 rpm, 4°C, 10 min). The pellet was resuspended in TRIS buffer (pH = 7.4, 200 mM, 100 mM NaCl, ca. 5 mL per L cells culture) and freeze dried for further storage and use.

Cell lysis

The whole cells containing the overexpressed 2-ODD enzyme were resuspended in TRIS buffer (pH = 7.4, 200 mM, 100 mM NaCl, 100 mg cells per mL, 2.6 mg $FeSO_4.7H_2O$ per mL) for 5 min at 30°C and 120 rpm (shaking in horizontal position). The suspension was cooled with ice and sonicated (30% amplitude, 1 s pulse on, 3 s pulse off, 5 min total pulse time). The cell-free extract (CFE) was obtained after centrifugation (18000 rpm, 4°C, 10 min).

Enzyme purification

The CFE (1 g cells in 10 mL buffer) were loaded on a 5 mL His trap™ column (GE Healthcare). The column was washed with buffer A (20 mL, TRIS, pH = 7.4, 100 mM, 200 mM NaCl, 20 mM imidazole). The protein was eluted using a gradient from buffer A (TRIS, pH = 7.4, 200 mM, 100 mM NaCl, 20 mM imidazole) to buffer B (TRIS, pH = 7.4, 200 mM, 100 mM NaCl, 300 mM imidazole) over 20 min at a 1 mL/min flow rate. Fractions were collected at a volume of 2 mL and the protein eluted from 9-18 min run time. The fractions were collected, concentrated via viva spin tubes (5 mL, Bio-Rad, 10 kDa cutoff) to 1-2 mL end volume using centrifugation (8000 rpm, 4°C) and desalted using a PD10 desalting column (GE Healthcare) according to the column's manual. The obtained purified protein solution (3 mL total volume, 11 mg protein/mL) was aliquoted into 1.5 mL Eppendorf vials and frozen in liquid nitrogen prior to storage at -80°C.

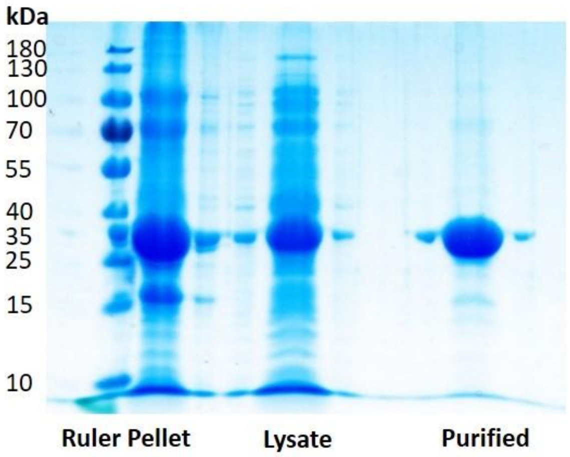

Figure SI04. SDS page of purified enzyme

General procedure for the allylation of aldehydes with bromolactone 7.

A 25 mL round bottom flask, equipped with a magnetic stir bar, was charged with zinc dust (2.0 eq), NH_4Cl (4.0 eq) and the corresponding aldehyde (1.0 eq). A mixture of dimethoxyethane and toluene (1:1) was added, followed by bromolactone **7** (1.2 eq). The flask was closed with a stopper and the reaction mixture was stirred over night at room temperature and 700 rpm. The gray to colorless slurry was mixed with saturated, aqueous NH_4Cl-solution and the mixture was extracted with EtOAc (3 x 20 mL). The combined organic phase was dried over Na_2SO_4, the solvent was removed under reduced pressure and the crude product was purified by flash column chromatography (cyclohexane/EtOAc, ratios are given below) to give the corresponding allylated lactone.

rac-4-[benzo[d][1,3]dioxol-5-yl(hydroxy)methyl]-3-methylenedihydrofuran-2(3*H*)-one (8).

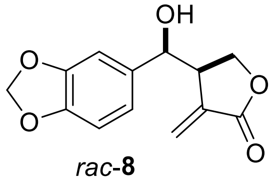

rac-**8**

Batch: zinc dust (< 10 µm, 261.6 mg, 4.0 mmol), ammonium chloride (427.9 mg, 8.0 mmol), piperonal (300 mg, 2.0 mmol), allylic bromide **7** (424.8 mg, 2.4 mmol) in toluene/dimethoxyethane 1/1 (5 mL). The obtained crude product was purified via flash chromatography (SiO_2, toluene/EtOAc 10/1) to give the title compound as a white solid (491.1 mg, 1.98 mmol, 99%).

Mp: 107-109 °C (from $CDCl_3$); ^{1}H-NMR (300 MHz, $CDCl_3$): 6.84 (s, 1H), 6.78 (s, 2H), 6.35 (d, J = 2.1, 1H), 5.97 (s, 2H), 5.86 (d, J = 2.1, 1H), 4.59 (d, J = 8.0, 1H), 4.15 (dd, J_1 = 9.6, J_2 = 8.3, 1H), 4.01 (dd, J_1 = 9.6, J_2 = 4.5, 1H), 3.39-3.30 (m, 1H), 2.25 (br s, 1H); ^{13}C-NMR (75 MHz, $CDCl_3$): 170.8, 148.3, 147.9, 135.2, 134.8, 125.6, 120.4, 108.4, 106.8, 101.4, 75.6, 67.6, 45.6; IR (film) ῦ = 3408 (br), 1733, 1488, 1443, 1396, 1379, 1248, 1144, 1039, 1005, 958, 930, 821; HRMS(ESI): *m/z*: calc. for $C_{13}H_{13}O_5^+$: 249.0758 [M+H]$^+$, found: 249.0756.

rac-4-[(3,4-dimethoxyphenyl)(hydroxy)methyl]-3-methylenedihydrofuran-2(3*H*)-one (SI-12).

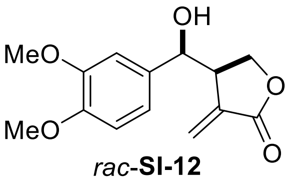
rac-**SI-12**

Batch: zinc dust (< 10 µm, 261.6 mg, 4.0 mmol), ammonium chloride (427.9 mg, 8.0 mmol), 3,4-dimethoxybenzaldehyde (332.3 mg, 2.0 mmol), allylic bromide **7** (424.8 mg, 2.4 mmol) in toluene/dimethoxyethane 1/1 (5 mL). The obtained crude product was purified via flash chromatography (SiO_2, cyclohexane/EtOAc 1/1 to 2/1) to give the title compound as a white solid (491.1 mg, 1.98 mmol, 99%).

Mp: 70-74 °C (from $CDCl_3$); ^{1}H-NMR (300 MHz, $CDCl_3$): 6.88 – 6.85 (m, 3H), 6.36 (d, J = 1.9, 1H), 5.85 (dd, J_1 = 2.1, J_2 = 0.7 Hz, 1H), 4.62 (d, J = 8.0 Hz, 1H), 4.15 (dd, J_1 = 9.6, J_2 = 8.3 Hz, 1H), 4.02 (dd, J_1 = 9.6, J_2 = 4.4 Hz, 1H), 3.88 (s, 3H), 3.87 (s, 3H), 3.43 – 3.33 (m, 1H), 2.34 (br s, 1H); ^{13}C-NMR (75 MHz, $CDCl_3$): 170.8, 149.4, 149.3, 135.3, 133.4, 125.5, 119.1, 111.1, 109.4, 75.7, 67.7, 56.1, 45.7; IR (film) ῦ = 3249 (br), 1760, 1751, 1516, 1507, 1461, 1260, 1237, 1143, 1123, 1022, 1003, 988, 950, 912, 856, 818, 725, 649; HRMS(ESI): m/z: calc. for $C_{14}H_{17}O_5^+$: 247.0965 $[M+H]^+$, found: 247.0966.

rac-4-[hydroxy(phenyl)methyl]-3-methylenedihydrofuran-2(3*H*)-one (SI-13).

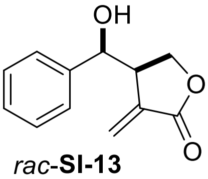
rac-**SI-13**

Batch: zinc dust (< 10 µm, 261.6 mg, 4.0 mmol), ammonium chloride (427.9 mg, 8.0 mmol), benzaldehyde (212.0 mg, 2.0 mmol), allylic bromide **7** (424.8 mg, 2.4 mmol) in toluene/dimethoxyethane 1/1 (5 mL). The obtained crude product was purified via flash chromatography (SiO_2, cyclohexane/EtOAc 3/1 to 2/1) to give the title compound as a yellow oil (315.1 mg, 1.54 mmol, 77%).

^{1}H-NMR (300 MHz, $CDCl_3$): 7.41 – 7.30 (m, 5H), 6.33 (d, J = 2.4 Hz, 1H), 5.76 (dd, J_1 = 2.2, J_2 = 0.7 Hz, 1H), 4.69 (d, J = 7.6 Hz, 1H), 4.15 (dd, J_1 = 9.6, J_2 = 8.2 Hz, 1H), 4.05 (dd, J_1 = 9.6, J_2 = 4.4 Hz, 1H), 3.40 (dddd, J_1 = 10.2, J_2 = 8.0, J_3 = 4.5, J_4 = 2.3 Hz, 1H), 2.54 (br s, 1H); ^{13}C-NMR (75 MHz, $CDCl_3$): 170.9, 140.8, 135.0, 128.9, 128.7, 126.7, 125.6, 75.7, 67.7, 45.5; IR (film) ῦ = 3456 (br), 1729, 1659, 1453, 1414, 1381, 1319, 1271, 1217, 1189, 1136, 1091, 1070, 1041, 1009, 974, 961, 948, 919, 818, 784, 762, 734, 713; HRMS(ESI): *m/z*: calc. for $C_{12}H_{13}O_3^+$: 205.0859 $[M+H]^+$, found: 205.0859.

rac-4-[hydroxy(naphthalen-2-yl)methyl]-3-methylenedihydrofuran-2(3*H*)-one (SI-14).

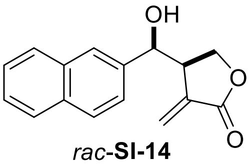
rac-**SI-14**

Batch: zinc dust (< 10 µm, 261.6 mg, 4.0 mmol), ammonium chloride (427.9 mg, 8.0 mmol), 2-naphtaldehyde (312.4 mg, 2.0 mmol), allylic bromide **7** (424.8 mg, 2.4 mmol) in toluene/dimethoxyethane 1/1 (5 mL). The obtained crude product was purified via flash chromatography (SiO_2, cyclohexane/EtOAc 2/1) to give the title compound as a yellow oil (396.7 mg, 1.56 mmol, 78%).

^{1}H-NMR (300 MHz, $CDCl_3$): 7.87 – 7.81 (m, 3H), 7.75 (s, 1H), 7.55 – 7.49 (m, 2H), 7.44 (dd, J_1 = 8.5, J_2 = 1.6 Hz, 1H), 6.34 (d, J = 2.2 Hz, 1H), 5.76 (d, J = 1.6 Hz, 1H), 4.82 (d, J = 7.6 Hz, 1H), 4.16-4.05 (m, 2H), 3.51 – 3.43 (m, 1H), 2.58 (s, 1H); ^{13}C-NMR (75 MHz, $CDCl_3$): 171.0, 138.1, 134.9, 133.4, 133.1, 129.0, 128.2, 127.9, 126.7, 126.6, 126.0, 125.7, 124.0, 75.8, 67.8, 45.3; IR (film) ṽ = 3436 (br), 1743, 1400, 1373, 1270, 1121, 1019, 950, 819, 746; HRMS(ESI): *m/z*: calc. for $C_{16}H_{14}O_3NH_4^+$: 272.1281 $[M+NH_4]^+$, found: 272.1280.

rac-4-[(3-chlorophenyl)(hydroxy)methyl]-3-methylenedihydrofuran-2(3*H*)-one (SI-15).

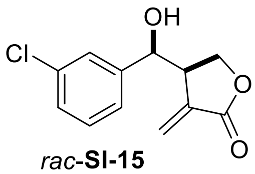

rac-**SI-15**

Batch: zinc dust (< 10 µm, 281.1 mg, 4.0 mmol), ammonium chloride (427.9 mg, 8.0 mmol), 3-chlorobenzaldehyde (281.1 mg, 2.0 mmol), allylic bromide **7** (424.8 mg, 2.4 mmol) in toluene/dimethoxyethane 1/1 (5 mL). The obtained crude product was purified via flash chromatography (SiO_2, cyclohexane/EtOAc 3/1 to 1/1) to give the title compound as a colorless oil (443.9 mg, 1.86 mmol, 93%).

^{1}H-NMR (300 MHz, $CDCl_3$): 7.37 (m, 1H), 7.35 – 7.30 (m, 2H), 7.22 (m, 1H), 6.37 (d, J = 2.3, 1H), 5.74 (d, J = 2.0, 1H), 4.72 (dd, J_1 = 7.4, J_2 = 3.1, 1H), 4.21 (dd, J_1 = 9.6, J_2 = 8.2, 1H), 4.09 (dd, J_1 = 9.7, J_2 = 4.1 Hz, 1H), 3.38 (dddd, J_1 = 10.2, J_2 = 8.4, J_3 = 4.3, J_4 = 2.2, 1H), 2.37 (d, J = 3.4, 1H); ^{13}C-NMR (75 MHz, $CDCl_3$): 170.6, 142.8, 135.0, 134.6, 130.3, 129.0, 126.9, 125.9, 124.9, 75.1, 67.6, 45.6; IR (film) ṽ = 3429 (br), 1741, 1597, 1574, 1475, 1409, 1375,, 1270, 1191, 1115, 1042, 1020, 1115, 1042, 1020, 999, 949, 883, 817, 783, 697; HRMS(ESI): *m/z*: calc. for $C_{12}H_{12}ClO_3^+$: 239.0469 $[M+H]^+$, found: 239.0470.

rac-4-[(4-chlorophenyl)(hydroxy)methyl]-3-methylenedihydrofuran-2(3*H*)-one (SI-16).

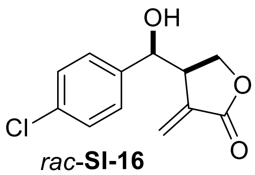

rac-**SI-16**

Batch: zinc dust (< 10 µm, 281.1 mg, 4.0 mmol), ammonium chloride (427.9 mg, 8.0 mmol), 4-chlorobenzaldehyde (281.1 mg, 2.0 mmol), allylic bromide **7** (424.8 mg, 2.4 mmol) in toluene/dimethoxyethane 1/1 (5 mL). The obtained crude product was purified via flash chromatography (SiO_2, cyclohexane/EtOAc 4/1 to 2/1) to give the title compound as a colorless oil (286.4 mg, 1.20 mmol, 60%).

^{1}H-NMR (300 MHz, $CDCl_3$): 7.41 – 7.34 (m, 2H), 7.33 – 7.27 (m, 2H), 6.37 (d, J = 2.3, 1H), 5.75 (dd, J_1 = 2.1, J_2 = 0.5, 1H), 4.72 (dd, J_1 = 7.4, J_2 = 3.4, 1H), 4.19 (dd, J_1 = 9.6, J_2 = 8.1, 1H), 4.07 (dd, J_1 = 9.7, J_2 = 4.1, 1H), 3.37 (tdt, J_1 = 8.4, J_2 = 4.3, J_3 = 2.2, 1H), 2.27 (d, J = 3.4, 1H); ^{13}C-NMR (75 MHz, $CDCl_3$): 170.6, 139.2, 134.8, 134.7, 129.2, 128.1, 125.8, 75.1, 67.5, 45.6; IR (film) ṽ = 3435 (br), 2976, 2912, 1737, 1490, 1399, 1375, 1270, 1188, 1116, 1088, 1038, 1012, 948, 819; HRMS(ESI): *m/z*: calc. for $C_{12}H_{11}ClO_3NH_4^+$: 256.0736 $[M+NH_4]^+$, found: 256.0735.

rac-4-[(3-Iodophenyl)(hydroxy)methyl]-3-methylenedihydrofuran-2(3H)-one (SI-17).

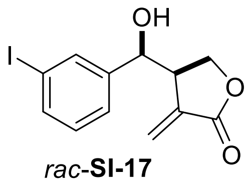

rac-**SI-17**

Batch: zinc dust (< 10 µm, 261.5 mg, 4.0 mmol), ammonium chloride (427.9 mg, 8.0 mmol), 3-iodobenzaldehyde (464.0 mg, 2.0 mmol), allylic bromide **7** (424.8 mg, 2.4 mmol) in toluene/dimethoxyethane 1/1 (5 mL). The obtained crude product was purified via flash chromatography (SiO_2, cyclohexane/EtOAc 4/1 to 2/1) to give the title compound as a colorless oil (425.0 mg, 1.29 mmol, 64%).

^{1}H-NMR (300 MHz, $CDCl_3$): 7.79 – 7.61 (m, 2H), 7.29 (d, J = 7.7 Hz, 1H), 7.12 (t, J = 7.8 Hz, 1H), 6.35 (s, 1H), 5.75 (d, J = 19.2 Hz, 1H), 4.66 (dd, J_1 = 7.3, J_2 = 3.5 Hz, 1H), 4.20 (dd, J_1 = 9.5, J_2 = 8.3 Hz, 1H), 4.07 (dd, J_1 = 9.6, J_2 = 4.1 Hz, 1H), 3.36 (ddd, J_1 = 9.9, J_2 = 7.7, J_3 = 2.0 Hz, 1H), 2.56 (br s, 1H); ^{13}C-NMR (CDCl3, 75 MHz): 170.6, 143.1, 137.8, 135.7, 134.7, 130.6, 126.0, 125.8, 94.9, 74.9, 67.6, 45.6; IR (film) ῡ = 3484 (br), 1731, 1655, 1588, 1410, 1271, 1217, 1106, 1081, 979, 842, 721; HRMS(ESI): *m/z*: calc. for $C_{12}H_{11}IO_3NH_4^+$: 348,0085 [M+NH$_4$]$^+$, found: 348,0091.

rac-Methyl 4-[hydroxy(4-methylene-5-oxotetrahydrofuran-3-yl)methyl]benzoate (SI-18).

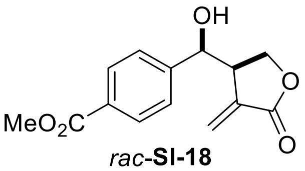

rac-**SI-18**

Batch: zinc dust (< 10 µm, 218.4 mg, 3.3 mmol), ammonium chloride (357.3 mg, 6.7 mmol), methyl 4-formylbenzoate (273.6 mg, 1.7 mmol), allylic bromide **7** (352.3 mg, 2.0 mmol) in toluene/dimethoxyethane 1/1 (4.2 mL). The obtained crude product was purified via flash chromatography (SiO2, cyclohexane/EtOAc 1/1) to give the title compound as a white solid (352.1 mg, 1.3 mmol, 80%).

Mp: 112-116 °C (from EtOAc); ^{1}H-NMR (300 MHz, $CDCl_3$): 8.09 – 7.96 (d, J = 8.5 Hz, 2H), 7.49 – 7.35 (d, J = 8.4 Hz, 2H), 6.32 (d, J = 2.3 Hz, 1H), 5.62 (d, J = 2.1, 1H), 4.83 – 4.81 (d, J = 6.9 Hz, 1H), 4.25 – 4.06 (m, 2H), 3.91 (s, 3H), 3.40 (m, 1H), 2.48 (br s, 1H) ; ^{13}C-NMR (75 MHz, $CDCl_3$): 170.7, 166.8 145.7, 134.5, 130.4, 130.1, 126.7, 125.7, 75.2, 67.7, 52.4, 45.5; IR (film) ῡ = 3484 (broad), 1752, 1698, 1609, 1439, 1275, 1119, 961, 856, 766, 710; HRMS(ESI): *m/z*: calc. for $C_{14}H_{15}O_5^+$: 263.0914 [M+H]$^+$, found: 263.0913.

rac-4-[hydroxy(4-methoxyphenyl)methyl]-3-methylenedihydrofuran-2(3*H*)-one (SI-19).

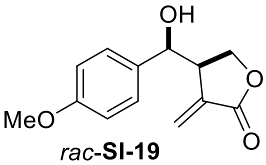

rac-**SI-19**

Batch: zinc dust (< 10 µm, 281.1 mg, 4.0 mmol), ammonium chloride (427.9 mg, 8.0 mmol), 4-methoxybenzaldehyde (243 µL, 272.3 mg, 2.0 mmol), allylic bromide **7** (424.8 mg, 2.4 mmol) in toluene/dimethoxyethane 1/1 (5 mL). The obtained crude product was

purified via flash chromatography (SiO_2, cyclohexane/EtOAc 2/1) to give the title compound as a yellow oil (380.0 mg, 1.62 mmol, 81%).

^{1}H-NMR (300 MHz, $CDCl_3$): 7.29 – 7.21 (m, 2H), 6.95 – 6.84 (m, 2H), 6.35 (dd, J_1 = 2.5, J_2 = 0.9 Hz, 1H), 5.85 (dd, J_1 = 2.2, J_2 = 0.8 Hz, 1H), 4.63 (d, J = 7.9 Hz, 1H), 4.13 (dd, J_1 = 9.6, J_2 = 8.3 Hz, 1H), 3.99 (dd, J_1 = 9.6, J_2 = 4.5 Hz, 1H), 3.81 (s, 3H), 3.39 (tdt, J_1 = 8.1, J_2 = 4.6, J_3 = 2.3 Hz, 1H); ^{13}C-NMR (75 MHz, $CDCl_3$): 170.9, 159.9, 135.4, 132.9, 128.0, 125.5, 114.4, 75.4, 67.6, 55.5, 45.6; IR (film) ῦ = 3431 (br), 2961, 2913, 2839, 1745, 1658, 1610, 1585, 1510, 1464, 1443, 1402, 1373, 1303, 1244, 1175, 1115, 1025, 950, 889, 871, 816, 792; HRMS(ESI): *m/z*: calc. for $C_{13}H_{15}O_4^+$: 235.0965 [M+H]$^+$, found: 235.0968.

rac-4-[hydroxy(3-methoxyphenyl)methyl]-3-methylenedihydrofuran-2(3*H*)-one (SI-20).

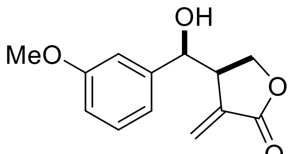

rac-**SI-20**

Batch: zinc dust (< 10 µm, 232.8 mg, 3.6 mmol), ammonium chloride (380.8 mg, 7.1 mmol), 3-methoxy benzaldehyde (242.3 mg, 1.8 mmol), allylic bromide **7** (377.0 mg, 2.1 mmol) in toluene/dimethoxyethane 1/1 (4.4 mL). The obtained crude product was purified via flash chromatography (SiO2, cyclohexane/EtOAc 3/1) to give the title compound as a pale yellow oil (271.9 mg, 1.2 mmol, 65%).

^{1}H-NMR (300 MHz, CDCl3): 7.32 – 7.24 (m, 1H), 6.93 – 6.82 (m, 3H), 6.34 (d, J = 2.1 Hz, 1H), 5.78 (dd, J = 2.1, 0.6 Hz, 1H), 4.66 (d, J = 7.6 Hz, 1H), 4.19 – 4.03 (m, 2H), 3.80 (s, 3H), 3.44 – 3.33 (m, 1H), 2.34 (br s, 1H); 13C-NMR (75 MHz, CDCl3): 170.9, 160.1, 142.5, 135.1, 130.0, 125.6, 118.9, 114.0, 112.3, 75.6, 67.7, 55.4, 45.5; IR (film) ῦ = 3446, 1743, 1655, 1600, 1585, 1488, 1257, 1118, 994, 949, 754, 676; HRMS(ESI): *m/z*: calc. for $C_{13}H_{15}O_4^+$: 235.0949 [M+H]$^+$, found: 235.0968.

rac-4-[(4-fluorophenyl)(hydroxy)methyl]-3-methylenedihydrofuran-2(3*H*)-one (SI-21).

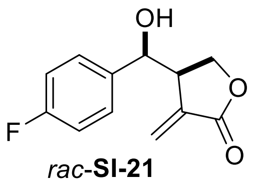

rac-**SI-21**

Batch: zinc dust (< 10 µm, 261.5 mg, 4.0 mmol), ammonium chloride (427.9 mg, 8.0 mmol), 4-fluorobenzaldehyde (248.2 mg, 2.0 mmol), allylic bromide **7** (424.8 mg, 2.4 mmol) in toluene/dimethoxyethane 1/1 (5 mL). The obtained crude product was purified via flash chromatography (SiO_2, cyclohexane/EtOAc 3/1 to 2/1) to give the title compound as a pale yellow solid (270.7 mg, 1.22 mmol, 61%).

Mp = 63-66 °C (from $CDCl_3$), ^{1}H-NMR (CDCl3, 300 MHz): 7.39 - 7.28 (m, 2H), 7.16 - 7.01 (m, 2H), 6.38 (d, J = 2.2 Hz, 1H), 5.79 (d, J = 1.6 Hz, 1H), 4.72 (d, J = 7.6 Hz, 1H), 4.18 (dd, J_1 = 9.6, J_2 = 8.2 Hz, 1H), 4.05 (dd, J_1 = 9.6, J_2 = 4.2 Hz, 1H), 3.38 (dddd, J_1 = 10.1, J_2 = 8.2, J_3 = 4.3, J_4 = 2.2 Hz, 1H), 2.20 (bs, 1H) ppm; ^{13}C-NMR (CDCl3, 75 MHz): 170.7, 162.8 (d, J = 247), 136.6 (d, J = 3.2),

134.9, 128.4 (d, J = 8.2), 125.6, 115.9 (d, J = 21.8), 75.0, 67.6, 45.7 ppm; IR (film) $\tilde{\nu}$ = 3467 (br), 1740, 1600, 1500, 1400, 1278, 1217, 1008, 960, 844, 813, 566. HRMS(ESI): *m/z*: calc. for $C_{12}H_{12}FO_3^+$: 223.0763 [M+H]$^+$, found: 223.0765.

rac-4-[(2-chlorophenyl)(hydroxy)methyl]-3-methylenedihydrofuran-2(3*H*)-one (SI-22).

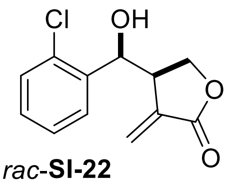

rac-**SI-22**

Batch: zinc dust (< 10 µm, 281.1 mg, 4.0 mmol), ammonium chloride (427.9 mg, 8.0 mmol), 2-chlorobenzaldehyde (281.1 mg, 2.0 mmol), allylic bromide **7** (424.8 mg, 2.4 mmol) in toluene/dimethoxyethane 1/1 (5 mL). The obtained crude product was purified via flash chromatography (SiO_2, cyclohexane/EtOAc 4/1 to 2/1) to give the title compound as a mixture of diastereoisomers (4:1) as a colorless oil (262.5 mg, 1.10 mmol, 55%).

^{1}H-NMR (300 MHz, $CDCl_3$): 7.49 (dd, J_1 = 7.3, J_2 = 2.2 Hz, 1H), 7.44 – 7.31 (m, 1H), 7.30 (ddd, J_1 = 7.1, J_2 = 6.0, J_3 = 2.0 Hz, 2H), 6.29 (d, *J* = 2.2 Hz, 1H), 5.31 (d, *J* = 2.0 Hz, 1H), 5.28 (d, *J* = 5.8 Hz, 1H), 4.34 (dd, J_1 = 9.4, J_2 = 7.8 Hz, 1H), 4.29 (dd, J_1 = 9.4, J_2 = 4.0 Hz, 1H), 3.54-3.46 (m, 1H), 2.14 (s, 1H); ^{13}C-NMR (75 MHz, $CDCl_3$): 171.0, 138.1, 134.0, 132.1, 129.8, 129.6, 128.6, 127.2, 125.6, 72.1, 68.6, 43.8; NMR-data is given for the major diastereoisomer; IR (film) $\tilde{\nu}$ = 3446 (br), 1738, 1466, 1443, 1412, 1382, 1272, 1189, 1136, 1098, 1022,976, 944, 8816, 765, 739, 704, 696, 621; HRMS(ESI): *m/z*: calc. for $C_{12}H_{12}ClO_3^+$: 239.0469 [M+H]$^+$, found: 239.0466.

rac-4-[furan-2-yl(hydroxy)methyl]-3-methylenedihydrofuran-2(3*H*)-one (SI-23).

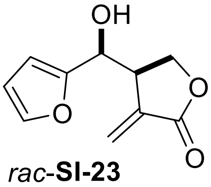

rac-**SI-23**

Batch: zinc dust (< 10 µm, 261.6 mg, 4.0 mmol), ammonium chloride (427.9 mg, 8.0 mmol), furfural (192 mg, 2.0 mmol), allylic bromide **7** (424.8 mg, 2.4 mmol) in toluene/dimethoxyethane 1/1 (5 mL). The obtained crude product was purified via flash chromatography (SiO_2, cyclohexane/EtOAc 1/1) to give the title compound as a pale yellow oil (168.4 mg, 0.96 mmol, 48%, d.r. = 8.5:1).

^{1}H-NMR (300 MHz, $CDCl_3$, major isomer): 7.39 – 7.38 (m, 1H), 6.36-6.31 (m, 3H), 5.73 (d, *J* = 2.2, 1H), 4.75 (d, *J* = 7.5, 1H), 4.33 (dd, J_1 = 9.7, J_2 = 8.4 Hz, 1H), 4.15 (dd, J_1 = 9.6, J_2 = 4.3 Hz, 1H), 3.62 – 3.53 (m, 1H), 2.81 (br s, 1H); ^{13}C-NMR (75 MHz, $CDCl_3$, major isomer): 170.7, 153.3, 142.7, 134.2, 125.7, 110.5, 108.2, 69.2, 67.5, 43.2; IR (film) $\tilde{\nu}$ = 3397 (br), 1743, 1403, 1267, 1117, 1007, 884, 816, 747; HRMS(ESI): *m/z*: calc. for $C_{10}H_{11}O_4^+$: 195.0652 [M+H]$^+$, found: 195.0653.

rac-4-[Hydroxy(4-hydroxyphenyl)methyl]-3-methylenedihydrofuran-2(3H)-one (SI-25).

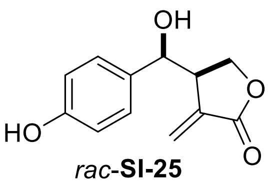

rac-**SI-25**

Batch: zinc dust (< 10 µm, 261.6 mg, 4.0 mmol), ammonium chloride (427.9 mg, 8.0 mmol), 4-hydroxybenzaldehyde (244.2 mg, 2.0 mmol), allylic bromide **7** (424.8 mg, 2.4 mmol) in toluene/dimethoxyethane 1/1 (5 mL). The obtained crude product was

purified via flash chromatography (SiO_2, cyclohexane/EtOAc 1/1) to give the title compound as a pale yellow oil (268.7 mg, 1.22 mmol, 61%).

^{1}H-NMR (300 MHz, acetone-d6): 8.33 (s, 1H), 7.27 – 7.22 (m, 2H), 6.84 – 6.79 (m, 2H), 6.12 (dd, J_1 = 2.5, J_2 = 1.3 Hz, 1H), 5.62 (dd, J_1 = 2.2, J_2 = 1.3 Hz, 1H), 4.76 (dd, J_1 = 6.8, J_2 = 4.2 Hz, 1H), 4.63 (d, J = 4.2 Hz, 1H), 4.19 (dd, J_1 = 9.2, J_2 = 8.4 Hz, 1H), 4.09 (dd, J_1 = 9.3, J_2 = 4.6 Hz, 1H), 3.46 (tdt, J_1 = 9.3, J_2 = 4.7, J_3 = 2.4 Hz, 1H); ^{13}C-NMR (75 MHz, acetone-d6): 171.1, 157.8, 137.3, 134.0, 128.8, 123.6, 115.8, 75.5, 68.4, 46.4; IR (film) ῦ = 3352 (br), 1735, 1699, 1613, 1596, 1514, 1365, 1267, 1222, 1090, 1012, 819; HRMS(ESI): *m/z*: calc. for $C_{12}H_{13}O_4^+$: 221.0808 [M+H]$^+$, found: 221.0808.

rac-4-[(4-Iodophenyl)(hydroxy)methyl]-3-methylenedihydrofuran-2(3H)-one (**SI-26**).

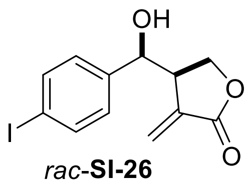

rac-**SI-26**

Batch: zinc dust (< 10 µm, 261.5 mg, 4.0 mmol), ammonium chloride (427.9 mg, 8.0 mmol), 4-iodobenzaldehyde (464.0 mg, 2.0 mmol), allylic bromide **7** (424.8 mg, 2.4 mmol) in toluene/dimethoxyethane 1/1 (5 mL). The obtained crude product was purified via flash chromatography (SiO_2, cyclohexane/EtOAc 4/1 to 2/1) to give the title compound as a colorless oil (581 mg, 1.76 mmol, 88%).

^{1}H-NMR (300 MHz, $CDCl_3$): 7.80 – 7.61 (m, 2H), 7.29 (d, J = 7.7 Hz, 1H), 7.12 (t, J = 7.8 Hz, 1H), 6.35 (d, J = 2.2 Hz, 1H), 5.72 (d, J = 1.7 Hz, 1H), 4.66 (d, J = 6.9 Hz, 1H), 4.20 (dd, J_1 = 9.6, J_2 = 8.2 Hz, 1H), 4.07 (dd, J_1 = 9.7, J_2 = 4.1 Hz, 1H), 3.36 (tdt, J_1 = 8.4, J_2 = 4.2, J_3 = 2.2 Hz, 1H), 2.55 (br s, 1H); ^{13}C-NMR (CDCl3, 75 MHz): 170.7, 143.1, 137.8, 135.7, 134.6, 130.6, 126.0, 125.9, 94.9, 74.9, 67.7, 45.5; IR (film) ῦ = 3488 (br), 1731, 1657, 1589, 1562, 1475, 1411, 1381, 1342, 1316, 1272, 1186, 1128, 1107, 1081, 1062, 1053, 1019, 991, 979, 965, 949, 900, 873, 842, 818, 785, 773, 721, 700, 684, 658, 637, 624, 573; HRMS(ESI): *m/z*: calc. for $C_{12}H_{11}IO_3Na^+$: 243.0628 [M+Na]$^+$, found: 243.0630.

General procedure for the 1,4-addition of boronic acid ester to a unsaturated lactone.

A 5 mL Biotage vial, equipped with a magnetic stir bar, was charged with boronic acid pinacol ester (1.5 eq), $[Rh(cod)Cl]_2$ (3 mol%, 0.03 eq) and the corresponding allyation product (1.0 eq). The mixture was dissolved in 1,4-dioxane and $H_2O_{des.}$ and Et_3N was added. The vial was capped, crimped with an alumina ring and stirred (310 rpm) in a preheated oil bath (70 °C bath temperature) for 3 h. After complete conversion the reaction mixture was cooled to rt., diluted with saturated $NaHCO_3$-solution and extracted with EtOAc (3 x 15 mL). The combined organic phase was washed with brine, dried over Na_2SO4, the solvent was removed under reduced pressure and the crude product was purified by flash column chromatography (SiO_2,

cyclohexane/EtOAc or toluene/EtOAc, for details see below) to give the corresponding product (all racemic substrates showed d.r. = >95:<5 unless otherwise noted below).

rac-4-{Benzo[d][1,3]dioxol-5-yl(hydroxy)methyl}-3-(3,4,5-trimethoxybenzyl)dihydrofuran-2(3H)-one (2d).

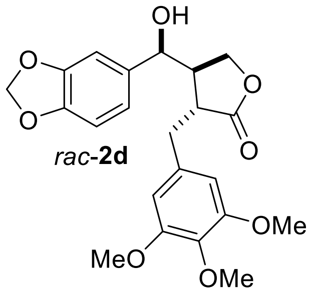

Batch: $[Rh(cod)Cl]_2$ (3 mg, 0.007 mmol), the (3,4,5-trimethoxyphenyl)boronic acid pinacol ester (**9**, 97 mg, 0.33 mmol), Et_3N (30 µL, 22 mg, 0.22 mmol) and lactone **8** (54 mg, 0.22 mmol) in dioxane/water 4/1 (750 µL). The crude product was purified via flash chromatography (SiO_2, toluene/EtOAc 3/1) to give the desired compound **2d** as a colorless oil (80 mg, 0.19 mmol, 87%).

^{1}H-NMR (300 MHz, $CDCl_3$): 6.74-6.66 (m, 3H), 6.35 (s, 2H), 5.95 (dd, J_1 = 3.8, J_2 = 1.2, 2H), 4.62 (d, J = 6.5, 1H), 3.94 (d, J = 7.6, 2H), 3.80 (s, 9H), 3.04-2.85 (m, 3H), 2.60 (p, J = 7.2, 1H), 2.36 (br s, 1H); ^{13}C-NMR (75 MHz, $CDCl_3$): 179.2, 153.1, 148.2, 147.6, 136.7, 135.5, 133.4, 119.3, 108.3, 106.7, 106.2, 101.4, 75.3, 68.5, 60.9, 56.1, 45.1, 43.7, 35.6; IR (film) ṽ = 3487 (br), 2939, 2839, 1757, 1591, 1505, 1488, 1459, 1443, 1422, 1384, 1348, 1322, 1237, 1185, 1123, 1033, 1005, 909, 813, 782, 726; HRMS(ESI): *m/z*: calc. for $C_{22}H_{24}O_8NH_4^+$: 434.1809 $[M+NH_4]^+$, found: 434.1809.

rac-4-[(3,4-dimethoxyphenyl)(hydroxy)methyl]-3-(3,4,5-trimethoxybenzyl)dihydrofuran-2(3H)-one (2c).

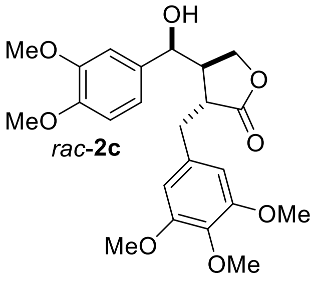

$[Rh(cod)Cl]_2$ (3 mg, 0.007 mmol), the (3,4,5-trimethoxyphenyl)boronic acid pinacol ester (**9**, 97 mg, 0.33 mmol), Et_3N (30 µL, 22 mg, 0.22 mmol) and lactone **SI-12** (58 mg, 0.22 mmol) in dioxane/water 4/1 (750 µL). The crude product was purified via flash chromatography (SiO_2, cyclohexane/EtOAc 1/1) to give the desired compound **2c** as a yellow oil (69 mg, 0.16 mmol, 71%).

^{1}H-NMR (300 MHz, $CDCl_3$): 6.81 – 6.65 (m, 3H), 6.34 (s, 2H), 4.62 (d, J = 6.7 Hz, 1H), 3.91 (dd, J_1 = 7.7, J_2 = 2.7 Hz, 2H), 3.84 (s, 3H), 3.81 (s, 3H), 3.78 (s, 3H), 3.77 (s, 6H), 3.09 – 2.79 (m, 3H), 2.60 (p, J = 7.4 Hz, 1H), 2.31 (br s, 1H); ^{13}C-NMR (75 MHz, $CDCl_3$): 179.3, 153.1, 149.3, 149.0, 136.8, 134.3, 133.4, 118.3, 111.2, 109.1, 106.9, 75.2, 68.5, 60.9, 56.1, 56.00, 55.95, 45.2, 43.7, 35.5; IR (film) ṽ = 3490 (br), 1731, 1591, 1464, 1420, 1247, 1185, 1125, 1013, 818, 751, 721; HRMS(ESI): *m/z*: calc. for $C_{23}H_{28}O_8NH_4^+$: 450.2122 $[M+NH_4]^+$, found: 450.2128.

rac-4-[hydroxy(phenyl)methyl)-3-(3,4,5-trimethoxybenzyl)dihydrofuran-2(3H)-one (**2e**).

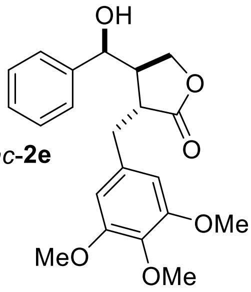

rac-**2e**

$[Rh(cod)Cl]_2$ (3 mg, 0.007 mmol), the (3,4,5-trimethoxyphenyl)boronic acid pinacol ester (**9**, 97 mg, 0.33 mmol), Et_3N (30 µL, 22 mg, 0.22 mmol) and lactone **SI-13** (23.4 mg, 0.22 mmol) in dioxane/water 4/1 (750 µL). The crude product was purified via flash chromatography (SiO_2, toluene/EtOAc 3/1) to give the desired compound **2e** as a pale yellow solid (72.9 mg, 0.20 mmol, 89%).

Mp: 95-97 °C (from $CDCl_3$); ^{1}H-NMR (300 MHz, $CDCl_3$): 7.38 – 7.22 (m, 5H), 6.32 (s, 2H), 4.72 (d, J = 6.5 Hz, 1H), 3.95 (p, J = 9.3 Hz, 2H), 3.81 (s, 3H), 3.79 (s, 6H), 3.10 – 2.97 (m, 2H), 2.82 (dd, J_1 = 13.1, J_2 = 4.9 Hz, 1H), 2.67 (dt, J_1 = 14.8, J_2 = 7.5 Hz, 1H), 2.25 (s, 1H); ^{13}C-NMR (75 MHz, $CDCl_3$): 179.2, 153.2, 141.6, 136.7, 133.3, 129.0, 128.6, 126.0, 106.8, 75.4, 68.4, 61.0, 56.2, 44.9, 43.6, 35.5; IR (film) ῦ = 3506 (br), 2928, 1770, 1589, 1505, 1451, 1422, 1244, 1232, 1183, 1161, 1144, 1118, 1072, 1012, 988, 849, 781, 732, 708; HRMS(ESI): *m/z*: calc. for $C_{21}H_{24}O_6NH_4^+$: 390.1911 $[M+NH_4]^+$, found: 390.1905.

rac-4-[hydroxy(naphthalen-2-yl)methyl]-3-(3,4,5-trimethoxybenzyl)dihydrofuran-2(3H)-one (**2f**).

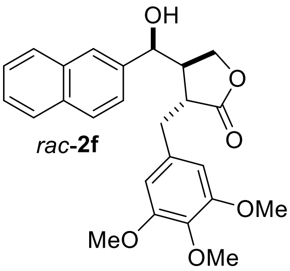

rac-**2f**

$[Rh(cod)Cl]_2$ (3 mg, 0.007 mmol), the (3,4,5-trimethoxyphenyl)boronic acid pinacol ester (**9**, 97 mg, 0.33 mmol), Et_3N (30 µL, 22 mg, 0.22 mmol) and lactone **SI-14** (55.9 mg, 0.22 mmol) in dioxane/water 4/1 (750 µL). The crude product was purified via flash chromatography (SiO_2, toluene/EtOAc 3/1 to 1/1) to give the desired compound **2f** as a colorless oil (63.4 mg, 0.15 mmol, 68%).

^{1}H-NMR (300 MHz, $CDCl_3$): 7.84 – 7.78 (m, 3H), 7.72 (s, 1H), 7.54 – 7.48 (m, 2H), 7.29 – 7.26 (m, 1H), 6.25 (s, 2H), 4.89 (d, J = 6.2, 1H), 4.03 (dt, J_1 = 17.4, J_2 = 9.2, 2H), 3.80 (s, 3H), 3.68 (s, 6H), 3.07 – 2.99 (m, 2H), 2.88 – 2.72 (m, 2H); ^{13}C-NMR (75 MHz, $CDCl_3$): 179.2, 153.1, 138.9, 136.7, 133.3, 133.2, 128.9, 128.00, 127.95, 126.8, 126.6, 125.1, 123.5, 106.6, 75.4, 68.6, 61.0, 56.1, 44.8, 43.4, 35.6; IR (film) ῦ = 3473 (br), 2935, 2837, 1759, 1591, 1507, 1459, 1422, 1237, 1122, 1021, 1004, 818, 745, 730; HRMS(ESI): *m/z*: calc. for $C_{25}H_{26}O_6NH_4^+$: 440.2068 $[M+NH_4]^+$, found: 440.2068.

rac-4-[(3-chlorophenyl)(hydroxy)methyl]-3-(3,4,5-trimethoxybenzyl)dihydrofuran-2(3H)-one (2g).

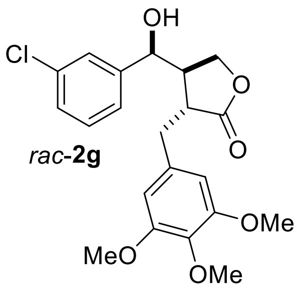

rac-**2g**

$[Rh(cod)Cl]_2$ (3 mg, 0.007 mmol), the (3,4,5-trimethoxyphenyl)boronic acid pinacol ester (**9**, 97 mg, 0.33 mmol), Et_3N (30 µL, 22 mg, 0.22 mmol) and lactone **SI-15** (52.5 mg, 0.22 mmol) in dioxane/water 4/1 (750 µL). The crude product was purified via flash chromatography (SiO_2, toluene/EtOAc 3/1) to give the desired compound **2g** as a colorless oil (44.8 mg, 0.11 mmol, 50%).

^{1}H-NMR (300 MHz, $CDCl_3$): 7.36 – 7.26 (m, 1H), 7.24 – 7.16 (m, 1H), 7.09 – 7.05 (m, 1H), 6.30 (s, 2H), 4.73 (d, *J* = 5.8 Hz, 1H), 4.02 (d, *J* = 7.6 Hz, 2H), 3.81 (s, 3H), 3.80 (s, 6H), 3.09 – 2.92 (m, 2H), 2.89 – 2.78 (m, 1H), 2.69 – 2.60 (m, 1H), 2.33 (s, 1H); ^{13}C-NMR (75 MHz, $CDCl_3$): 179.0, 153.3, 143.7, 136.8, 135.0, 133.2, 130.2, 128.6, 126.2, 124.1, 106.6, 74.6, 68.5, 61.0, 56.2, 45.0, 43.2, 35.8; IR (film) ῦ = 3477 (br), 2938, 2839, 1756, 1591, 1507, 1460, 1422, 1324, 1238, 1185, 1124, 1005, 909, 781, 753, 729, 667; HRMS(ESI): *m/z*: calc. for $C_{21}H_{23}ClO_6Na^+$: 429.1075 $[M+Na]^+$, found: 429.1068.

rac-4-[(4-chlorophenyl)(hydroxy)methyl]-3-(3,4,5-trimethoxybenzyl)dihydrofuran-2(3*H*)-one (2h).

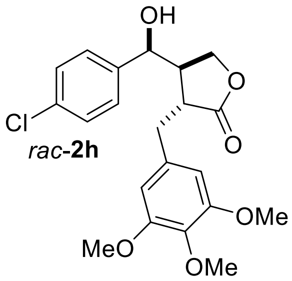

rac-**2h**

$[Rh(cod)Cl]_2$ (3 mg, 0.007 mmol), the (3,4,5-trimethoxyphenyl)boronic acid pinacol ester (**9**, 97 mg, 0.33 mmol), Et_3N (30 µL, 22 mg, 0.22 mmol) and lactone **SI-16** (52.5 mg, 0.22 mmol) in dioxane/water 4/1 (750 µL). The crude product was purified via flash chromatography (SiO_2, toluene/EtOAc 2/1 to 1/1) to give the desired compound **2h** as a pale yellow solid (59.1 mg, 0.15 mmol, 66%).

Mp: 68-70 °C (from $CDCl_3$); ^{1}H-NMR (300 MHz, $CDCl_3$): 7.31 (d, *J* = 8.4 Hz, 2H), 7.17 (d, *J* = 8.4 Hz, 2H), 6.29 (s, 2H), 4.72 (d, *J* = 6.2 Hz, 1H), 3.97 (d, *J* = 7.5 Hz, 2H), 3.82 (s, 3H), 3.79 (s, 6H), 3.05 – 2.92 (m, 2H), 2.84 (dd, J_1 = 12.9, J_2 = 4.8 Hz, 1H), 2.66 – 2.57 (m, 1H); ^{13}C-NMR (75 MHz, $CDCl_3$): 179.0, 153.2, 140.1, 136.8, 134.3, 133.2, 129.1, 127.4, 106.6, 74.7, 68.4, 61.0, 56.2, 45.0, 43.4, 35.6; IR (film) ῦ = 3490 (br), 1731, 1591, 1464, 1420, 13181, 1345, 1317, 1247, 1185, 1125, 1013, 992, 979, 949, 818, 751, 721; HRMS(ESI): *m/z*: calc. for $C_{21}H_{23}ClO_6NH_4^+$: 424.1521 $[M+NH_4]^+$, found: 424.1526.

rac-4-[(3-Iodophenyl)(hydroxy)methyl]-3-(3,4,5-trimethoxybenz-yl)dihydrofuran-2(3*H*)-one (**2i**):

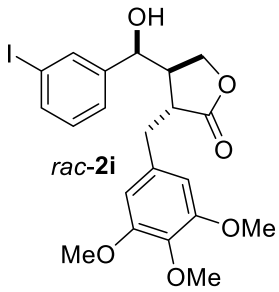

rac-**2i**

[Rh(cod)Cl]$_2$ (17 mg, 0.04 mmol), the (3,4,5-trimethoxyphenyl)boronic acid pinacol ester (**9**, 363 mg, 1.71 mmol), Et$_3$N (159 µL, 115 mg, 1.14 mmol) and lactone **SI-17** (377 mg, 1.14 mmol) in dioxane/water 4/1 (3.9 mL). The crude product was purified via flash chromatography (SiO$_2$, toluene/EtOAc 4/1) to give the desired compound **2i** as a pale yellow solid (449 mg, 0.91 mmol, 80%).

Mp = 47-50 °C (from CDCl$_3$); ^{1}H-NMR (300 MHz, CDCl$_3$): 7.61 (d, *J* = 8.2 Hz, 2H), 7.17 – 7.09 (m, 1H), 7.03 (t, *J* = 7.7 Hz, 1H), 6.30 (d, *J* = 3.5 Hz, 2H), 4.68 (t, *J* = 7.4 Hz, 1H), 3.97 (dd, J_1 = 12.3, J_2 = 4.9 Hz, 2H), 3.79 (s, 9H), 3.05 – 2.78 (m, 3H), 2.69 – 2.57 (m, 1H), 2.35 (m, 1H); ^{13}C-NMR (75 MHz, CDCl$_3$): 179.1, 153.2, 144.1, 137.5, 136.7, 135.1, 133.2, 130.5, 129.1, 128.3, 125.2, 106.6, 94.9, 74.4, 68.5, 61.0, 56.3, 45.0, 43.2, 35.7. IR (film) ũ = 3479 (br), 2934, 1750, 1589, 1506, 1457, 1420, 1387, 1237, 1121, 1004, 777, 687; HRMS(ESI): m/z: calc. for $C_{21}H_{23}IO_6$ NH_4^+: 516.0879 $[M+NH_4]^+$, found: 516.0877.

rac-Methyl 4-{hydroxy[5-oxo-4-(3,4,5-trimethoxybenzyl)tetrahydrofuran-3-yl]methyl}benzoate (**2j**).

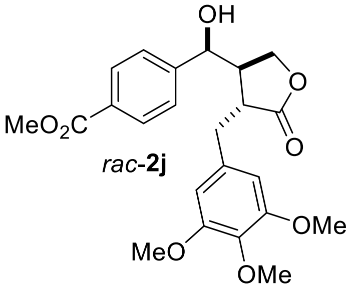

rac-**2j**

[Rh(cod)Cl]$_2$ (17.4 mg, 0.04 mmol), the (3,4,5-trimethoxyphenyl)boronic acid (375.3 mg, 1.76 mmol), Et$_3$N (165 µL, 119.4 mg, 1.18 mmol) and lactone **SI-18** (308.5 mg, 1.18 mmol) in dioxane/water 4/1 (4.2 mL). The crude product was purified via flash chromatography (SiO$_2$, cyclohexane/EtOAc 1/1) to give the desired compound **2j** as a pale yellow solid (337.1 mg, 0.78 mmol, 66%, d.r. = 93:7).

Mp = 43-47 °C (from CHCl$_3$); ^{1}H-NMR (300 MHz, CDCl$_3$): 7.98 (d, J = 8.3 Hz, 2H), 7.31 (d, J = 8.3 Hz, 2H), 6.23 (s, 2H), 4.81 (d, J = 5.7 Hz, 1H), 4.02 (p, J = 9.1 Hz, 2H),3.91 (s, 3H), 3.79 (s, 3H), 3.76 (s, 6H), 2.96 (d, J = 6.8 Hz, 2H), 2.78 – 2.71 (m, 1H), 2.64 (p, J = 6.7 Hz, 1H), 2.43 (br s, 1H); 13C-NMR (75 MHz, CDCl3): 179.1, 166.6, 153.2, 146.7, 136.7, 133.1, 130.2, 130.1, 126.0, 106.5, 74.5, 68.5, 60.9, 56.2, 52.4, 44.9, 42.9, 35.7; IR (film) ũ = 3474 (br), 2942, 2839, 1759, 1715, 1610, 1590, 1507, 1421, 1121, 975, 760, 691; HRMS(ESI): m/z: calc. for $C_{23}H_{27}O_8^+$: 431.1700 $[M+H]^+$, found: 431.1697.

rac-4-[hydroxy(4-methoxyphenyl)methyl]-3-(3,4,5-trimethoxybenzyl)dihydrofuran-2(3*H*)-one (**2k**).

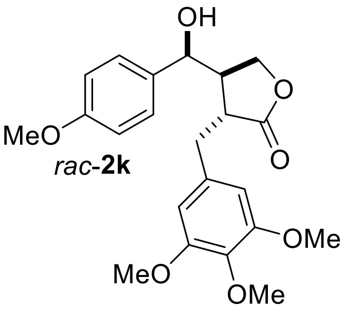

rac-**2k**

[Rh(cod)Cl]$_2$ (12 mg, 0.025 mmol), the (3,4,5-trimethoxyphenyl)boronic acid (262 mg, 1.23 mmol), Et$_3$N (113 µL, 83 mg, 0.82 mmol) and lactone **SI-19** (193 mg, 0.82 mmol) in dioxane/water 4/1 (2.75 mL). The crude product was purified via flash chromatography (SiO$_2$, cyclohexane/EtOAc 3/2) to give the desired compound **2k** as a colorless oil (281 mg, 0.70 mmol, 85%).

^{1}H-NMR (300 MHz, CDCl$_3$): 7.18 – 7.11 (m, 2H), 6.91 – 6.80 (m, 2H), 6.37 (s, 2H), 4.65 (d, *J* = 6.9 Hz, 1H), 3.90 (dd, J_1 = 7.8, J_2 = 1.3 Hz, 2H), 3.81 (s, 3H), 3.80 (s, 6H), 3.79 (s, 3H), 3.07 (dd, J_1 = 12.9, J_2 = 4.8 Hz, 1H), 2.96 (dt, J_1 = 7.2, J_2 = 4.9 Hz, 1H), 2.89 (dd, J_1 = 12.9, J_2 = 5.3 Hz, 1H), 2.64 (p, *J* = 7.5 Hz, 1H); ^{13}C-NMR (75 MHz, CDCl$_3$): 179.2, 159.7, 153.2, 136.8, 133.7, 133.4, 127.3, 114.3, 106.9, 75.3, 68.4, 61.0, 56.2, 55.4, 45.0, 43.9, 35.5; IR (film) ῦ = 3487 (br), 2938, 2838, 1759, 1611, 1590, 1509, 1459, 1422, 1386, 1242, 1176, 1124, 1029, 1005, 910, 832, 727; HRMS(ESI): *m/z*: calc. for $C_{22}H_{26}O_7NH_4^+$: 420.2017 $[M+NH_4]^+$, found: 420.2016.

rac-4-[hydroxy(3-methoxyphenyl)methyl]-3-(3,4,5-trimethoxybenzyl)dihydrofuran-2(3*H*)-one (**2l**).

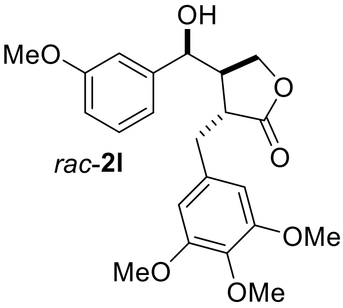

rac-**2l**

[Rh(cod)Cl]2 (15.8 mg, 0.032 mmol), the (3,4,5-trimethoxyphenyl)boronic acid (340.1 mg, 1.60 mmol), Et3N (149.2 µL, 108.3 mg, 1.07 mmol) and lactone **SI-20** (250.5 mg, 1.07 mmol) in dioxane/water 4/1 (3.88 mL). The crude product was purified via flash chromatography (SiO$_2$, cyclohexane/EtOAc 1/1) to give the desired compound **2l** as a pale yellow solid (314.6 mg, 0.78 mmol, 73%, d.r. = >90:<10).

Mp = 117-122 °C (from CHCl$_3$); ^{1}H-NMR (300 MHz, CDCl3): 7.25 (t, J = 7.8 Hz, 1H), 6.85 – 6.77 (m, 3H), 6.32 (s, 2H), 4.69 (d, J = 6.2 Hz, 1H), 4.05 – 3.89 (m, 2H), 3.80 (s, 3H), 3.79 (s, 6H), 3.78 (s, 3H), 3.09 – 2.95 (m, 2H), 2.81 (dd, J = 12.5, 4.2 Hz, 1H), 2.65 (p, J = 7.5 Hz, 1H), 2.27 (s, 1H); 13C-NMR (75 MHz, CDCl3): 179.2, 160.1, 153.1, 143.4, 136.7, 133.4, 130.0, 118.1, 113.4, 112.0, 106.8, 75.1, 68.5, 60.9, 56.2, 55.3, 44.9, 43.4, 35.6; IR (film) ῦ =3511, 3003, 2946, 2840, 1756, 1591, 1423, 1387, 1257, 1244, 1185, 1160, 1011, 996, 977, 773, 691; HRMS(ESI): *m/z*: calc. for $C_{22}H_{27}O_7^+$: 403.1751 $[M+H]^+$, found: 403.1751.

rac-4-[hydroxy(4-fluorophenyl)methyl]-3-(3,4,5-trimethoxybenzyl)dihydrofuran-2(3*H*)-one (**2m**).

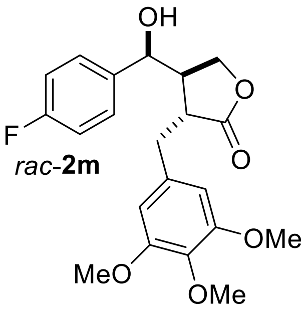

rac-**2m**

$[Rh(cod)Cl]_2$ (17 mg, 0.033 mmol), the (3,4,5-trimethoxyphenyl)boronic acid (355 mg, 1.67 mmol), Et_3N (155 µL, 112 mg, 1.11 mmol) and lactone **SI-19** (248 mg, 1.11 mmol) in dioxane/water 4/1 (3.80 mL). The crude product was purified via flash chromatography (SiO_2, toluene/EtOAc 4:1 to 3:1) to give the desired compound **2m** as a white solid (322 mg, 0.91 mmol, 75%, d.r. = 95:5).

Mp = 105-107 °C (from $CDCl_3$); ^{1}H NMR (300 MHz, $CDCl_3$) δ 7.21 (dd, J_1 = 8.6, J_2 = 5.3 Hz, 2H), 7.02 (t, J = 8.6 Hz, 2H), 6.33 (s, 2H), 4.71 (d, J = 4.5 Hz, 1H), 3.94 (d, J = 7.7 Hz, 2H), 3.81 (s, 3H), 3.80 (s, 6H), 3.10 – 2.82 (m, 3H), 2.63 (p, J = 7.3 Hz, 1H), 2.24 (t, J = 8.7 Hz, 1H). ^{13}C NMR (75 MHz, $CDCl_3$) δ 179.0, 162.6 (d, J = 247.6 Hz), 153.2, 137.4 (d, J = 3.2 Hz), 136.8, 133.3 (s), 127.8 (d, J = 8.1 Hz), 115.9 (d, J = 21.5 Hz), 106.7, 74.9, 68.4, 61.0, 56.2, 45.1, 43.7, 35.6. IR (film) ṽ = 3476 (br), 2939, 1756, 1590, 1507, 1458, 1421, 1387, 1323, 1219, 1184, 1122, 1004, 835, 750; HRMS(ESI): *m*/*z*: calc. for $C_{21}H_{24}FO_6^+$: 391.1551 $[M+H]^+$, found: 391.1554.

rac-4-[(4-iodophenyl)(hydroxy)methyl]-3-(3,4,5-trimethoxybenzyl)dihydrofuran-2(3*H*)-one (**SI-1**).

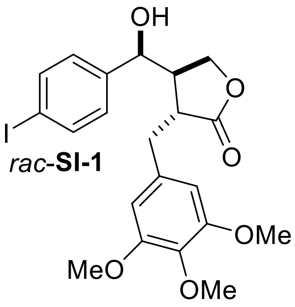

rac-**SI-1**

$[Rh(cod)Cl]_2$ (3 mg, 0.007 mmol), the (3,4,5-trimethoxyphenyl)boronic acid pinacol ester (**9**, 97 mg, 0.33 mmol), Et_3N (30 µL, 22 mg, 0.22 mmol) and lactone **SI-26** (72.6 mg, 0.22 mmol) in dioxane/water 4/1 (750 µL). The crude product was purified via flash chromatography (SiO_2, toluene/EtOAc 4/1 to 3/1) to give the desired compound **SI-1** as a yellow oil (60.3 mg, 0.12 mmol, 55%).

^{1}H-NMR (300 MHz, $CDCl_3$): 7.64 – 7.61 (m, 2H), 7.18 – 7.02 (m, 2H), 6.31 (s, 2H), 4.68 (dd, J_1 = 6.0, J_2 = 2.9 Hz, 1H), 3.99 (d, J = 7.6 Hz, 2H), 3.81 (s, 9H), 3.05 – 2.91 (m, 2H), 2.85 (dd, J_1 = 12.7, J_2 = 4.7 Hz, 1H), 2.69 – 2.59 (m, 1H); ^{13}C-NMR (75 MHz, $CDCl_3$): 179.0, 153.3, 144.0, 137.5, 136.8, 135.1, 133.2, 130.6, 125.2, 106.6, 94.9, 74.6, 68.4, 61.0, 56.3, 45.0, 43.4, 35.7; IR (film) ṽ = 3471 (br), 2935, 2837, 1752, 1590, 1507, 1459, 1421, 1237, 1184, 1152, 1028, 1005, 909, 848, 831, 778, 731, 688; HRMS(ESI): *m*/*z*: calc. for $C_{21}H_{23}IO_6NH_4^+$: 516.0878 $[M+NH_4]^+$, found: 516.0882.

rac-4-[Benzo[d][1,3]dioxol-5-yl(hydroxy)methyl]-3-(3,4-dimethoxybenzyl)dihydrofuran-2(3*H*)-one (**SI-2**).

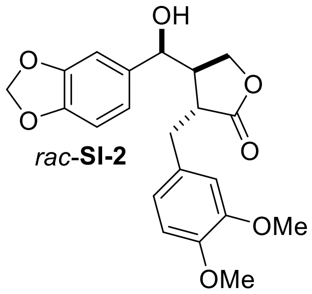

rac-**SI-2**

$[Rh(cod)Cl]_2$ (3 mg, 0.007 mmol), the (3,4-dimethoxyphenyl)boronic acid (60.1 mg, 0.33 mmol), Et_3N (30 µL, 22 mg, 0.22 mmol) and lactone **8** (54.6 mg, 0.22 mmol) in dioxane/water 4/1 (750 µL). The crude product was purified via flash chromatography (SiO_2, toluene/EtOAc 1/1) to give the desired compound **SI-2** as a yellow oil (61.8 mg, 0.16 mmol, 74%).

Mp: 106-108 °C (from $CDCl_3$); ^{1}H-NMR (300 MHz, acetone-d6): 6.82 – 6.71 (m, 4H), 6.65 – 6.61 (m, 2H), 5.98 (dd, J_1 = 1.7, J_2 = 1.1 Hz, 2H), 4.79 – 4.76 (m, 2H), 4.07 (dd, J_1 = 7.2, J_2 = 3.9 Hz, 2H), 3.76 (s, 3H), 3.73 (s, 3H), 2.91 – 2.75 (m, 3H), 2.68 – 2.60 (m, 1H); ^{13}C-NMR (75 MHz, acetone-d6): 179.4, 150.0, 149.0, 148.6, 147.7, 137.8, 131.4, 122.5, 119.9, 114.1, 112.4, 108.4, 107.0, 102.0, 74.6, 69.4, 55.9, 55.8, 46.0, 43.6, 35.7; IR (film) ṽ = 3523 (br), 3020, 2920, 1735, 1514, 1502, 1485, 1439, 1257, 1196, 1123, 1035, 1022, 1004, 937, 924, 859, 806, 760, 744, 667, 624; HRMS(ESI): *m/z*: calc. for $C_{21}H_{22}O_7NH_4^+$: 404.1704 $[M+NH_4]^+$, found: 404.1707.

rac-4-[Benzo[d][1,3]dioxol-5-yl(hydroxy)methyl]-3-(4-methoxybenzyl)dihydrofuran-2(3*H*)-one (**SI-3**).

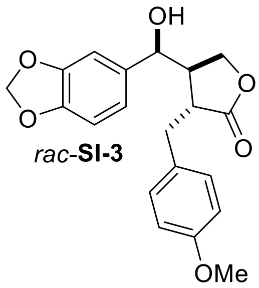

rac-**SI-3**

$[Rh(cod)Cl]_2$ (3 mg, 0.007 mmol), (4-methoxyphenyl)boronic acid (50.1 mg, 0.33 mmol), Et_3N (30 µL, 22 mg, 0.22 mmol) and lactone **8** (54.6 mg, 0.22 mmol) in dioxane/water 4/1 (750 µL). The crude product was purified via flash chromatography (SiO_2, toluene/EtOAc 6/1) to give the desired compound **SI-3** as a pale yellow oil (35.6 mg, 0.10 mmol, 47%).

^{1}H-NMR (300 MHz, acetone-d6): 7.06 – 7.00 (m, 2H), 6.84 – 6.75 (m, 5H), 5.98 (dd, J_1 = 2.0, J_2 = 1.1 Hz, 2H), 4.77 – 4.75 (m, 2H), 4.06 – 3.93 (m, 2H), 3.76 (s, 3H), 2.97 – 2.79 (m, 3H), 2.65 – 2.55 (m, 1H); ^{13}C-NMR (75 MHz, acetone-d6): 179.3, 159.3, 148.7, 147.8, 138.0, 131.5, 130.8, 120.1, 114.4, 108.6, 107.2, 102.0, 74.8, 69.2, 55.4, 46.1, 43.8, 35.1; IR (film) ṽ = 3485 (br), 2919, 2849, 1734, 1612, 1513, 1501, 1482, 1435, 1385, 1307, 1295, 1233, 1197, 1087, 1034, 1006, 984, 936, 807, 784, 749, 685, 669, 514; HRMS(ESI): *m/z*: calc. for $C_{20}H_{20}O_6NH_4^+$: 374.1598 $[M+NH_4]^+$, found: 374.1604.

rac-4-[(2-chlorophenyl)(hydroxy)methyl]-3-(3,4,5-trimethoxybenzyl)dihydrofuran-2(3*H*)-one (SI-5).

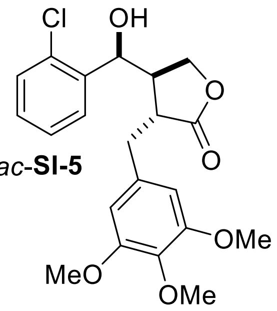

rac-**SI-5**

$[Rh(cod)Cl]_2$ (3 mg, 0.007 mmol), the (3,4,5-trimethoxyphenyl)boronic acid pinacol ester (**9**, 97 mg, 0.33 mmol), Et_3N (30 µL, 22 mg, 0.22 mmol) and lactone **SI-22** (52.5 mg, 0.22 mmol) in dioxane/water 4/1 (750 µL). The crude product was purified via flash chromatography (SiO_2, toluene/EtOAc 5/1) to give the desired compound **SI-5** as a colorless oil (61.8 mg, 0.15 mmol, 69%).

^{1}H-NMR (300 MHz, $CDCl_3$): 7.54 (d, J = 7.1 Hz, 1H), 7.32 – 7.19 (m, 3H), 6.09 (s, 2H), 5.17 (d, J = 3.7 Hz, 1H), 4.31 – 4.20 (m, 2H), 3.79 (s, 3H), 3.76 (s, 6H), 2.99 (dd, J_1 = 11.5, J_2 = 6.3 Hz, 1H), 2.85 (dt, J_1 = 13.9, J_2 = 6.4 Hz, 2H), 2.59 (dd, J_1 = 13.6, J_2 = 4.9 Hz, 1H), 2.26 (br s, 1H); ^{13}C-NMR (75 MHz, $CDCl_3$): 179.4, 153.1, 138.8, 136.6, 133.0, 131.4, 129.8, 129.4, 127.5, 127.3, 106.3, 70.9, 69.3, 60.9, 56.0, 42.1, 41.8, 36.1; IR (film) ῦ = 3346 (br), 2976, 2914, 1738, 1659, 1594, 1573, 1466, 1443, 1412, 1382, 1344, 1313, 1272, 1189, 1136, 1098, 1669, 1050, 1022, 976, 944, 884, 835, 816, 765, 739; HRMS(ESI): *m/z*: calc. for $C_{21}H_{23}ClO_6NH_4^+$: 424.1521 $[M+NH_4]^+$, found: 424.1530.

rac-4-[furan-2-yl(hydroxy)methyl]-3-(3,4,5-trimethoxybenzyl)dihydrofuran-2(3H)-one (SI-6).

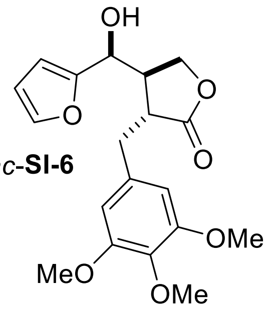

rac-**SI-6**

$[Rh(cod)Cl]_2$ (3 mg, 0.007 mmol), the (3,4,5-trimethoxyphenyl)boronic acid pinacol ester (**9**, 97 mg, 0.33 mmol), Et_3N (30 µL, 22 mg, 0.22 mmol) and lactone **SI-23** (52.5 mg, 0.22 mmol) in dioxane/water 4/1 (750 µL). The crude product was purified via flash chromatography (SiO_2, toluene/EtOAc 5/1) to give the desired compound **SI-6** as a colorless oil (61.8 mg, 0.15 mmol, 69%, d.r. = 7.6:1).

^{1}H-NMR (300 MHz, $CDCl_3$, major isomer): 7.34 (dd, J_1 = 1.9, J_2 = 0.8 Hz, 1H), 6.39 (s, 2H), 6.32 (dd, J_1 = 3.2, J_2 = 1.8 Hz, 1H), 6.20 (d, J = 3.3 Hz, 1H), 4.68 (d, J = 6.4 Hz, 1H), 4.06 (d, J = 7.3 Hz, 2H), 3.81 (s, 10H), 3.12 – 2.75 (m, 4H); ^{13}C-NMR (75 MHz, $CDCl_3$): 179.0, 153.9, 153.2, 142.8, 136.8, 133.3, 110.5, 107.7, 106.6, 68.9, 68.3, 61.0, 56.2, 43.5, 43.2, 35.8; IR (film) ῦ = 3447 (br), 2928, 2841, 1759, 1590, 1507, 1459, 1422, 1385, 1349, 1324, 1238, 1184, 1149, 1123, 1074, 1003, 911, 730; HRMS(ESI): *m/z*: calc. for $C_{19}H_{22}O_7NH_4^+$: 380.1704 $[M+NH_4]^+$, found: 380.1705.

rac-4-[hydroxy(4-hydroxyphenyl)methyl]-3-(3,4,5-trimethoxybenzyl)dihydrofuran-2(3*H*)-one (**SI-10**).

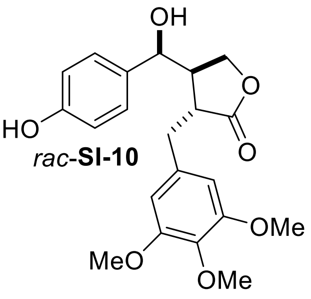

$[Rh(cod)Cl]_2$ (3 mg, 0.007 mmol), the (3,4,5-methoxyphenyl)boronic acid (50.1 mg, 0.33 mmol), Et_3N (30 µL, 22 mg, 0.22 mmol) and lactone **SI-25** (48.4 mg, 0.22 mmol) in dioxane/water 4/1 (750 µL). The crude product was purified via flash chromatography (SiO_2, toluene/EtOAc 2/1) to give the desired compound **SI-10** as a white solid (18.8 mg, 0.05 mmol, 22%).

Mp: 61-64 °C (from acetone-d6); ^{1}H-NMR (300 MHz, acetone-d6): 8.42 (s, 1H), 7.16 (d, *J* = 8.4 Hz, 2H), 6.79 (d, *J* = 8.6 Hz, 2H), 6.42 (s, 2H), 4.78 – 4.69 (m, 2H), 4.00 (dd, J_1 = 7.7, J_2 = 4.0 Hz, 2H), 3.76 (s, 6H), 3.68 (s, 3H), 3.01 – 2.89 (m, 2H), 2.83 – 2.62 (m, 2H); ^{13}C-NMR (75 MHz, acetone-d6): 179.5, 157.7, 154.0, 137.7, 134.7, 134.6, 128.1, 115.9, 108.0, 74.4, 69.1, 60.5, 56.3, 46.1, 43.7, 36.1; IR (film) ṽ = 3425 (br), 2922, 1737, 1593, 1513, 1478, 1457, 1235, 1004, 938, 856, 835, 823, 729; HRMS(ESI): *m/z*: calc. for $C_{21}H_{24}O_7NH_4^+$: 406.1860 $[M+NH_4]^+$, found: 406.1857.

rac-Yatein (**2a**).

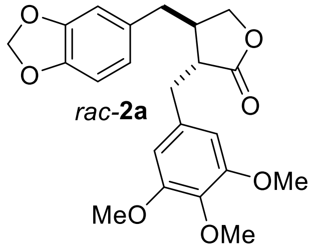

Compound **2d** (154 mg, 0.37 mmol) was dissolved in MeOH (12 mL). Pd/C (10 wt-%, 62 mg, 6.2 mg Pd, 0.059 mmol, 0.16 eq.) and $HClO_4$ (70 wt-%, 120 µL) were added and the reaction mixture was placed under an atmosphere of hydrogen and stirred for 16 h. The mixture was passed through a pad of celite, the pad was washed with CH_2Cl_2 (20 mL) and the combined filtrate was washed with saturated, aqueous $NaHCO_3$ solution (10 mL). The aqueous phase was reextracted with CH_2Cl_2 (2 x 20 mL) and the combined organic phase was dried over Na_2SO_4, filtered and concentrated. The obtained crude product was purified via flash chromatography (silica gel, hexanes/EtOAc 1/1) to give product **2a** as yellow oil (140 mg, 0.35 mmol, 95%).

^{1}H-NMR (300 MHz, $CDCl_3$): 6.72 – 6.66 (m, 1H), 6.50 – 6.43 (m, 2H), 6.35 (s, 2H), 5.93 (dd, J_1 = 2.9, J_2 = 1.4 Hz, 2H), 4.17 (dd, J_1 = 9.2, J_2 = 7.1 Hz, 1H), 3.90-3.85 (m, 1H), 3.83 (s, 6H), 3.82 (s, 3H), 2.90 (dd, J_1 = 5.8, J_2 = 3.2 Hz, 2H), 2.66 – 2.41 (m, 4H); ^{13}C-NMR (75 MHz, $CDCl_3$): 178.7, 153.4, 148.1, 146.5, 137.0, 133.5, 131.7, 121.7, 108.9, 108.4, 106.3, 101.2, 71.3, 61.0, 56.2, 46.6, 41.2, 38.5, 35.4; IR (film) ṽ = 2937, 2840, 1764, 1590, 1504, 1489, 1459, 1444, 1422, 1345, 1320, 1239, 1186, 1124, 1035, 1011, 909, 810, 726; HRMS(ESI): *m/z*: calc. for $C_{22}H_{25}O_7^+$: 401.1595 $[M+H]^+$, found: 401.1594.

rac-4-(benzo[d][1,3]dioxole-5-carbonyl)-3-(3,4,5-trimethoxybenzyl)dihydrofuran-2(3*H*)-one (**SI-7**).

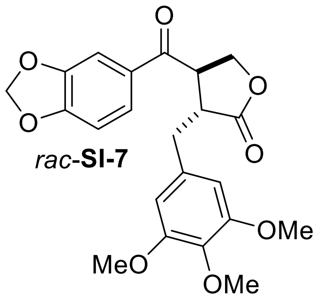

rac-**SI-7**

Compound **2d** (150 mg, 0.36 mmol, 1 eq.) was dissolved in DCM (4.0 mL). Pyridine (60 µl, 60 mg, 0.72 mmol, 2 eq.) was added and the mixture was cooled to 0°C and stirred. After cooling DMP (183.2 mg, 0.43 mmol, 1.2 eq) was added and the reaction mixture was stirred for 48h, after which TLC indicated full conversion. The mixture was quenched with saturated, aqueous NH_4Cl solution and extracted with EtOAc (3x 20 mL) and the combined organic phase was dried over Na_2SO_4, filtered and concentrated. The obtained crude product was purified via flash chromatography (silica gel, CyHex/EtOAc 1/1) to give product **SI-7** as a pale yellow solid (100 mg, 0.24 mmol, 66%).

Mp: 126-129 °C (from CHCl3); 1H-NMR (300 MHz, CDCl3) 7.31 – 7.19 (m, 3H), 6.79 (d, J = 8.1 Hz, 1H), 6.28 (s, 2H), 6.05 (s, 2H), 4.42 (t, J = 7.8 Hz, 1H), 4.05 (dq, J = 17.5, 8.9 Hz, 2H), 3.76 (s, 3H), 3.70 (s, 6H), 3.56 (q, J = 7.1 Hz, 1H), 3.06 (dd, J = 14.1, 5.4 Hz, 1H), 2.95 (dd, J = 14.2, 7.2 Hz, 1H) ; 13C-NMR (75 MHz, CDCl3): 194.5, 177.2, 153.4, 152.9, 148.8, 136.9, 132.9, 130.6, 124.9, 108.1, 108.0, 106.3, 102.4, 68.4, 60.9, 56.1, 47.0, 44.7, 35.0; IR (film) ῦ = 2940,2840, 2252, 1771, 1671, 1591,1506, 1461, 1252, 1126, 1034, 905, 723, 647; HRMS(ESI): *m/z*: calc. for $C_{22}H_{23}O_8^+$: 415.1387 $[M+H]^+$, found: 415.1388.

rac-benzo[d][1,3]dioxol-5-yl[5-oxo-4-(3,4,5-trimethoxybenzyl)tetrahydrofuran-3-yl]methyl acetate (**SI-8**).

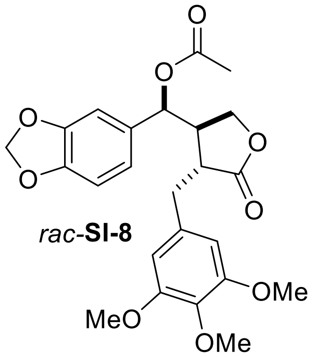

rac-**SI-8**

Compound **2d** (41.6 mg, 0.1 mmol, 1.0 eq.), acetic acid (6.8 µL, 0.12 mmol, 1.2 eq.), *N*,*N'*-dicyclohexylcarbodiimide (DCC, 24.7 mg, 0.12 mmol, 1.2 eq.) and 4-(*N*,*N*-dimethylamino)pyridine (DMAP, 2.5 mg, 0.02 mmol, 0.2 eq.) were dissolved in CH_2Cl_2 (5 mL) and stirred at room temperature for 5 h. The mixture was diluted with CH_2Cl_2 (20 mL) and washed with HCl (0.1 M, 15 mL), saturated, aqueous $NaHCO_3$ (15 mL) and brine (15 mL). The combined organic phase was dried over Na_2SO_4, filtered and concentrated under reduced pressure. The obtained crude product was purified via flash chromatography (SiO_2, cyclohexane/EtOAc 2/1) to give compound SI-8 (30.5 mg, 0.066 mmol, 66%) as colorless oil.

^{1}H-NMR (300 MHz, $CDCl_3$): 6.72 (d, *J* = 7.9 Hz, 1H), 6.65 – 6.54 (m, 2H), 6.36 (s, 2H), 5.96 (d, *J* = 3.3 Hz, 2H), 5.76 (d, *J* = 5.9 Hz, 1H), 4.08 – 3.89 (m, 2H), 3.82 (s, 9H), 3.01 (dd, J_1= 13.2, J_2= 5.0 Hz, 1H), 2.83 (dt, J_1= 10.9, J_2= 5.2 Hz, 3H), 2.11 (s, 3H); ^{13}C-NMR (75 MHz, $CDCl_3$): 178.1, 169.7, 153.4, 148.3, 148.0, 137.1, 132.9, 130.8, 120.1, 108.4, 106.6, 101.6, 76.1, 67.9, 61.0, 56.2, 44.0, 43.6, 35.7, 21.2; IR (film) ῦ = 2935, 1766, 1743, 1590, 1504, 1490, 1446, 1422, 1224, 1123, 1030,

978, 924, 751, 730; HRMS(ESI): *m/z*: calc. for $C_{24}H_{26}O_9NH_4^+$: 476.1916 $[M+NH_4]^+$, found: 476.1915.

rac-4-[(-Benzo[d][1,3]dioxol-5-yl(hydroxy)methyl]-3-(3,4,5-trimethoxybenzyl)dihydrofuran-2(3*H*)-one (2b).

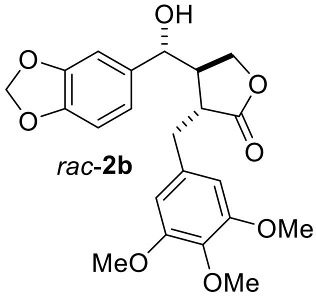

rac-**2b**

Compound **2d** (82.3 mg, 0.2 mmol, 1.0 eq.), *para*-nitrobenzoic acid (100.3 mg, 0.6 mmol, 3.0 eq.) and triphenylphosphine (157.4 mg, 0.6 mmol 3.0 eq) were dissolved in THF (4.5 mL) at 0 °C. Diisopropyl azodicarboxylate (118 µL, 0.6 mmol, 3.0 eq) was added dropwise to this solution over 2 hours and the mixture was allowed to warm to room temperature and stirred for 16 h. The solvent was evaporated under reduced pressure and the crude product was filtered through silica (eluent: toluene/EtOAc 4/1). The obtained intermediate was dissolved in THF/MeOH (5/1, 3 mL) and treated with aqueous K_2CO_3 solution (250 µL, 20 wt-%) for 16 h. The obtained mixture was diluted with Et_2O and quenched with saturated, aqueous NH_4Cl solution. The phases were separated and the aqueous phase was extracted with Et_2O (2 x 10 mL). The combined organic phase was dried over Na_2SO_4, filtered and concentrated under reduced pressure. The obtained crude product was purified via flash chromatography (SiO_2, toluene/EtOAc 5/1) to give **2b** (47 mg, 0.12 mmol, 57 % over two steps) as a pale yellow oil.

^{1}H-NMR (300 MHz, $CDCl_3$): 6.70 (t, *J* = 7.7 Hz, 1H), 6.60 (d, *J* = 7.3 Hz, 2H), 6.24 (s, 2H), 5.97 (dd, J_1 = 9.3, J_2 = 1.3 Hz, 2H), 4.45 – 4.31 (m, 2H), 4.16 (dt, J_1 = 16.3, J_2 = 8.1 Hz, 1H), 3.80 (d, *J* = 3.4 Hz, 9H), 2.77 – 2.59 (m, 3H), 2.53 (p, *J* = 7.0 Hz, 1H), 2.08 (br s, 1H); ^{13}C-NMR (75 MHz, $CDCl_3$): 178.9, 153.3, 148.3, 147.8, 136.9, 135.6, 133.2, 119.7, 108.1, 106.2, 106.1, 101.6, 74.5, 68.5, 61.0, 56.2, 46.2, 43.8, 35.6; IR (film) ũ = 3478 (br), 2934, 1760, 1590, 1504, 1487, 1443, 1421, 1237, 1122, 1022, 1004, 923, 813, 750, 731; HRMS(ESI): *m/z*: calc. for $C_{22}H_{24}O_8NH_4^+$: 434.1816 $[M+NH_4]^+$, found: 434.1809.

Preparation of substrate SI-4.

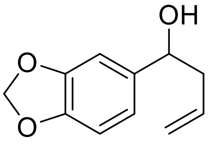

rac-**SI-26**

***rac*-1-(benzo[d][1,3]dioxol-5-yl)but-3-en-1-ol (SI-26).** Piperonal (300 mg, 2.00 mmol) was dissolved in THF (480 µL) and zinc dust (483 mg, 4.00 mmol) was added. Saturated, aqueous NH_4Cl-solution (2.40 mL) was added and the suspension was cooled to 0 °C. Subsequently allyl bromide (490 mg, 350 µL, 4.0 mmol) was added dropwise within 3 min and the suspension was stirred for 7 h at 0 °C and 13 h at room temperature. The grey to colorless slurry was mixed with saturated NH_4Cl-solution (30 mL), the mixture was extracted with EtOAc (3 x 15 mL) and the combined organic phase was washed with brine (20 mL) . The combined organic phase was dried over Na_2SO_4, the solvent

was removed under reduced pressure and the crude product was purified by flash column chromatography (toluene/EtOAc 5/1) to give **SI-26** (271 mg, 1.41 mmol, 71%) as an orange oil. ^{1}H-NMR (300 MHz, $CDCl_3$): δ 6.88 – 6.87 (m, 1H), 6.82 – 6.75 (m, 2H), 5.95 (s, 2H), 5.79 (ddt, J_1 = 17.2, J_2 = 10.2, J_3 = 7.1 Hz, 1H), 5.19 – 5.11 (m, 2H), 4.64 (t, J = 6.5 Hz, 1H), 2.57 – 2.44 (m, 2H), 2.02 (bs, 1H); ^{13}C-NMR (75 MHz, $CDCl_3$): 147.9, 147.0, 138.1, 134.5, 119.3, 118.5, 108.2, 106.5, 101.1, 73.3, 44.0; IR (film) ũ = 3420 (br), 2933, 2901, 1639, 1584, 1503, 1456, 1440, 1412, 1392, 1330, 1292, 1236, 1185, 1124, 1099, 1035, 1005, 919, 865, 810.

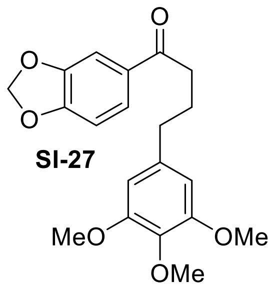

SI-27

***rac*-1-(benzo[d][1,3]dioxol-5-yl)-4-(3,4,5-trimethoxyphenyl)butan-1-one (SI-27).** In a 10 mL microwave tube 3,4,5-trimethoxyphenyliodide (359 mg, 1.24 mmol), allyl alcohol **SI-26** (200 mg, 1.04 mmol), triethylamine (210 mg, 290 µL, 2.081 mmol) and $Pd(OAc)_2$ (7.2 mg, 0.031 mmol, 3 mol-%) were dissolved in dry acetonitrile (2 mL). The vial was crimped, and stirred for 72 h in an oil bath (70 °C oil bath temperature). The reaction was quenched by the addition of saturated, aqueous NH_4Cl solution (10 mL), the layers were separated and the aqueous phase was reextracted with ethyl acetate (3 x 10 mL). The combined organic phases were dried over Na_2SO_4, filtered and the solvent was removed under reduced pressure. Purification of the crude product by flash chromatography (SiO_2, cyclohexane/EtOAc 8/2). The product was isolated as a yellow oil (**SI-27**, 128 mg, 0.36 mmol, 34%, contains 7% of compound **SI-28**, mixture was inseparable).

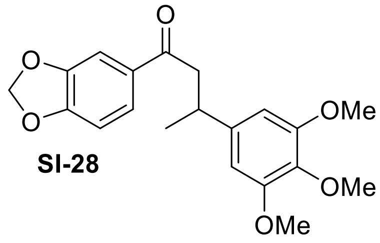

SI-28

^{1}H-NMR (300 MHz, $CDCl_3$, major product **SI-27**): 7.52 (dd, J_1 = 8.1, J_2 = 1.7 Hz, 1H), 7.41 (d, J = 1.7 Hz, 1H), 6.83 (d, J = 8.2 Hz, 1H), 6.41 (s, 2H), 6.03 (s, 2H), 3.83 (s, 6H), 3.82 (s, 3H), 2.90 (t, J = 7.2 Hz, 2H), 2.64 (dd, J_1 = 8.4, J_2 = 6.8 Hz, 2H), 2.10-2.00 (m, 2H); ^{13}C-NMR (75 MHz, $CDCl_3$, major product **SI-27**): 198.3, 153.3, 151.8, 148.3, 137.6, 136.3, 132.0, 124.3, 107.99, 107.97, 105.5, 102.0, 61.0, 56.2, 37.5, 35.7, 26.1; IR (film, mixture) ũ = 2935, 2838, 1672, 1588, 1505, 1488, 1440, 1420, 1351, 1235, 1184, 1121, 1034, 1005, 970, 930, 891, 808, 779, 729; HRMS(ESI): m/z: calc. for $C_{20}H_{23}O_6^{+}$: 359.1489 $[M+H]^{+}$, found: 359.1494.

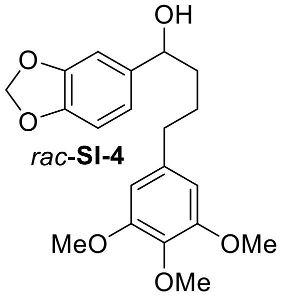

***rac*-1-(benzo[d][1,3]dioxol-5-yl)-4-(3,4,5-trimethoxyphenyl)butan-1-ol (SI-4).** Powdered $NaBH_4$ (21 mg, 0.56 mmol, 2 eq.) was added to a solution of ketone **SI-28** (97 mg, 0.28 mmol) in methanol (2 mL) and the mixture was stirred at room temperature for 30 min. The solvent was then removed under reduced pressure, the residue was quenched with sat. $NaHCO_3$ solution (5 mL), and the product was extracted with ethyl acetate (3 x 2 mL), dried over Na_2SO_4 and the solvent was removed. The crude product was purified via flash chromatography (SiO_2, cyclohexane/EtOAc 8/2) to yield **SI-4** (78 mg, 0.22 mmol, 77 %, contains ca. 10% of compound **SI-29**) as yellow oil.

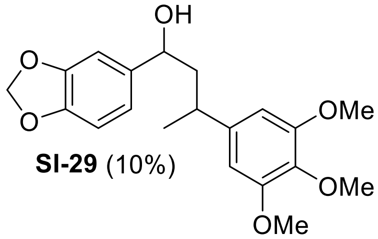

^{1}H-NMR (300 MHz, $CDCl_3$, major product **SI-4**): 6.82 – 6.82 (m, 1H), 6.74 (d, J = 1.0 Hz, 2H), 6.35 (s, 2H), 5.91 (s, 2H), 4.57 (dd, J_1 = 7.2, J_2 = 5.1 Hz, 1H), 3.81 (s, 6H), 3.80 (s, 3H), 2.54 (t, J = 7.2 Hz, 2H), 2.05 (br s, 1H), 1.87 – 1.48 (m, 4H); ^{13}C-NMR (75 MHz, $CDCl_3$, major product **SI-4**): 153.1, 147.8, 146.9, 138.9, 138.1, 136.1, 119.4, 108.1, 106.4, 105.3, 101.0, 74.4, 60.9, 56.1, 38.6, 36.2, 27.7; IR (film, mixture) $\tilde{\nu}$ = 3446 (br), 2936, 2838, 1588, 1505, 1487, 1442, 1419, 1327, 1234, 1183, 1121, 1035, 1005, 931, 863, 811; HRMS(ESI): m/z: calc. for $C_{20}H_{24}O_6Na^+$: 383.1465 $[M+Na]^+$, found: 383.1470.

General procedure for screening of the 2-ODD enzyme on analytical scale

CFE (50 µL) was combined with a stock solution [12.5 µL; 2-oxoglutarate (21.0 mg, 140 µmol) and sodium ascorbat (46.7 mg, 236 µmol) in TRIS buffer (0.5 mL; pH = 7.4, 200 mM, 100 mM NaCl); the pH of the stock solution was adjusted to pH = 7.4 after addition of the reagents again and filled up with buffer to a final volume of 1 mL] in a 1.5 mL Eppendorf vial. The substrate was added as a DMSO stock solution (19 µL) to a final concentration of 20 mM. The vial was closed and placed into a bench top shaker at 30°C and 700 rpm in horizontal position for 16 h. The mixture was extracted with EtOAc (2 x 350 µL), the phases were separated via centrifugation (1 min at 13000 rpm) and decanting, the combined organic phase was dried over Na_2SO_4, centrifuged and the supernatant was subjected to HPLC-UV/MS analysis.

Table SI15. HPLC conditions for the separation of the substrate enantiomers

Entry	Substrate	Chiral HPLC column[a] conditions	Eluent *n*-heptane/2-PrOH
1	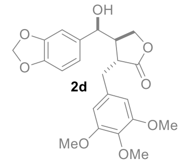 2d	IC 1.0 mL/min, 30°C	70/30
2	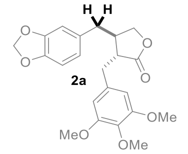 2a	IA 1.0 mL/min, 30°C	80/20
3	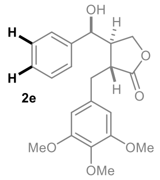 2e	IE 1.0 mL/min, 30°C	80/20
4	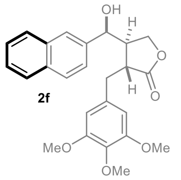 2f	IC 1.0 mL/min, 30°C	70/30
5	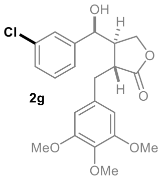 2g	IE 1.0 mL/min, 30°C	80/20
6	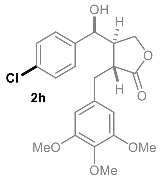 2h	IE 1.0 mL/min, 30°C	80/20
7	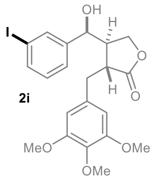 2i	ID 1.0 mL/min, 30°C	80/20
8	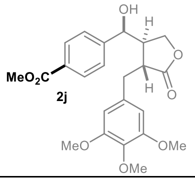 2j	IE 1.0 mL/min, 30°C	80/20

[a] Diacel Chiralpak column (4.6 mm x 250 mm, 5 µm particle size) were used;

Table SI15 – continued.

Entry	Substrate	Chiral HPLC column conditions	Eluent *n*-heptane/2-PrOH
9	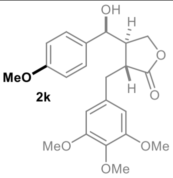 2k	IE 1.0 mL/min, 30°C	80/20
10	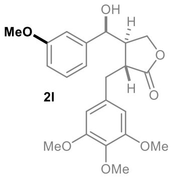 2l	IE 1.0 mL/min, 30°C	80/20
11	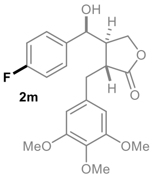 2m	IE 1.0 mL/min, 30°C	80/20
12	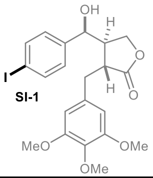 SI-1	IE 1.0 mL/min, 30°C	85/15

[a] Diacel Chiralpak column (4.6 mm x 250 mm, 5 µm particle size) were used;

General procedure for biotransformation with the 2-ODD enzyme on preparative scale.

CFE (7.25 mL) was combined with a stock solution [1.81 mL; 2-oxoglutarate (63.0 mg, 420 µmol) and sodium ascorbat (140.1 mg, 708 µmol) in TRIS buffer (1.5 mL; pH = 7.4, 200 mM, 100 mM NaCl); the pH of the stock solution was adjusted to pH = 7.4 after addition of the reagents again and filled up with buffer to a final volume of 3 mL] in a 50 mL Sarstedt tube. The substrate (240 µmol) was added in DMSO (2.76 mL). The tube was closed and placed into a bench top shaker at 30°C and 700 rpm in horizontal position for 16 h. The mixture was extracted with EtOAc (3 x 10 mL), the phases were separated via centrifugation (10 min at 4000 rpm) and decanting, the combined organic phase was dried over Na_2SO_4, filtered and the filtrate concentrated to give the crude product. The latter was purified via flash chromatography (SiO_2, eluents are given below) to give the target compound.

epi-Podophyllotoxine (**11d**).

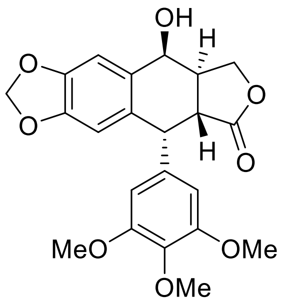

epi-Podophyllotoxin (**11d**)

Substrate **2d** (100 mg, 0.24 mmol); column chromatography (SiO_2, cyclohexane/EtOAc 1/1); yield: **11d** (pale yellow oil, 38 mg, 92 µmol, 38%, d.r. = >95:<5): $[\alpha]_D^{20}$ = -55.9 ($CHCl_3$, c = 1.40), lit.: $[\alpha]_D^{20}$ = -60.0 ($CHCl_3$, c = 1.40);[20] ^{1}H-NMR (300 MHz, $CDCl_3$): 6.88 (s, 1H), 6.54 (s, 1H), 6.27 (s, 2H), 5.98 (dd, J_1 = 7.9, J_2 = 1.2 Hz, 2H), 4.86 (d, J = 3.4 Hz, 1H), 4.60 (d, J = 5.1 Hz, 1H), 4.42 – 4.32 (m, 2H), 3.79 (s, 3H), 3.73 (s, 6H), 3.27 (dd, J_1 = 14.1, J_2 = 5.1 Hz, 1H), 2.89 – 2.77 (m, 1H); ^{13}C-NMR (75 MHz, $CDCl_3$): 175.2, 152.7, 148.7, 147.6, 137.3, 135.2, 132.1, 132.0, 110.7, 109.1, 108.3, 101.7, 67.8, 66.9, 60.9, 56.4, 44.1, 40.6, 38.4; IR (film) ũ = 3446 (br), 2909, 2837, 1768, 1587, 1504, 1482, 1456, 1419, 1332, 1227, 1186, 1156, 1120, 1091, 1033, 995, 929, 858; HRMS(ESI): *m/z*: calc. for $C_{22}H_{22}O_8NH_4^+$: 432.1653 $[M+NH_4]^+$, found: 432.1661.

Table SI16. Comparison of ^{1}H-NMR- and ^{13}C-NMR-data of *epi*-podophyllotoxin (11d)

^{1}H-NMR					^{13}C-NMR		
observed		*literature*[20]			*observed*	*literature*[20]	
ppm	*J*	ppm	*J*	Δppm	ppm	ppm	Δppm
6.88	-	6.87	-	0.01	175.2	175.0	0.02
6.54	-	6.53	-	0.01	152.7	152.6	0.01
6.27	-	6.27	-	0.00	148.7	148.5	0.02
5.98	7.9, 1.2	5.98<br>5.96	-	0.01	147.6	147.5	0.01
4.86	3.4	4.85	2.8	0.01	137.3	137.2	0.01
4.60	5.1	4.59	5.2	0.01	135.2	135.1	0.01
4.42-4.43	-	4.37<br>4.34	10.4, 8.4, 8.0	-	132.1	131.94	0.02
3.79	-	3.79	-	0.00	132.0	131.89	0.01
3.73	-	3.73	-	0.00	110.7	110.5	0.02
3.27	14.1, 5.1	3.26	14.0, 5.2	0.01	109.1	109.0	0.01
2.89-2.77	-	2.82	14.0, 10.8, 8.0, 3.2	-	108.3	108.2	0.01
					101.7	101.5	0.02
					67.8	67.6	0.02
					66.9	66.7	0.02
					60.9	60.7	0.02
					56.4	56.2	0.02
					44.1	43.9	0.02
					40.6	40.5	0.01
					38.4	38.3	0.01

Recovered substrate **2d**: 50.1 mg, 50% yield; $[\alpha]_D^{20}$ = +8.9 ($CHCl_3$, c = 2.50), ee = 72% (for conditions of determination see Table SI15 and HPLC chromatograms); other analytical data are in accordance with that one reported for the racemate above.

Upscale of yatein (**2a**).

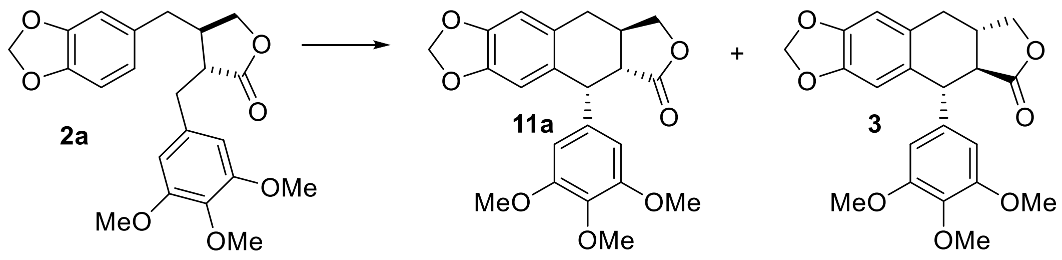

Substrate **2a** (96 mg, 240 µmol); column chromatography (SiO_2, cyclohexane/EtOAc 3/1); Preparative HPLC purification of **11a** from **3**: stationary phase: Phenomenex LUNA AXIA™ pack (5 µm, C18(2), 100 Å, 250 x 21.2 mm, 30 mL/min flow, eluent: H_2O/MeCN; gradient: 10% MeCN for 2 min, 10 to 100% MeCN over 23 min, 100% MeCN for 5 min;

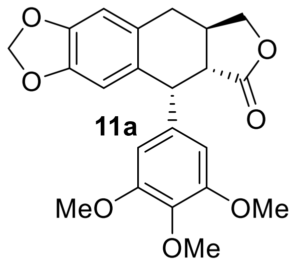

Deoxypodophyllotoxin (11a): yield: yellow resin, 18.2 mg, 46 µmol, 19%, d.r. = >95:<5; $[\alpha]_D^{20}$ = -88.8 ($CHCl_3$, c = 1.34); lit: $[\alpha]_D^{20}$ = -94.0 ($CHCl_3$, c = 1.34);[20] ^{1}H-NMR (300 MHz, $CDCl_3$): 6.66 (s, 1H), 6.52 (s, 1H), 6.34 (s, 2H), 5.94 (dd, J_1 = 6.5, J_2 = 1.3, 2H), 4.59 (d, J = 2.4 Hz, 1H), 4.51 – 4.39 (m, 1H), 3.91 (ddd, J_1 = 8.4, J_2 = 6.5, J_3 = 3.1 Hz, 1H), 3.80 (s, 3H), 3.75 (s, 6H), 3.10 – 3.03 (m, 1H), 2.81 – 2.68 (m, 3H); ^{13}C-NMR (75 MHz, $CDCl_3$): 175.0, 152.6, 147.2, 146.9, 137.2, 136.4, 130.8, 128.4, 110.6, 108.6, 108.5, 101.3, 72.2, 60.9, 56.4, 47.6, 43.8, 33.3, 32.9; IR (film) ῦ = 2899, 2839, 1766, 1587, 1504, 1482, 1455, 1418, 1377, 1359, 1332, 1288, 1222, 1176, 1156, 1121, 1091, 1034, 994, 958, 942, 929, 870, 857, 832, 798, 747; HRMS(ESI): *m/z*: calc. for $C_{22}H_{22}O_7NH_4^+$: 416.1704 $[M+NH_4]^+$, found: 416.1707.

Table SI17. Comparison of ^{1}H-NMR- and ^{13}C-NMR-data of deoxypodophyllotoxin (11a)

^{1}H-NMR					^{13}C-NMR		
observed		*literature*[20]			*observed*	*literature*[20]	
ppm	*J*	ppm	*J*	Δppm	ppm	ppm	Δppm
6.66	-	6.67	-	-0.01	175.0	174.9	0.01
6.52	-	6.52	-	0.00	152.6	152.5	0.01
6.34	-	6.35	-	-0.01	147.2	147.0	0.02
5.94	6.4, 1.3	5.95<br>5.93	-	-	146.9	146.7	0.02
4.59	2.4	4.60	-	-0.01	137.2	137.1	0.01
4.47-4.43	-	4.48-4.43	-	-0.01	136.4	136.3	0.01
3.91	8.4, 6.5, 3.1	3.95-3.89	-	-	130.8	130.6	0.02
3.80	-	3.80	-	0.00	128.4	128.3	0.01
3.75	-	3.75	-	0.00	110.6	110.5	0.01
3.10-3.03	-	3.09-3.04	-	0.01	108.6	108.5	0.01
2.83-2.66	-	2.81-2.68	-	0.02	108.5	108.3	0.02
					101.3	101.2	0.01
					72.2	72.0	0.02
					60.9	60.7	0.02
					56.4	56.2	0.02
					47.6	47.5	0.01
					43.8	43.7	0.01
					33.3	33.1	0.02
					32.9	32.7	0.02

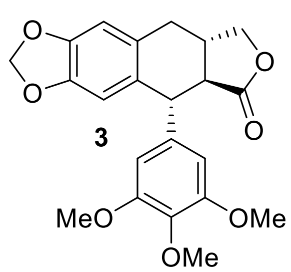

3

Isodeoxypodophyllotoxin (3): yield: yellow resin, 19 mg, 48 µmol, 20%, d.r. = >95:<5; $[\alpha]_D^{20}$ = $[\alpha]_D^{20}$ = +51.5 ($CHCl_3$, c = 0.52); lit: $[\alpha]_D^{20}$ = +50.0 ($CHCl_3$, c = 0.52);[20] ^{1}H-NMR (300 MHz, $CDCl_3$): 6.59 (s, 1H), 6.41 (s, 2H), 6.34 (d, *J* = 0.8 Hz, 1H), 5.89 (dd, J_1 = 4.5, J_2 = 1.4, 2H), 4.51 (dd, J_1 = 8.6, J_2 = 6.2 Hz, 1H), 4.05 (d, *J* = 10.4 Hz, 1H), 3.98 (dd, J_1 = 10.4, J_2 = 8.7 Hz, 1H), 3.84 (s, 3H), 3.81 (s, 6H), 3.01 – 2.86 (m, 2H), 2.68 – 2.48 (m, 2H); ^{13}C-NMR (75 MHz, $CDCl_3$): 175.5, 153.3, 146.8, 146.6, 138.8, 137.1, 132.4, 127.9, 110.1, 108.6, 106.7, 101.3, 71.1, 61.0, 56.3, 48.8, 46.9, 40.3, 33.1; IR (film) ṽ = 2898, 2839, 1786, 1590, 1505, 1482, 1463, 1425, 1389, 1368, 1347, 1329, 1289, 1266, 1225, 1199, 1171, 1149, 1123, 1094, 1035, 997, 949, 930, 918, 884, 868, 785, 739, 726; HRMS(ESI): *m/z*: calc. for $C_{22}H_{23}O_7^+$: 399.1438 $[M+H]^+$, found: 399.1445.

Table SI18. Comparison of ^{1}H-NMR- and ^{13}C-NMR-data of isodeoxypodophyllotoxin (3)

^{1}H-NMR					^{13}C-NMR		
observed		*literature*[20]			*observed*	*literature*[20]	
ppm	*J*	ppm	*J*	Δppm	ppm	ppm	Δppm
6.59	-	6.60	-	-0.01	175.5	175.4	0.01
6.41	-	6.40	-	0.01	153.3	153.1	0.02
6.34	0.8	6.34	-	0.00	146.8	146.6	0.02
5.89	4.5, 1.4	5.90<br>5.89	-	0.01	146.6	146.4	0.02
4.51	8.6, 6.2	4.52	8.4, 6.4	-0.01	138.8	138.7	0.01
4.05	10.4	4.05	10.4	0.00	137.1	136.8	0.03
3.98	10.4, 8.7	3.99	10.0, 9.2	-0.01	132.4	132.2	0.02
3.84	-	3.84	-	0.00	127.9	127.7	0.02
3.81	-	3.82	-	-0.01	110.1	109.9	0.02
3.01-2.86	-	2.97<br>2.92	15.6, 11.6, 5.2	-	108.6	108.4	0.02
2.68-2.48		2.66-2.54	-	0.02	106.7	106.4	0.03
					101.3	101.1	0.02
					71.1	70.9	0.02
					61.0	60.8	0.02
					56.3	56.1	0.02
					48.8	48.6	0.02
					46.9	46.7	0.02
					40.3	40.1	0.02
					33.1	32.9	0.02

Recovered substrate **2a**: 25 mg, 62 µmol, 26 % yield; $[\alpha]_D^{20}$ = 0.7 ($CHCl_3$, c = 2.6), ee = 10 % (for conditions of determination see Table SI15 and HPLC chromatograms); other analytical data are in accordance with that one reported for the racemate above.

Upscale of substrate 2c.

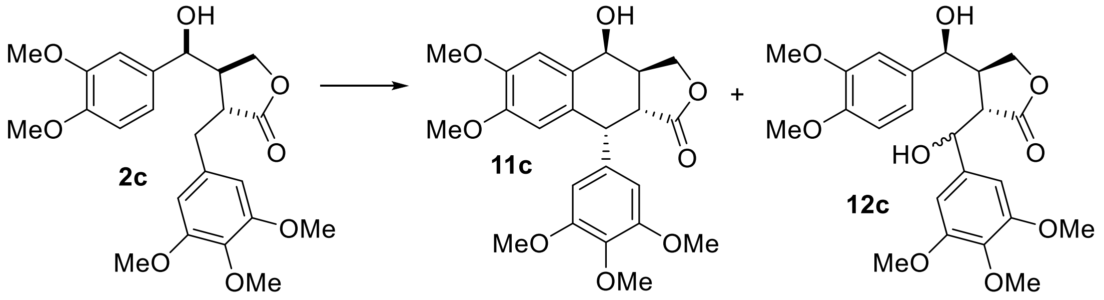

Substrate **2c** (100 mg, 230 µmol); column chromatography (SiO_2, cyclohexane/EtOAc 1/1); Preparative HPLC seperation of **11c** from **12c**: stationary phase: Phenomenex LUNA AXIA™ pack (5 µm, C18(2), 100 Å, 250 x 21.2 mm, 30 mL/min flow, eluent: H_2O/MeCN; gradient: 10% MeCN for 2 min, 10 to 100% MeCN over 23 min, 100% MeCN for 5 min;

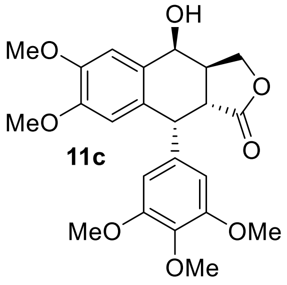

11c

(3a*R*,4*S*,9*R*,9a*R*)-4-hydroxy-6,7-dimethoxy-9-(3,4,5-trimethoxyphenyl)-3a,4,9,9a-tetrahydronaphtho[2,3-c]furan-1(3*H*)-one (11c): yield: yellow resin, 7.0 mg, 16 µmol, 7%, d.r. = >95:<5; $[\alpha]_D^{20}$ = -56.2 ($CHCl_3$, c = 0.8); ^{1}H-NMR (300 MHz, $CDCl_3$): 6.91 (s, 1H), 6.56 (s, 1H), 6.27 (s, 2H), 4.90 (d, *J* = 3.4 Hz, 1H), 4.66 (d, *J* = 5.0 Hz, 1H), 4.45 – 4.35 (m, 2H), 3.94 (s, 3H), 3.81 (s, 3H), 3.80 (s, 3H), 3.72 (s, 6H), 3.28 (dd, J_1 = 14.1, J_2 = 5.1 Hz, 1H), 2.90 – 2.75 (m, 1H); ^{13}C-NMR (75 MHz, $CDCl_3$): 175.3, 152.8, 150.0, 149.0, 137.4, 135.4, 130.8, 130.6, 113.2, 111.9, 108.4, 67.8, 66.9, 60.9, 56.4, 56.20, 56.16, 43.7, 40.9, 38.5; IR (film) ῦ = 3472 (br), 2919, 2850, 1766, 1589, 1508, 1461, 1353, 1290, 1244, 1186, 1159, 1123, 1103, 1028, 859, 744; HRMS(ESI): *m/z*: calc. for $C_{23}H_{26}O_8NH_4^+$: 448.1966 $[M+NH_4]^+$, found: 448.1972.

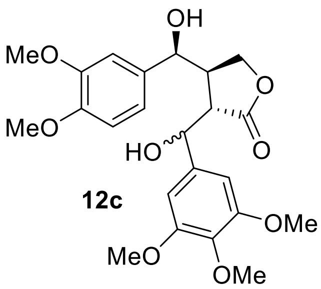

12c

(3*S*,4*R*)-4-[(*S*)-(3,4-dimethoxyphenyl)(hydroxy)methyl]-3-[hydroxy(3,4,5-trimethoxyphenyl)methyl]dihydrofuran-2(3*H*)-one (12c): yield: yellow resin, 3.4 mg, 8 µmol, 3.5%, d.r. = >95:<5; $[\alpha]_D^{20}$ = -10.0 ($CHCl_3$, c = 0.17); ^{1}H-NMR (300 MHz, $CDCl_3$): 6.72 – 6.66 (m, 2H), 6.55 (dd, J_1 = 8.2, J_2 = 2.0 Hz, 1H), 6.45 (s, 2H), 5.18 (d, *J* = 3.8 Hz, 1H), 4.65 (d, *J* = 6.0 Hz, 1H), 4.26 (t, *J* = 8.6 Hz, 1H), 4.14 (dd, J_1 = 9.0, J_2 = 4.9 Hz, 1H), 3.86 (s, 3H), 3.83 (s, 3H), 3.82 (s, 3H), 3.81 (s, 6H), 3.02 (dd, J_1 = 5.2, J_2 = 3.9 Hz, 1H), 2.80 (dq, J_1 = 8.2, J_2 = 5.3 Hz, 1H); ^{13}C-NMR (75 MHz, $CDCl_3$): 178.7, 153.4, 149.2, 148.9, 137.5, 136.7, 133.4, 118.1, 111.1, 108.8, 102.9, 75.2, 73.5, 70.5, 61.0, 56.2, 56.0, 49.2, 43.1; IR (film) ῦ = 3482 (br), 2924, 2851, 1744, 1593, 1513, 1456, 1418, 1390, 1331, 1256, 1232, 1186, 1123, 1081, 1023, 747; HRMS(ESI): *m/z*: calc. for $C_{23}H_{28}O_9NH_4^+$: 466.2072 $[M+NH_4]^+$, found: 466.2081.

Recovered substrate **2c**: 56 mg, 130 µmol, 57 % yield; $[\alpha]_D^{20}$ = +7.3 ($CHCl_3$, c = 1.5), ee = 23% (for conditions of determination see Table SI15 and HPLC chromatograms); other analytical data are in accordance with that one reported for the racemate above.

(3*S*,4*R*)-3-[Hydroxy(3,4,5-trimethoxyphenyl)methyl]-4-[(*S*)-hydroxy(phenyl)methyl]dihydrofuran-2(3*H*)-one (12e).

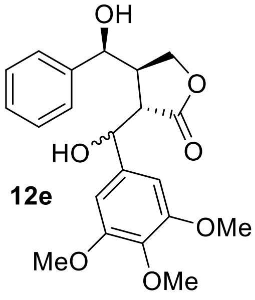

12e

Substrate **2e** (50 mg, 134 µmol); column chromatography (SiO_2, cyclohexane/EtOAc 1/1); yield: **12e** (white solid, 16.7 mg, 43 µmol, 32%, d.r. = >95:<5): $[\alpha]_D^{20}$ = -22.1 ($CHCl_3$, c = 1.6); Mp: 154-155°C (from acetone-d6); ^{1}H-NMR (300 MHz, d3-MeCN): 7.10 – 7.01 (m, 3H), 6.98 – 6.92 (m, 2H), 6.29 (d, *J* = 0.7 Hz, 2H), 5.02 (t, *J* = 3.4 Hz, 1H), 4.72 (t, *J* = 3.7 Hz, 1H), 4.43 (dd, J_1 = 8.7, J_2 = 7.9 Hz, 1H), 4.29 (dd, J_1 = 8.7, J_2 = 2.5 Hz, 1H), 3.693 (s, 6H), 3.685 (s, 3H), 3.64 (d, *J* = 4.1 Hz, 1H), 3.52 (d, *J* =

4.0 Hz, 1H), 2.79 (dq, J_1 = 8.2, J_2 = 2.8 Hz, 1H), 2.64 (t, J = 2.7 Hz, 1H); ^{13}C-NMR (75 MHz, d3-MeCN): 179.7, 153.9, 142.7, 138.4, 137.5, 128.7, 127.7, 126.2, 103.4, 75.0, 73.7, 72.9, 60.6, 56.4, 49.0, 42.7; IR (film) ῦ = 3504 (br), 2966, 2943, 2903, 2839, 1753, 1594, 1505, 1483, 1463, 1420, 1384, 1333, 1228, 1187, 1120, 1078, 1033, 996; HRMS(ESI): *m/z*: calc. for $C_{21}H_{24}O_7Na^+$: 411.1414 $[M+Na]^+$, found: 411.1419.

Recovered substrate **2e**: 22.1 mg, 44 % yield; $[\alpha]_D^{20}$ = +11.9 ($CHCl_3$, c = 2.20), ee = 93% (for conditions of determination see Table SI15 and HPLC chromatograms); other analytical data are in accordance with that one reported for the racemate above.

(3*S*,4*R*)-3-[Hydroxy(3,4,5-trimethoxyphenyl)methyl]-4-[(*S*)-hydroxy(naphthalen-2-yl)methyl]dihydrofuran-2(3*H*)-one (2f).

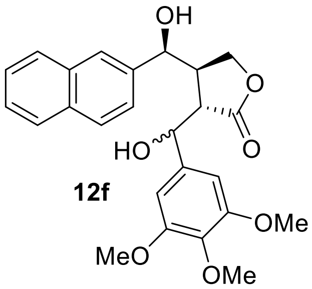

12f

Substrate **2f** (40 mg, 95 µmol); column chromatography (SiO_2, cyclohexane/EtOAc 1/1); yield: **12f** (yellow oil, 9.1 mg, 21 µmol, 22%, d.r. = >95:<5): $[\alpha]_D^{20}$ = +7.9 ($CHCl_3$, c = 0.53); ^{1}H-NMR (300 MHz, d3-MeCN): 7.82 – 7.77 (m, 1H), 7.75 – 7.70 (m, 1H), 7.67 (s, 1H), 7.48 – 7.38 (m, 3H), 6.81 (dd, J_1 = 8.6, J_2 = 1.8 Hz, 1H), 6.17 (d, J = 0.7 Hz, 2H), 4.98 (t, J = 3.5 Hz, 1H), 4.90 (t, J = 3.5 Hz, 1H), 4.51 (dd, J_1 = 8.6, J_2 = 7.8 Hz, 1H), 4.38 (dd, J_1 = 8.6, J_2 = 2.3 Hz, 1H), 3.65 (d, J = 3.6 Hz, 1H), 3.60 (dd, J_1 = 4.3, J_2 = 1.4 Hz, 1H), 3.56 (s, 3H), 3.36 (s, 6H), 2.98 – 2.83 (m, 1H), 2.75 – 2.65 (m, 1H); ^{13}C-NMR (75 MHz, d3-MeCN): 179.7, 153.7, 140.4, 138.3, 137.5, 133.9, 133.3, 128.7, 128.6, 128.3, 126.9, 126.5, 124.8, 124.4, 103.1, 75.1, 73.6, 73.1, 60.6, 56.1, 48.7, 42.2; IR (film) ῦ = 3435 (br), 2927, 2852, 1743, 1594, 1507, 1456, 1418, 1231, 1186, 1122, 1079, 1011, 818; HRMS(ESI): *m/z*: calc. for $C_{25}H_{26}O_7Na^+$: 461.1571 $[M+Na]^+$, found: 461.1576.

Recovered substrate **2f**: 8.6 mg, 22 % yield; $[\alpha]_D^{20}$ = -4.3 ($CHCl_3$, c = 0.85), ee = 89% (for conditions of determination see Table SI15 and HPLC chromatograms); other analytical data are in accordance with that one reported for the racemate above.

(3*S*,4*R*)-4-[(*S*)-(3-chlorophenyl)(hydroxy)methyl]-3-[hydroxy(3,4,5-trimethoxyphenyl)methyl]dihydrofuran-2(3*H*)-one (12g).

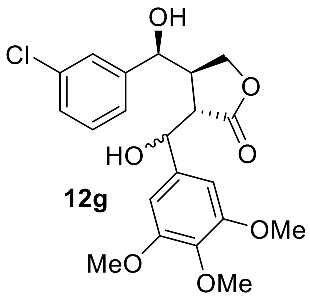

12g

Substrate **2g** (96.3 mg, 240 µmol); column chromatography (SiO_2, cyclohexane/EtOAc 2/1 then 1/1); yield: **12g** (white solid, 43.3 mg, 102 µmol, 43%, d.r. = >95:<5):

$[\alpha]_D^{20}$ = + 19.46 (CH_3CN, c = 1.86). Mp = 169-172 °C (from acetone). ^{1}H NMR (300 MHz, acetone-d6) δ 7.16 (s, 1H), 7.06 – 6.92 (m, 2H), 6.87 – 6.79 (m, 1H), 6.39 (s, 2H), 5.13 – 5.07 (m, 1H), 4.95 (dd, J_1 = 4.3, J_2 = 0.7 Hz, 1H), 4.89 (dt, J_1 = 4.6, J_2 = 2.4 Hz, 2H), 4.52 (t, J = 8.3 Hz, 1H), 4.37 (dd, J_1 = 8.5, J_2

= 2.4 Hz, 1H), 3.71 (dd, J_1 = 7.9, J_2 = 5.9 Hz, 9H), 2.95 – 2.88 (m, 1H), 2.66 – 2.59 (m, 1H). ^{13}C NMR (75 MHz, acetone-d6) δ 179.0, 153.7, 145.4, 138.5, 137.4, 133.7, 129.8, 127.2, 126.0, 124.4, 102.9, 74.1, 73.3, 72.5, 60.1, 55.9, 48.6, 42.3. IR (film) ῦ = 3506 (br), 3440, 2964, 2940, 1750, 1595, 1506, 1458, 1420, 1386, 1327, 1277, 1187, 1118, 1077, 1029, 797, 740; HRMS(ESI): no molecular peak was observed, due to the elimination of H_2O; *m/z*: calc. for $C_{21}H_{22}ClO_6^+$: 405.1099 ($[M+H]^+$ - H_2O), found: 405.1101.

Recovered substrate **2g**: 36.4 mg, 91 µmol, 37 % yield; $[\alpha]_D^{20}$ = +17.99 ($CHCl_3$, c = 2.24), ee = 95% (for conditions of determination see Table SI15 and HPLC chromatograms); other analytical data are in accordance with that one reported for the racemate above.

(3*S*,4*R*)-4-[(*S*)-(4-chlorophenyl)(hydroxy)methyl]-3-[hydroxy(3,4,5-trimethoxyphenyl)methyl]dihydrofuran-2(3*H*)-one (**12h**).

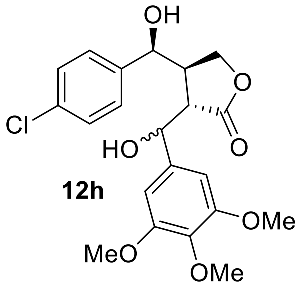

12h

Substrate **2h** (98 mg, 240 µmol); column chromatography (SiO_2, cyclohexane/EtOAc 1/1); yield: **12h** (yellow resin, 31 mg, 73 µmol, 30%, d.r. = >95:<5):

$[\alpha]_D^{20}$ = -8.7 (MeCN, c = 0.9); ^{1}H-NMR (300 MHz, acetone-d6): 7.11 – 6.98 (m, 4H), 6.40 (d, J = 0.7 Hz, 2H), 5.10 (br s, 1H), 4.93 – 4.83 (m, 3H), 4.51 (t, J = 8.2 Hz, 1H), 4.36 (dd, J_1 = 8.5, J_2 = 2.3 Hz, 1H), 3.76 (s, 3H), 3.72 (s, 6H), 2.89 (dt, J_1 = 7.9, J_2 = 2.7 Hz, 1H), 2.61 (t, J = 2.6 Hz, 1H); ^{13}C-NMR (75 MHz, acetone-d6): 179.1, 154.0, 142.0, 138.6, 137.8, 132.5, 128.5, 127.9, 103.3, 74.2, 73.5, 72.5, 60.5, 56.1, 48.9, 42.6, 42.5; IR (film) ῦ = 3396 (br), 2921, 2851, 1763, 1595, 1506, 1491, 1458, 1420, 1394, 1336, 1238, 1184, 1143, 1123, 1084, 1037, 1013, 984, 799, 716; HRMS(ESI): *m/z*: calc. for $C_{21}H_{23}ClO_7NH_4^+$: 440.1471 $[M+NH_4]^+$, found: 440.1473.

Crystals proper for X-ray diffraction were obtained from acetone/MeCN. The compound was dispersed in MeCN (ca. 1 mL) and acetone was added dropwise until all material was dissolved. The solvent was allowed to evaporate slowly and the crystals of **12h** were obtained.

Recovered substrate **2h**: 49 mg, 120 µmol, 50 % yield; $[\alpha]_D^{20}$ = +8.8 ($CHCl_3$, c = 1.63), ee = 63% (for conditions of determination see Table SI15 and HPLC chromatograms); other analytical data are in accordance with that one reported for the racemate above.

(3*S*,4*R*)-4-[(*S*)-(3-iodophenyl)(hydroxy)methyl]-3-[hydroxy(3,4,5-trimethoxyphenyl)methyl]dihydrofuran-2(3*H*)-one (**12i**).

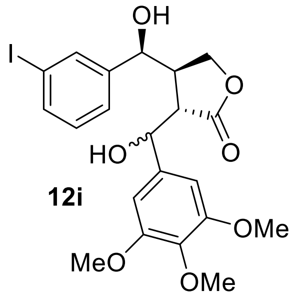

12i

Substrate **2i** (120 mg, 240 µmol); column chromatography (SiO_2, cyclohexane/EtOAc 2/1 then 1/1); yield: **12i** (white solid, 49 mg, 95 µmol, 40%, d.r. = >95:<5):

$[\alpha]_D^{20}$ = + 23.73 (CH_3CN, c = 2.25). Mp = 175-177 °C (from acetone). ^{1}H NMR (300 MHz, Acetone-d6) δ 7.57 (s, 1H), 7.37 (t, *J* = 8.2 Hz, 1H), 6.93 – 6.84 (m, 1H), 6.77 (t, *J* = 7.7 Hz, 1H), 6.42 (s, 2H), 5.13 – 5.09 (m, 1H), 4.94 – 4.73 (m, 3H), 4.56 – 4.44 (m, 1H), 4.41 – 4.30 (m, 1H), 3.78 – 3.70 (m, 9H), 2.92 (dt, J_1 = 15.2, J_2 = 6.3 Hz, 1H), 2.64 (d, *J* = 0.9 Hz, 1H). ^{13}C NMR (75 MHz, Acetone-d6) δ 179.1, 154.1, 145.9, 138.8, 137.8, 136.5, 135.3, 130.6, 125.8, 103.2, 94.4, 74.3, 73.7, 72.6, 60.4, 56.5, 49.0, 42.6. IR (film) ṽ = 3543 (br), 2974, 2924, 1713, 1586, 1497, 1455, 1434, 1339, 1286, 1178, 1137, 1041, 945, 779; HRMS(ESI): no molecular peak was observed, due to the elimination of H_2O; *m/z*: calc. for $C_{21}H_{22}IO_6^+$: 497.0456 ($[M+H]^+$ - H_2O), found: 497.0455.

Recovered substrate **2i**: 49 mg, 98 µmol 41 % yield; $[\alpha]_D^{20}$ = +14.23 ($CHCl_3$, c = 2.67), ee = 82% (for conditions of determination see Table SI15 and HPLC chromatograms); other analytical data are in accordance with that one reported for the racemate above.

Methyl 4-((1*S*)-hydroxy{(3*R*,4*S*)-4-[hydroxy(3,4,5-trimethoxyphenyl)methyl]-5-oxotetrahydrofuran-3-yl}methyl)benzoate (**12j**).

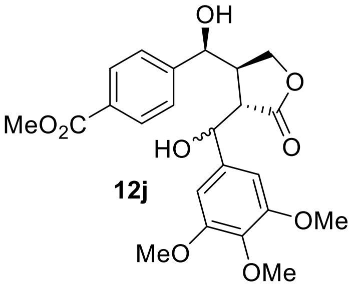

12j

Substrate **2j** (258.3 mg, 600 µmol); column chromatography (SiO_2, cyclohexane/EtOAc 1/1); yield: **12j** (colourless resin, 42.3 mg, 94 µmol, 16%, d.r. = >95:<5):

$[\alpha]_D^{20}$ = -10.5 (MeCN, c = 2.4); ^{1}H NMR (300 MHz, acetone-d6) δ 7.76 – 7.66 (m, 2H), 7.13 (d, *J* = 8.1 Hz, 2H), 6.30 (s, 2H), 5.11 – 5.00 (m, 2H), 4.96 (dd, J_1 = 3.8, J_2 = 2.7 Hz, 2H), 4.54 (t, *J* = 8.2 Hz, 1H), 4.41 (dd, J_1 = 8.4, J_2 = 2.1 Hz, 1H), 3.87 (d, *J* = 3.6 Hz, 3H), 3.70 – 3.62 (m, 9H), 2.93 – 2.81 (m, 1H), 2.60 (d, *J* = 0.8 Hz, 1H). ^{13}C NMR (75 MHz, acetone-d6) δ 178.9, 166.7, 153.4, 148.2, 138.0, 137.3, 129.2, 128.8, 125.9, 102.7, 74.2, 73.0, 72.5, 60.0, 55.7, 51.7, 48.3, 42.3; IR (film) ṽ = 3463 (br), 2941, 2838, 1761, 1716, 1611, 1505, 1456, 1433, 1418, 1378, 1336, 1236, 1121, 1032, 998, 933, 749; HRMS(ESI): *m/z*: calc. for $C_{23}H_{27}O_9^+$: 447.1650 $[M+H]^+$, found: 447.1648.

Recovered substrate **2j**: 157.6 mg, 366 µmol, 61 % yield; $[\alpha]_D^{20}$ = + 2.67 ($CHCl_3$, c = 1.50), ee = 21% (for conditions of determination see Table SI15 and HPLC chromatograms); other analytical data are in accordance with that one reported for the racemate above.

(3S,4R)-3-[hydroxy(3,4,5-trimethoxyphenyl)methyl]-4-[(S)-hydroxy(4-methoxyphenyl)methyl]dihydrofuran-2(3H)-one (**12k**).

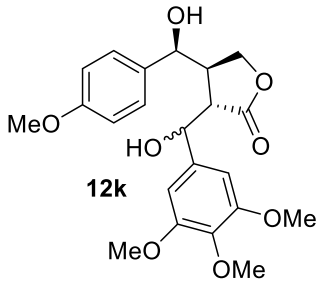

Substrate **2k** (91 mg, 240 µmol); column chromatography (SiO_2, cyclohexane/EtOAc 1/1); yield: **12k** (colourless resin, 34 mg, 81 µmol, 34%, d.r. = >95:<5):

$[\alpha]_D^{20}$ = -4.7 ($CHCl_3$, c = 1.5); ^{1}H-NMR [300 MHz, $CDCl_3$ + MeOD-d3 (2 drops)]: 6.98 – 6.91 (m, 2H), 6.74 – 6.67 (m, 2H), 6.39 (s, 2H), 5.12 (d, J = 3.7 Hz, 1H), 4.62 (d, J = 5.7 Hz, 1H), 4.26 (t, J = 8.6 Hz, 1H), 4.13 (dd, J_1 = 9.0, J_2 = 4.7 Hz, 1H), 3.80 (s, 3H), 3.78 (s, 6H), 3.77 (s, 3H), 2.93 (dd, J_1 = 5.0, J_2 = 3.8 Hz, 1H), 2.78 (dq, J_1 = 8.1, J_2 = 5.0 Hz, 1H); ^{13}C-NMR [75 MHz, $CDCl_3$ + MeOD-d3 (2 drops)]: 179.0, 159.3, 153.2, 137.2, 136.8, 132.9, 126.9, 113.9, 102.7, 74.8, 73.3, 70.7, 60.9, 56.1, 55.3, 42.9, 42.8; IR (film) ῦ = 3476 (br), 2938, 2838, 1747, 1593, 1510, 1459, 1419, 1330, 1297, 1235, 1177, 1122, 1078, 1028, 1005, 911, 841, 811, 724; HRMS(ESI): m/z: calc. for $C_{22}H_{26}O_8Na^+$: 441.1520 $[M+Na]^+$, found: 441.1523.

Recovered substrate **2k**: 38 mg, 100 µmol, 42 % yield; $[\alpha]_D^{20}$ = +19.1 (MeCN, c = 3.8), ee = 63% (for conditions of determination see Table SI15 and HPLC chromatograms); other analytical data are in accordance with that one reported for the racemate above.

Upscale of substrate 2l.

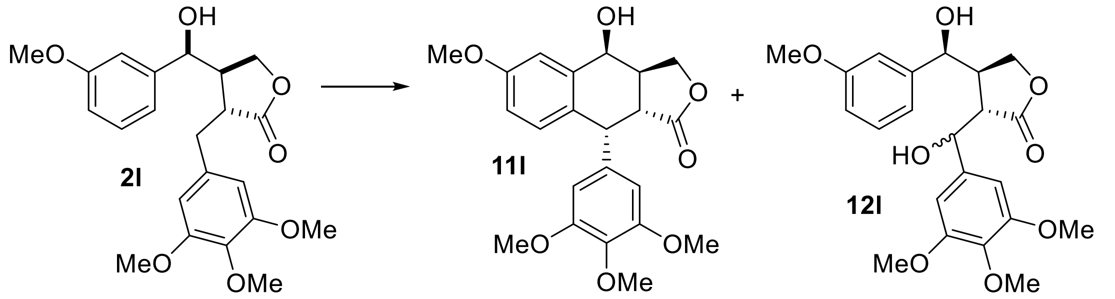

Substrate **2l** (241.5 mg, 600 µmol); column chromatography (SiO_2, cyclohexane/EtOAc 1/1); yield:

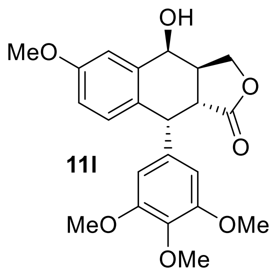

11l (colourless oil, 78.3 mg, 197 µmol, 33 %, d.r. = >95:<5): $[\alpha]_D^{20}$ = -82.91 ($CHCl_3$, c = 1.27); ^{1}H NMR (300 MHz, $CDCl_3$) δ 7.05 (d, J = 8.6 Hz, 1H), 6.95 (d, J = 2.6 Hz, 1H), 6.88 (dd, J_1 = 8.5, J_2 = 2.6 Hz, 1H), 6.23 (s, 2H), 4.91 (d, J = 3.0 Hz, 1H), 4.67 (d, J = 5.1 Hz, 1H), 4.46 – 4.29 (m, 2H), 3.83 (s, 3H), 3.78 (s, 3H), 3.69 (s, 6H), 3.30 (dd, J_1 = 14.1, J_2 = 5.1 Hz, 1H), 2.94 – 2.74 (m, 1H), 2.22 (s, 1H). ^{13}C NMR (75 MHz, $CDCl_3$) δ 175.4, 159.1, 152.6, 139.7, 137.1, 135.9, 132.6, 130.0, 115.8, 114.5, 108.2, 67.7, 67.0, 60.8, 56.3, 55.5, 43.2, 40.9, 38.4; IR (film) ῦ = 3447 (br), 3011, 2937, 2837, 1768, 1610, 1588, 1498, 1457, 1419,

1330, 1237, 1122, 1078, 1033, 1000, 863, 746, 665; HRMS(ESI): *m/z*: calc. for $C_{22}H_{24}O_7NH_4^+$: 418.1860 $[M+NH_4]^+$, found: 418.1860.

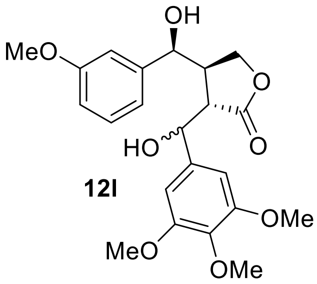

12l

12l (white resin, 4 mg, 10 µmol, 2 %, d.r. = >95:<5): $[\alpha]_D^{20}$ = +3.5 ($CHCl_3$, c = 0.4); Mp = 178-181 °C (from acetone). ^{1}H NMR (300 MHz, acetone-d6) δ 6.94 (t, *J* = 7.9 Hz, 1H), 6.65 – 6.62 (m, 1H), 6.61 – 6.50 (m, 2H), 6.39 (s, 2H), 5.11 – 5.06 (m, 1H), 4.94 (d, *J* = 4.2 Hz, 1H), 4.79 (d, *J* = 1.6 Hz, 2H), 4.48 (t, *J* = 8.2 Hz, 1H), 4.34 (dd, J_1 = 8.4, J_2 = 2.6 Hz, 1H), 3.72 (s, 6 H), 3.70 (s, 6 H), 2.93 – 2.83 (m, 1H), 2.71 (dd, J_1 = 2.5, J_2 = 1.5 Hz, 1H). ^{13}C NMR (75 MHz, Acetone) δ 179.1, 159.7, 153.5, 144.5, 138.4, 137.3, 129.1, 118.1, 112.4, 111.7, 102.9, 74.6, 73.3, 72.3, 60.0, 55.8, 54.8, 48.7, 42.5; IR (film) ṽ = 3509 (br), 2966, 2945, 2841, 1760, 1596, 1506, 1434, 1375, 1330, 1234, 1187, 1124, 1084, 1028, 1020, 998, 777; HRMS(ESI): no molecular peak was observed, due to the elimination of H_2O; *m/z*: calc. for $C_{22}H_{25}O_7^+$: 401.1595 ($[M+H]^+$ - H_2O), found: 401.1594.

Recovered substrate **2l**: 94.0 mg, 233 µmol, 39 % yield; $[\alpha]_D^{20}$ = +24.44 ($CHCl_3$, c = 1.50), ee = 92% (for conditions of determination see Table SI15 and HPLC chromatograms); other analytical data are in accordance with that one reported for the racemate above.

(3*S*,4*R*)-4-[(*S*)-(4-fluorophenyl)(hydroxy)methyl]-3-[hydroxy(3,4,5-trimethoxyphenyl)methyl]dihydrofuran-2(3*H*)-one (**12m**).

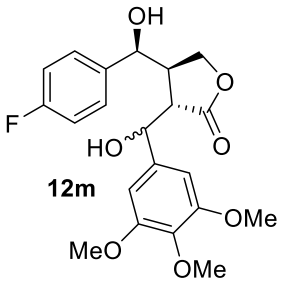

12m

Substrate **2m** (281.1 mg, 720 µmol); column chromatography (SiO_2, cyclohexane/EtOAc 1/1); yield: **12m** (white solid, 78.9 mg, 197 µmol, 27%, d.r. = >95:<5):

$[\alpha]_D^{20}$ = -8.9 (MeCN, c = 1.0); Mp = 161-164 °C (from acetone). ^{1}H NMR (300 MHz, acetone-d6) δ 7.10 – 6.98 (m, 2H), 6.87 – 6.72 (m, 2H), 6.39 (s, 2H), 5.12 – 5.07 (m, 1H), 4.95 (dd, J_1 = 4.3, J_2 = 1.2 Hz, 1H), 4.90 (d, *J* =3.8 Hz, 1H), 4.85 (t, *J* = 3.5 Hz, 1H), 4.50 (t, *J* = 8.2 Hz, 1H), 4.35 (dd, J_1 = 8.5, J_2 = 2.3 Hz, 1H), 3.72 (s, 6H), 3.72 (s, 3H), 2.88 (ddd, J_1 = 7.2, J_2 = 4.7, J_3 = 2.2 Hz, 1H), 2.63 (dd, J_1 = 2.9, J_2 = 1.8 Hz, 1H). ^{13}C NMR (75 MHz, acetone-d6) δ 178.9, 161.8 (d, *J* = 242.7 Hz), 153.5, 138.7 (d, *J* = 2.9 Hz), 138.3, 137.3, 127.6 (d, *J* = 8.1 Hz), 114.7 (d, *J* = 21.3 Hz), 102.9, 73.9, 73.1, 72.2, 60.0, 55.7, 48.6, 42.3; IR (film) ṽ = 3414 (br), 3001, 2937, 2888, 2841, 1758, 1596, 1507, 1459, 1419, 1389, 1328, 1188, 1159, 1123, 1083, 1016, 985, 822, 722; HRMS(ESI): no molecular peak was observed, due to the elimination of H_2O; *m/z*: calc. for $C_{21}H_{22}FO_6^+$: 389.1395 ($[M+H]^+$ - H_2O), found: 389.1395.

Recovered substrate **2m**: 109.9 mg, 281 µmol, 39 % yield; $[\alpha]_D^{20}$ = +21.12 ($CHCl_3$, c = 1.07), ee = 97% (for conditions of determination see Table SI15 and HPLC chromatograms); other analytical data are in accordance with that one reported for the racemate above.

Two gram upscale for the preparation of *epi*-podophyllotoxin (**11d**).

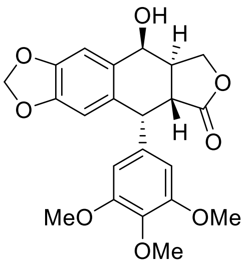

epi-Podophyllotoxin (**11d**)
2 GRAM UPSCALE

CFE (144 mL) was combined with a stock solution of 2-oxoglutarate (1.03 g, 7.06 mmol) and sodium ascorbate (1.49 g, 7.52 mmol) in TRIS buffer (36 mL, pH = 7.4, 200 mM, 100 mM NaCl) in a 2 L flask. Substrate **2d** (2.000 g, 4.76 mmol) was dissolved in DMSO (55 mL) and added to the reaction mixture. The flask was capped (cap allowed exchange of air) and placed into a shaker at 18 °C and 150 rpm for 18 h. The mixture was extracted with EtOAc (3 x 200 mL), the phases were separated via centrifugation (15 min at 8000 rpm). The combined organic phase was dried over Na_2SO_4, filtered and the filtrate concentrated to give the crude product. The latter was purified via flash chromatography (SiO_2, cyclohexane/EtOAc 6/4) to give the target compound *epi*-podophyllotoxin (**11d**, 770 mg, 1.84 mmol, 39%, d.r. = >95:<5) as a pale yellow oil with the same physical data as reported above.

Recovered substrate **2d**: 998 mg, 2.38 mmol, 45 % yield, ee = 66% (for conditions of determination see Table SI15 and HPLC chromatograms); other analytical data are in accordance with that one reported for the racemate above.

(3*S*,4*R*)-4-[(R)-(-Benzo[d][1,3]dioxol-5-yl(hydroxy)methyl]-3-[hydroxy(3,4,5-trimethoxyphenyl)methyl]dihydrofuran-2(3*H*)-one (**12b**).

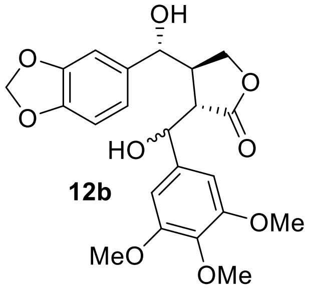

12b

Substrate **2b** (42 mg, 0.10 mmol); column chromatography (SiO_2, cyclohexane/EtOAc 1/1); yield: **12b** (pale yellow oil, 6.0 mg, 14 µmol, 15 %, d.r. = >95:<5):

$[\alpha]_D^{20}$ = + 14.66 ($CHCl_3$, c = 0.6). ^{1}H NMR (300 MHz, CD_3CN) δ 6.77 (d, J = 7.9 Hz, 1H), 6.71 – 6.61 (m, 2H), 6.54 (s, 2H), 5.93 (s, 2H), 4.63 (dd, J_1 = 5.8, J_2 = 3.6 Hz, 1H), 4.28 – 4.19 (m, 2H), 4.06 – 3.97 (m, 1H), 3.93 (d, J = 3.6 Hz, 1H), 3.77 (d, J = 7.4 Hz, 6H), 3.70 (d, J = 3.9 Hz, 3H), 3.59 (d, J = 4.3 Hz, 1H), 2.77 (t, J = 6.3 Hz, 1H), 2.64 (dq, J_1= 12.7, J_2 = 6.2 Hz, 1H). ^{13}C NMR (75 MHz, CD_3CN) δ 178.1, 154.0, 148.8, 147.9, 138.3, 138.2, 137.7, 120.4, 108.7, 107.1, 104.4, 102.3, 73.9, 73.3, 68.6, 60.8, 56.6, 50.1, 46.3. IR (film) ṽ = 3442 (br), 2921, 1748, 1592, 1504, 1487, 1458, 1444, 1420, 1327, 1234, 1180, 1121, 1033, 999, 923, 728; HRMS(ESI): no molecular peak was observed, due to the elimination of H_2O; *m/z*: calc. for $C_{22}H_{23}O_8^+$: 415.1387 ($[M+NH_4]^+$ - H_2O), found: 415.1387.

Recovered substrate **2b**: 16.8 mg, 40 % yield; $[\alpha]_D^{20}$ = +6.47 ($CHCl_3$, c = 1.90), ee = 46% (for conditions of determination see Table SI15 and HPLC chromatograms); other analytical data are in accordance with that one reported for the racemate above.

Podophyllotoxone (SI-42).

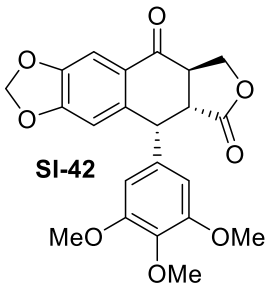

SI-42

epi-Podophyllotoxin **11d** (500 mg, 120.6 mmol, 1.0 eq) was dissolved in anhydrous CH_2Cl_2 (12.5 mL) and pyridine was added (195 µL, 241.2 mmol, 2.0 eq). The mixture was cooled to 0 °C for 15 minutes, then Dess-Martin Periodinane (DMP) was added (767.5 mg, 180.9 mmol, 1.5 eq) and stirred overnight till TLC showed full conversion. The reaction was quenched with saturated aqueous NH_4Cl (25 mL) and the mixture was extracted with CH_2Cl_2 (3 x 20 mL). The combined organic phase was washed with brine (30 mL) and then dried over anhydrous Na_2SO_4 and then evaporated. The crude product was purified by flash column chromatography (SiO_2, cyclohexane/EtOAc 1:1) to give compound **SI-42** (377 mg, 91.5 mmol, 76 %, d.r. = >95:<5) as a pale yellow solid.

$[\alpha]_D^{20}$ = -92.8 ($CHCl_3$, c = 1.04), lit.: $[a]_D^{20}$ = -106.98 ($CHCl_3$, c = 1.08),[20] Mp = 164-167 °C (from $CDCl_3$). ^{1}H NMR (300 MHz, $CDCl_3$) δ 7.52 (s, 1H), 6.68 (s, 1H), 6.36 (s, 2H), 6.07 (dd, J_1 = 5.3, , J_2 = 1.0 Hz, 2H), 4.83 (d, J = 4.2 Hz, 1H), 4.54 (dd, , J_1 = 9.2, , J_2 = 7.6 Hz, 1H), 4.33 (dd, J_1 = 10.2, , J_2 = 9.4 Hz, 1H), 3.79 (s, 3H), 3.73 (s, 6H), 3.58 – 3.44 (m, 1H), 3.27 (dd, J_1 = 15.5, J_2 = 4.3 Hz, 1H). ^{13}C NMR (75 MHz, $CDCl_3$) δ 192.5, 173.2, 153.3, 153.1, 148.2, 141.6, 137.7, 132.2, 128.2, 109.7, 107.7, 106.1, 102.5, 67.1, 60.8, 56.3, 46.7, 44.7, 43.5. IR (film) ṽ = 2904, 2837, 1781, 1683, 1586, 1504, 1477, 1447, 1430, 1242, 1120, 1032, 994, 764; HRMS(ESI): *m/z*: calc. for $C_{22}H_{20}O_8NH_4^+$: 430.1496 $[M+NH_4]^+$, found: 430.1496.

Podophyllotoxin (1).

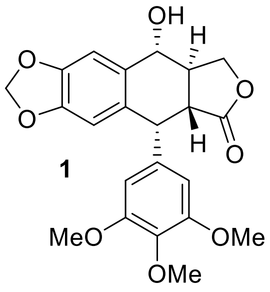

1

To a solution of podophyllotoxone **SI-42** (300.0 mg, 0.720 mmol, 1.0 eq) in anhydrous THF (60 mL) was added a solution of L-Selectride (1.0 M in THF, 960 µL, 0.946 mmol, 1.3 eq) at −78 °C under argon. The resulting mixture was stirred at −78 °C for 2 h then treated with saturated aqueous NH_4Cl (20 mL). The mixture was stirred for 30 minutes at room temperature. The reaction mixture was extracted with EtOAc (3 × 180 mL). The combined organic phases were washed with water (2 × 90 mL) and brine (90 mL) respectively, dried over Na_2SO_4, filtered and concentrated under reduced pressure. The residue was purified by flash column chromatography (SiO_2, cyclohexane/EtOAc 2/1 then 1/1) to give **1** (228 mg, 70% yield, d.r. = >95:<5) as colourless resin.

$[\alpha]_D^{20}$ = -132.7 ($CHCl_3$, c = 1.00), lit: $[\alpha]_D^{20}$ = -115.0 ($CHCl_3$, c = 1.00);[20] ^1H NMR (300 MHz, $CDCl_3$) δ 7.11 (s, 1H), 6.49 (s, 1H), 6.36 (s, 2H), 5.98 (d, 1H), 5.96 (d, 1H), 4.75 (d, *J* = 7.4 Hz, 1H), 4.58 (m, 2H), 4.06 (m, 1H), 3.80 (s, 3H), 3.74 (s, 6H), 2.89 – 2.68 (m, 2H), 2.47 (brs, 1H). ^{13}C NMR (75 MHz, $CDCl_3$) δ 174.7, 152.7, 147.8, 147.7, 137.2, 135.6, 133.3, 131.2, 109.8, 108.5, 106.4, 101.6, 72.8, 71.5, 60.9, 56.4, 45.4, 44.2, 40.8; IR (film) ῦ = 3461 (br), 3010, 2938, 2888, 2838, 1758 1596, 1503, 1480, 1461, 1419, 1329, 1291, 1233, 1185, 1122, 1036, 995, 954, 841, 748, 730; HPLC-UV analysis on a chiral stationary phase {Daicel Chiralpak AD (4.6 x 250 mm, 10 µm particle size), n-heptane/2-propanol 70/30, 1.0 mL/min, 30 °C, UV 215 nm, t_{ret}[(-)-podophyllotoxin] = 8.2 min, t_{ret}[(+)-podophyllotoxin] = 14.0 min}:[16] t_{ret}(compound **1**) = 7.4 min, ee = >99%; HRMS(ESI): *m*/*z*: calc. for $C_{22}H_{22}O_8NH_4^+$: 432.1653 $[M+NH_4]^+$, found: 432.1653.

Table SI-19. Comparison of ^1H-NMR- and ^{13}C-NMR-data of podophyllotoxin (**1**)

^1H-NMR					^{13}C-NMR		
observed		*literature*[20]			*observed*	*literature* [20]	
ppm	*J*	ppm	*J*	Δppm	ppm	ppm	Δppm
7.11	-	7.11	-	0.00	174.7	174.6	0.01
6.49	-	6.51	-	0.02	152.7	152.5	0.02
6.36	-	6.37	-	-0.01	147.8	147.7	0.01
5.97	-	5.98	-	-0.01	146.7	147.6	0.01
5.95	-	5.96	-	-0.01			
4.75	7.4	4.77	8.4	-0.02	137.2	137.1	0.01
4.58	-	4.60	8.0	-	135.6	135.5	0.01
		4.59	4.4	-			
4.06	-	4.08	9.8, 8.8	-0.02	133.3	133.3	0.00
3.80	-	3.81	-	-0.01	131.2	131.0	0.02
3.74	-	3.75	-	-0.01	119.8	109.7	0.01
2.89-2.68	-	2.84	-		108.5	108.4	0.01
		2.83-2.74					
2.47	br s	2.13	8.0	-	106.4	106.3	0.01
					101.6	101.4	0.02
					72.8	72.6	0.02
					71.5	71.4	0.01
					60.9	60.7	0.02
					56.4	56.2	0.02
					45.4	45.2	0.02
					44.2	44.1	0.01
					40.8	40.6	0.02

Control experiment for an enzyme-catalyzed ring closure (see page 12 in this supporting information) – experimental procedure.

In a 1.5 mL Eppendorf vial, CFE (50 µL, 3.3 mg/mL 2-ODD-PH protein) was combined with a stock solution [12.5 µL; 2-oxoglutarate (82.8 mg, 567 µmol) and sodium ascorbate (119.8 mg, 605 µmol) in TRIS buffer (0.5 mL; pH = 7.4, 200 mM, 100 mM NaCl); pH of the stock solution was adjusted to pH = 7.4 after addition of the reactants and filled to a final volume of 2 mL with buffer]. The substrate **2l** was solubilized in DMSO (88 mM DMSO stock solution) and added to the reaction vial (19 µL, for a final concentration of 20 mM). Afterwards the vial was closed and placed in a bench shaker at 18 °C and 700 rpm in horizontal position for 18 hours. The mixture was extracted with EtOAc (2 x 350 µL). The phases were separated via centrifugation (1 min at 13000 rpm) and decanting. The organic phases were combined and dried over Na_2SO_4, centrifuged and the supernatant was subjected to HPLC-UV analysis.

Crystal Structure Determination of Compound **12h**

All the measurements were performed using monochromatized Mo K_α radiation at 100K: $C_{21}H_{23}ClO_7 \cdot C_2H_3N$, M_r 463.90, orthorhombic, space group P 2_1 2_1 2_1, a = 11.6472(7)Å, b = 12.1108(7)Å, c = 15.8838(9)Å, V = 2240.5(2)Å^3, Z = 4, d_{calc} = 1.375g cm^{-3}, μ = 0.215mm^{-1}. A total of 16147 reflections were collected (Θ_{max} = 28.0°), from which 5402 were unique (R_{int} = 0.0336), with 4925 having I > 2σ(I). The structure was solved by direct methods (SHELXS-97)[24] and refined by full-matrix least-squares techniques against F^2 (SHELXL-2014/6).[25] The non-hydrogen atoms were refined with anisotropic displacement parameters without any constraints. The absolute configuration was established by anomalous dispersion effects in the diffraction measurements on the crystal. The positions of the H atoms of the OH groups were taken from a difference Fourier map, the O–H distances were fixed to 0.84Å, and the H atoms were refined with individual isotropic displacement parameters without any constraints to the bond angles. The H atoms of the tertiary C–H groups were refined with a common isotropic displacement parameter and all X–C–H angles equal at a C–H distance of 1.00Å. The H atoms of the CH_2 group were refined with a common isotropic displacement parameters and idealized geometry with approximately tetrahedral angles and C–H distances of 0.99Å. The H atoms of the phenyl rings were put at the external bisectors of the C–C–C angles at C–H distances of 0.95Å and common isotropic displacement parameters were refined for the H atoms of the same ring. The H atoms

of the methyl groups were refined with common isotropic displacement parameters for the H atoms of the same group and idealized geometries with tetrahedral angles, enabling rotations around the C–C bonds, and C–H distances of 0.98Å. For 309 parameters final *R* indices of R1 = 0.0332 and wR^2 = 0.0794 (GOF = 1.015) were obtained. The largest peak in a difference Fourier map was 0.262eÅ^{-3}.

Crystal Structure. The crystal structure analysis of product **12h** confirmed the compound as (3*S*,4*R*)-4-[(*S*)-(4-chlorophenyl)(hydroxy)methyl]-3-[(*S*)-hydroxy(3,4,5-trimethoxyphe-nyl)methyl]dihydrofuran-2(3*H*)-one acetonitrile solvate (1:1). All atoms lie on general positions (see Figure SI05). The absolute configuration was established by anomalous dispersion effects in the diffraction measurements on the crystal. The two phenyl rings are almost parallel [angle of 0.75(10)° between the least-squares planes through their C atoms] and show an intra-molecular pi stacking [distance of 3.9308(12)Å between their centroids; mean distance of 3.698(12)Å between a centroid and a least-squares plane]. The molecules are inter-connected by hydrogen bonds [O3···O34 2.762(2)Å, O3–H3···O34′ 163.2(11)°; O4···O3 2.860(2)Å, O4–H4···O3″ 165.7(8)°] forming a three-dimensional network. The non-coordinating acetonitrile solvent molecule occupies a void of approx. 76Å^3.

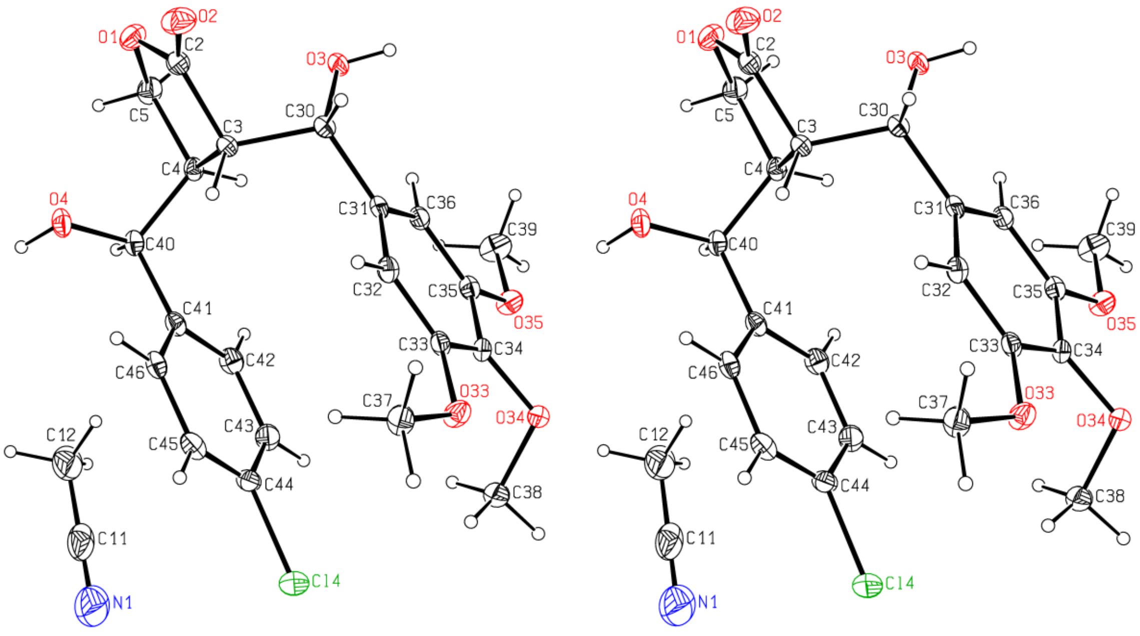

Figure SI05. Stereoscopic ORTEP[26] plot of product **12h** showing the atomic numbering scheme. The probability ellipsoids are drawn at the 50% probability level. The H atoms are drawn with arbitrary radii.

Table SI20. Crystal data and structure refinement for product **12h**.

Crystal data	
Identification code	FUMFE39
Empirical formula	$C_{21}H_{23}ClO_7 \cdot C_2H_3N$
Formula weight	463.90
Crystal description	block, colourless
Crystal size	0.35 x 0.32 x 0.26mm
Crystal system, space group	orthorhombic, P 2_1 2_1 2_1
Unit cell dimensions: a	11.6472(7)Å
b	12.1108(7)Å
c	15.8838(9)Å
Volume	2240.5(2)Å^3
Z	4
Calculated density	1.375Mg/m^3
F(000)	976
Linear absorption coefficient μ	0.215mm^{-1}
Absorption correction	semi-empirical from equivalents
Max. and min. transmission	1.000 and 0.768
Unit cell determination	2.17° < Θ < 30.19°
	5972 reflections used at 100K
Data collection	
Temperature	100K
Diffractometer	Bruker APEX-II CCD
Radiation source	Incoatec microfocus sealed tube
Radiation and wavelength	MoK$_\alpha$, 0.71073Å
Monochromator	multilayer monochromator

Scan type	ϕ and ω scans
Θ range for data collection	2.12 to 28.00°
Reflections collected / unique	16147 / 5402
Significant unique reflections	4925 with $I > 2\sigma(I)$
R(int), R(sigma)	0.0336, 0.0378
Completeness to Θ = 28.0°	99.9%

Refinement

Refinement method	Full-matrix least-squares on F^2
Data / parameters / restraints	5402 / 309 / 2
Goodness-of-fit on F^2	1.015
Final R indices [$I > 2\sigma(I)$]	R1 = 0.0332, wR2 = 0.0765
R indices (all data)	R1 = 0.0391, wR2 = 0.0794
Absolute structure parameter	0.04(3)
Extinction expression	none
Weighting scheme	$w = 1/[\sigma^2(F_o^2)+(aP)^2+bP]$ where $P = (F_o^2+2F_c^2)/3$
Weighting scheme parameters a, b	0.0366, 0.5891
Largest Δ/σ in last cycle	0.001
Largest difference peak and hole	0.262 and -0.261e/Å^3
Structure Solution Program	SHELXS-97 (Sheldrick, 2008)
Structure Refinement Program	SHELXL-2014/6 (Sheldrick, 2015)
CCDC deposition number	1889727

Table SI21. Hydrogen bonds for product **12h** [Å, °].

D-H···A	d(D-H)	d(H···A)	d(D···A)	<(DHA)
O(3)-H(3)···O(34)[i]	0.84	1.947(4)	2.762(2)	163.2(11)
O(4)-H(4)···O(3)[ii]	0.84	2.038(3)	2.860(2)	165.7(8)

Symmetry transformations used to generate equivalent atoms:
[i] x-1/2, 1/2-y, 1-z [ii] 1-x, y+1/2, 3/2-z

Table SI22. Full list of bond lengths [Å] and angles [°] for product **12h**.

O(1)-C(2)	1.352(3)
O(1)-C(5)	1.453(3)
C(2)-O(2)	1.200(3)
C(2)-C(3)	1.528(3)
C(3)-C(4)	1.537(3)
C(3)-C(30)	1.538(3)
C(3)-H(31)	1.00
C(4)-C(40)	1.534(3)
C(4)-C(5)	1.537(3)
C(4)-H(41)	1.00
C(5)-H(51)	0.99
C(5)-H(52)	0.99
C(30)-O(3)	1.433(2)
C(30)-C(31)	1.528(3)
C(30)-H(30)	1.00
O(3)-H(3)	0.84
C(31)-C(36)	1.392(3)
C(31)-C(32)	1.399(3)
C(32)-C(33)	1.393(3)
C(32)-H(32)	0.95
C(33)-O(33)	1.371(3)
C(33)-C(34)	1.403(3)
C(34)-O(34)	1.389(3)
C(34)-C(35)	1.396(3)
C(35)-O(35)	1.370(3)
C(35)-C(36)	1.399(3)

C(36)-H(36)	0.95
O(33)-C(37)	1.439(3)
C(37)-H(371)	0.98
C(37)-H(372)	0.98
C(37)-H(373)	0.98
O(34)-C(38)	1.446(3)
C(38)-H(381)	0.98
C(38)-H(382)	0.98
C(38)-H(383)	0.98
O(35)-C(39)	1.435(3)
C(39)-H(391)	0.98
C(39)-H(392)	0.98
C(39)-H(393)	0.98
C(40)-O(4)	1.429(3)
C(40)-C(41)	1.520(3)
C(40)-H(40)	1.00
O(4)-H(4)	0.84
C(41)-C(42)	1.394(3)
C(41)-C(46)	1.399(3)
C(42)-C(43)	1.390(3)
C(42)-H(42)	0.95
C(43)-C(44)	1.391(3)
C(43)-H(43)	0.95
C(44)-C(45)	1.384(3)
C(44)-Cl(4)	1.744(2)
C(45)-C(46)	1.393(3)
C(45)-H(45)	0.95
C(46)-H(46)	0.95
N(1)-C(11)	1.136(4)
C(11)-C(12)	1.456(5)
C(12)-H(121)	0.98
C(12)-H(122)	0.98
C(12)-H(123)	0.98

C(2)-O(1)-C(5)	110.93(17)
O(2)-C(2)-O(1)	121.9(2)
O(2)-C(2)-C(3)	127.4(2)
O(1)-C(2)-C(3)	110.75(19)
C(2)-C(3)-C(4)	104.49(17)
C(2)-C(3)-C(30)	108.36(18)
C(4)-C(3)-C(30)	114.95(17)
C(2)-C(3)-H(31)	109.6
C(4)-C(3)-H(31)	109.6
C(30)-C(3)-H(31)	109.6
C(40)-C(4)-C(5)	110.70(17)
C(40)-C(4)-C(3)	113.20(17)
C(5)-C(4)-C(3)	103.62(17)
C(40)-C(4)-H(41)	109.7
C(5)-C(4)-H(41)	109.7
C(3)-C(4)-H(41)	109.7
O(1)-C(5)-C(4)	107.23(17)
O(1)-C(5)-H(51)	110.3
C(4)-C(5)-H(51)	110.3
O(1)-C(5)-H(52)	110.3
C(4)-C(5)-H(52)	110.3
H(51)-C(5)-H(52)	108.5
O(3)-C(30)-C(31)	112.56(17)
O(3)-C(30)-C(3)	106.42(16)
C(31)-C(30)-C(3)	112.91(17)
O(3)-C(30)-H(30)	108.3
C(31)-C(30)-H(30)	108.3
C(3)-C(30)-H(30)	108.3
C(30)-O(3)-H(3)	108.3(8)
C(36)-C(31)-C(32)	121.2(2)
C(36)-C(31)-C(30)	120.82(19)
C(32)-C(31)-C(30)	117.97(19)
C(33)-C(32)-C(31)	119.4(2)
C(33)-C(32)-H(32)	120.3
C(31)-C(32)-H(32)	120.3
O(33)-C(33)-C(32)	125.04(19)
O(33)-C(33)-C(34)	115.17(18)
C(32)-C(33)-C(34)	119.8(2)
O(34)-C(34)-C(35)	118.9(2)
O(34)-C(34)-C(33)	120.71(19)
C(35)-C(34)-C(33)	120.33(19)
O(35)-C(35)-C(34)	115.01(19)
O(35)-C(35)-C(36)	125.0(2)
C(34)-C(35)-C(36)	120.0(2)
C(31)-C(36)-C(35)	119.2(2)
C(31)-C(36)-H(36)	120.4
C(35)-C(36)-H(36)	120.4
C(33)-O(33)-C(37)	117.08(17)
O(33)-C(37)-H(371)	109.5
O(33)-C(37)-H(372)	109.5
H(371)-C(37)-H(372)	109.5
O(33)-C(37)-H(373)	109.5
H(371)-C(37)-H(373)	109.5
H(372)-C(37)-H(373)	109.5
C(34)-O(34)-C(38)	114.40(16)
O(34)-C(38)-H(381)	109.5
O(34)-C(38)-H(382)	109.5
H(381)-C(38)-H(382)	109.5
O(34)-C(38)-H(383)	109.5
H(381)-C(38)-H(383)	109.5
H(382)-C(38)-H(383)	109.5
C(35)-O(35)-C(39)	117.52(17)
O(35)-C(39)-H(391)	109.5
O(35)-C(39)-H(392)	109.5
H(391)-C(39)-H(392)	109.5
O(35)-C(39)-H(393)	109.5
H(391)-C(39)-H(393)	109.5
H(392)-C(39)-H(393)	109.5
O(4)-C(40)-C(41)	112.23(18)
O(4)-C(40)-C(4)	107.88(17)
C(41)-C(40)-C(4)	112.01(17)
O(4)-C(40)-H(40)	108.2

C(41)-C(40)-H(40)	108.2
C(4)-C(40)-H(40)	108.2
C(40)-O(4)-H(4)	106.8(10)
C(42)-C(41)-C(46)	118.8(2)
C(42)-C(41)-C(40)	120.11(19)
C(46)-C(41)-C(40)	121.1(2)
C(43)-C(42)-C(41)	121.1(2)
C(43)-C(42)-H(42)	119.5
C(41)-C(42)-H(42)	119.5
C(42)-C(43)-C(44)	118.7(2)
C(42)-C(43)-H(43)	120.7
C(44)-C(43)-H(43)	120.7
C(45)-C(44)-C(43)	121.8(2)
C(45)-C(44)-Cl(4)	119.34(18)
C(43)-C(44)-Cl(4)	118.90(19)
C(44)-C(45)-C(46)	118.7(2)
C(44)-C(45)-H(45)	120.7
C(46)-C(45)-H(45)	120.7
C(45)-C(46)-C(41)	121.0(2)
C(45)-C(46)-H(46)	119.5
C(41)-C(46)-H(46)	119.5
N(1)-C(11)-C(12)	179.2(4)
C(11)-C(12)-H(121)	109.5
C(11)-C(12)-H(122)	109.5
H(121)-C(12)-H(122)	109.5
C(11)-C(12)-H(123)	109.5
H(121)-C(12)-H(123)	109.5
H(122)-C(12)-H(123)	109.5

C(5)-O(1)-C(2)-O(2)	174.6(2)
C(5)-O(1)-C(2)-C(3)	-4.2(2)
O(2)-C(2)-C(3)-C(4)	174.2(2)
O(1)-C(2)-C(3)-C(4)	-7.1(2)
O(2)-C(2)-C(3)-C(30)	-62.8(3)
O(1)-C(2)-C(3)-C(30)	115.94(19)
C(2)-C(3)-C(4)-C(40)	-105.5(2)
C(30)-C(3)-C(4)-C(40)	135.91(19)
C(2)-C(3)-C(4)-C(5)	14.5(2)
C(30)-C(3)-C(4)-C(5)	-104.1(2)
C(2)-O(1)-C(5)-C(4)	13.9(2)
C(40)-C(4)-C(5)-O(1)	104.3(2)
C(3)-C(4)-C(5)-O(1)	-17.4(2)
C(2)-C(3)-C(30)-O(3)	-63.3(2)
C(4)-C(3)-C(30)-O(3)	53.2(2)
C(2)-C(3)-C(30)-C(31)	172.75(17)
C(4)-C(3)-C(30)-C(31)	-70.8(2)
O(3)-C(30)-C(31)-C(36)	-12.1(3)
C(3)-C(30)-C(31)-C(36)	108.4(2)
O(3)-C(30)-C(31)-C(32)	166.14(17)
C(3)-C(30)-C(31)-C(32)	-73.3(2)
C(36)-C(31)-C(32)-C(33)	0.2(3)
C(30)-C(31)-C(32)-C(33)	-178.09(19)
C(31)-C(32)-C(33)-O(33)	179.43(19)
C(31)-C(32)-C(33)-C(34)	1.5(3)
O(33)-C(33)-C(34)-O(34)	-3.6(3)
C(32)-C(33)-C(34)-O(34)	174.53(19)
O(33)-C(33)-C(34)-C(35)	179.37(19)
C(32)-C(33)-C(34)-C(35)	-2.5(3)
O(34)-C(34)-C(35)-O(35)	3.8(3)
C(33)-C(34)-C(35)-O(35)	-179.17(19)
O(34)-C(34)-C(35)-C(36)	-175.26(19)
C(33)-C(34)-C(35)-C(36)	1.8(3)
C(32)-C(31)-C(36)-C(35)	-0.9(3)
C(30)-C(31)-C(36)-C(35)	177.36(18)
O(35)-C(35)-C(36)-C(31)	-179.05(19)
C(34)-C(35)-C(36)-C(31)	-0.1(3)
C(32)-C(33)-O(33)-C(37)	11.9(3)
C(34)-C(33)-O(33)-C(37)	-170.05(19)
C(35)-C(34)-O(34)-C(38)	-104.9(2)
C(33)-C(34)-O(34)-C(38)	78.0(2)
C(34)-C(35)-O(35)-C(39)	176.7(2)
C(36)-C(35)-O(35)-C(39)	-4.3(3)
C(5)-C(4)-C(40)-O(4)	-55.0(2)
C(3)-C(4)-C(40)-O(4)	60.8(2)
C(5)-C(4)-C(40)-C(41)	-179.03(18)
C(3)-C(4)-C(40)-C(41)	-63.2(2)
O(4)-C(40)-C(41)-C(42)	164.80(18)
C(4)-C(40)-C(41)-C(42)	-73.7(2)
O(4)-C(40)-C(41)-C(46)	-13.9(3)
C(4)-C(40)-C(41)-C(46)	107.6(2)
C(46)-C(41)-C(42)-C(43)	2.0(3)
C(40)-C(41)-C(42)-C(43)	-176.7(2)
C(41)-C(42)-C(43)-C(44)	-0.4(3)
C(42)-C(43)-C(44)-C(45)	-1.2(3)
C(42)-C(43)-C(44)-Cl(4)	178.41(18)
C(43)-C(44)-C(45)-C(46)	1.3(3)
Cl(4)-C(44)-C(45)-C(46)	-178.33(16)
C(44)-C(45)-C(46)-C(41)	0.3(3)
C(42)-C(41)-C(46)-C(45)	-1.9(3)
C(40)-C(41)-C(46)-C(45)	176.81(19)

References

[1] a) W. J. Gensler, C. M. Samour, S. Y. Wang, F. Johnson, *J. Am. Chem. Soc.* **1960**, *82*, 1714-1727; b) W. J. Gensler, C. D. Gatsonis, *J. Org. Chem.* **1966**, *31*, 4004-4008.
[2] J. Van der Eycken, P. De Clercq, M. Vandewalle, *Tetrahedron* **1986**, *42*, 4297-4308.
[3] T. Kaneko, H. Wong, *Tetrahedron Lett.* **1987**, *28*, 517-520.
[4] D. I. Macdonald, T. Durst, *J. Org. Chem.* **1988**, *53*, 3663-3669.
[5] R. C. Andrews, S. J. Teague, A. I. Meyers, *J. Am. Chem. Soc.* **1988**, *110*, 7854-7858.
[6] D. W. Jones, A. M. Thompson, *J. Chem. Soc., Chem. Commun.* **1989**, 1370-1371.
[7] R. Van Speybroeck, H. Guo, J. Van der Eycken, M. Vandewalle, *Tetrahedron* **1991**, *47*, 4675-4682.
[8] E. J. Bush, D. W. Jones, *J. Chem. Soc., Chem. Commun.* **1993**, 1200-1201.
[9] M. Medarde, A. C. Ramos, E. Caballero, J. Luis López, R. Peláez-Lamamiéde Clairac, A. San Feliciano, *Tetrahedron Lett.* **1996**, *37*, 2663-2666.
[10] S. B. Hadimani, R. P. Tanpure, S. V. Bhat, *Tetrahedron Lett.* **1996**, *37*, 4791-4794.
[11] D. B. Berkowitz, S. Choi, J.-H. Maeng, *J. Org. Chem.* **2000**, *65*, 847-860.
[12] I. Fumito, T. Eiji, *Bull. Chem. Soc. Jpn.* **1988**, *61*, 4361-4366.
[13] U. Engelhardt, A. Sarkar, T. Linker, *Angew. Chem. Int. Ed.* **2003**, *42*, 2487-2489.
[14] Y. Wu, H. Zhang, Y. Zhao, J. Zhao, J. Chen, L. Li, *Org. Lett.* **2007**, *9*, 1199-1202.
[15] D. Stadler, T. Bach, *Angew. Chem. Int. Ed.* **2008**, *47*, 7557-7559.
[16] Y. Wu, J. Zhao, J. Chen, C. Pan, L. Li, H. Zhang, *Org. Lett.* **2009**, *11*, 597-600.
[17] C. P. Ting, T. J. Maimone, *Angew. Chem. Int. Ed.* **2014**, *53*, 3115-3119.
[18] K. Lisiecki, K. K. Krawczyk, P. Roszkowski, J. K. Maurin, Z. Czarnocki, *Org. Biomol. Chem.* **2016**, *14*, 460-469.
[19] S. Hajra, S. Garai, S. Hazra, *Org. Lett.* **2017**, *19*, 6530-6533.
[20] J. Xiao, X.-W. Cong, G.-Z. Yang, Y.-W. Wang, Y. Peng, *Org. Lett.* **2018**, *20*, 1651-1654.
[21] S. Martinez, R. P. Hausinger, *J. Biol. Chem.* **2015**, *290*, 20702-20711.
[22] a) L. C. Blasiak, F. H. Vaillancourt, C. T. Walsh, C. L. Drennan, *Nature* **2006**, *440*, 368; b) S. Pandian, M. A. Vincent, I. H. Hillier, N. A. Burton, *Dalton Trans.* **2009**, 6201-6207.
[23] M. Fuchs, M. Schober, A. Orthaber, K. Faber, *Adv. Synth. Catal.* **2013**, *355*, 2499-2505.
[24] G. M. Sheldrick, *Acta Cryst.* **2008**, *A64*, 112-122.
[25] G. M. Sheldruck, *Acta Cryst.* **2015**, *C71*, 3-8.
[26] C. K. Johnson, in *ONRL Report #3794*, Oak Ridge National Laboratory, Tennessee, USA, **1965**.

NMR-Data

rac-4-[Benzo[d][1,3]dioxol-5-yl(hydroxy)methyl]-3-methylenedihydrofuran-2(3_H_)-one (8).

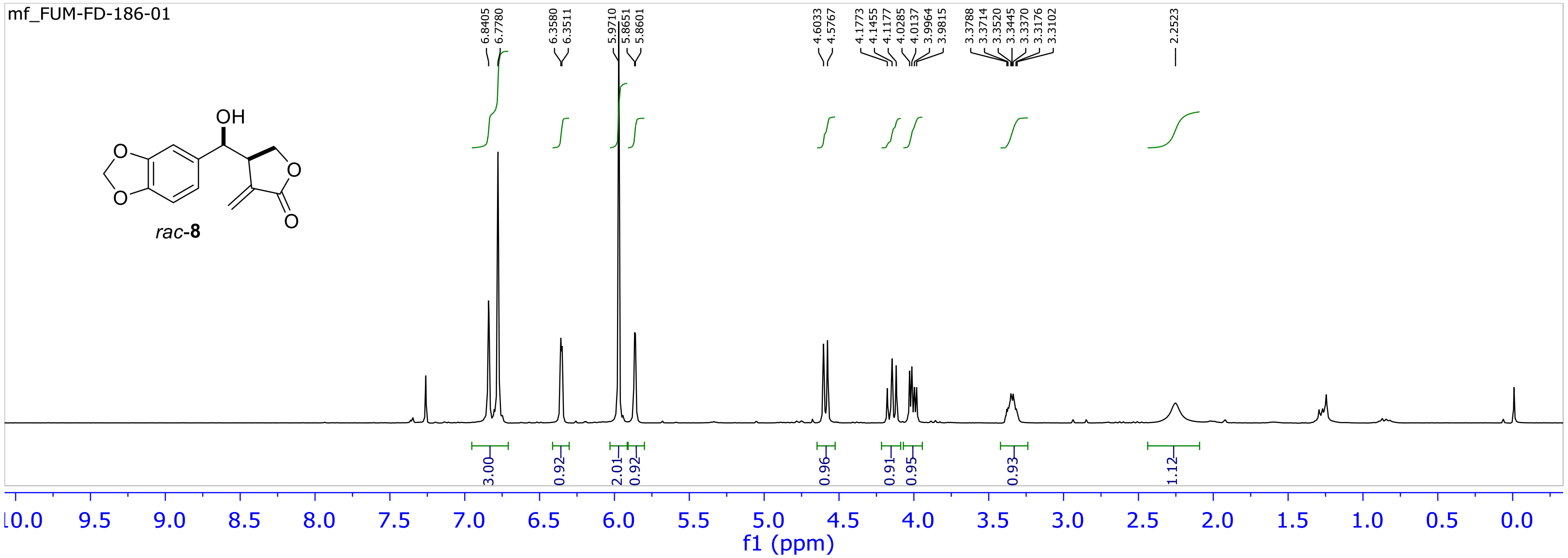

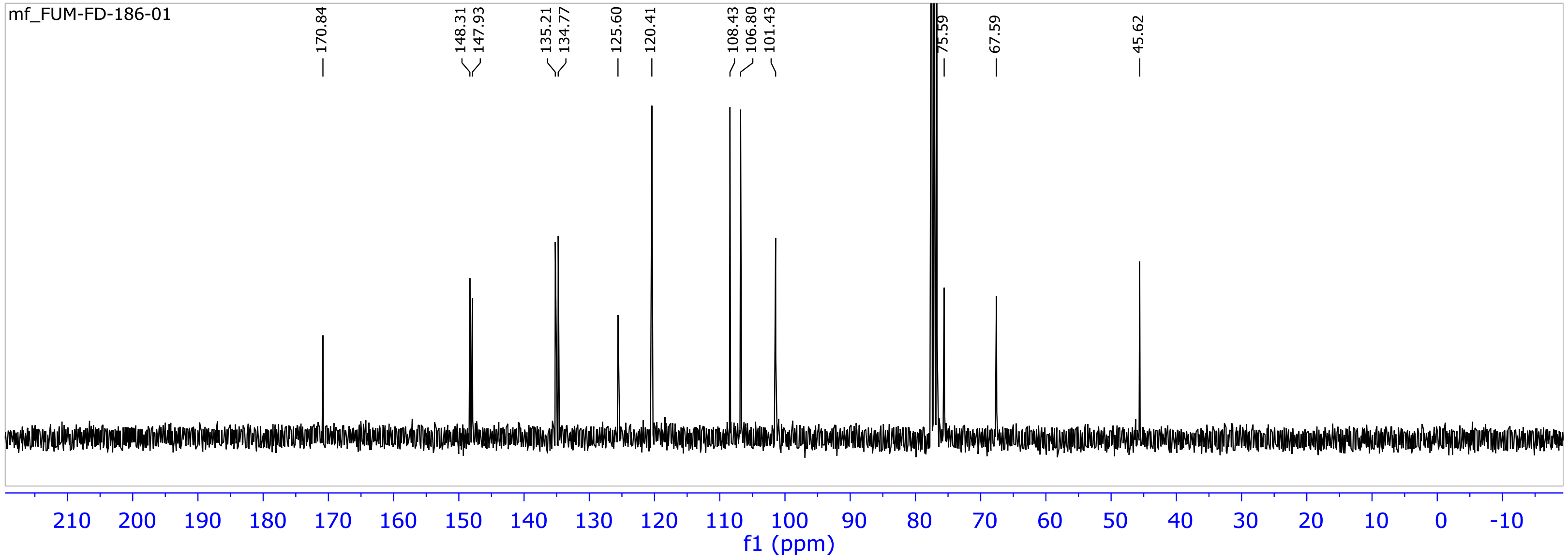

rac-4-[(3,4-dimethoxyphenyl)(hydroxy)methyl]-3-methylenedihydrofuran-2(3H)-one (SI-12).

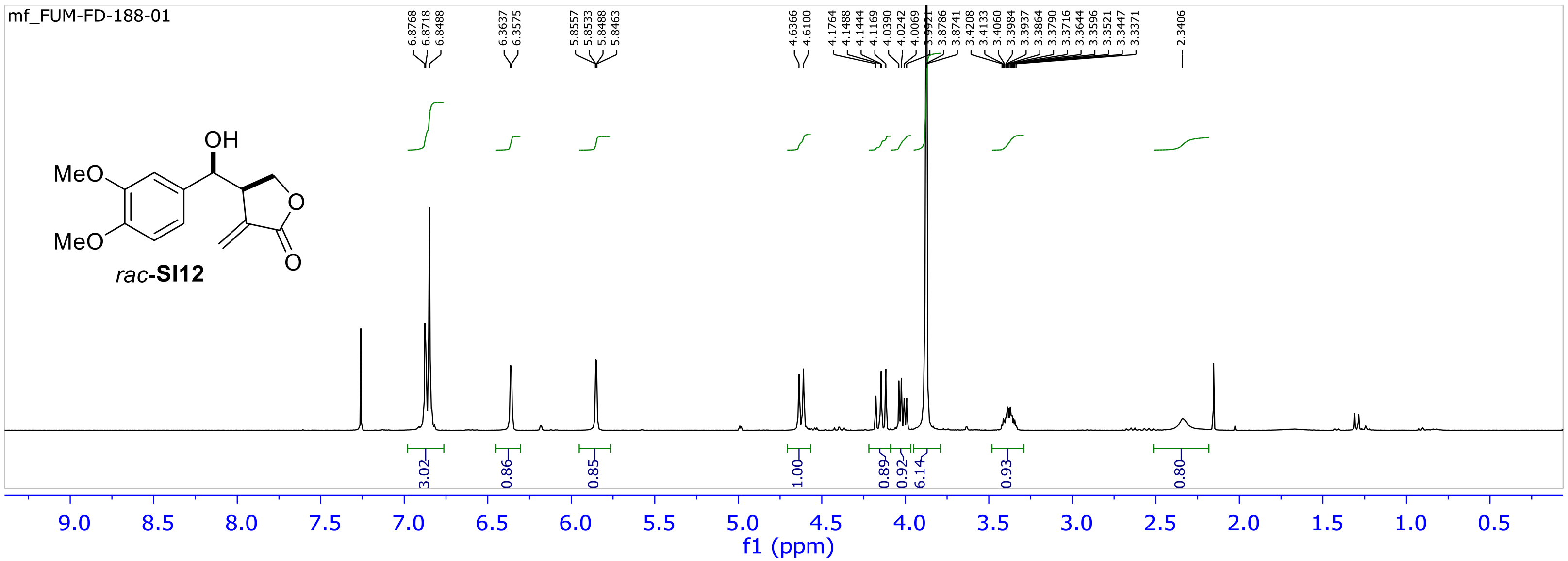

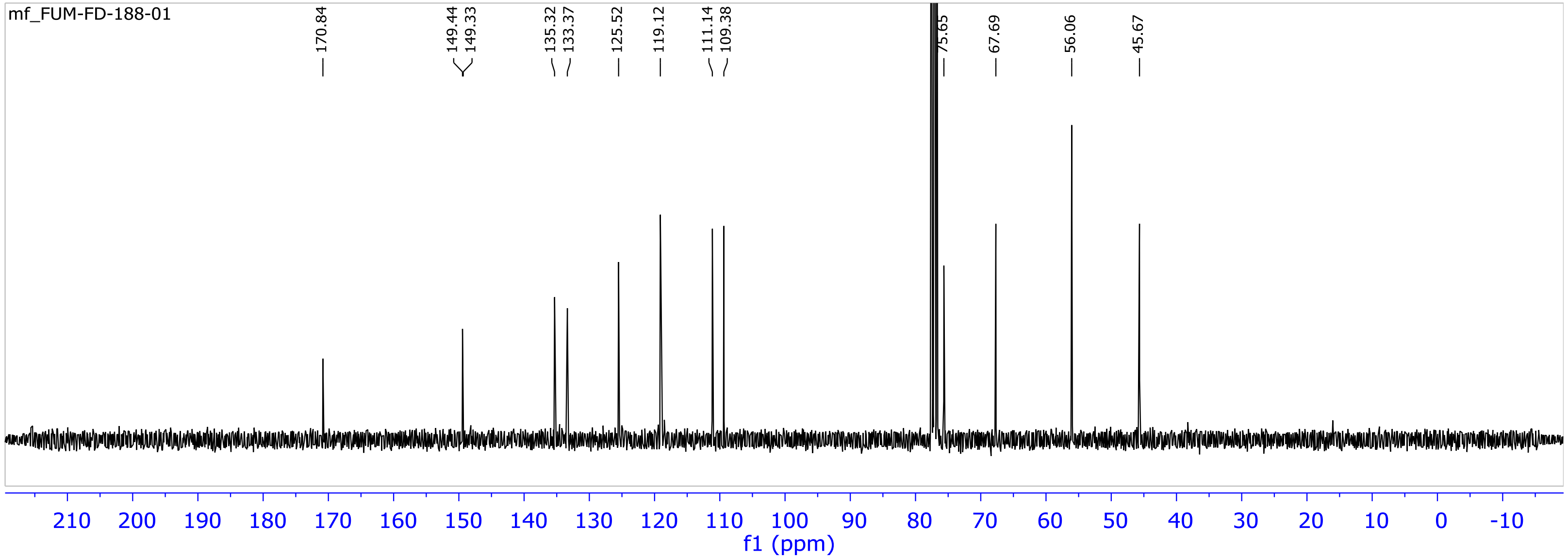

***rac*-4-[hydroxy(phenyl)methyl]-3-methylenedihydrofuran-2(3*H*)-one (SI-13).**

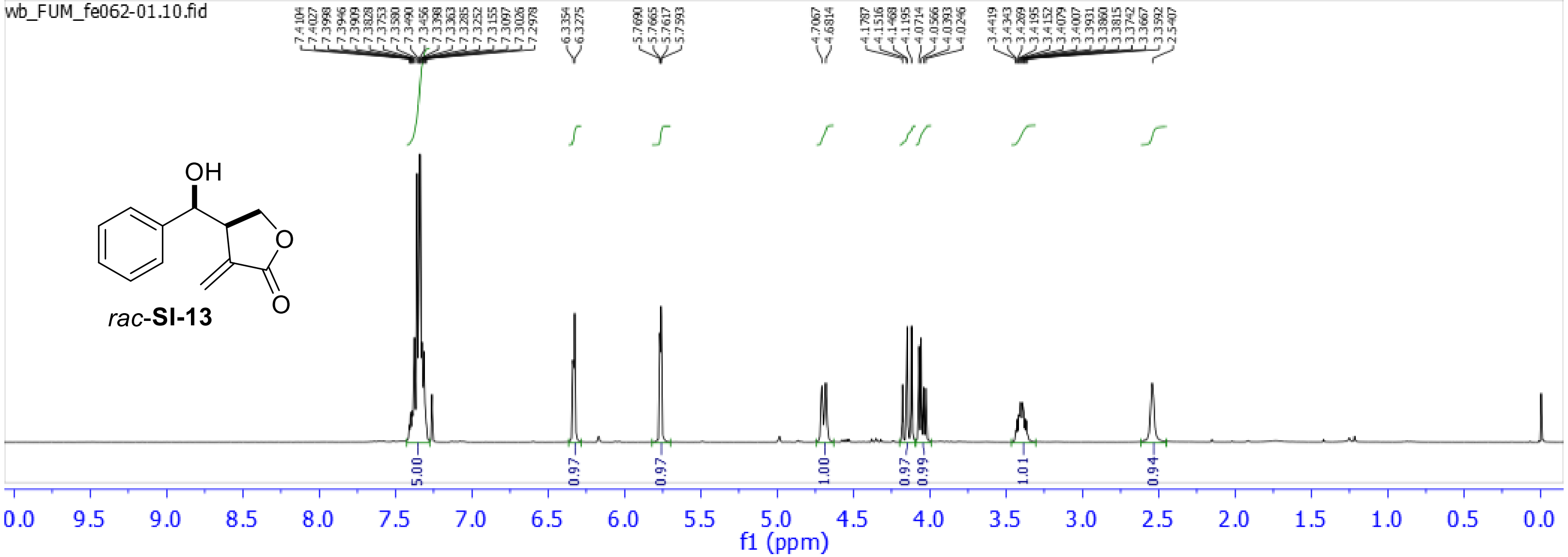

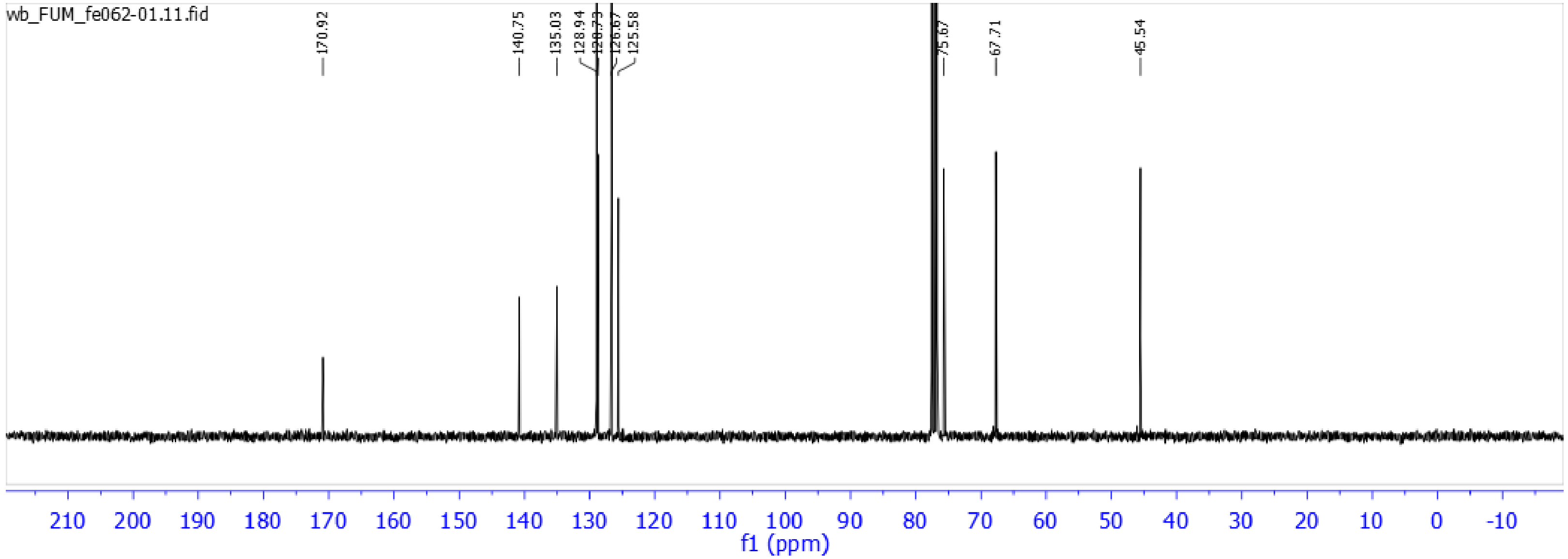

rac-4-[hydroxy(naphthalen-2-yl)methyl]-3-methylenedihydrofuran-2(3_H_)-one (SI-14).

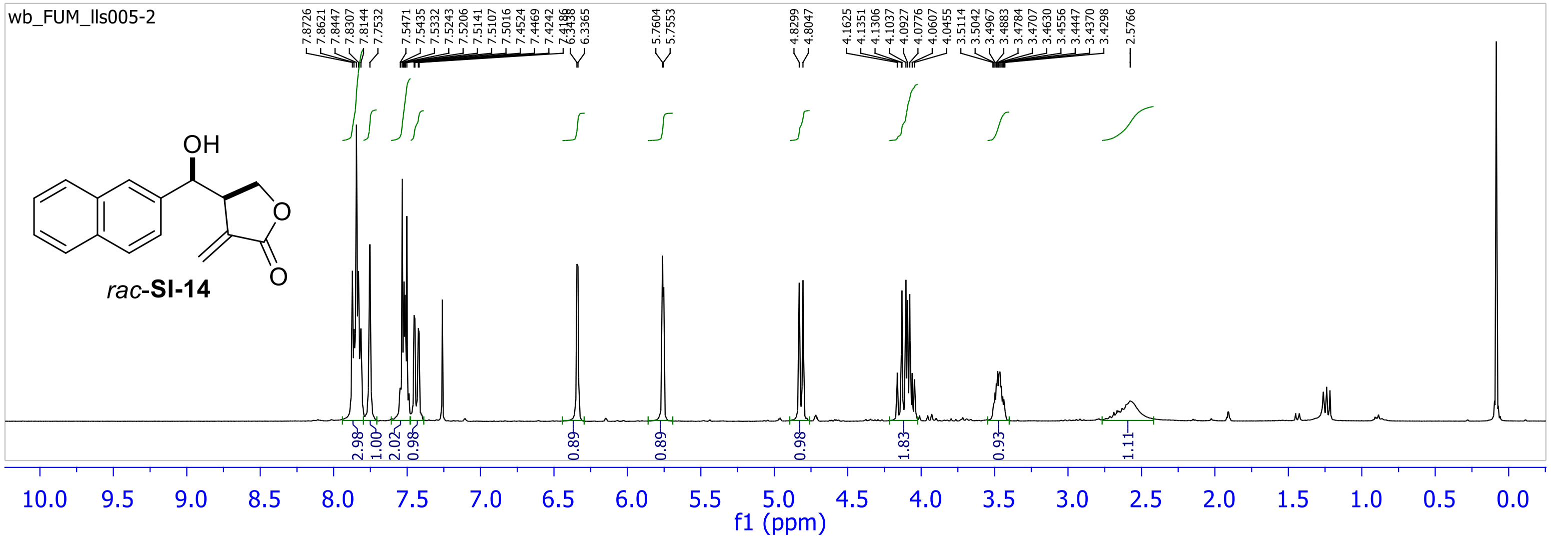

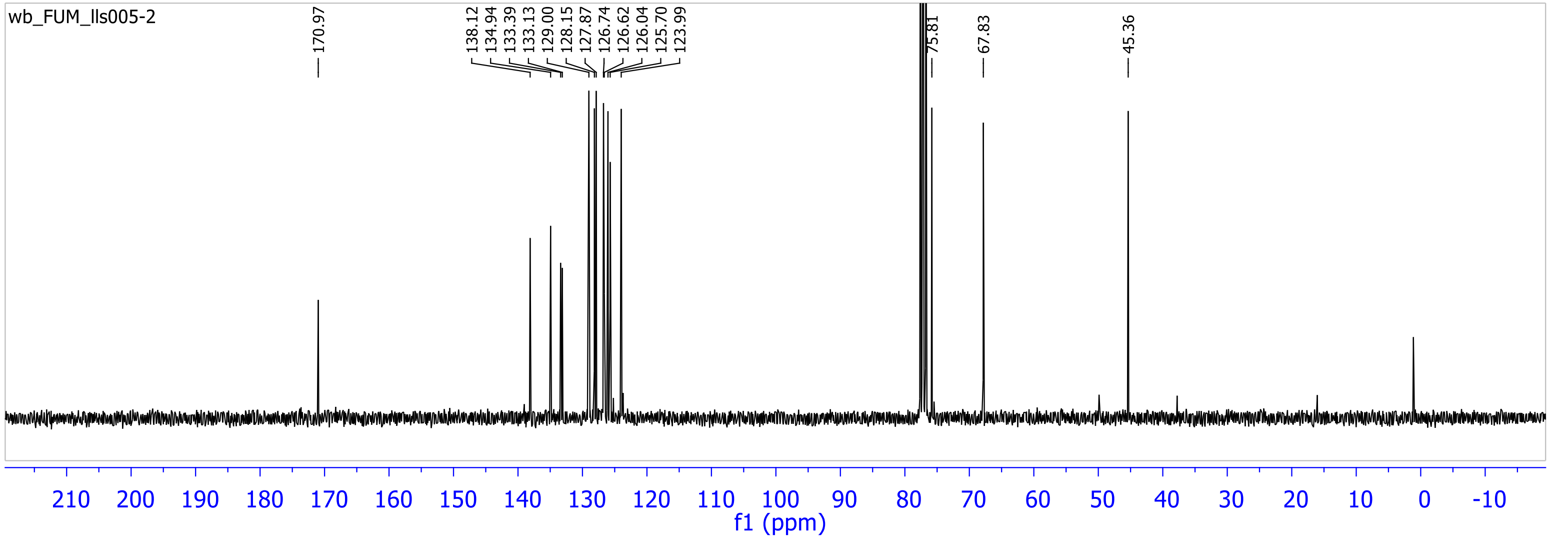

***rac*-4-[(3-chlorophenyl)(hydroxy)methyl]-3-methylenedihydrofuran-2(3*H*)-one (SI-15).**

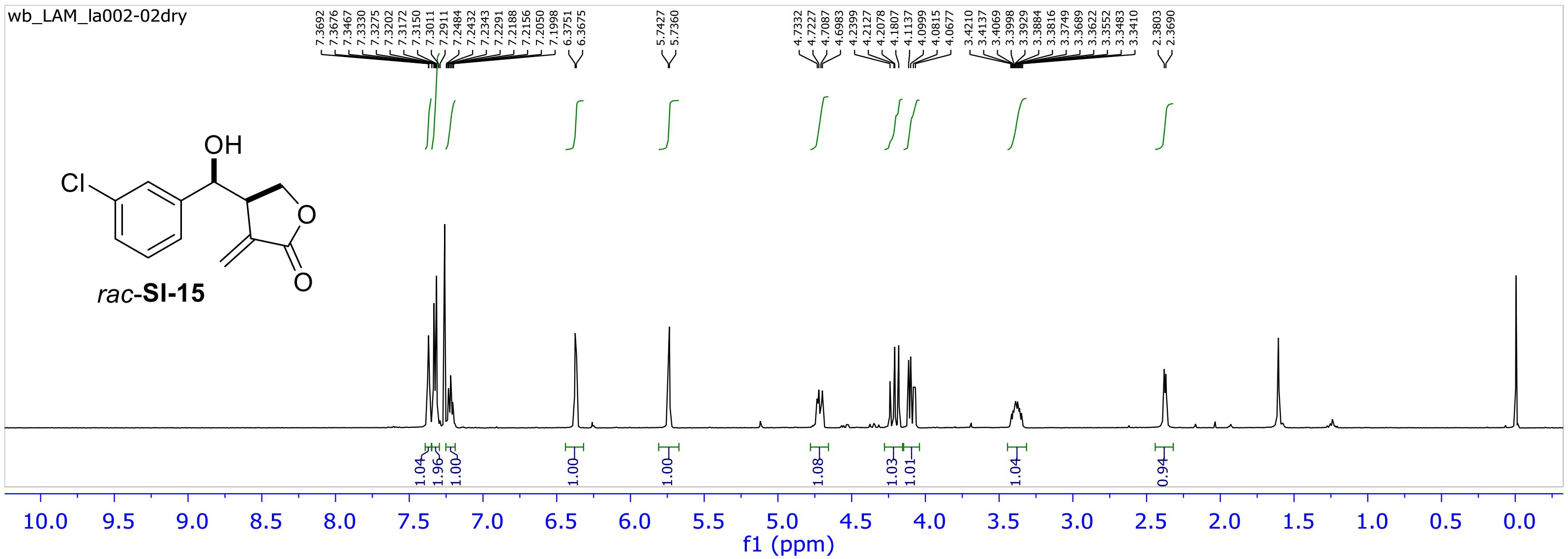

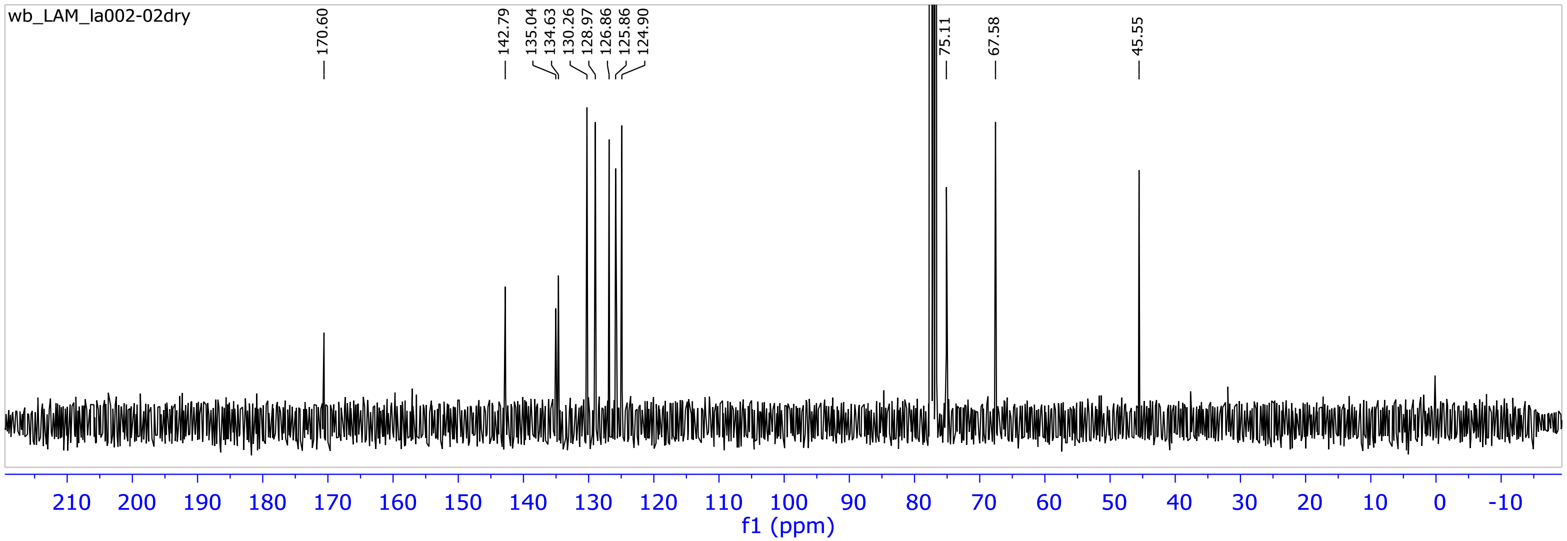

***rac*-4-[(4-chlorophenyl)(hydroxy)methyl]-3-methylenedihydrofuran-2(3*H*)-one (SI-16).**

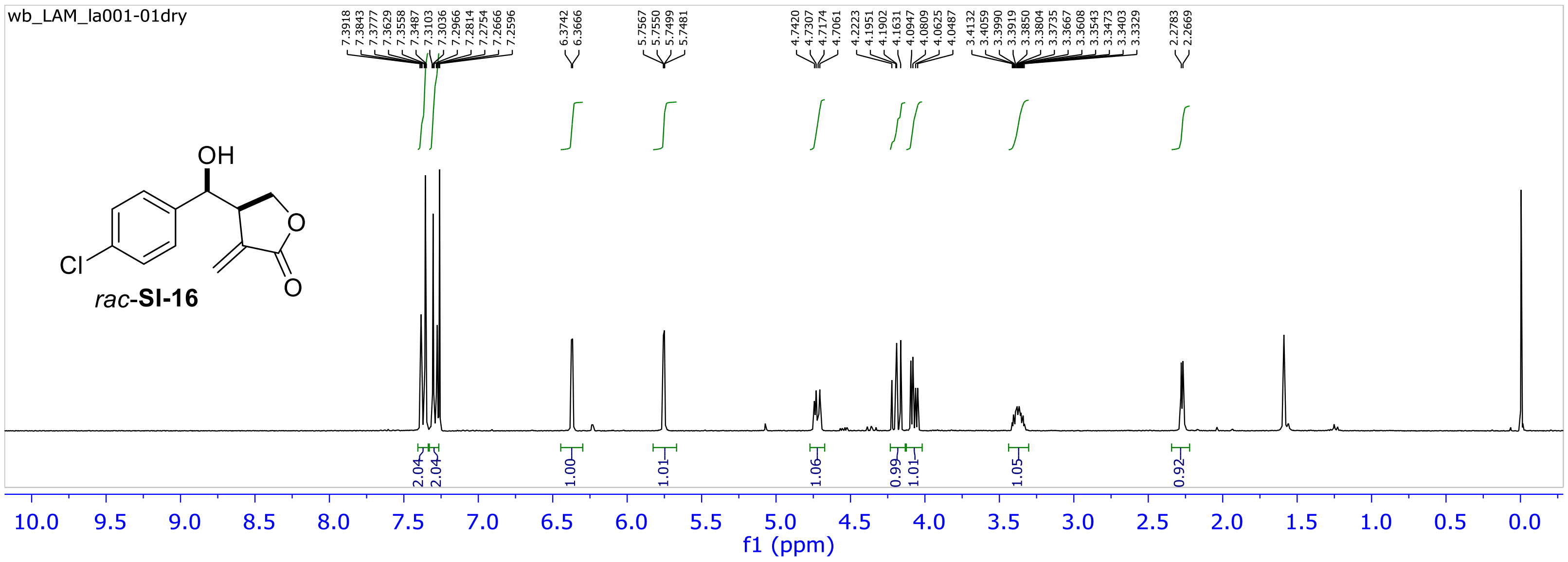

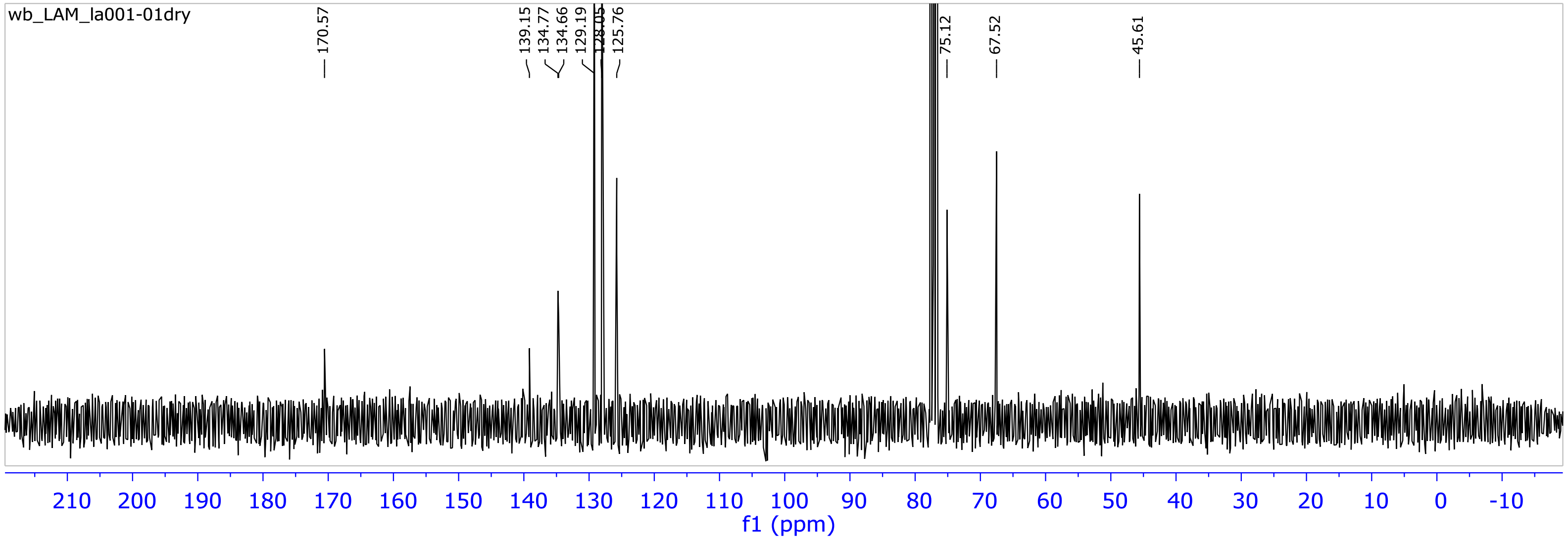

***rac*-4-[(3-Iodophenyl)(hydroxy)methyl]-3-methylenedihydrofuran-2(3H)-one (SI-17).**

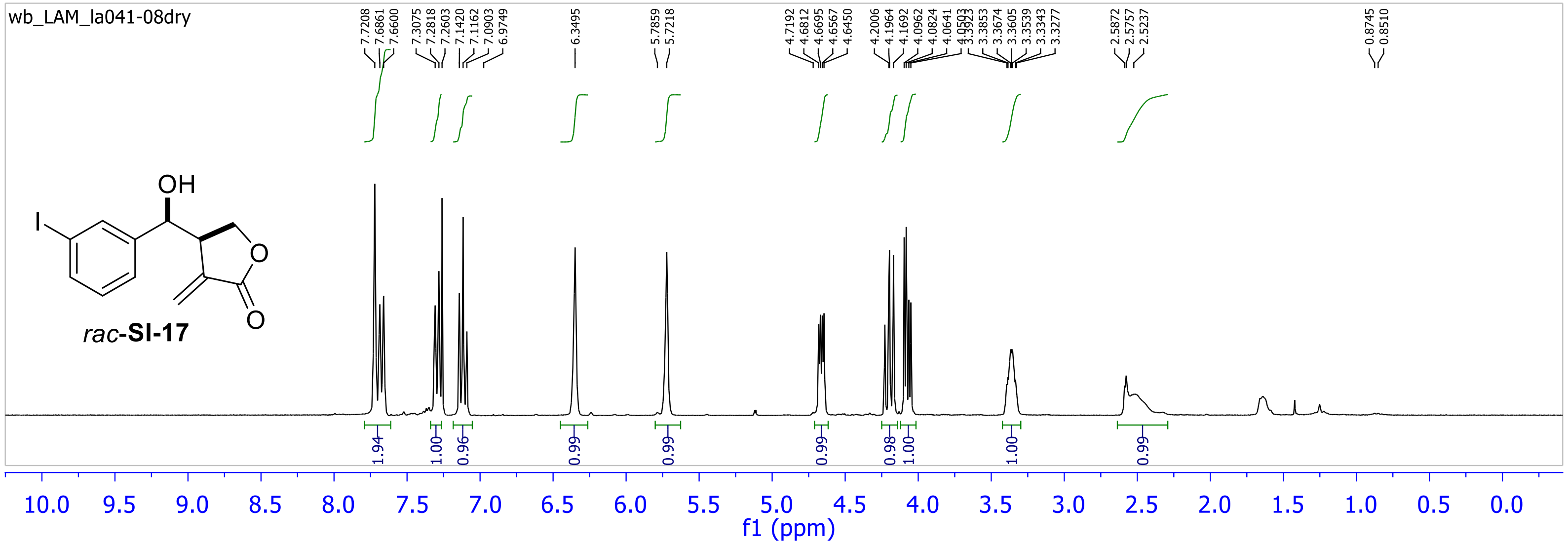

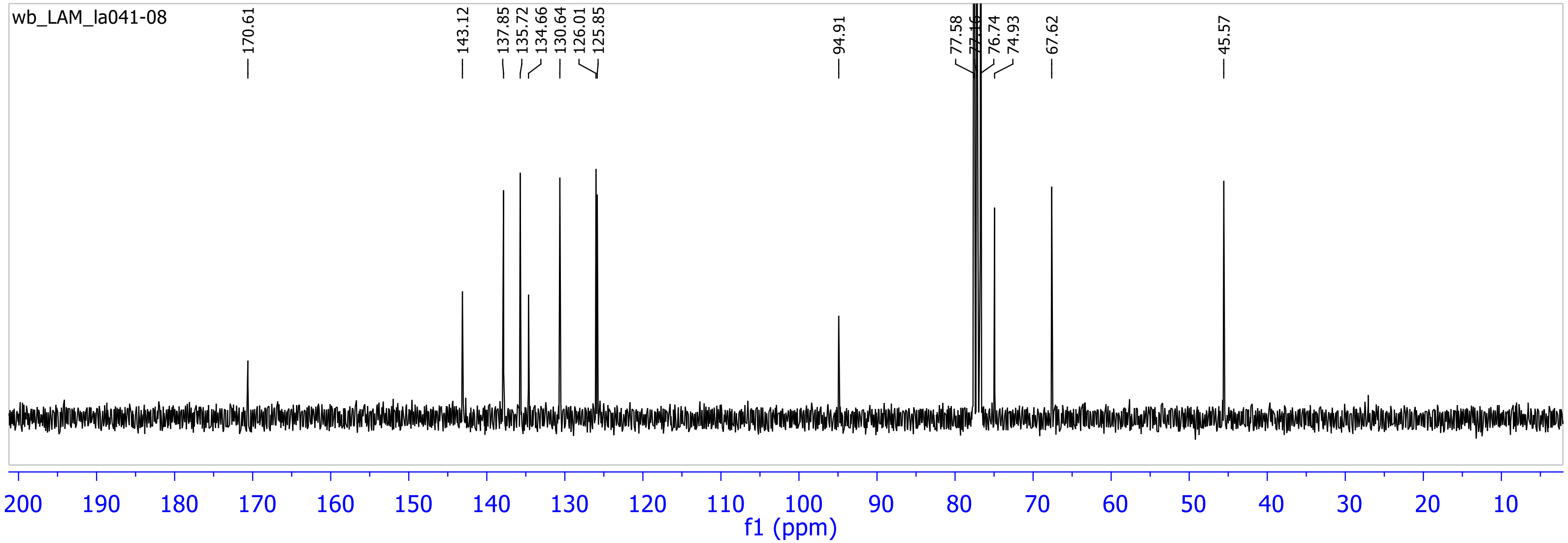

***rac*-Methyl 4-[hydroxy(4-methylene-5-oxotetrahydrofuran-3-yl)methyl]benzoate (SI-18).**

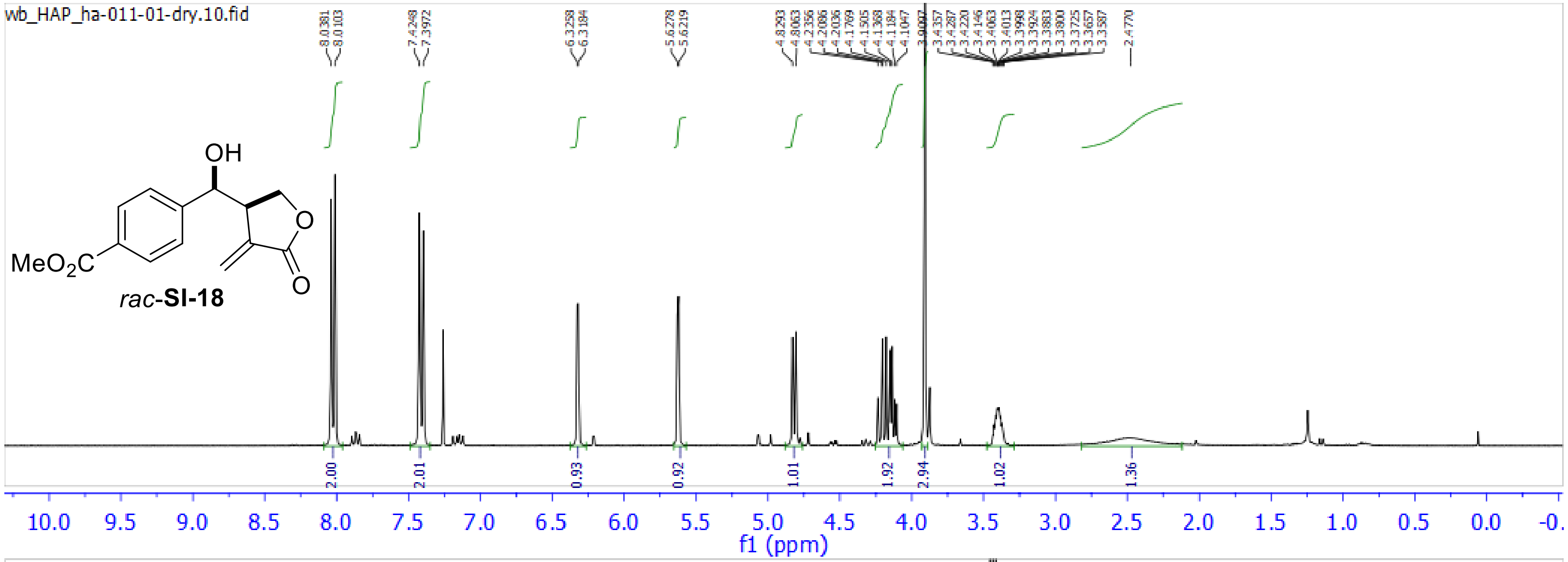

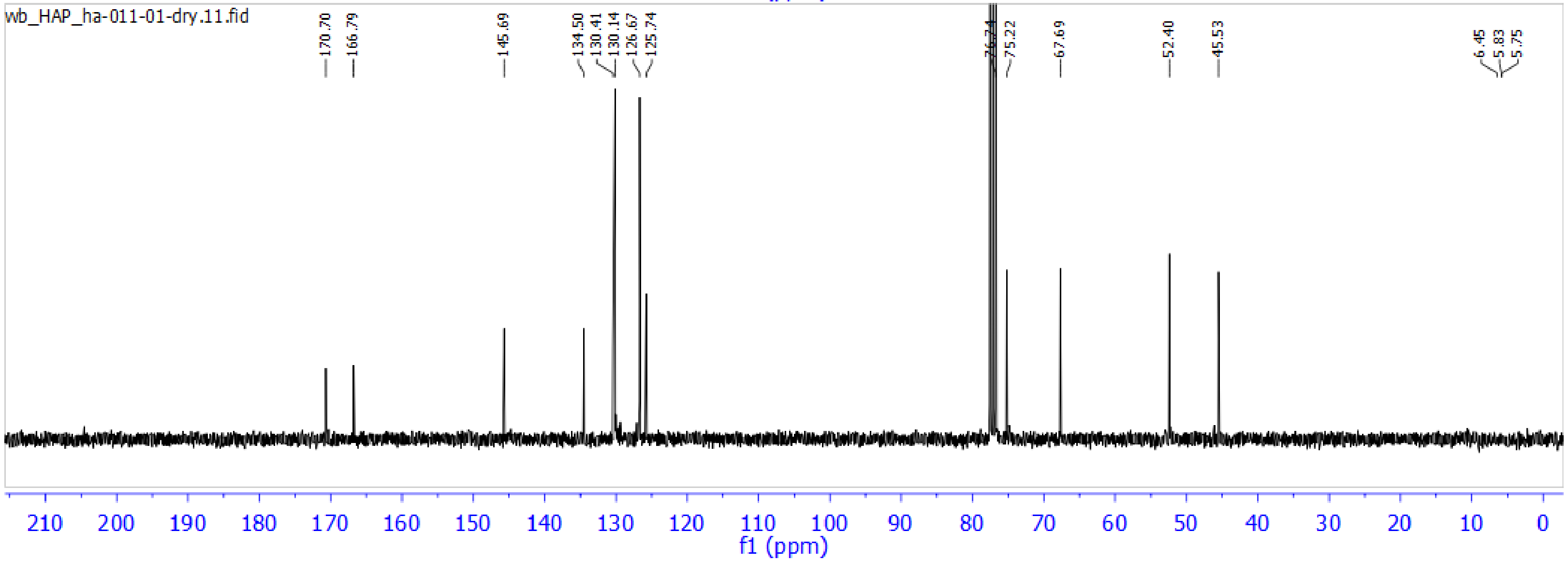

***rac*-4-[hydroxy(4-methoxyphenyl)methyl]-3-methylenedihydrofuran-2(3H)-one (SI-19).**

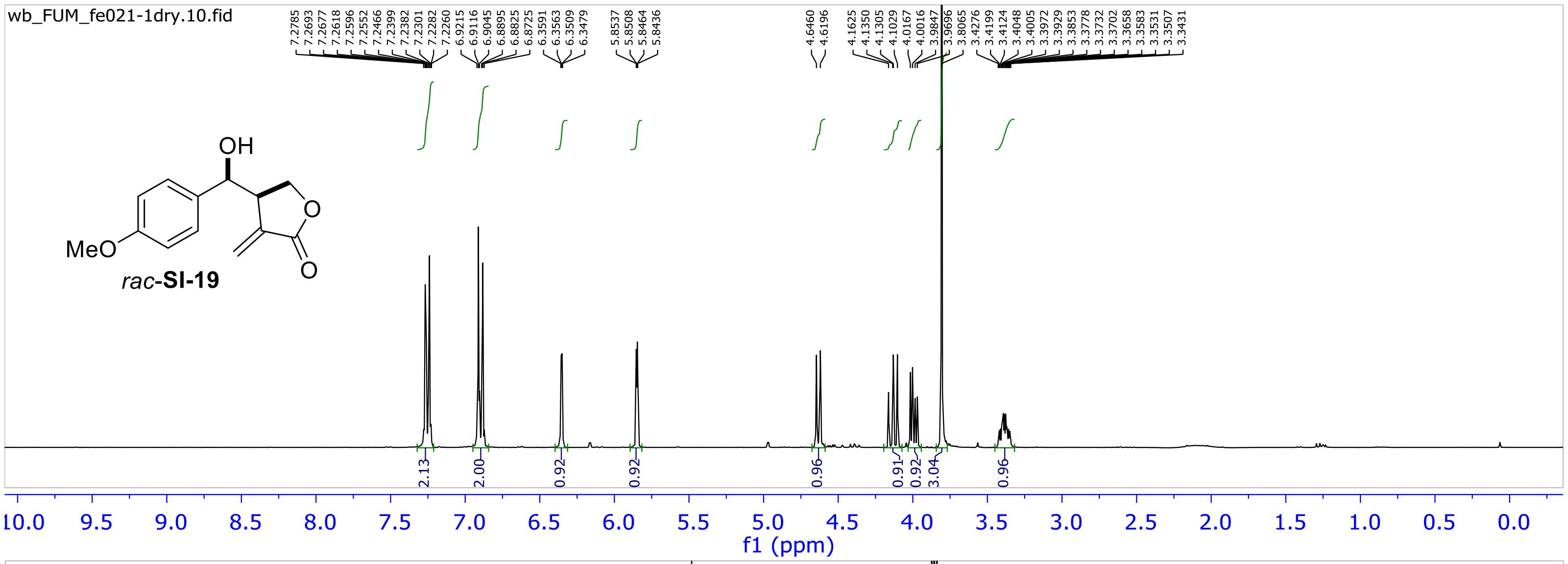

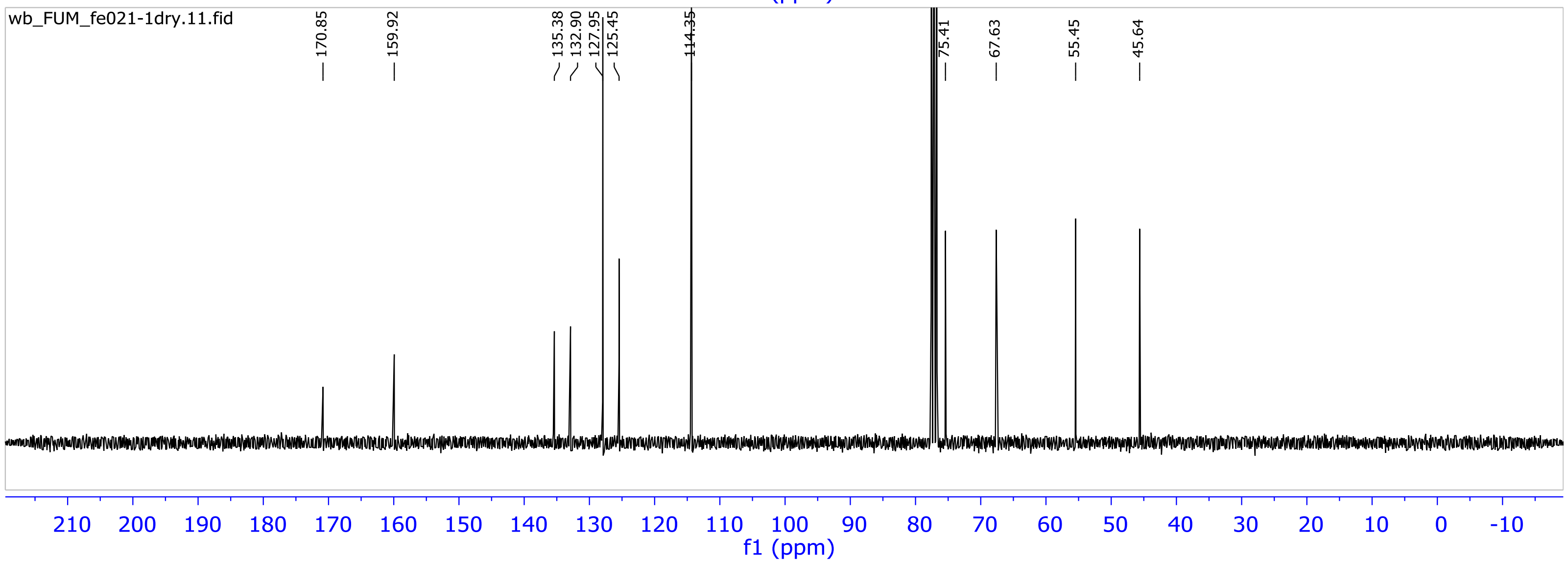

rac-4-[hydroxy(3-methoxyphenyl)methyl]-3-methylenedihydrofuran-2(3H)-one (SI-20).

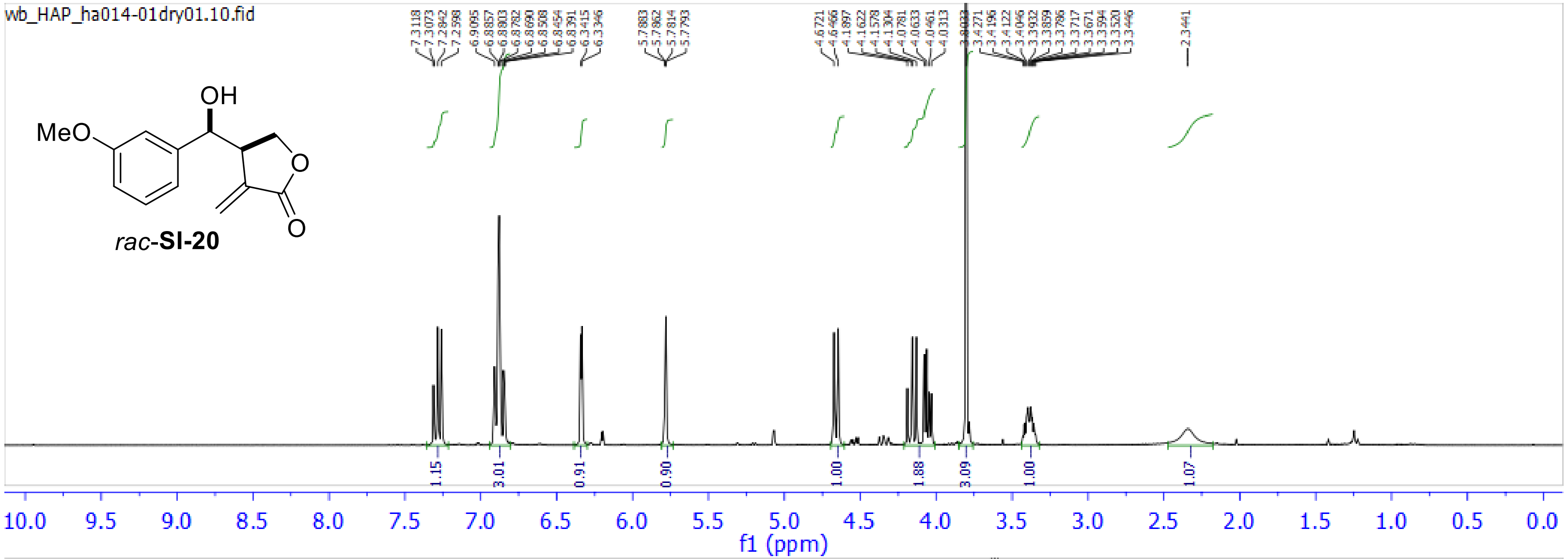

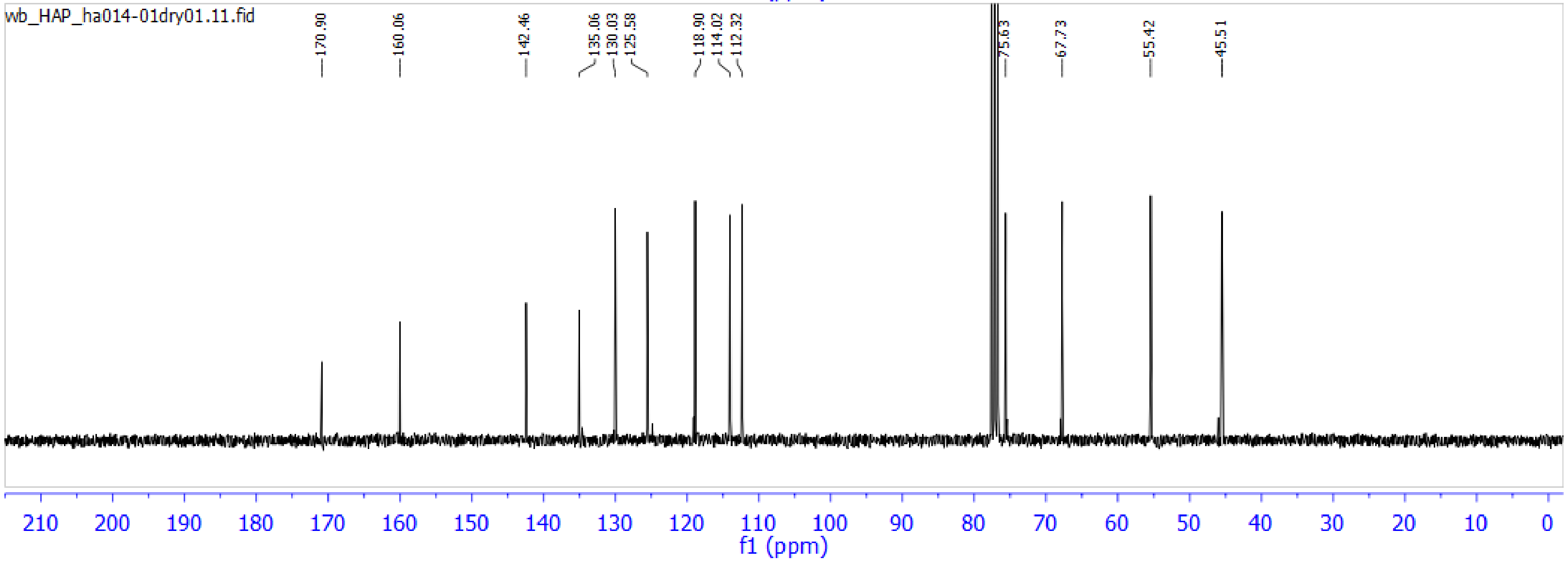

***rac*-4-[(4-fluorophenyl)(hydroxy)methyl]-3-methylenedihydrofuran-2(3H)-one (SI-21).**

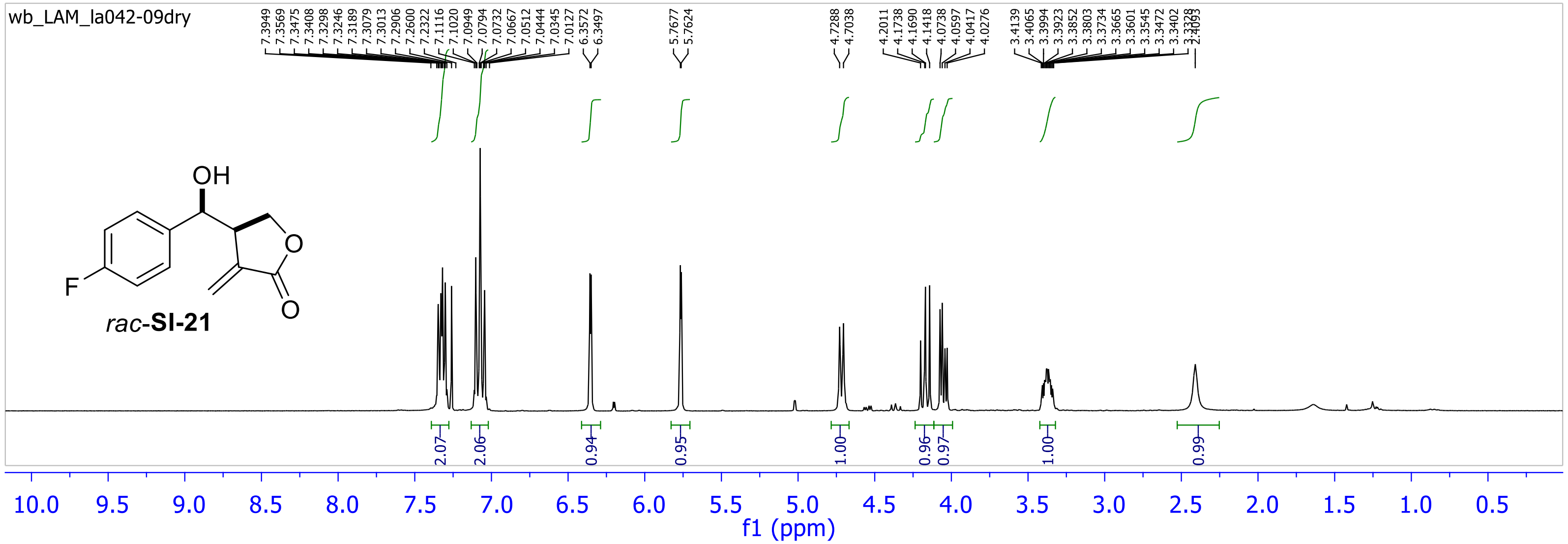

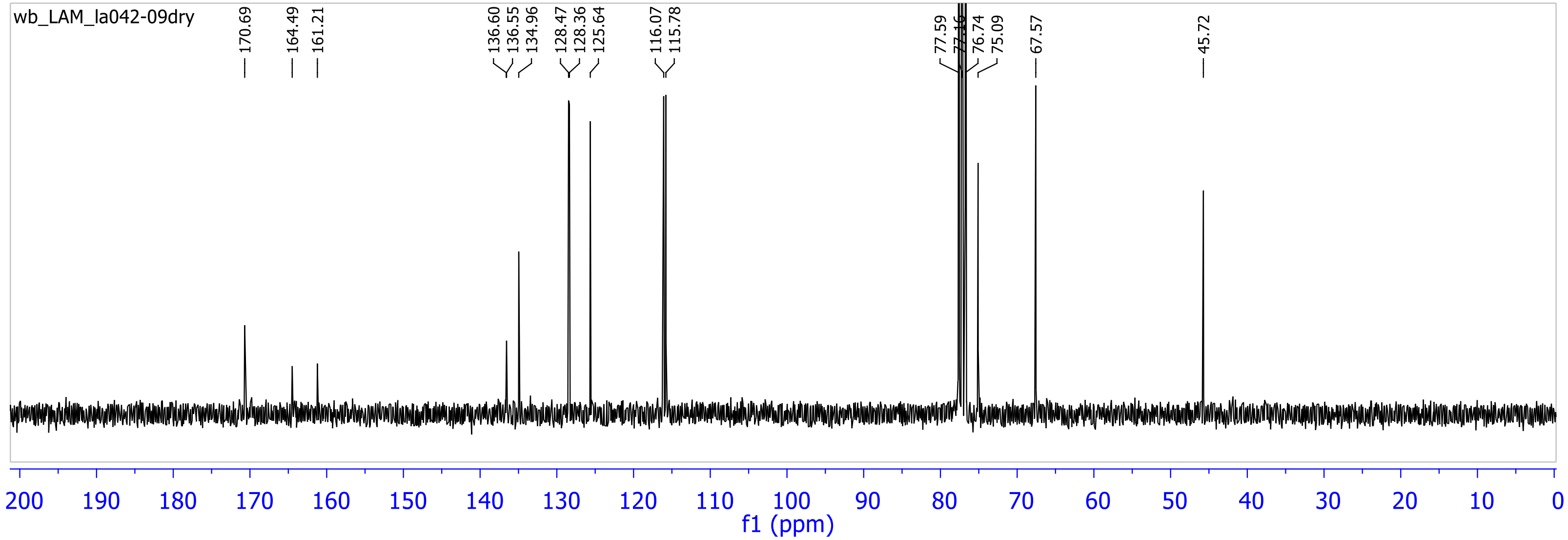

rac-4-[(2-chlorophenyl)(hydroxy)methyl]-3-methylenedihydrofuran-2(3_H_)-one (SI-22).

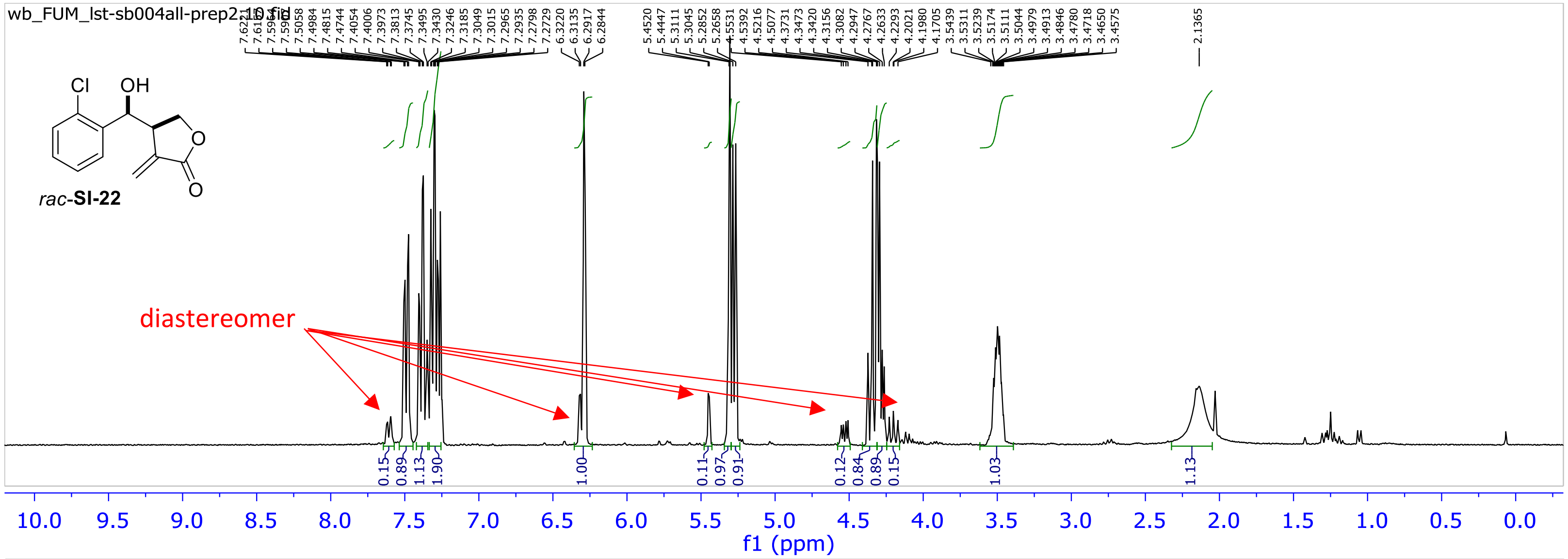

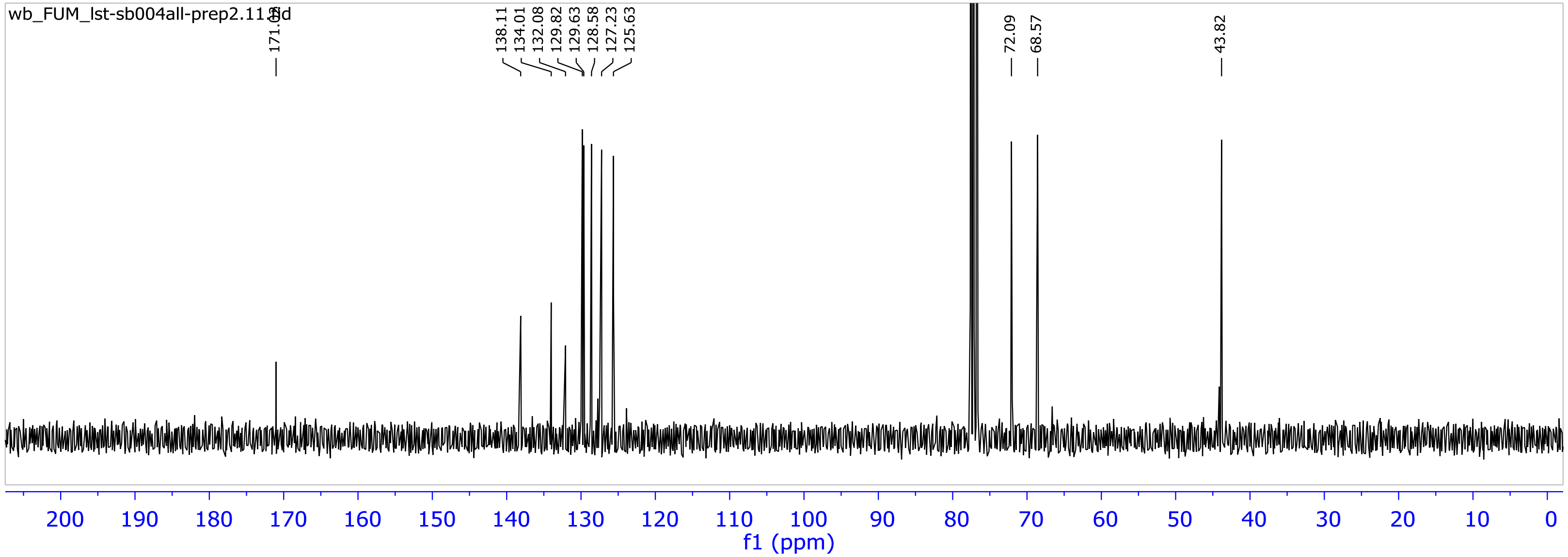

rac-4-[furan-2-yl(hydroxy)methyl]-3-methylenedihydrofuran-2(3*H*)-one (SI-23).

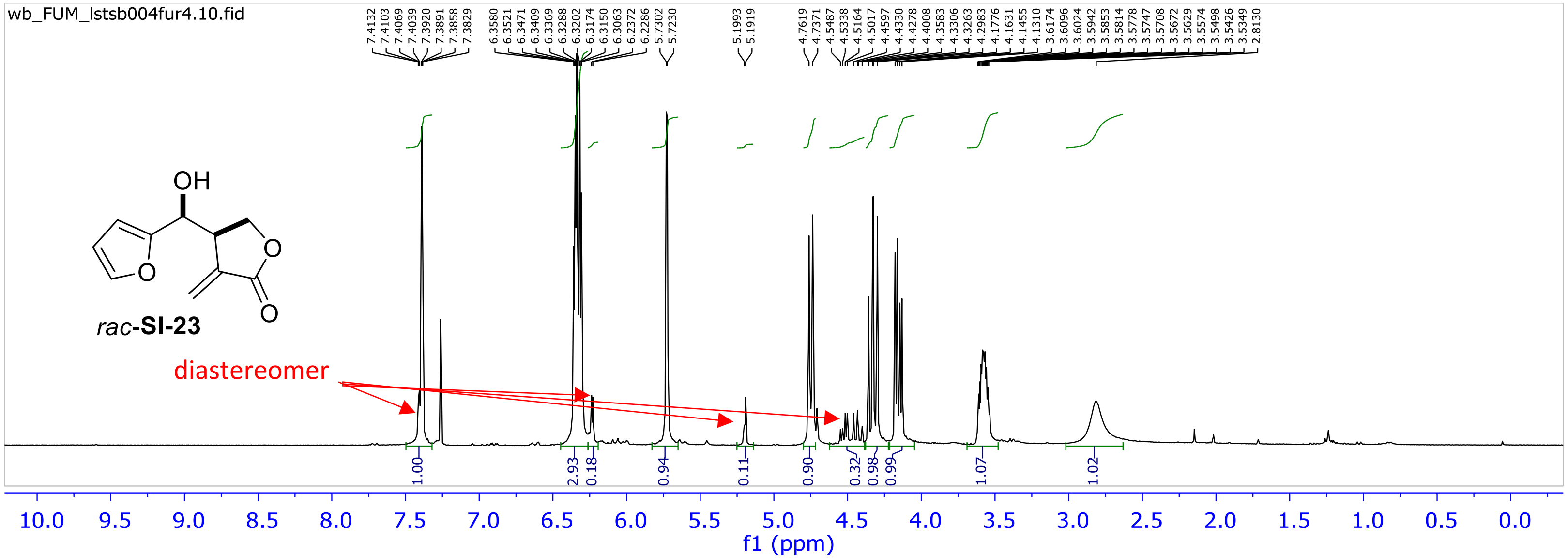

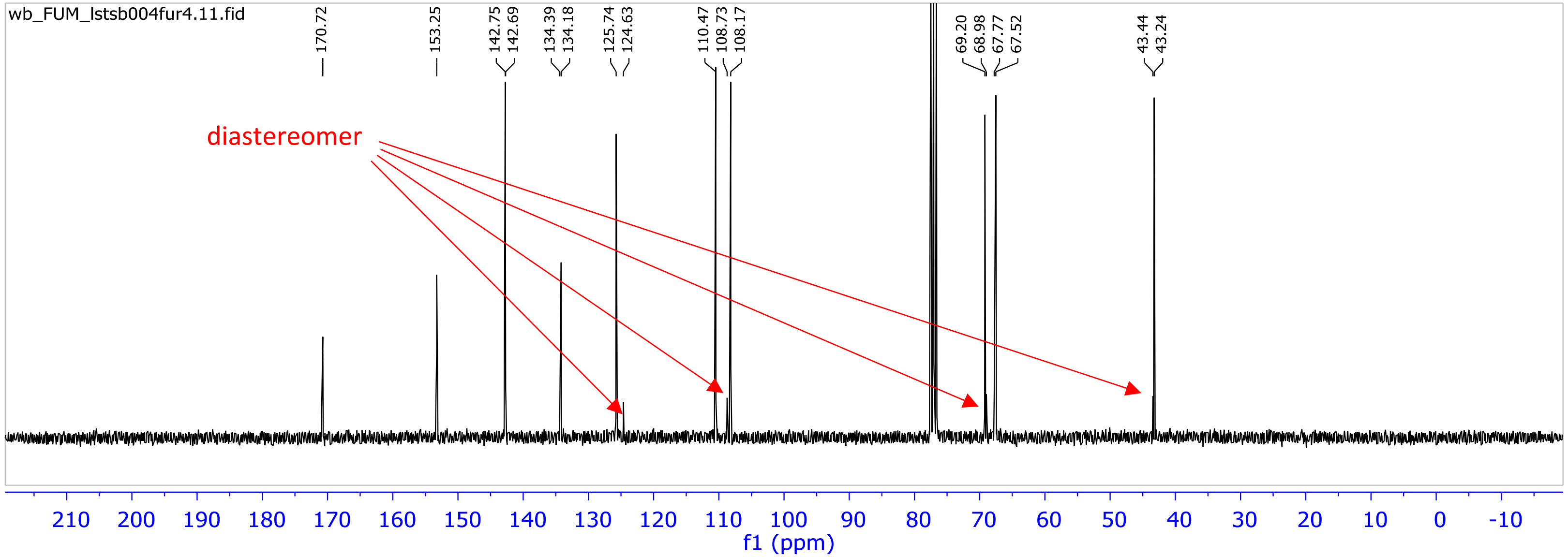

***rac*-4-[Hydroxy(4-hydroxyphenyl)methyl]-3-methylenedihydrofuran-2(3*H*)-one (SI-25).**

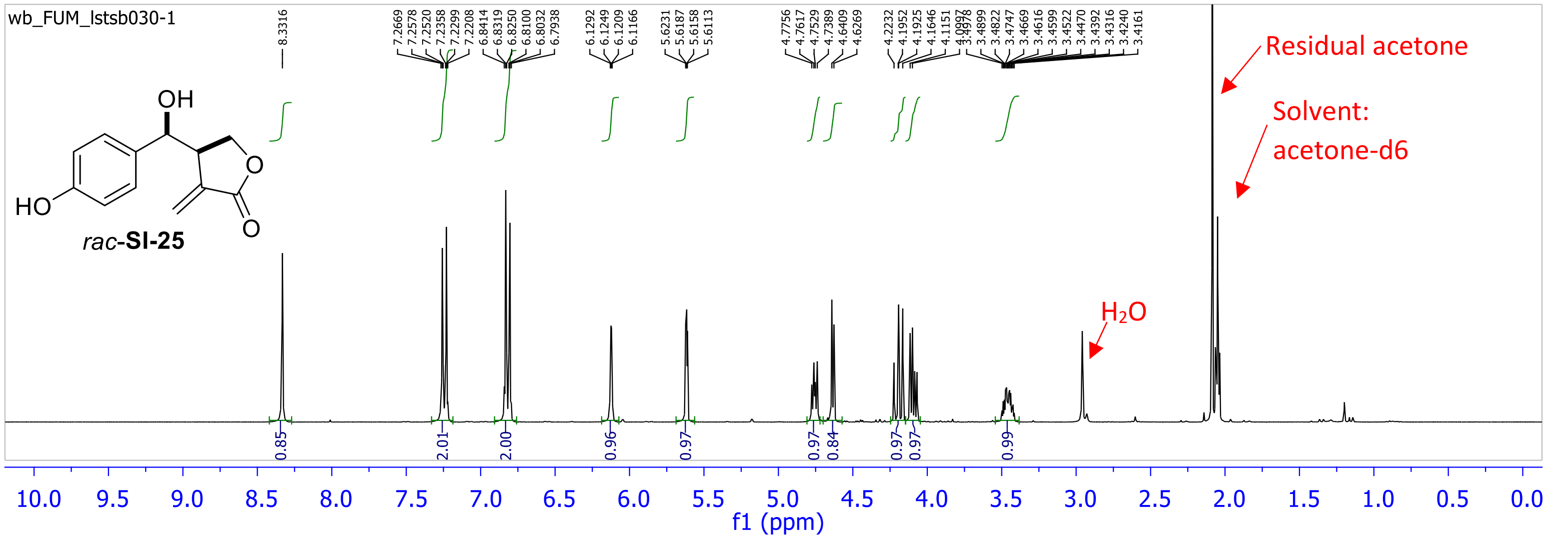

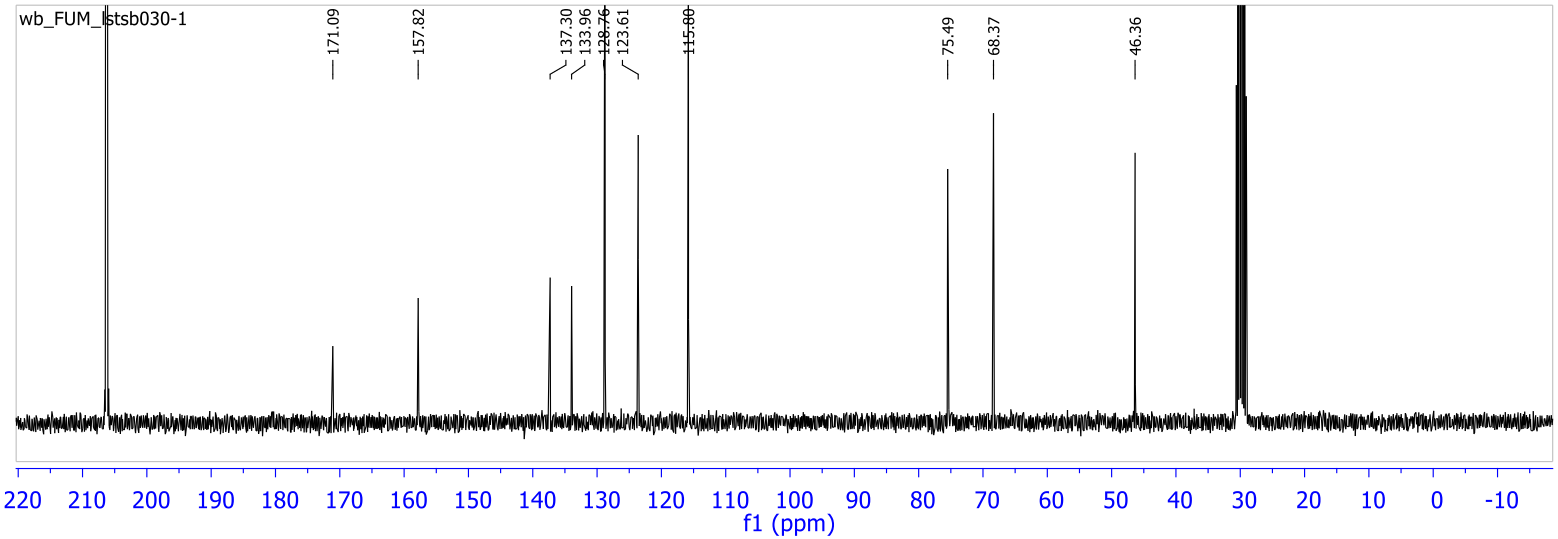

***rac*-4-[(4-Iodophenyl)(hydroxy)methyl]-3-methylenedihydrofuran-2(3*H*)-one (SI-26).**

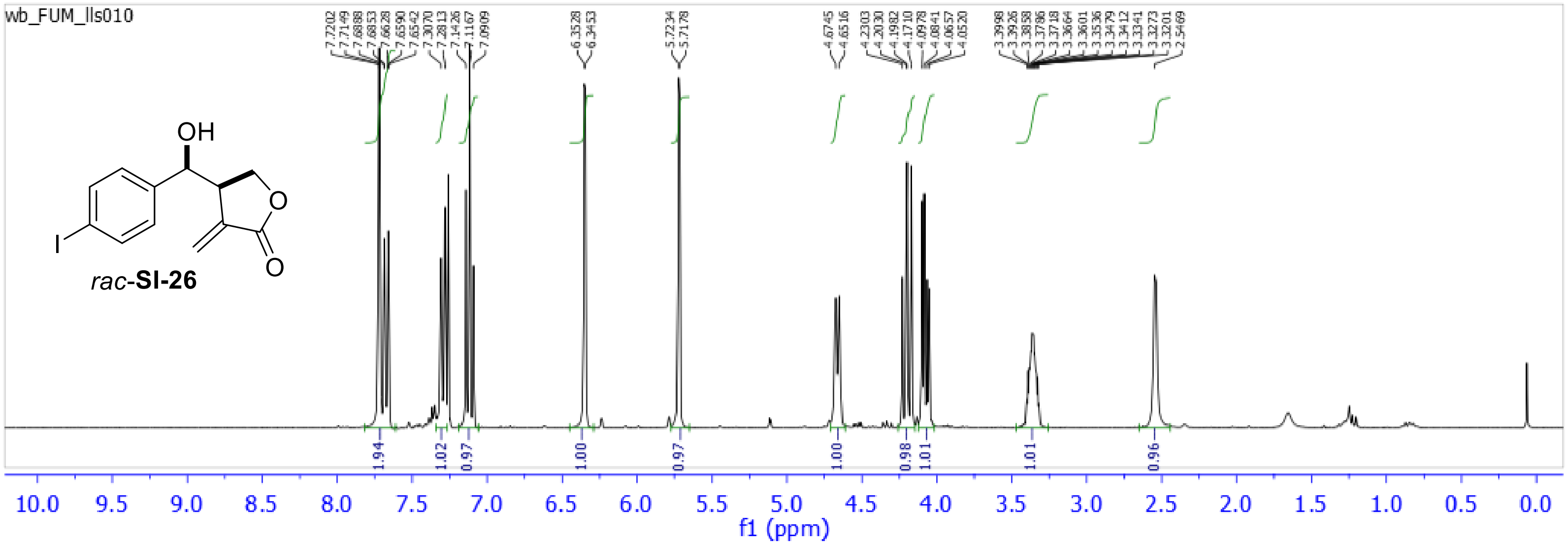

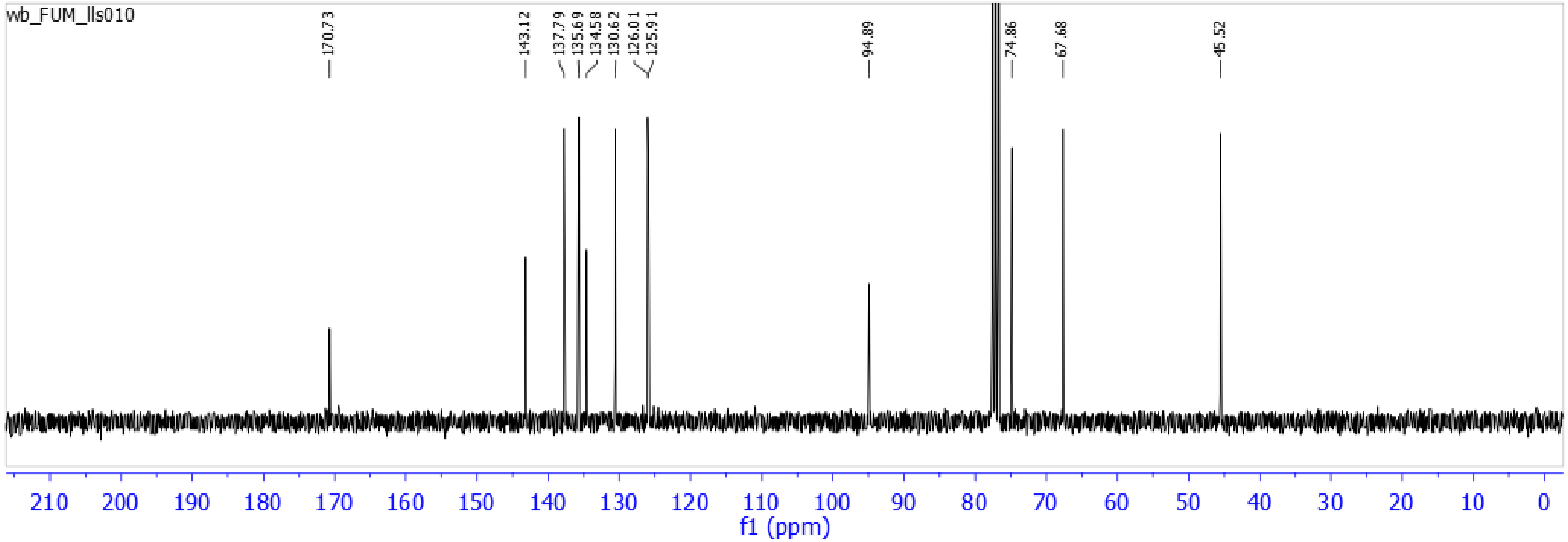

rac-4-[Benzo[d][1,3]dioxol-5-yl(hydroxy)methyl]-3-(3,4,5-trimethoxybenzyl)dihydrofuran-2(3H)-one (2d).

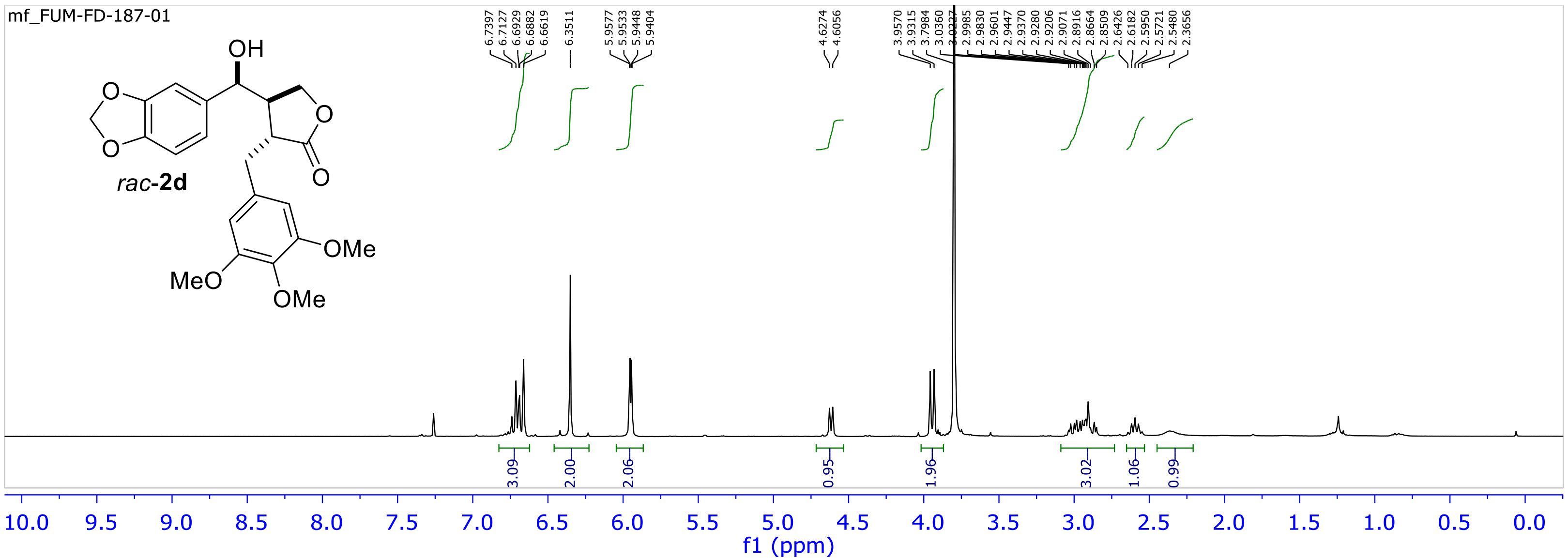

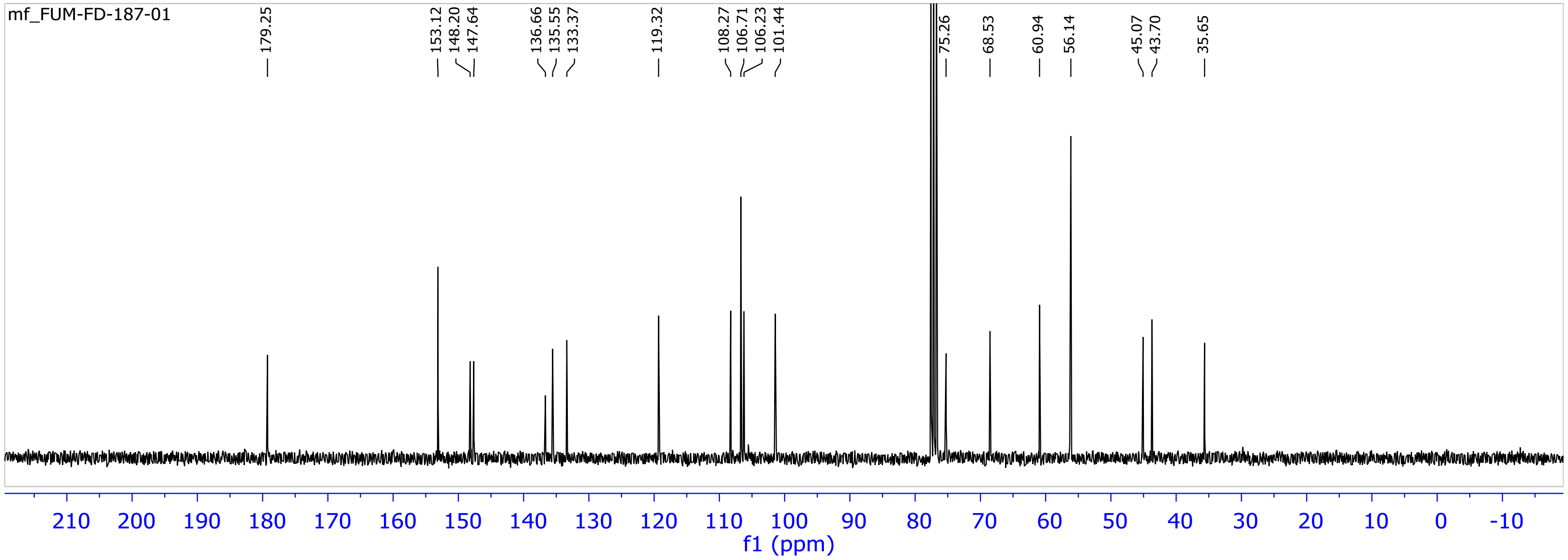

***rac*-4-[(3,4-dimethoxyphenyl)(hydroxy)methyl]-3-(3,4,5-trimethoxybenzyl)dihydrofuran-2(3*H*)-one (2c).**

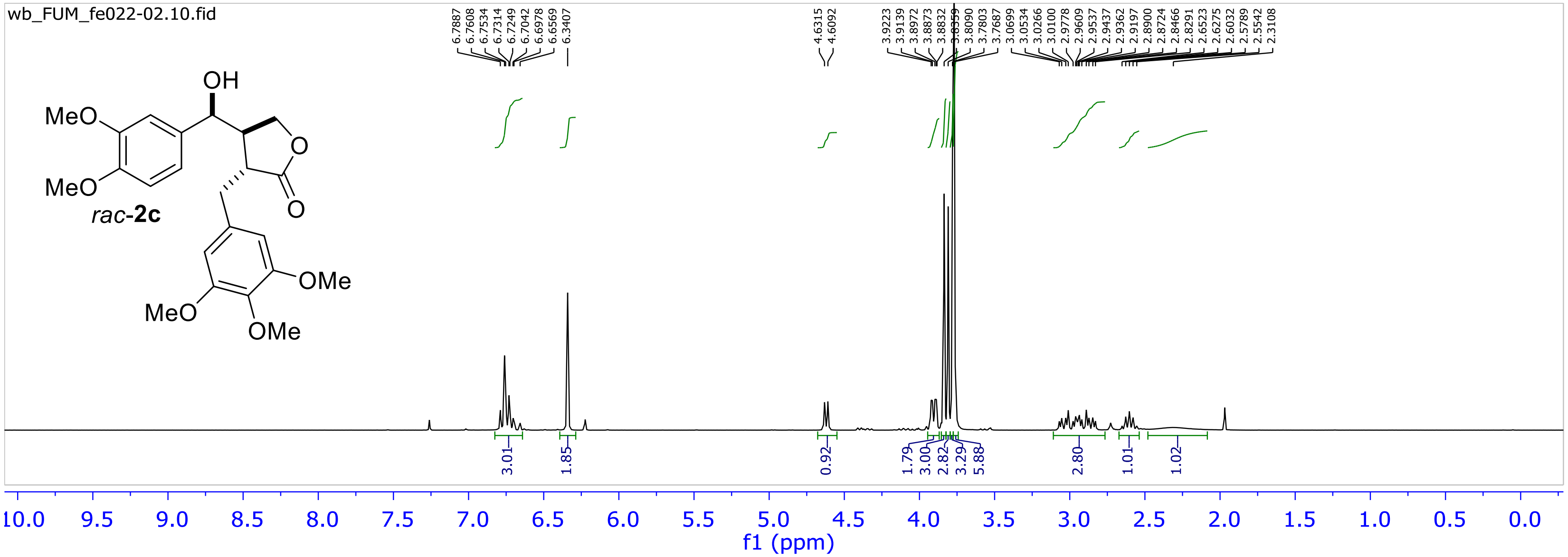

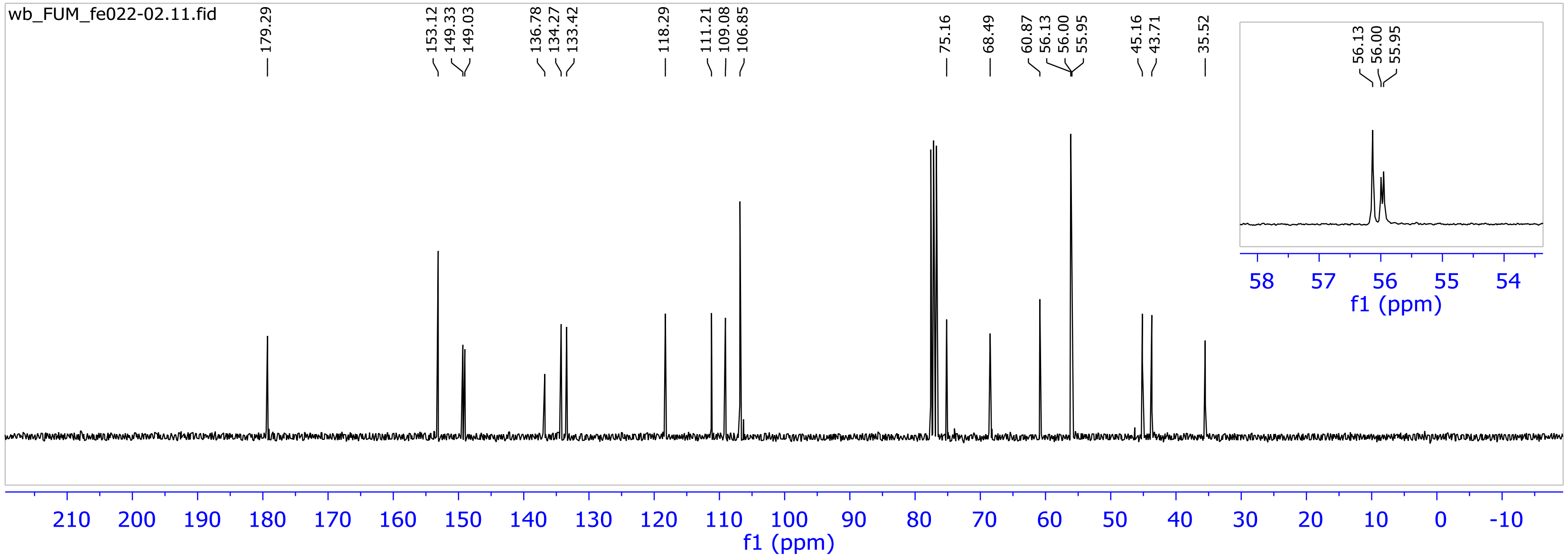

***rac*-4-[hydroxy(phenyl)methyl]-3-(3,4,5-trimethoxybenzyl)dihydrofuran-2(3*H*)-one (2e).**

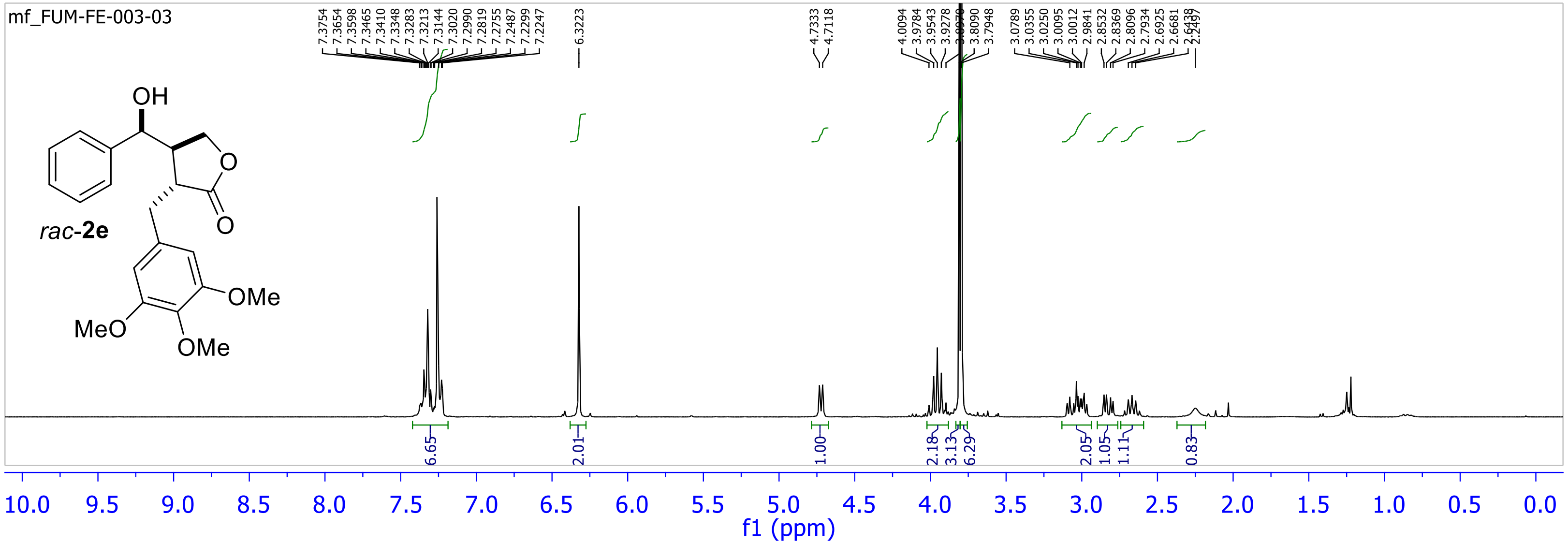

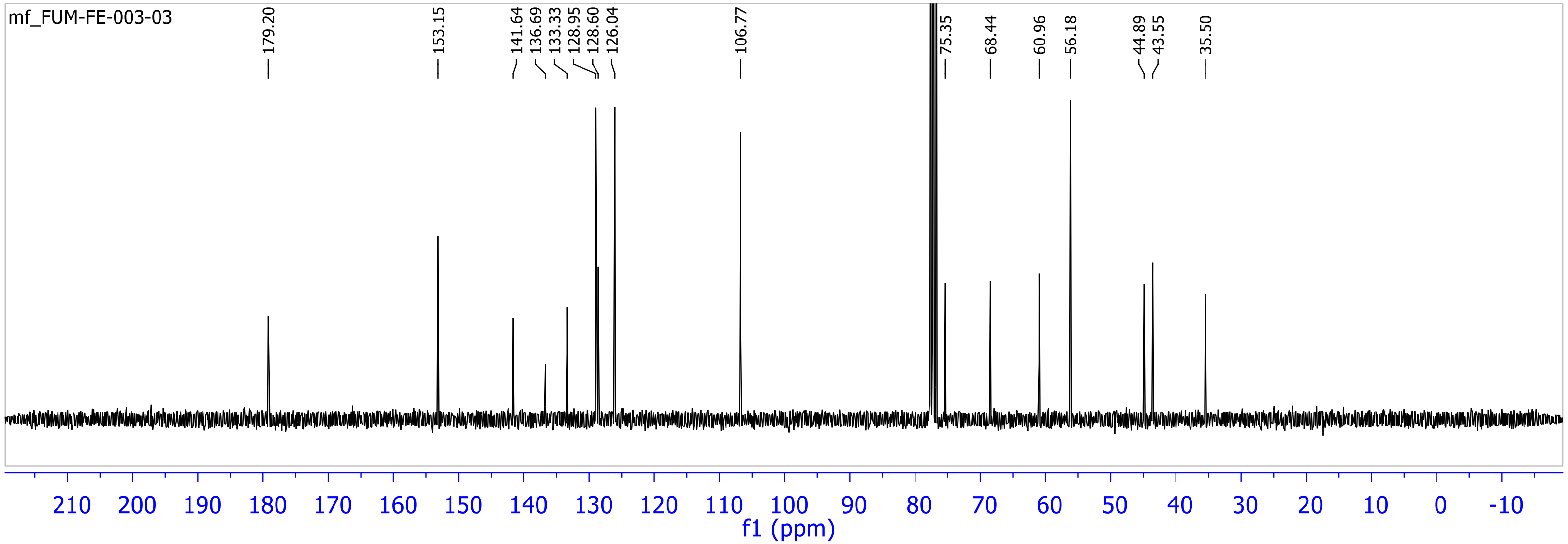

rac-4-[hydroxy(naphthalen-2-yl)methyl]-3-(3,4,5-trimethoxybenzyl)dihydrofuran-2(3*H*)-one (2f).

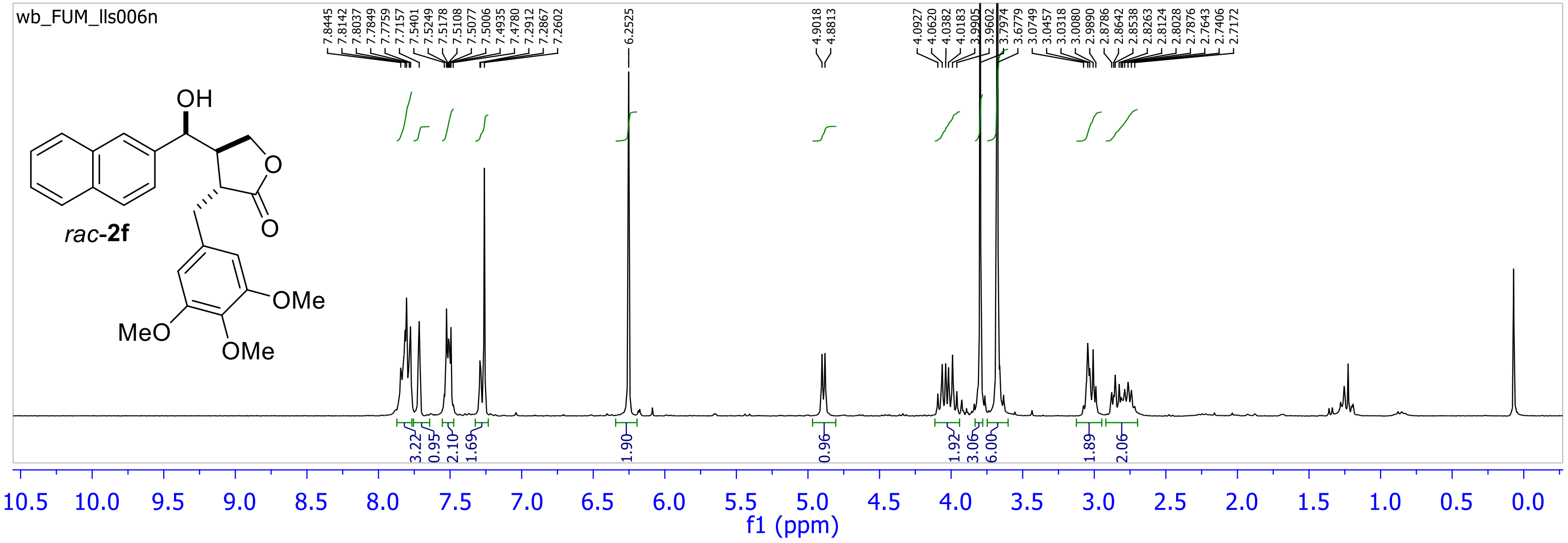

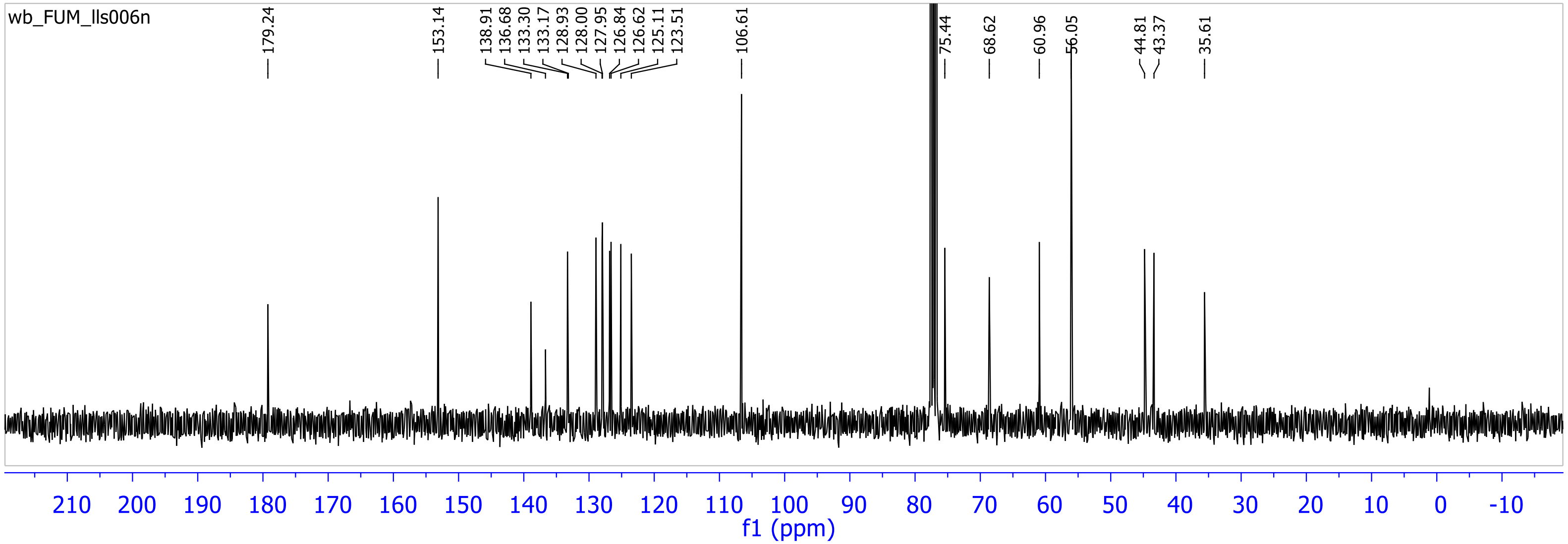

***rac*-4-[(3-chlorophenyl)(hydroxy)methyl]-3-(3,4,5-trimethoxybenzyl)dihydrofuran-2(3*H*)-one (2g).**

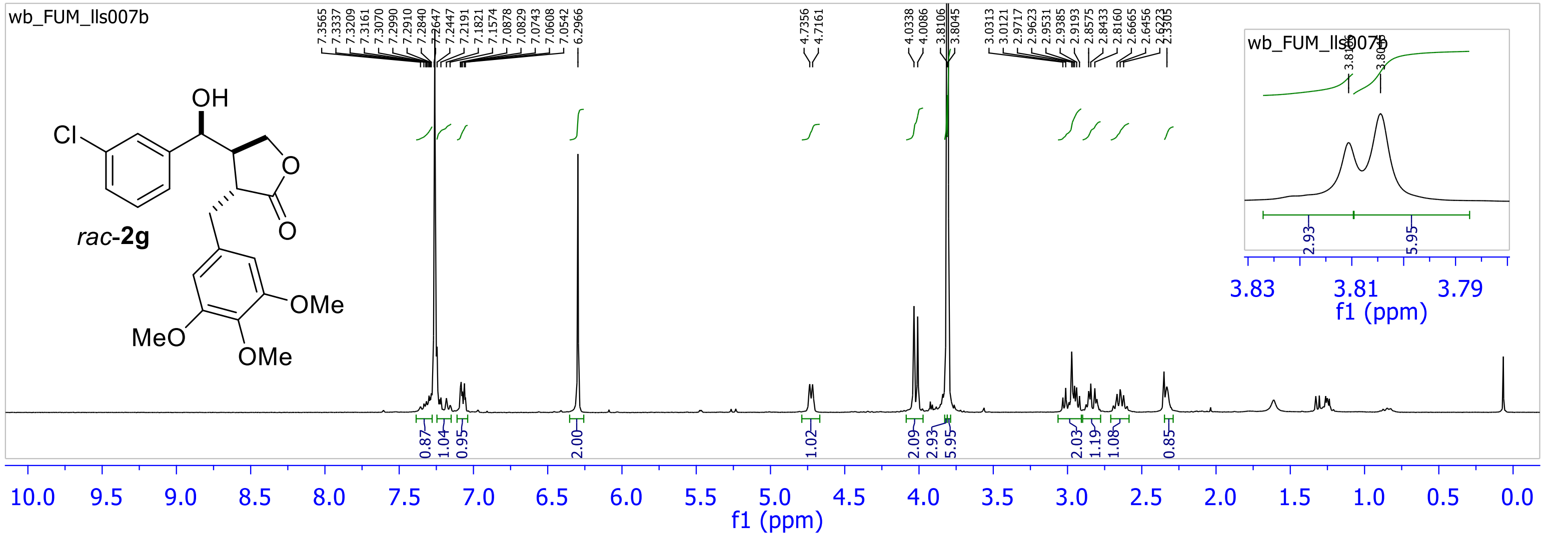

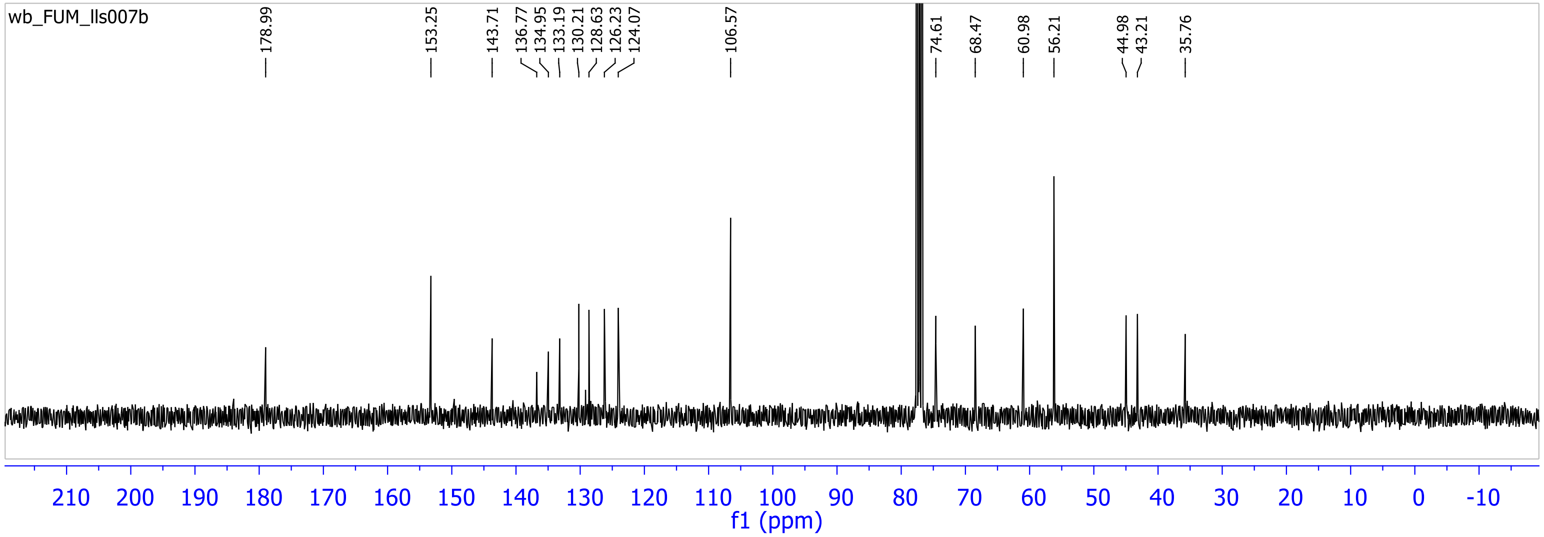

***rac*-4-[(4-chlorophenyl)(hydroxy)methyl]-3-(3,4,5-trimethoxybenzyl)dihydrofuran-2(3*H*)-one (2h).**

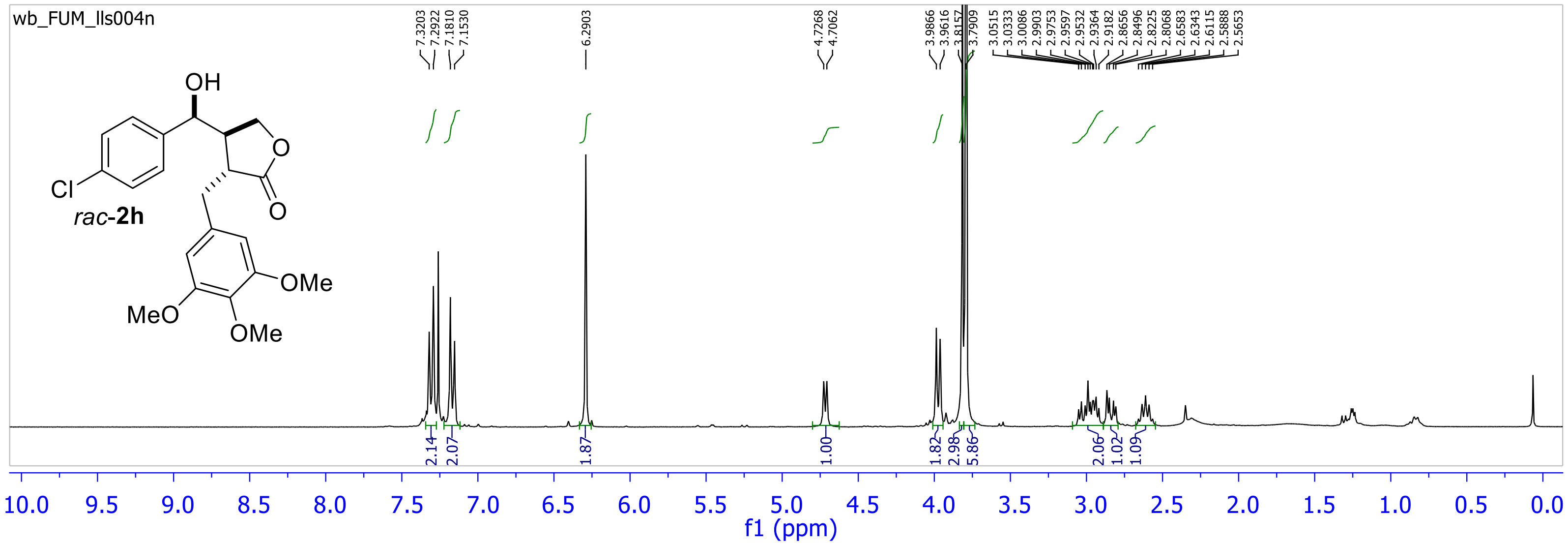

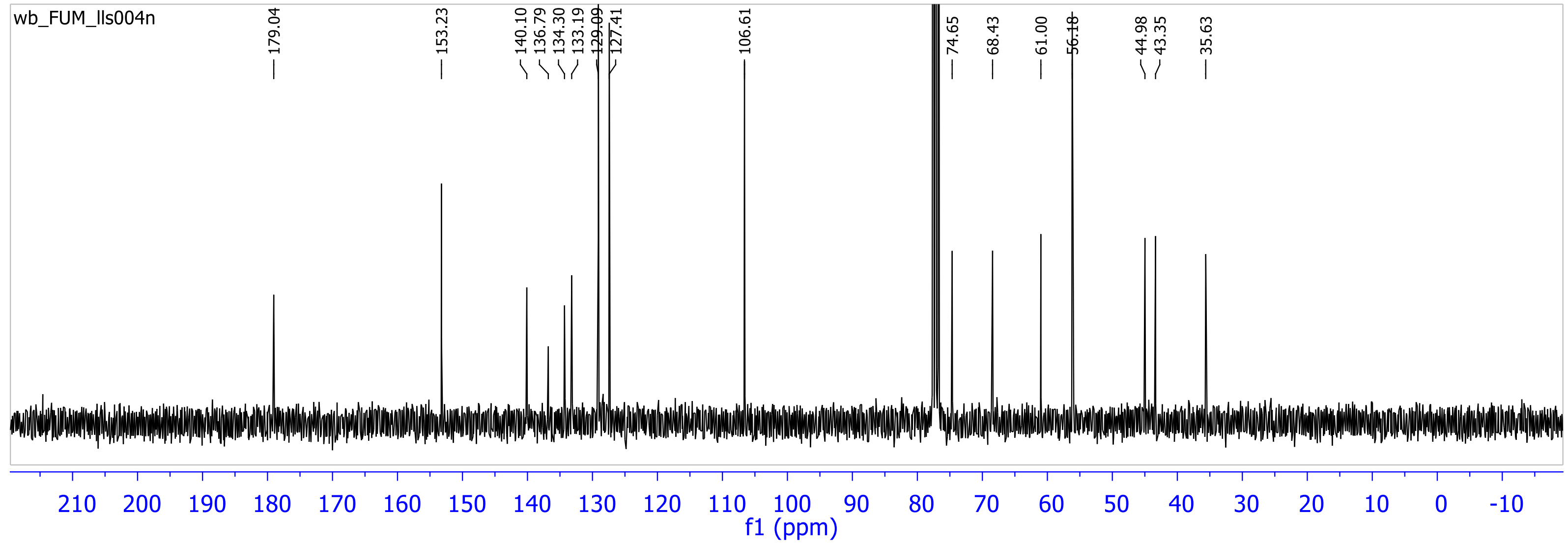

***rac*-4-[(3-Iodophenyl)(hydroxy)methyl]-3-(3,4,5-trimethoxybenz-yl)dihydrofuran-2(3*H*)-one (2i).**

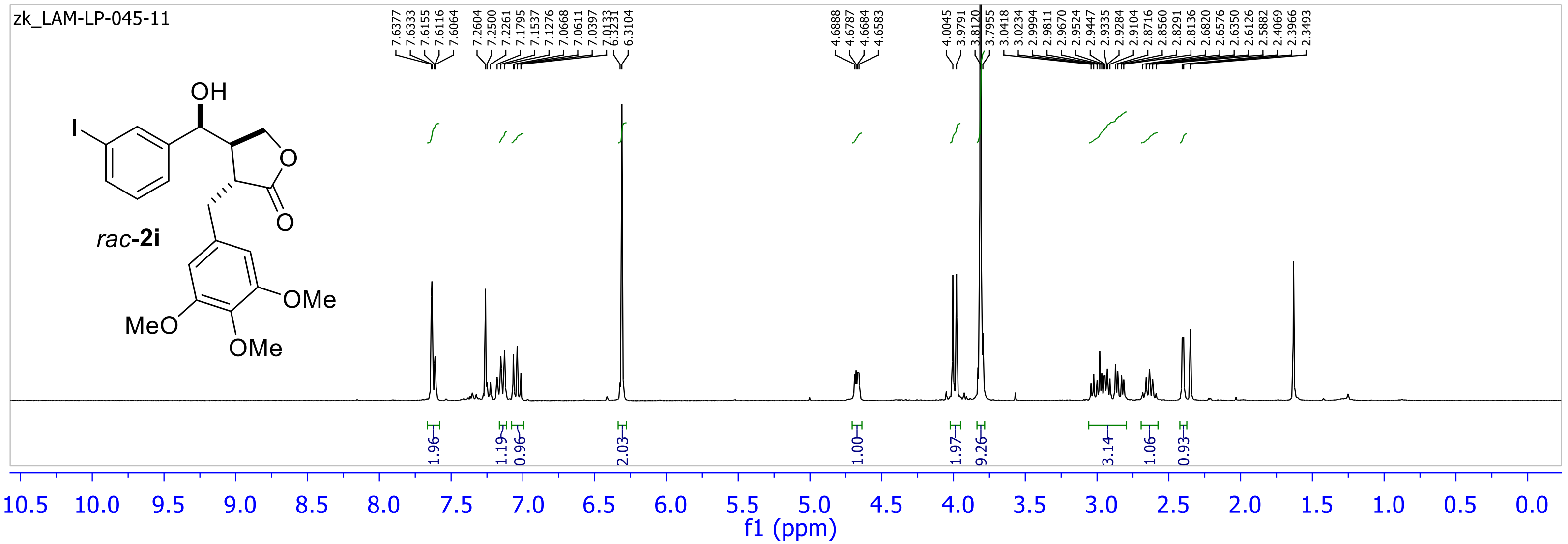

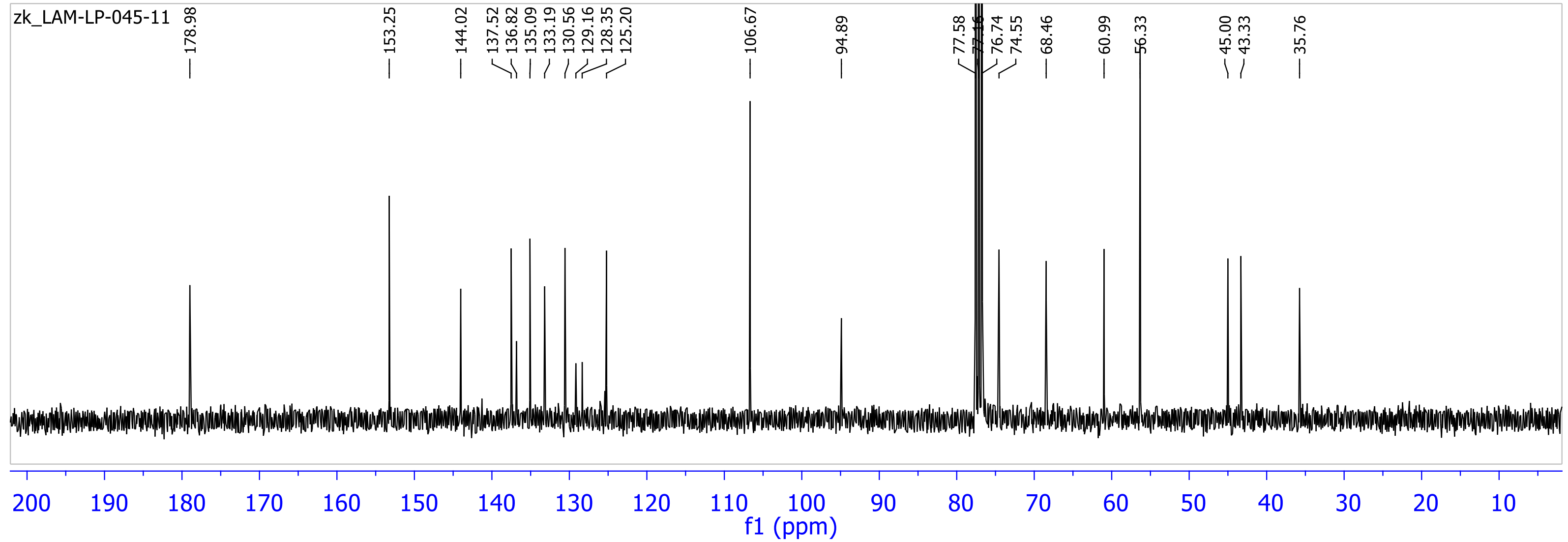

rac-Methyl 4-{hydroxy[5-oxo-4-(3,4,5-trimethoxybenzyl)tetrahydrofuran-3-yl]methyl}benzoate (2j).

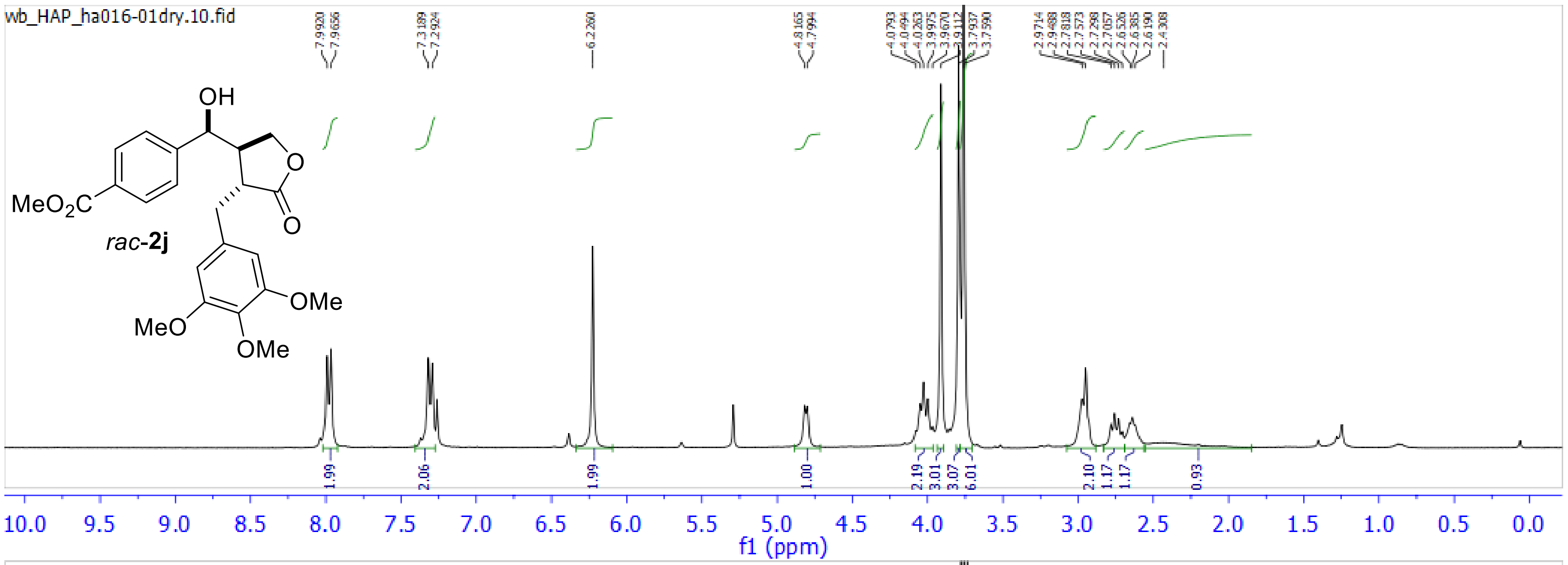

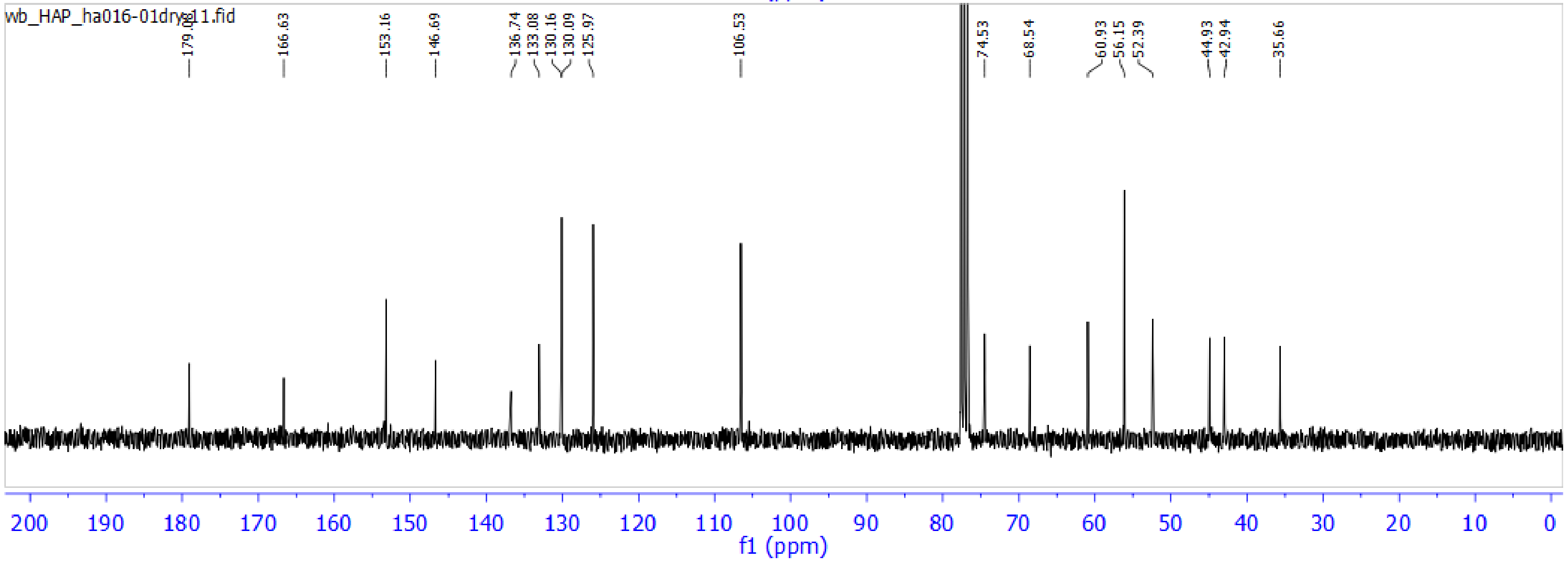

***rac*-4-[hydroxy(4-methoxyphenyl)methyl]-3-(3,4,5-trimethoxybenzyl)dihydrofuran-2(3*H*)-one (2k).**

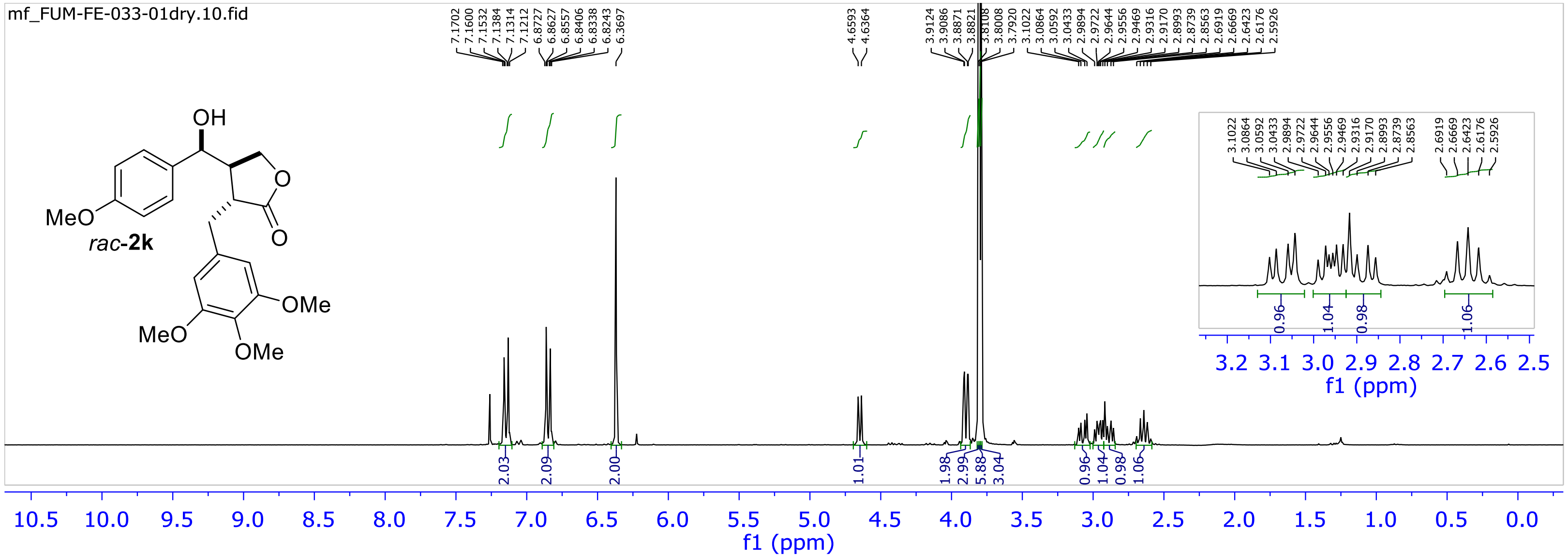

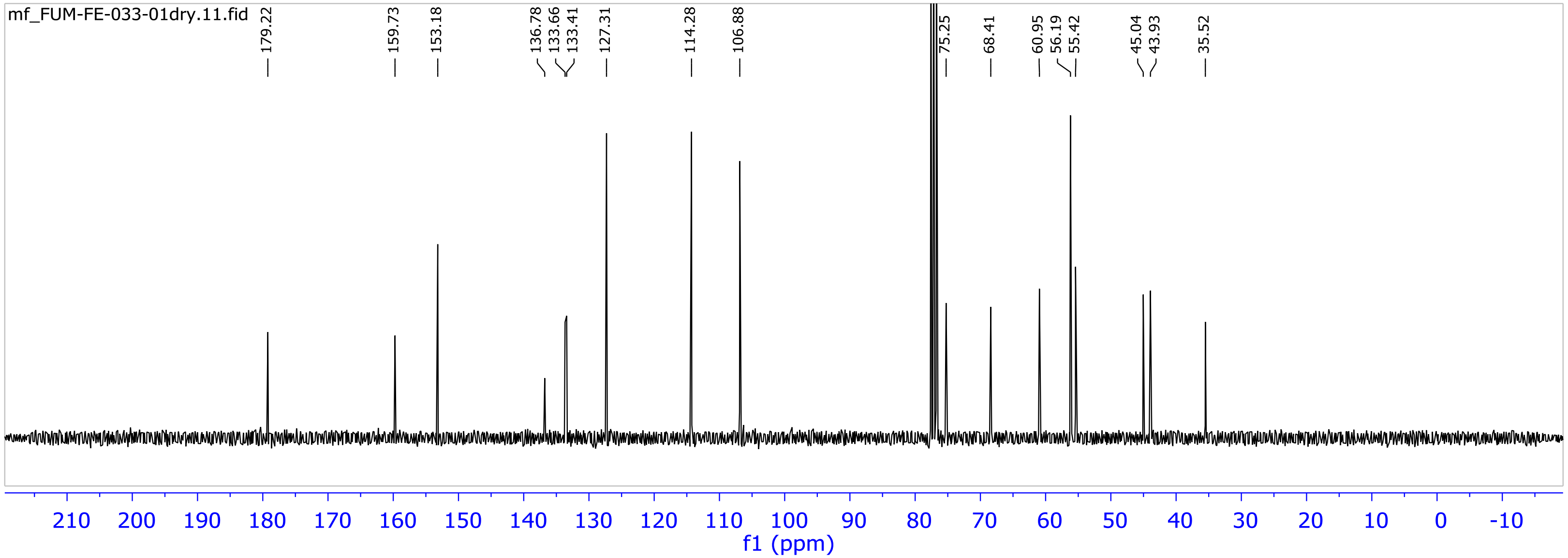

***rac*-4-(hydroxy(3-methoxyphenyl)methyl)-3-(3,4,5-trimethoxybenzyl)dihydrofuran-2(3H)-one (2l)**

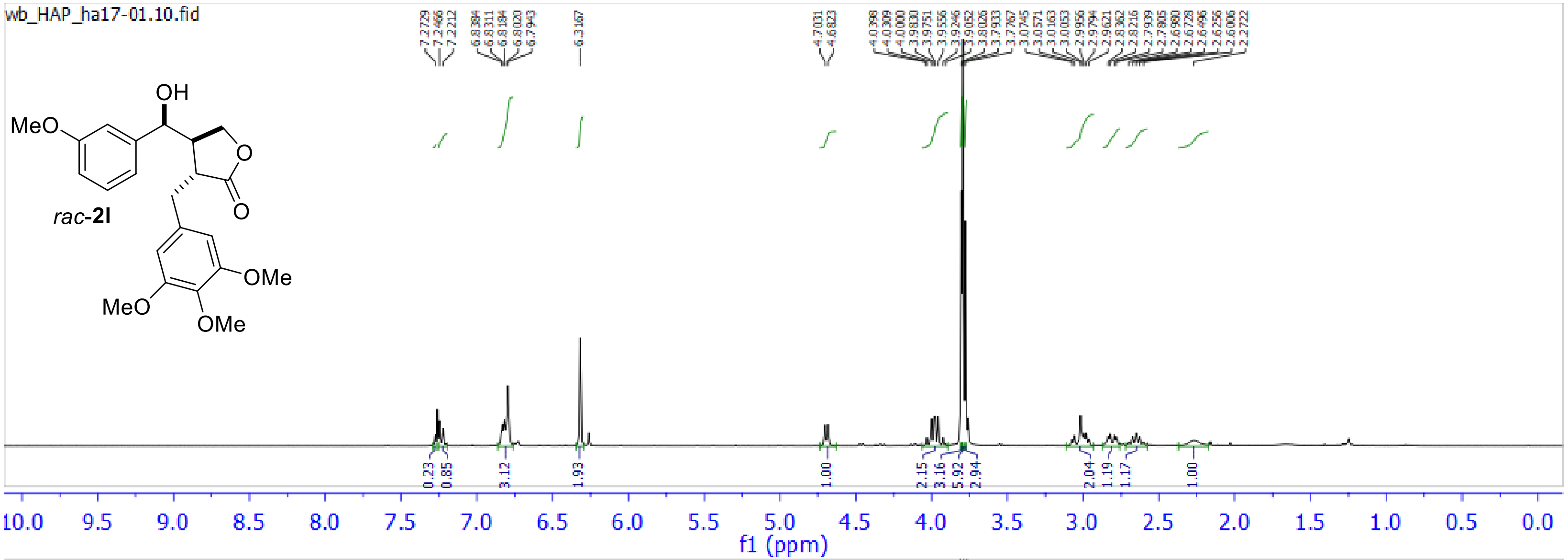

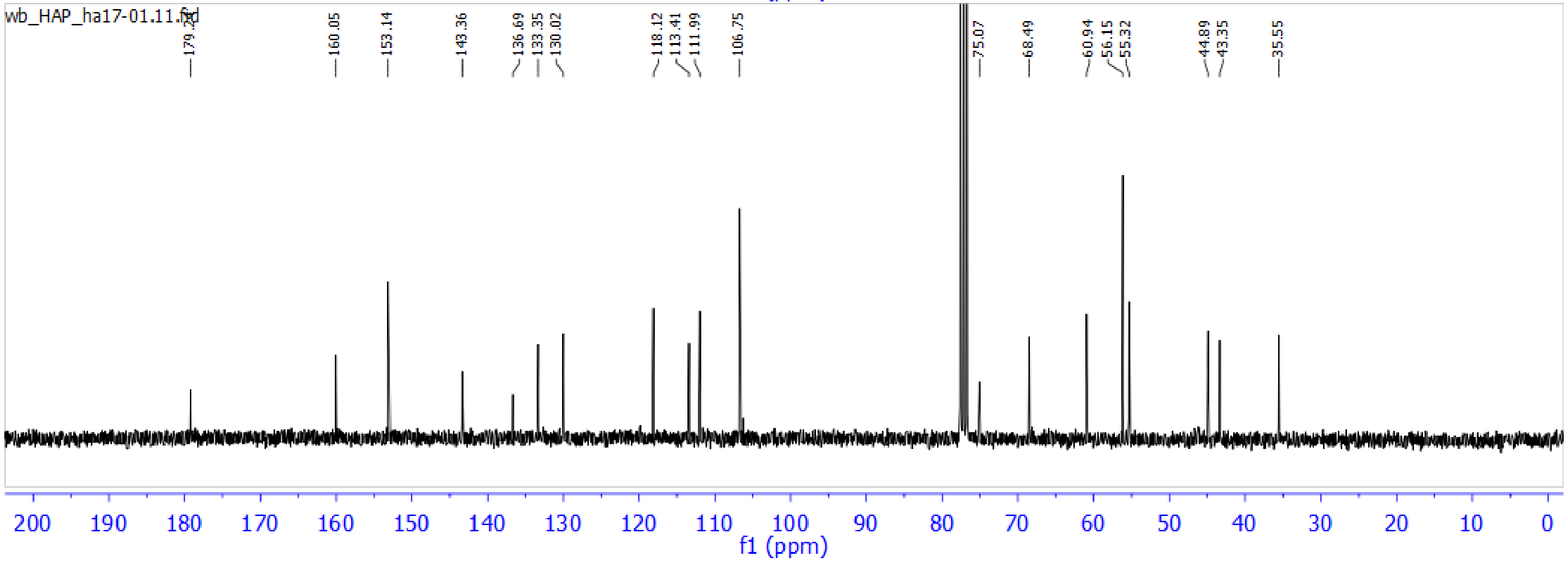

***rac*-4-[hydroxy(4-fluorophenyl)methyl]-3-(3,4,5-trimethoxybenzyl)dihydrofuran-2(3*H*)-one (2m).**

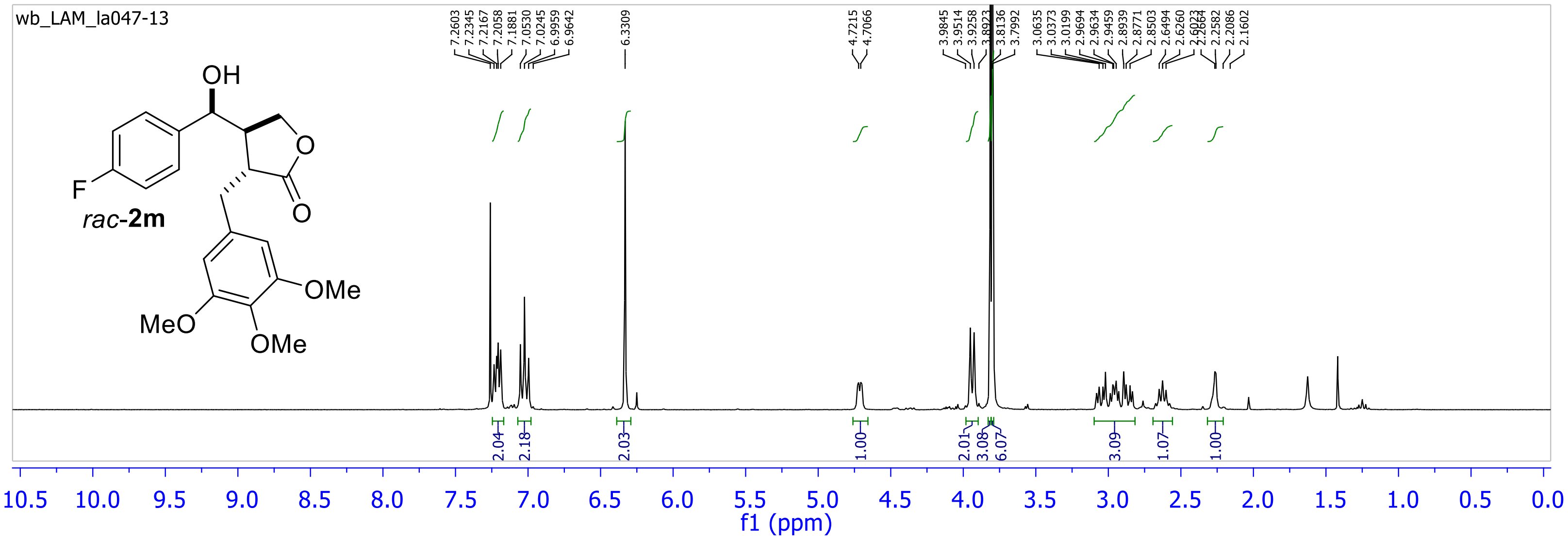

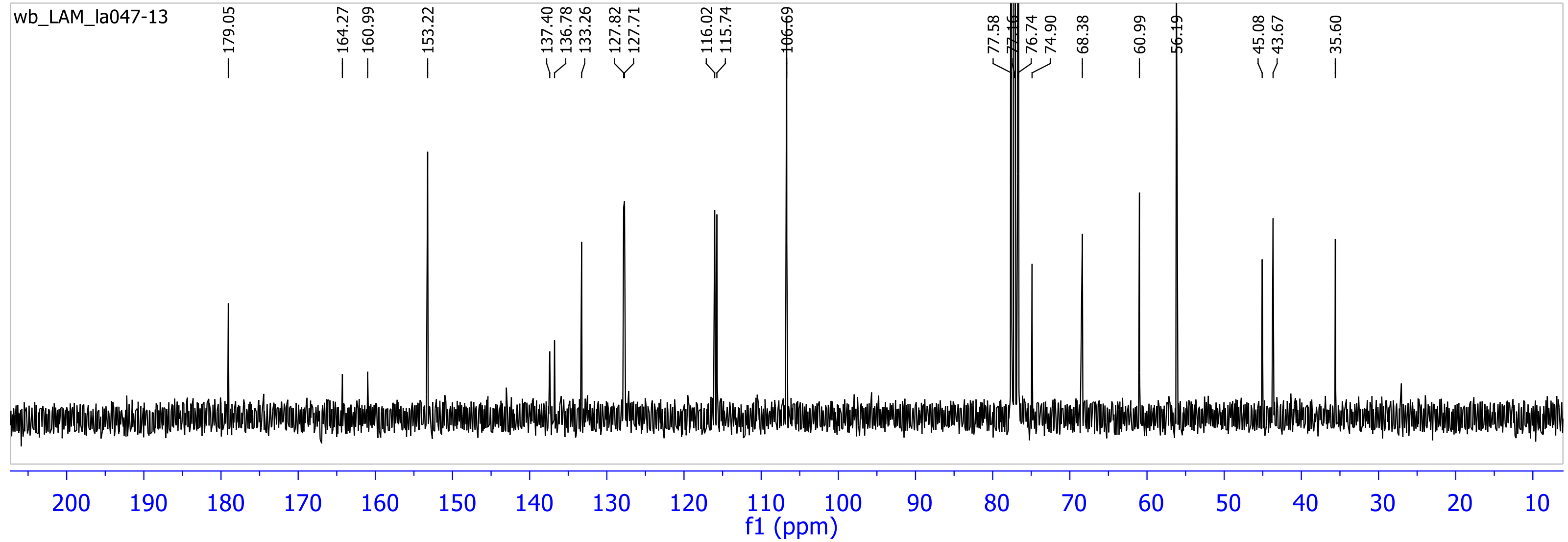

***rac*-4-[(4-iodophenyl)(hydroxy)methyl]-3-(3,4,5-trimethoxybenzyl)dihydrofuran-2(3*H*)-one (SI-1).**

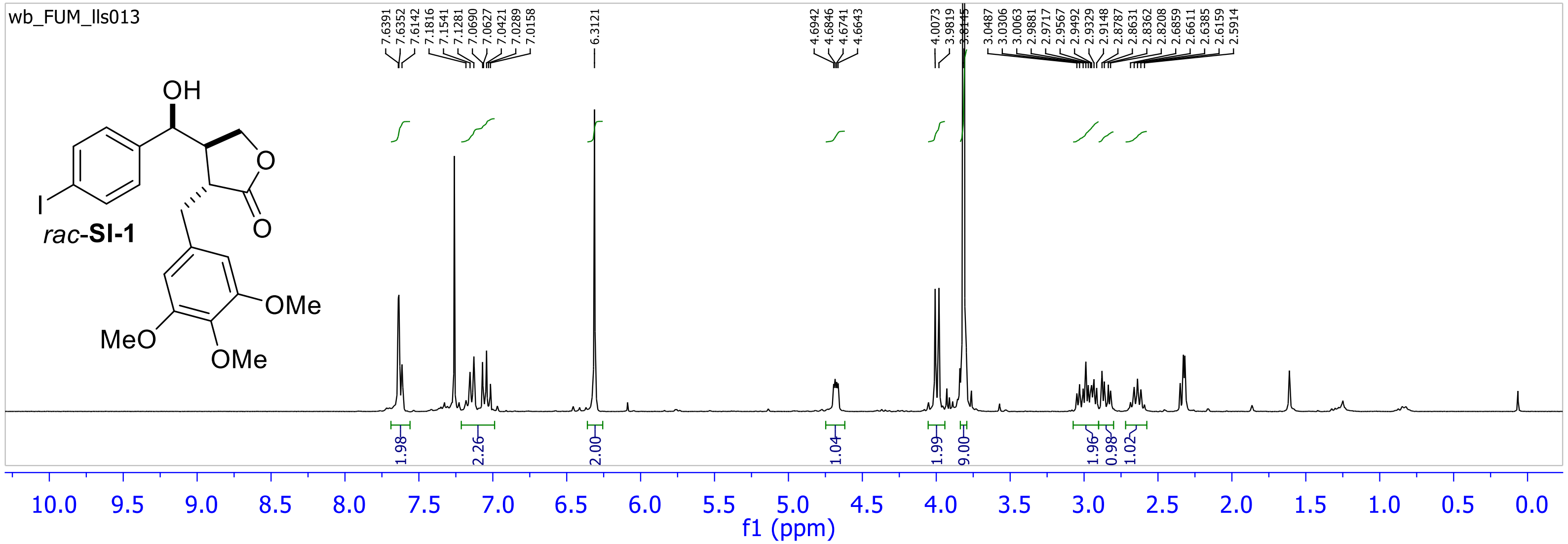

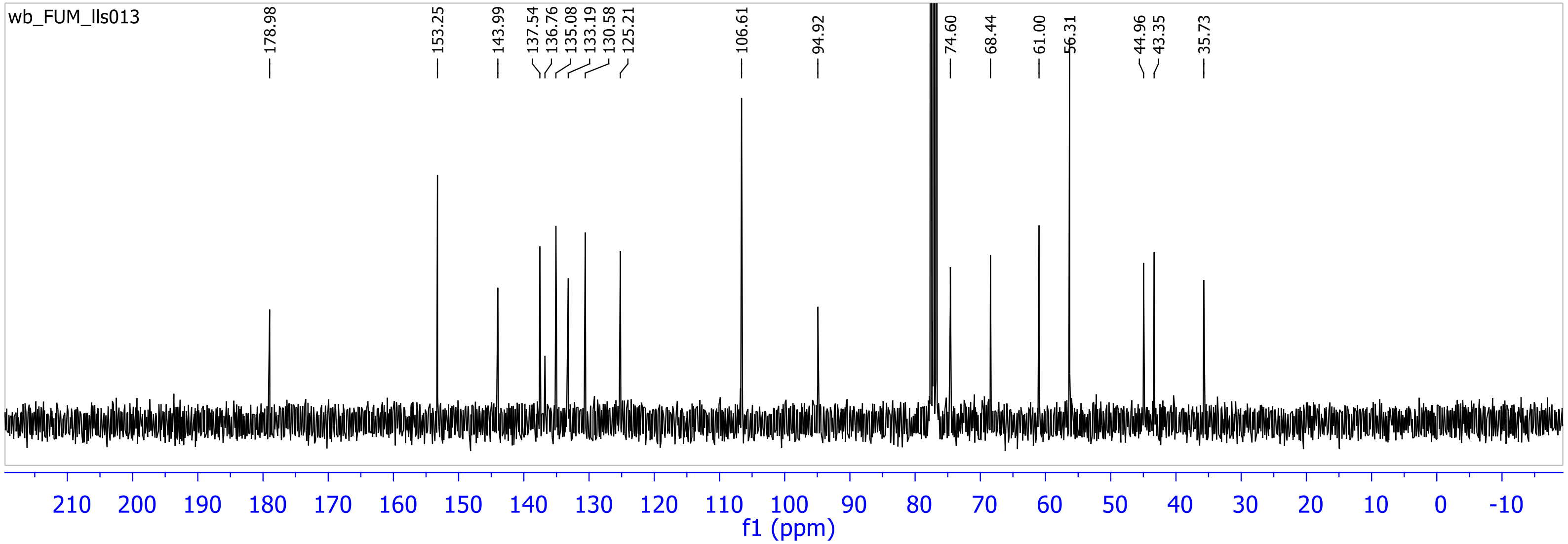

***rac*-4-[Benzo[d][1,3]dioxol-5-yl(hydroxy)methyl]-3-(3,4-dimethoxybenzyl)dihydrofuran-2(3*H*)-one (SI-2).**

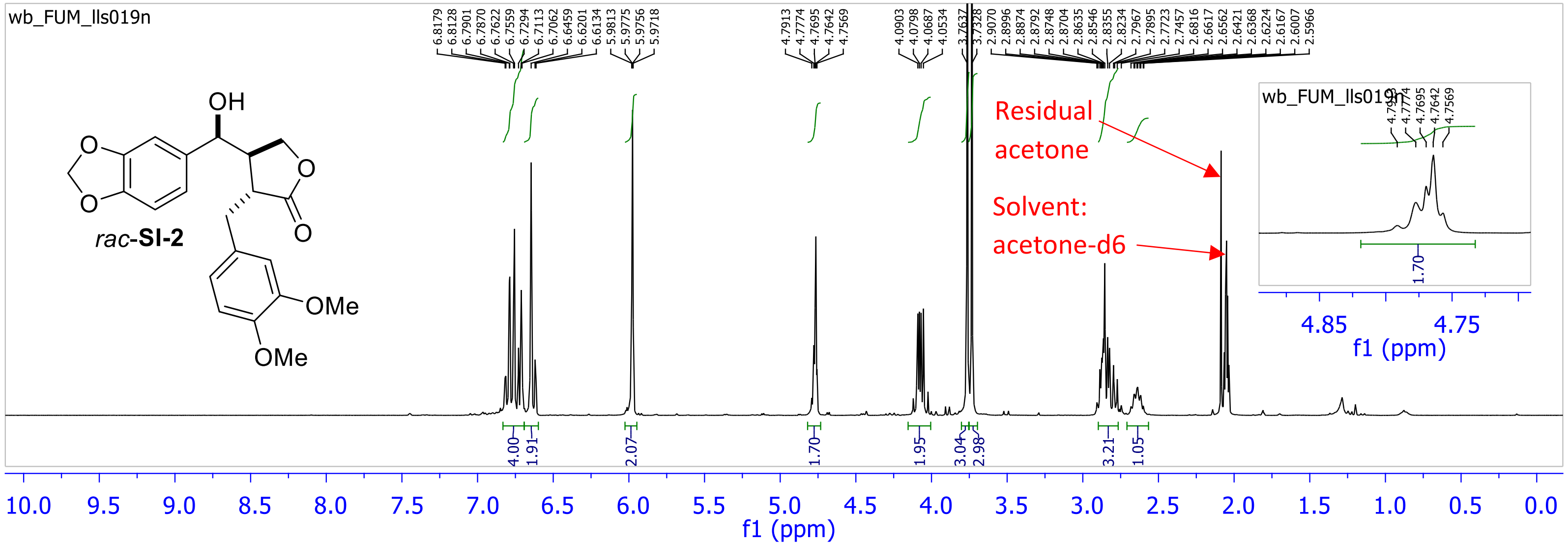

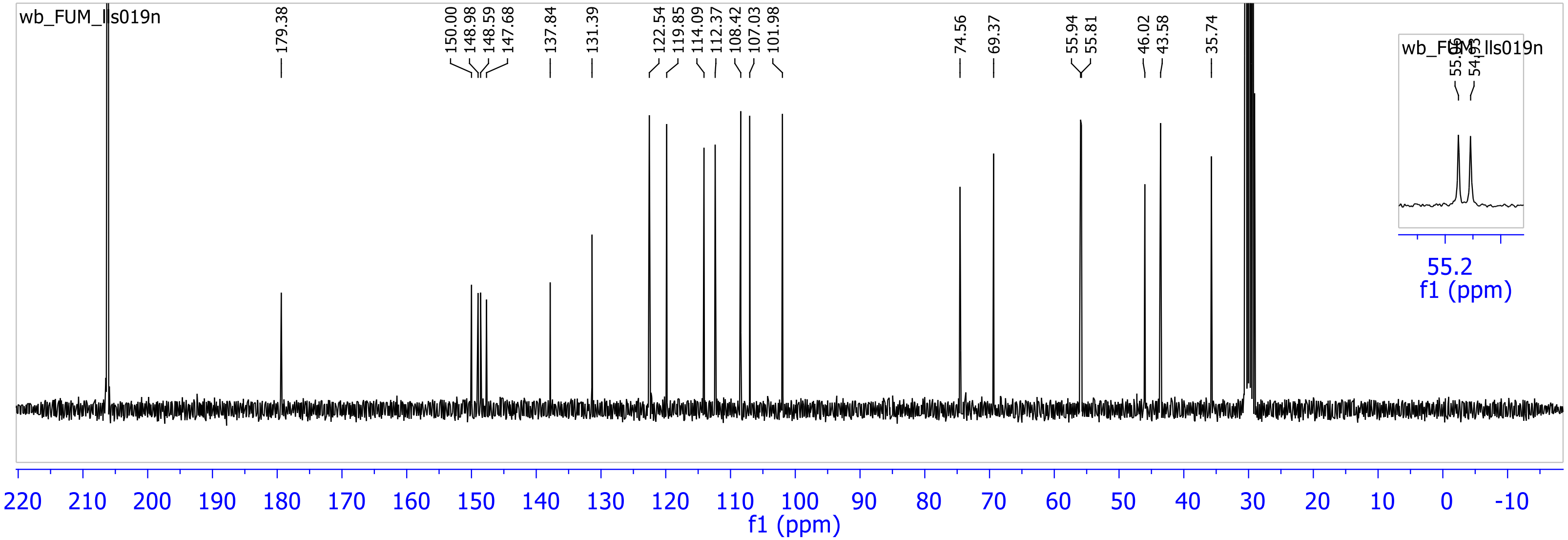

7

***rac*-4-[Benzo[d][1,3]dioxol-5-yl(hydroxy)methyl]-3-(4-methoxybenzyl)dihydrofuran-2(3*H*)-one (SI-3).**

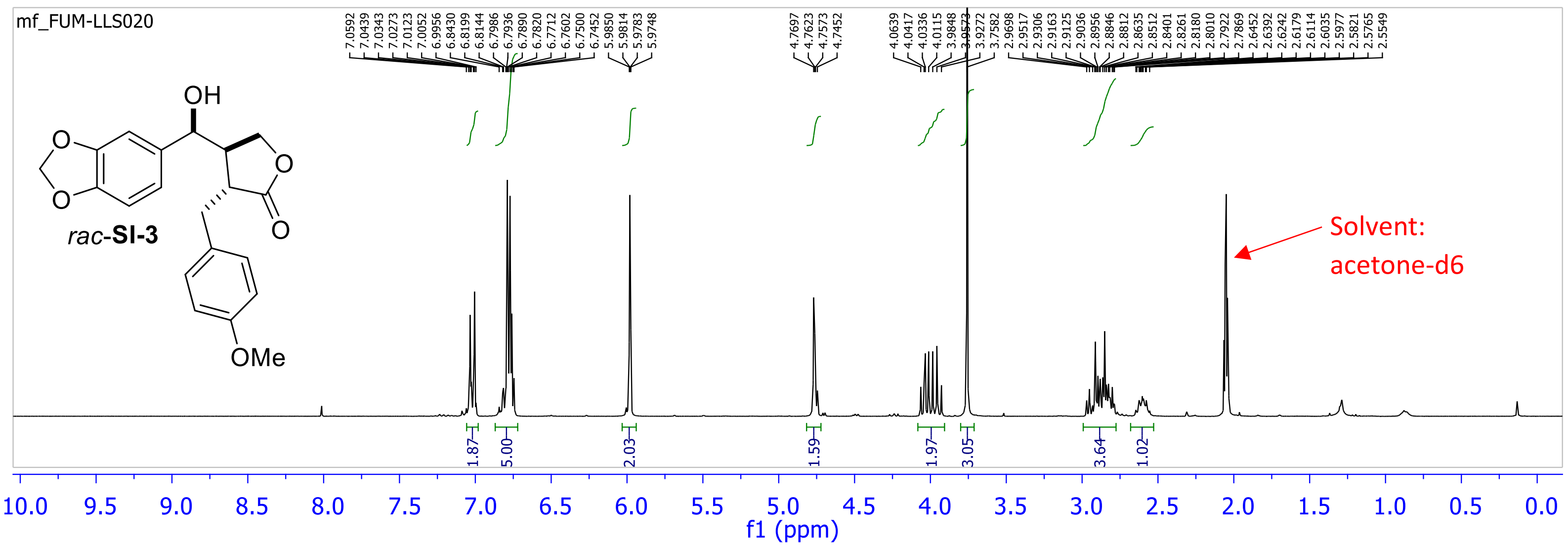

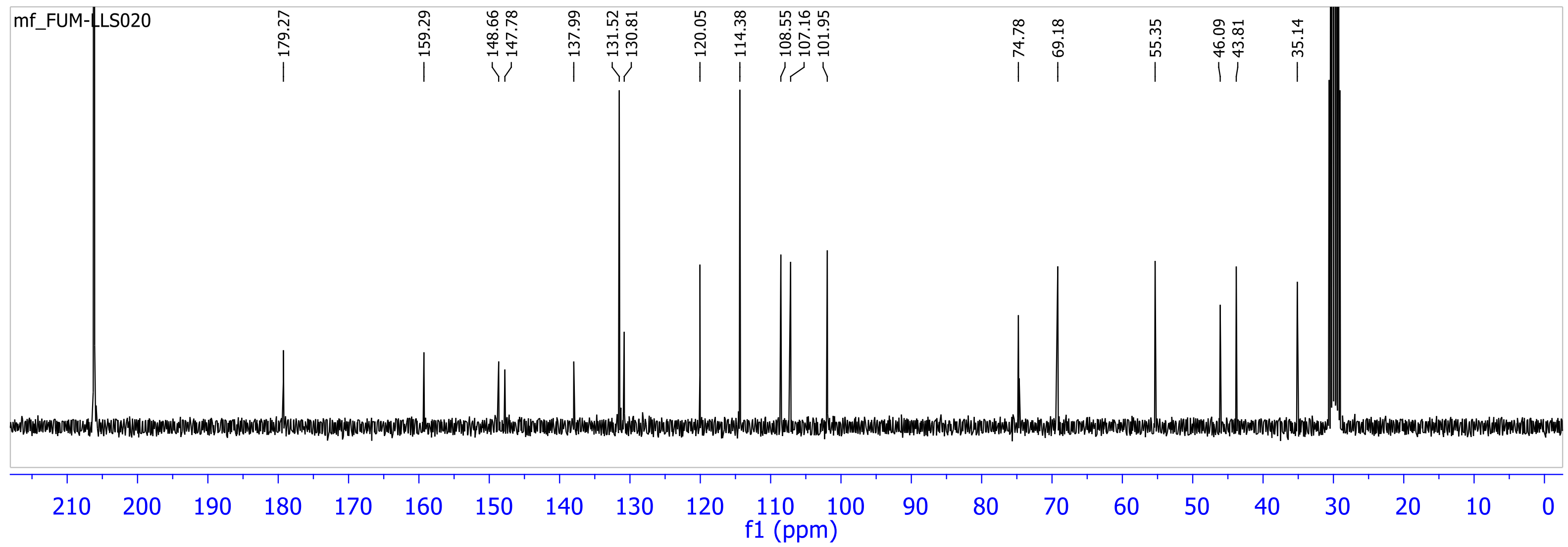

rac-4-[(2-chlorophenyl)(hydroxy)methyl]-3-(3,4,5-trimethoxybenzyl)dihydrofuran-2(3*H*)-one (SI-5).

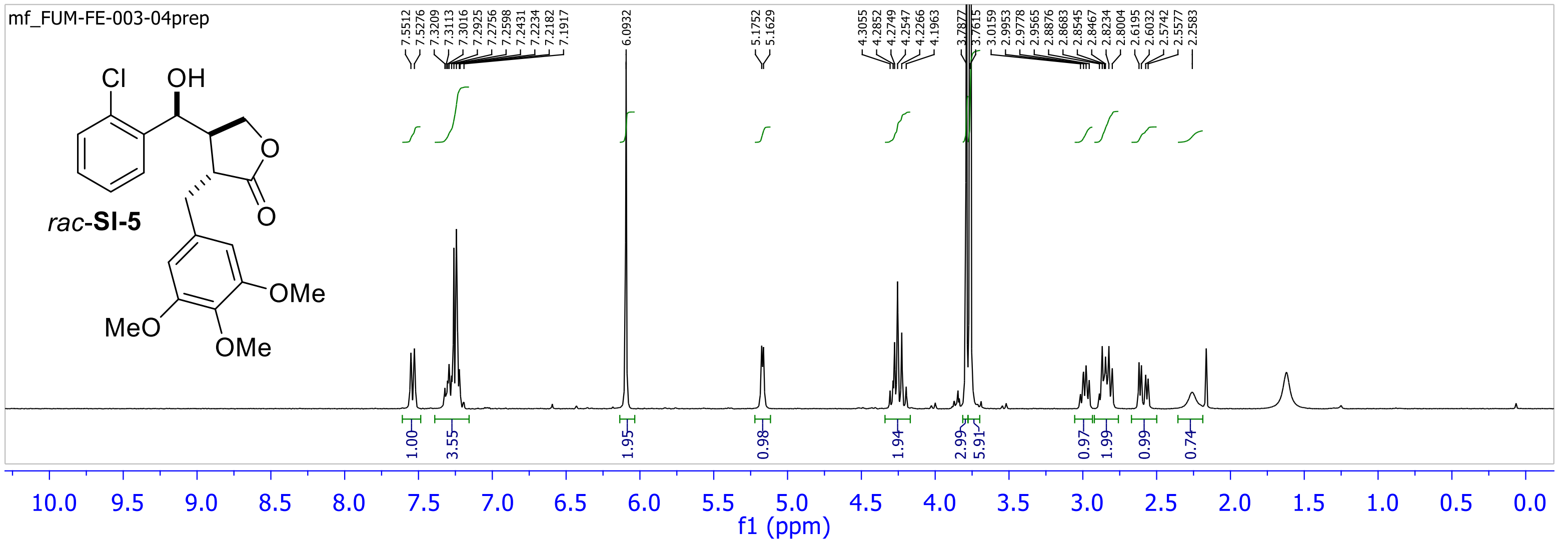

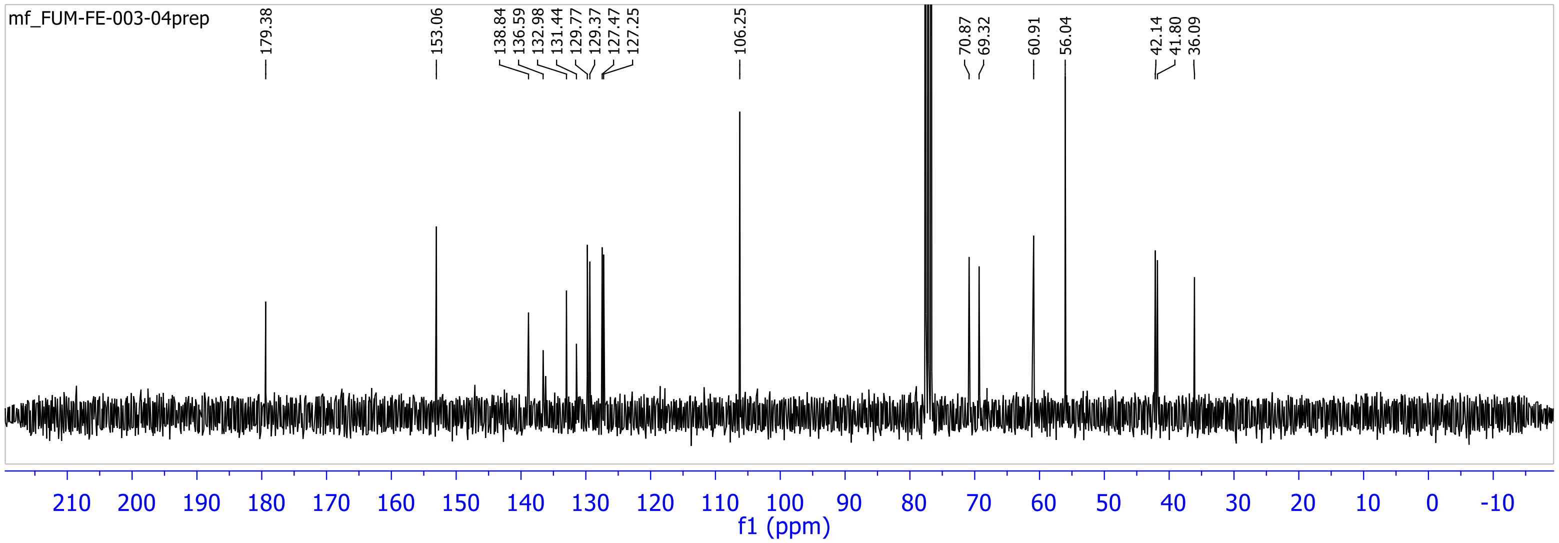

***rac*-4-[furan-2-yl(hydroxy)methyl]-3-(3,4,5-trimethoxybenzyl)dihydrofuran-2(3*H*)-one (SI-6).**

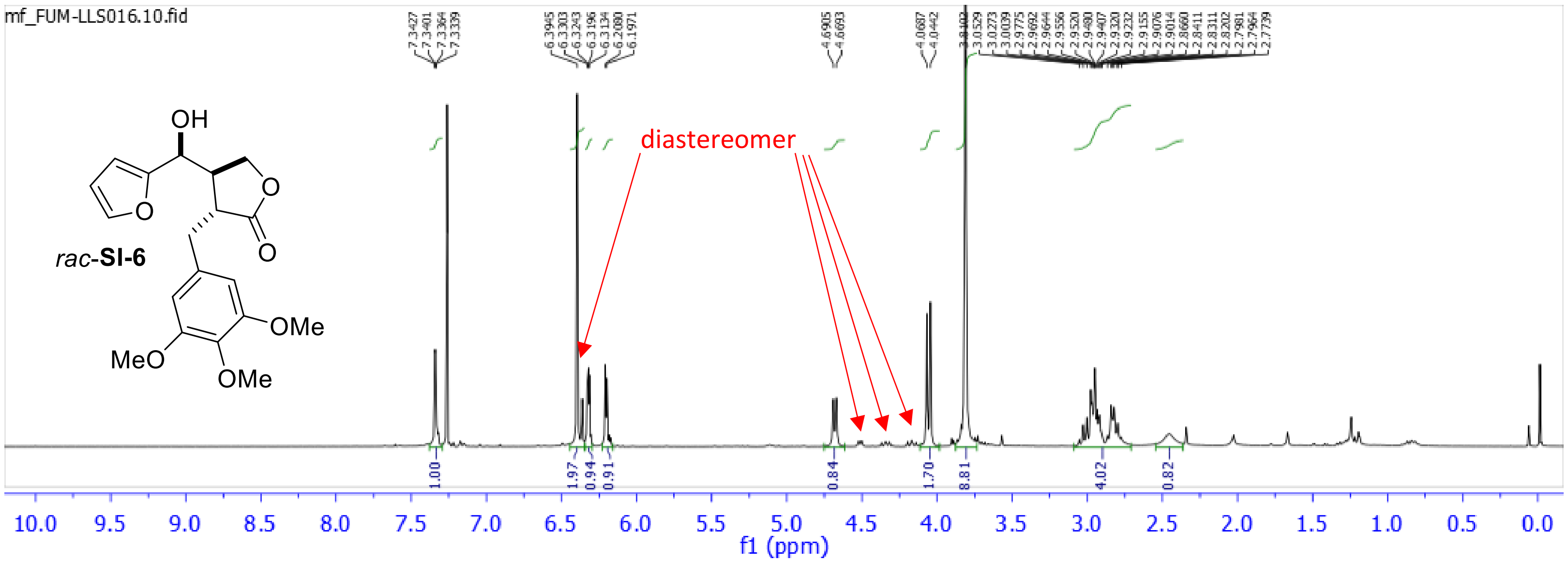

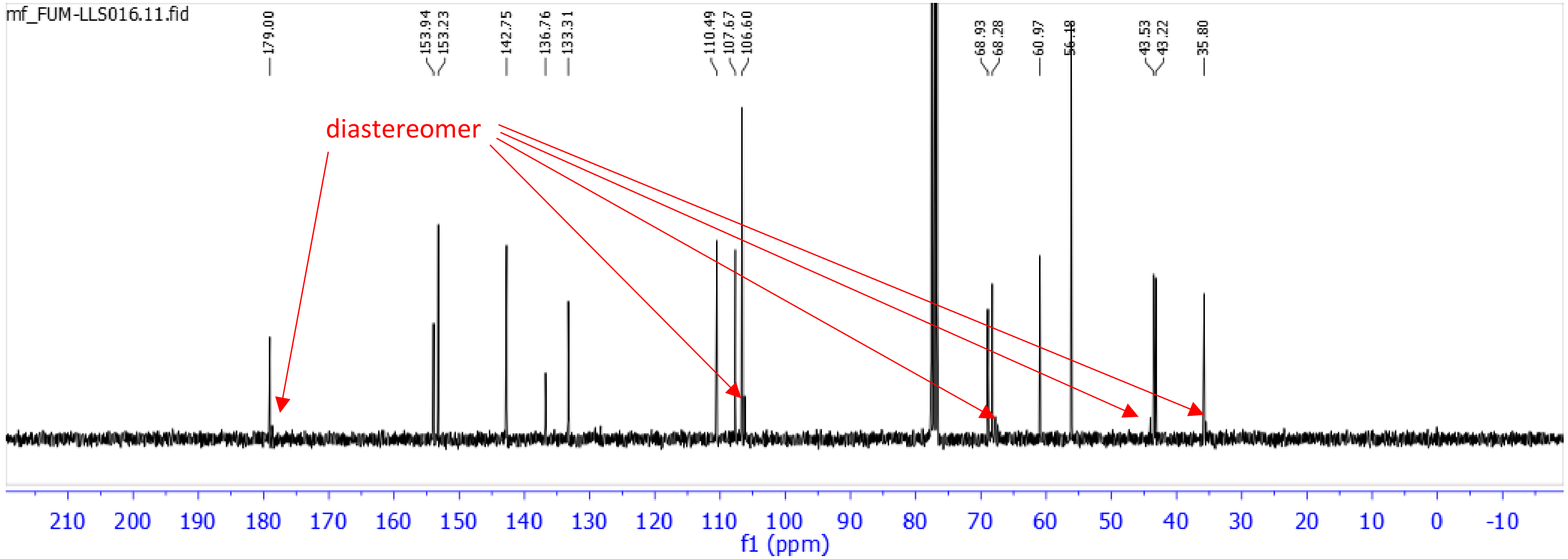

***rac*-4-[hydroxy(4-hydroxyphenyl)methyl]-3-(3,4,5-trimethoxybenzyl)dihydrofuran-2(3*H*)-one (SI-10).**

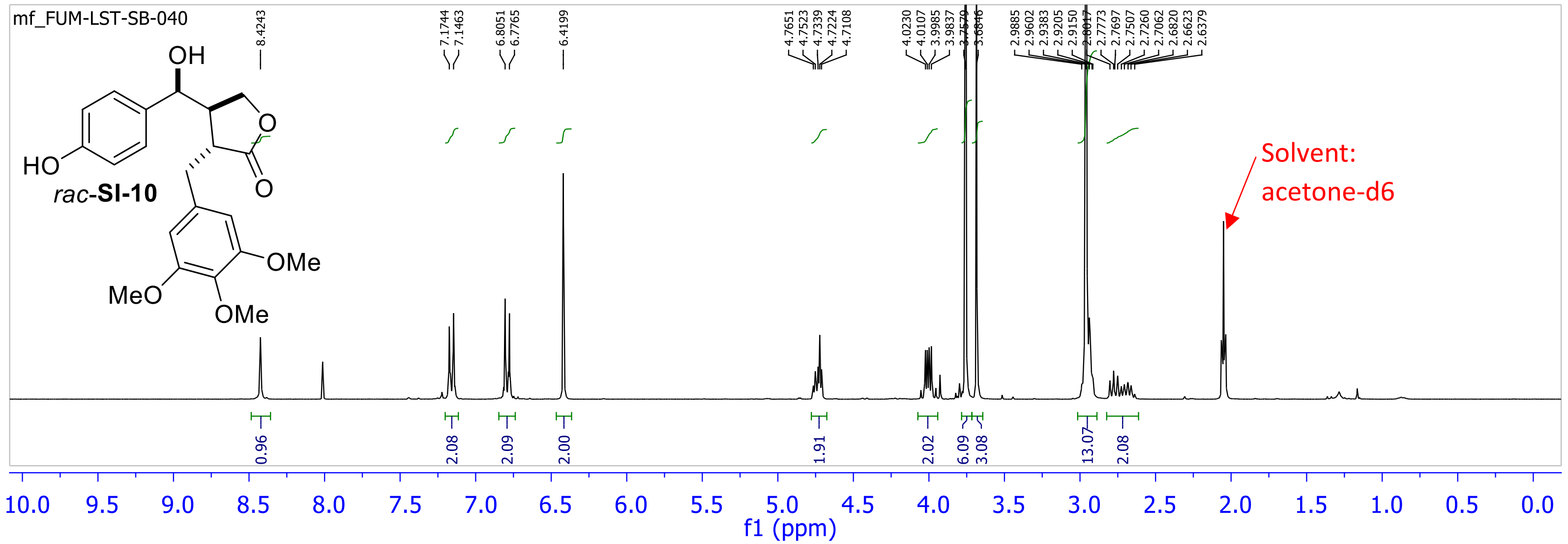

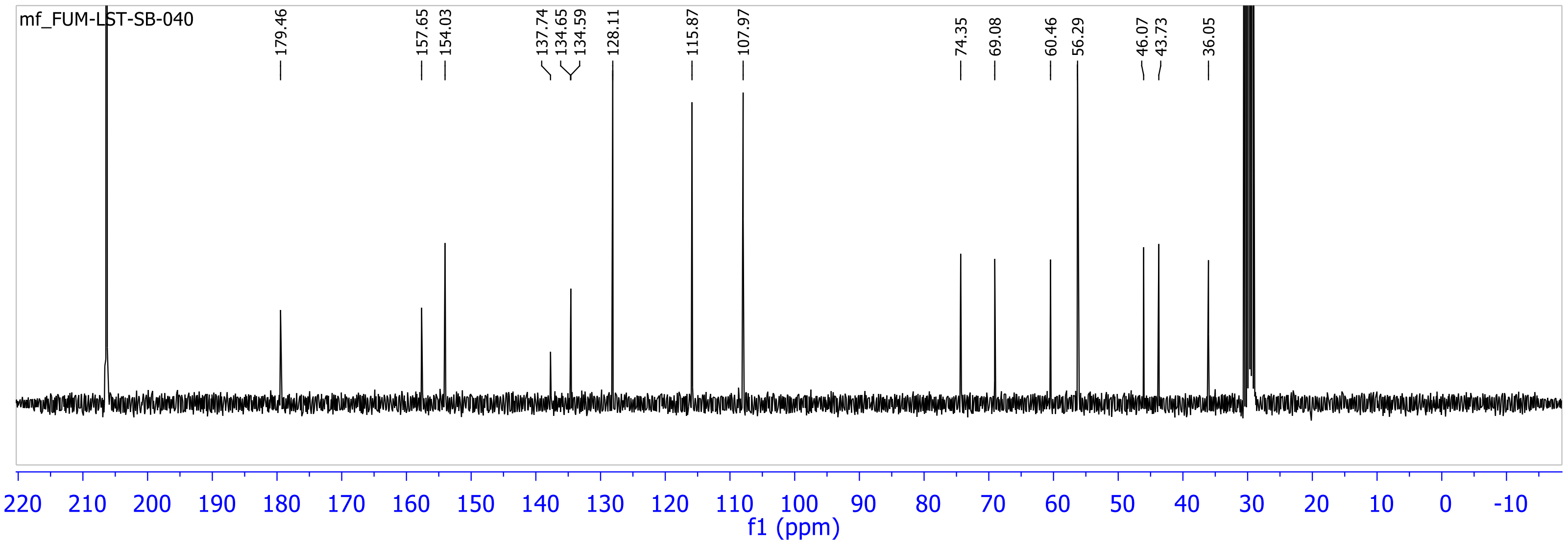

***rac*-Yatein (2a).**

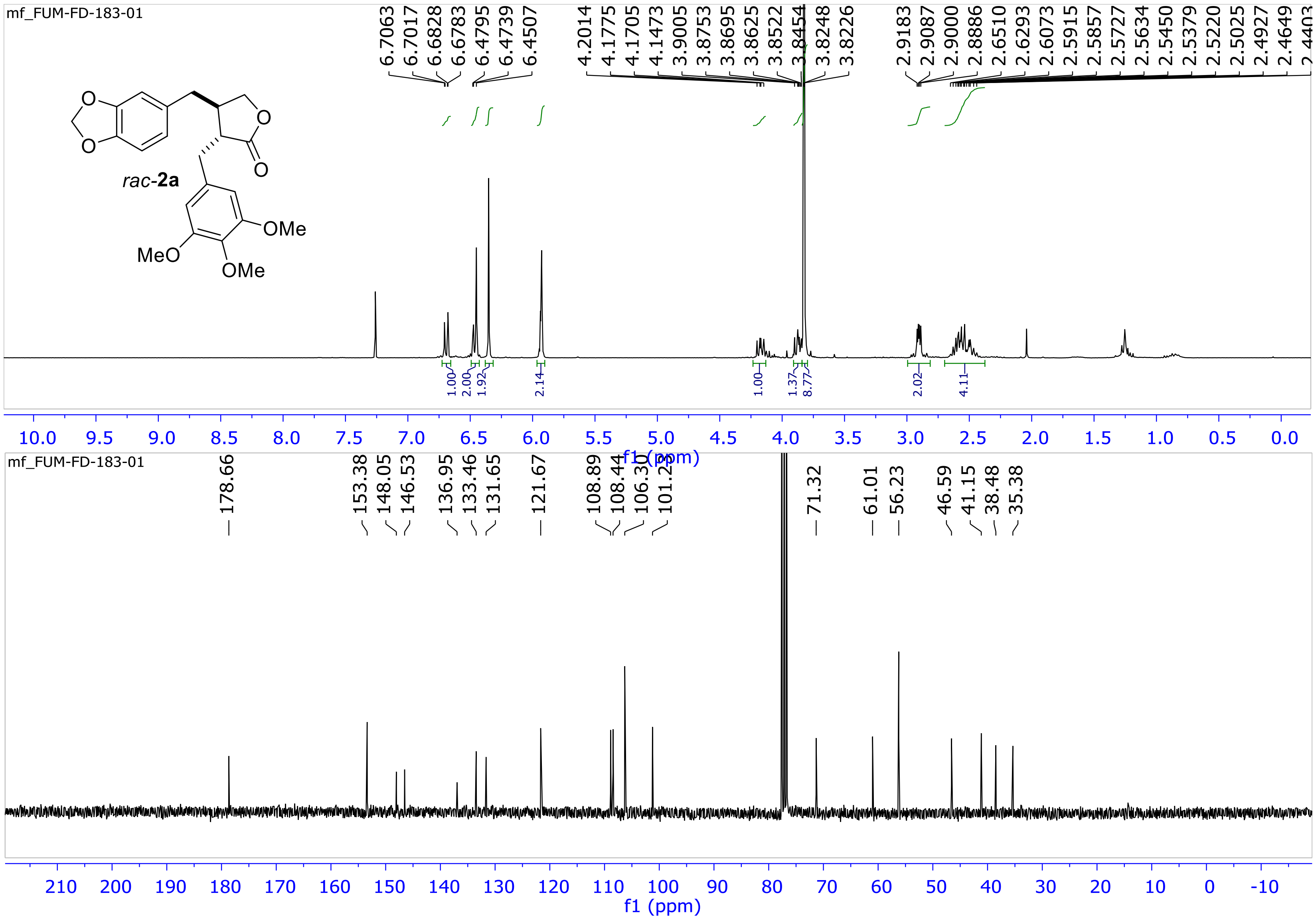

***rac*-4-(benzo[d][1,3]dioxole-5-carbonyl)-3-(3,4,5-trimethoxybenzyl)dihydrofuran-2(3*H*)-one (SI-7).**

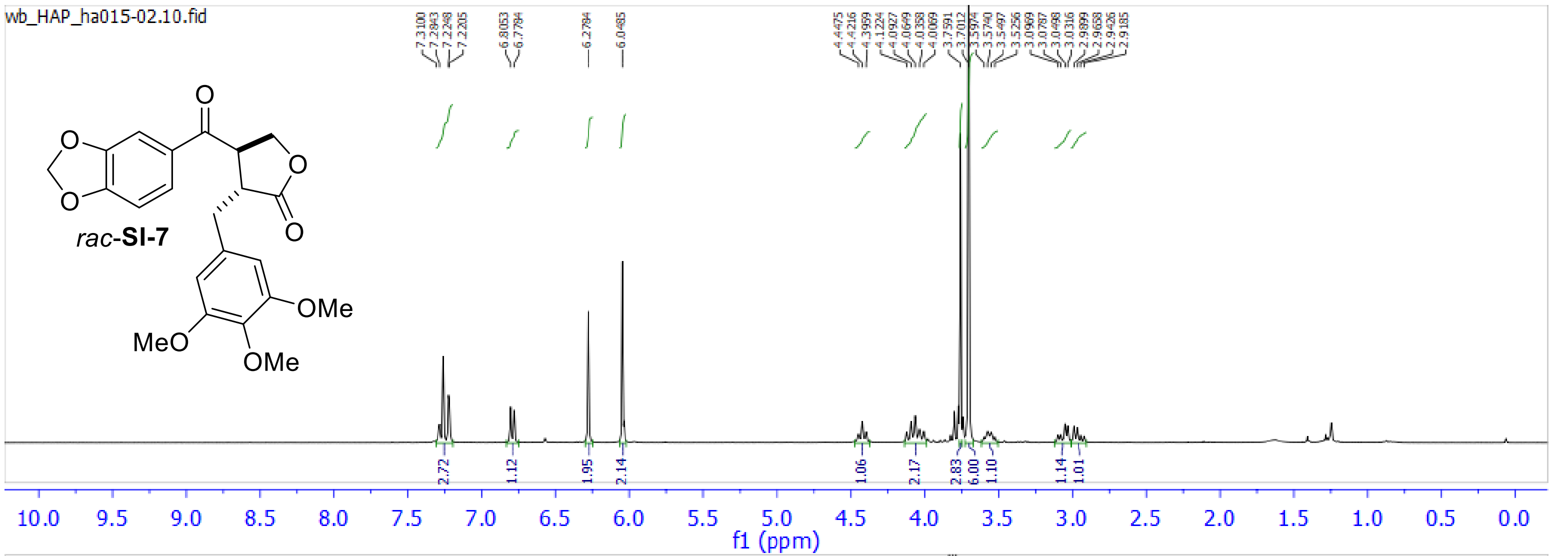

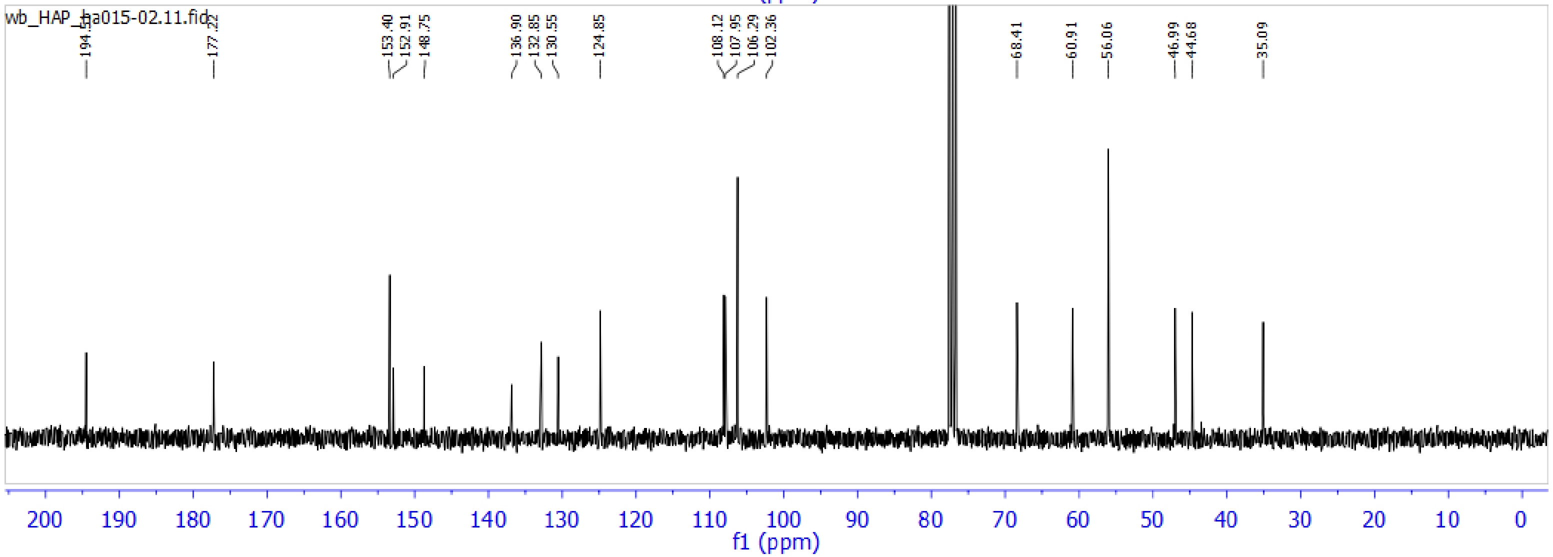

rac-benzo[d][1,3]dioxol-5-yl[5-oxo-4-(3,4,5-trimethoxybenzyl)tetrahydrofuran-3-yl]methyl acetate (SI-8).

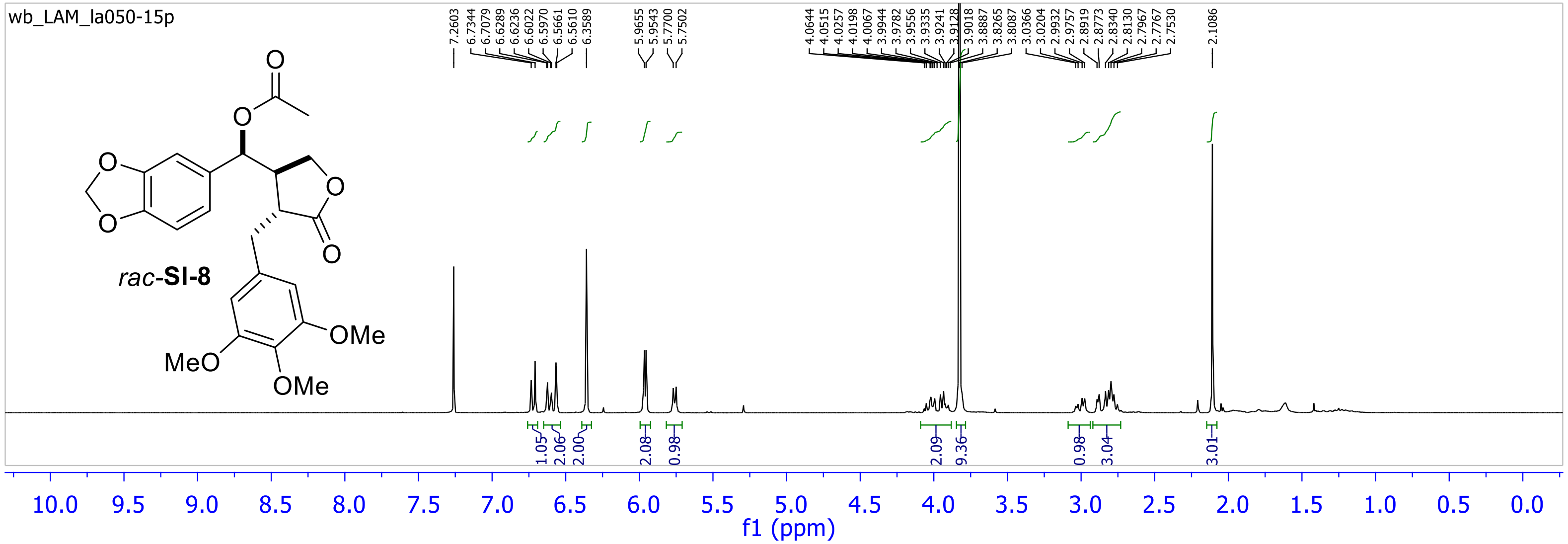

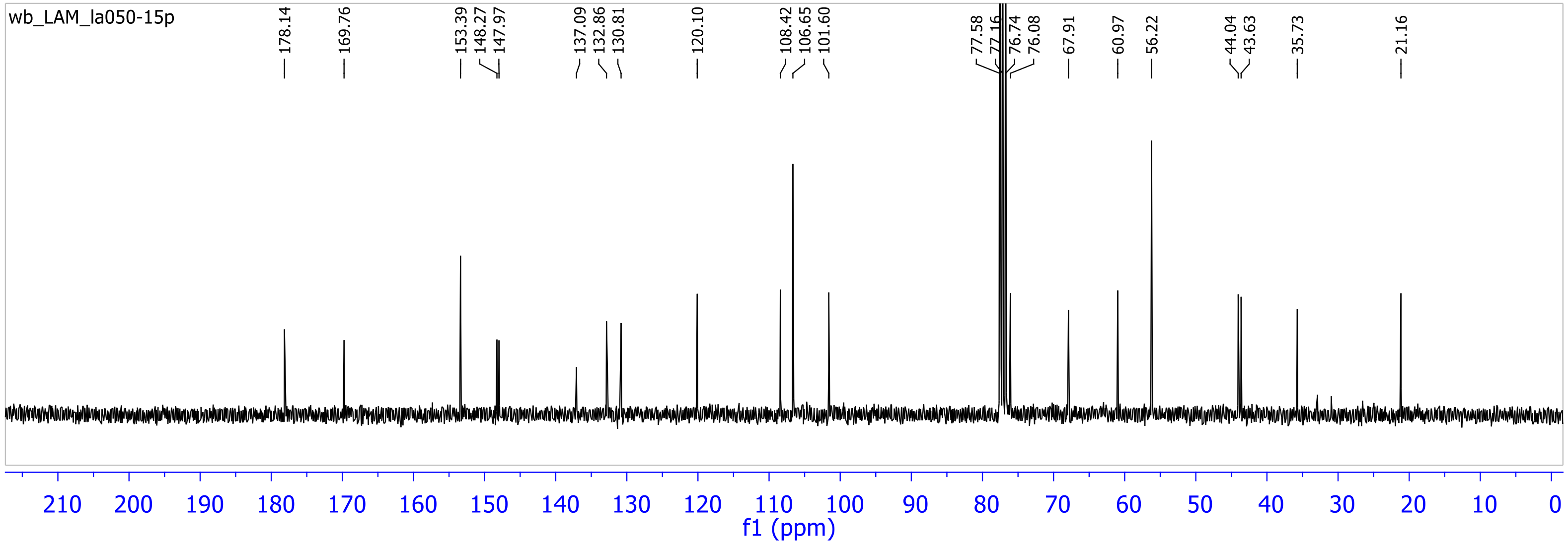

rac-4-[(-Benzo[d][1,3]dioxol-5-yl(hydroxy)methyl]-3-(3,4,5-trimethoxybenz-yl)dihydrofuran-2(3H)-one (2b).

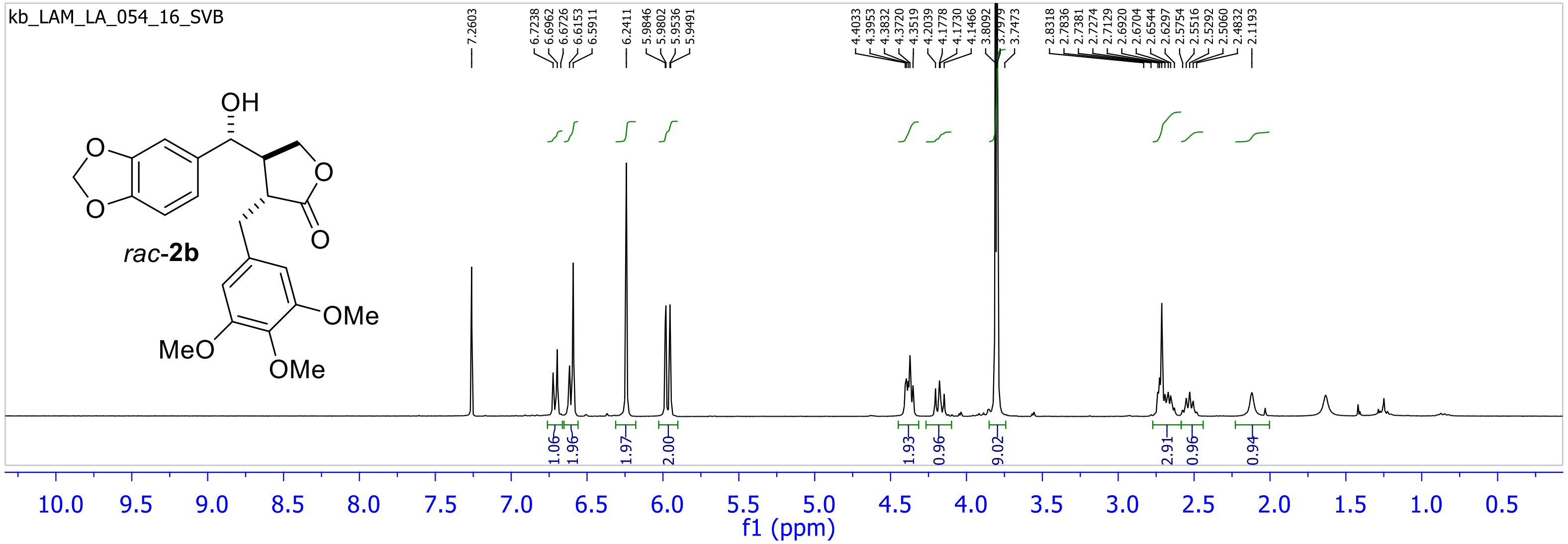

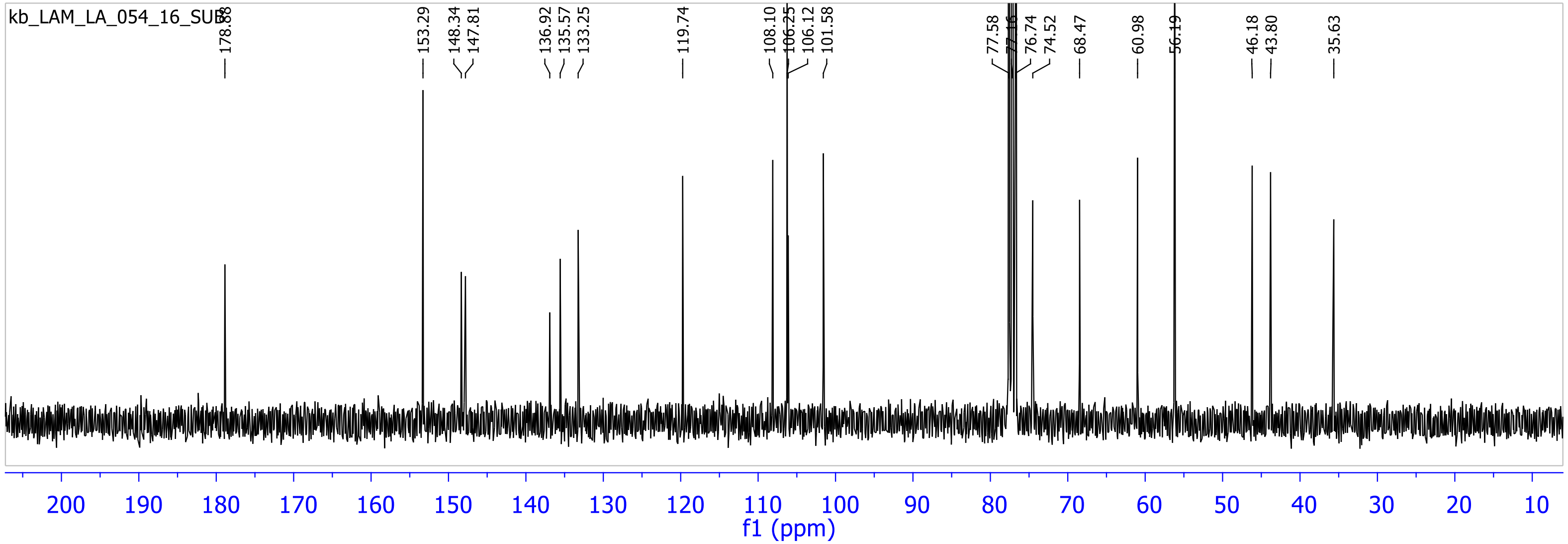

***rac*-1-(benzo[d][1,3]dioxol-5-yl)but-3-en-1-ol (SI-26).**

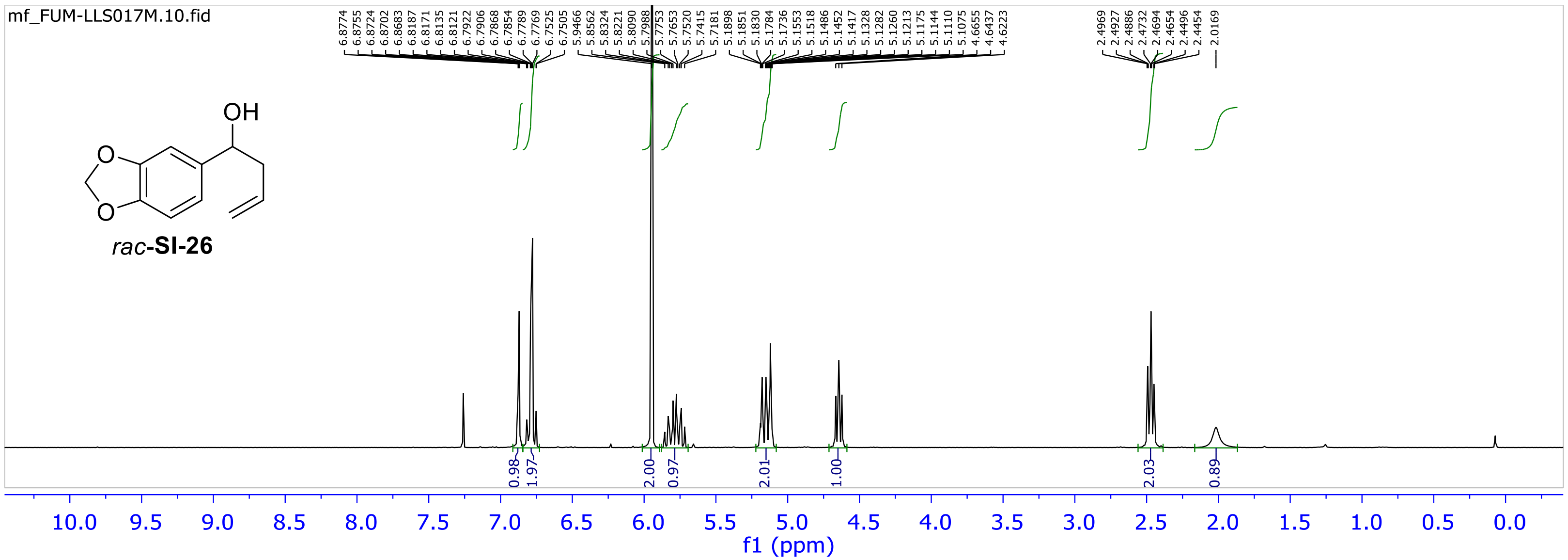

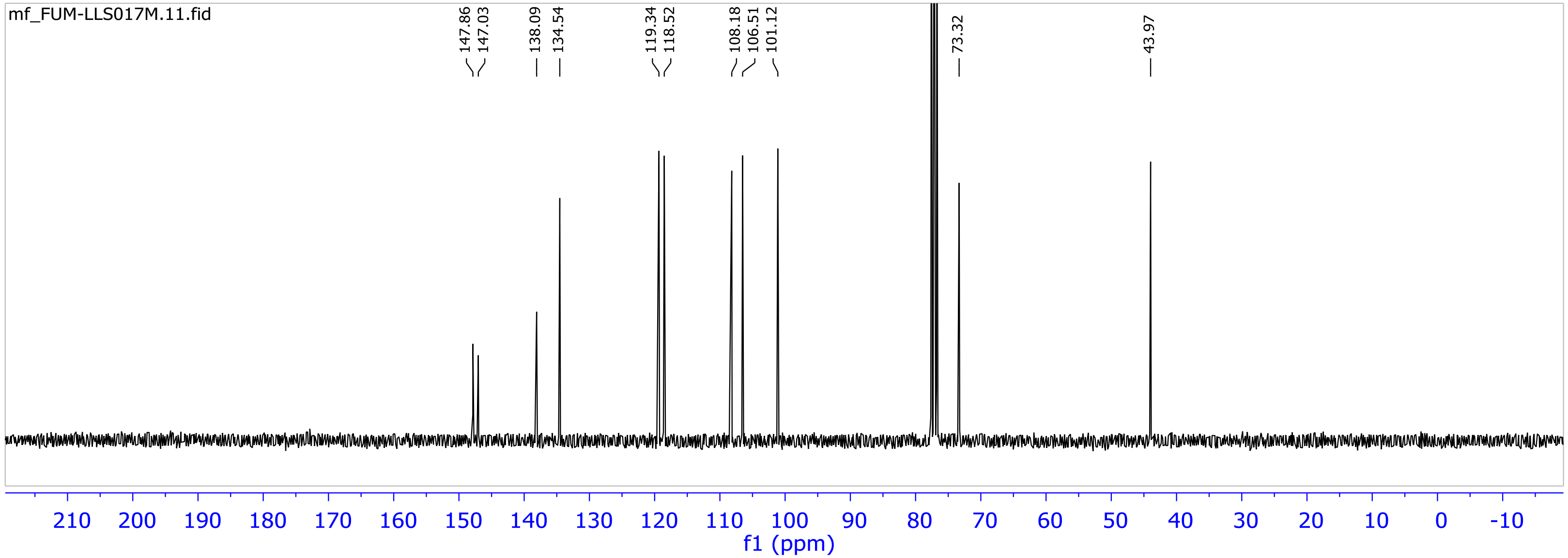

***rac*-1-(benzo[d][1,3]dioxol-5-yl)-4-(3,4,5-trimethoxyphenyl)butan-1-one (SI-27).**

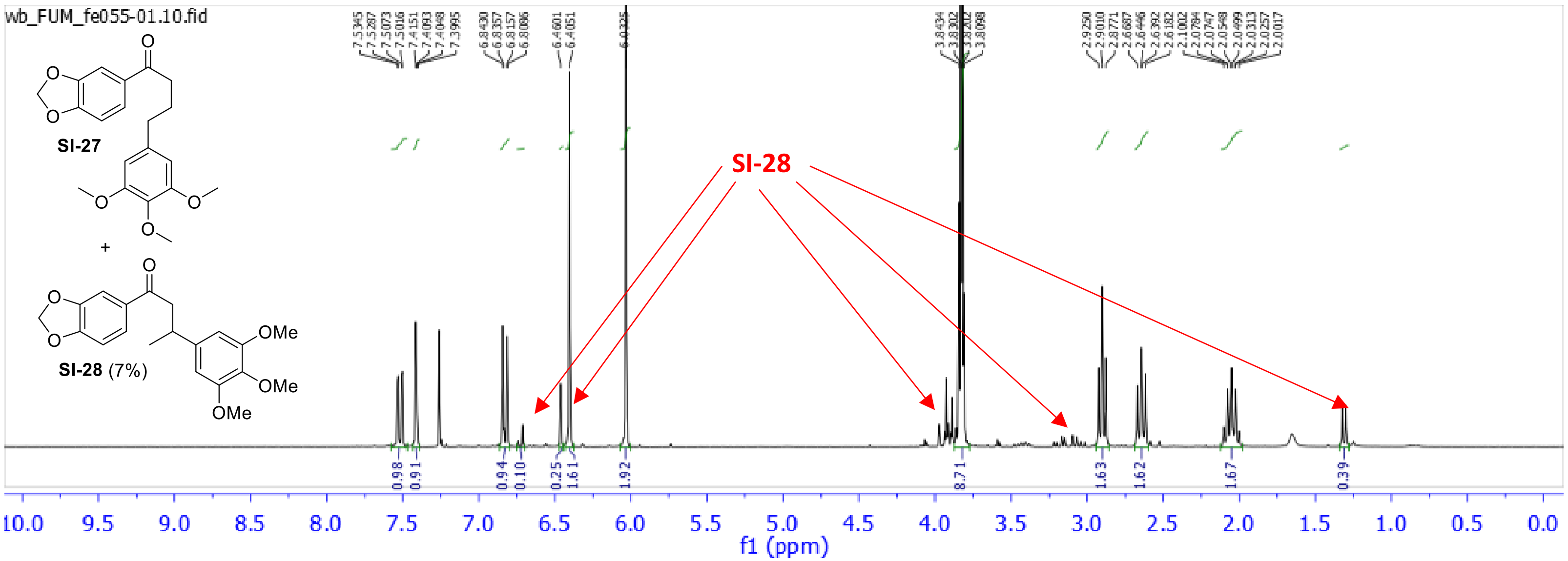

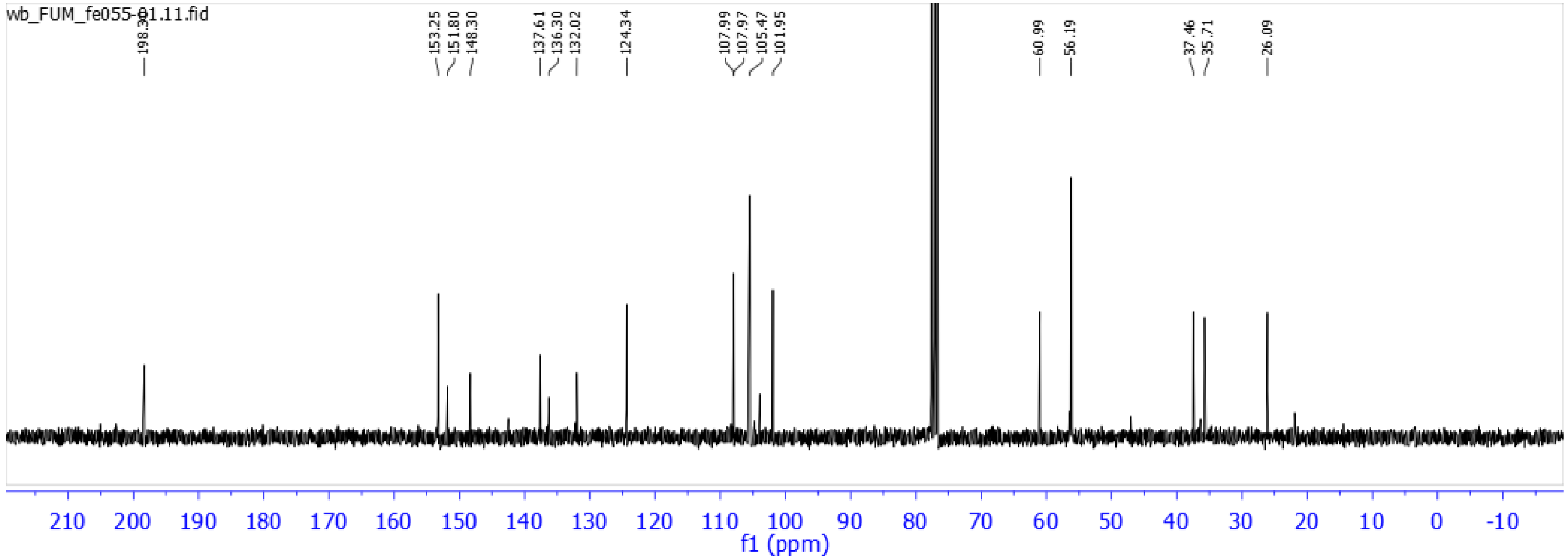

***rac*-1-(benzo[d][1,3]dioxol-5-yl)-4-(3,4,5-trimethoxyphenyl)butan-1-ol (SI-4).**

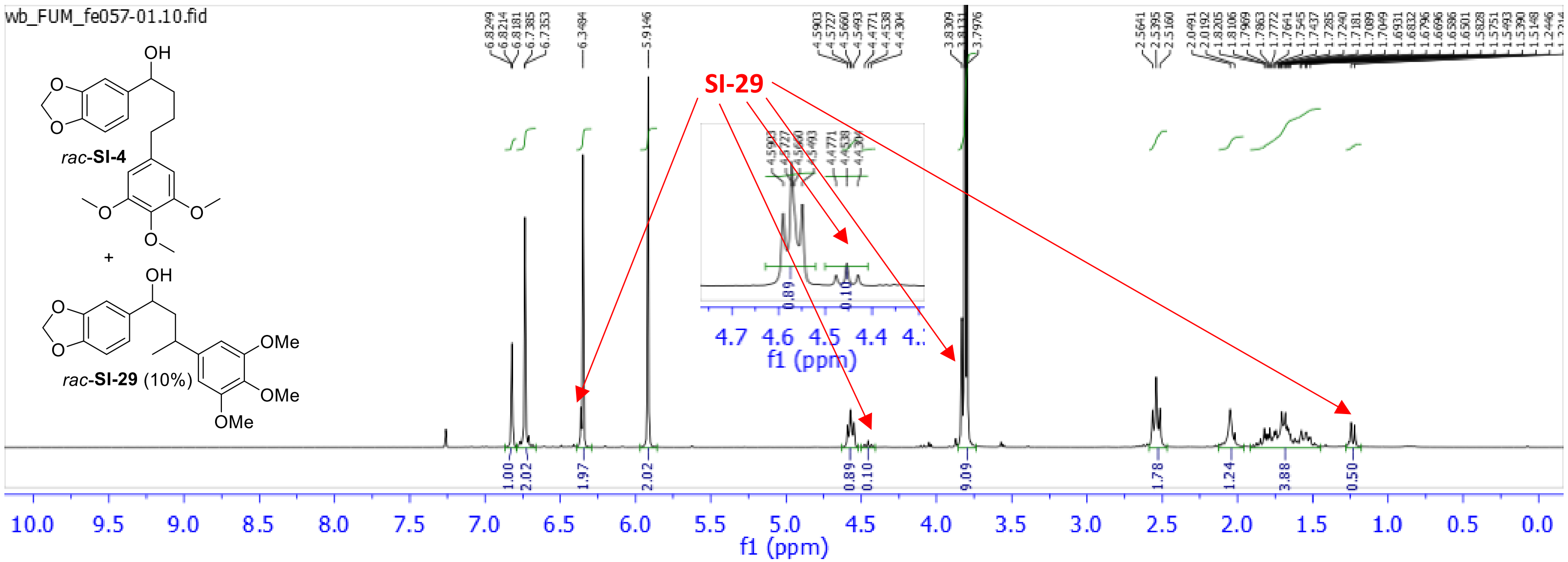

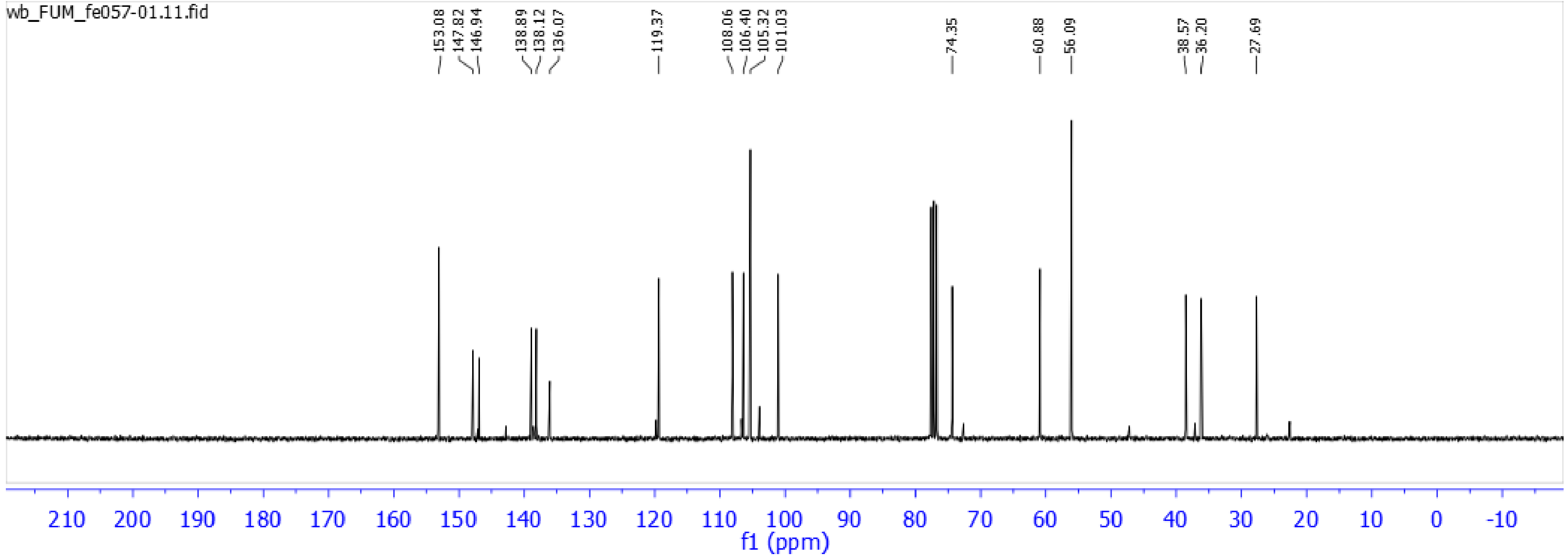

epi-Podophyllotoxine (11d).

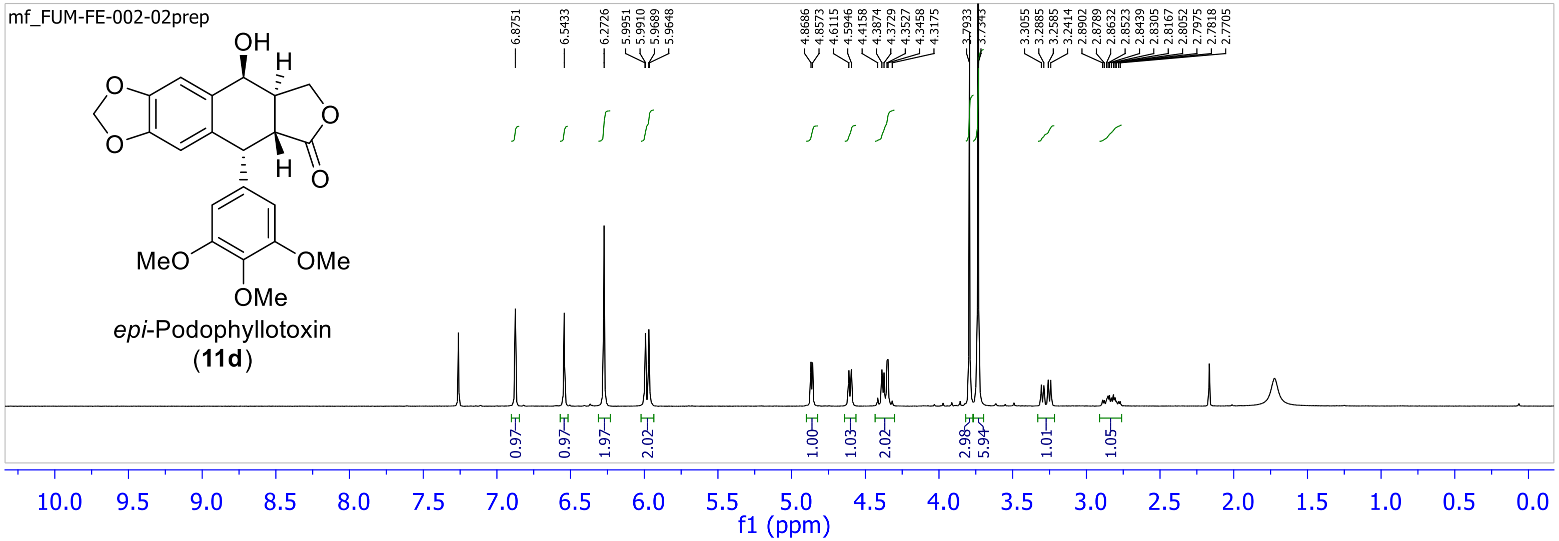

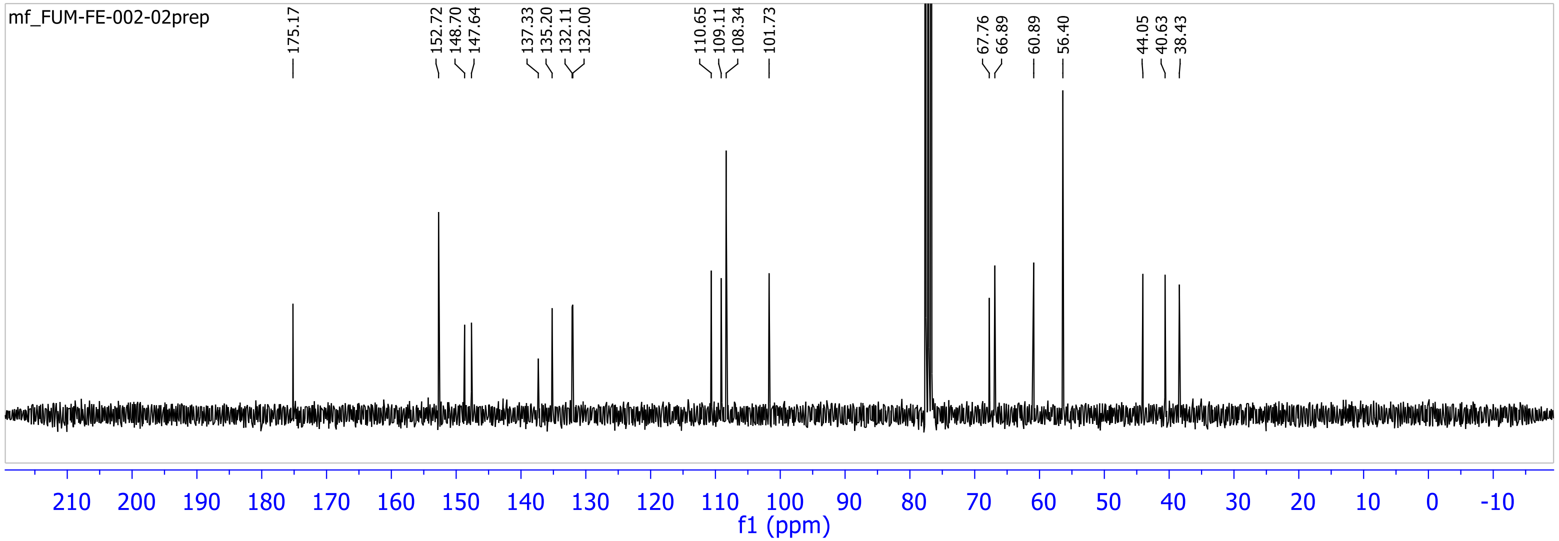

Deoxypodophyllotoxin (11a).

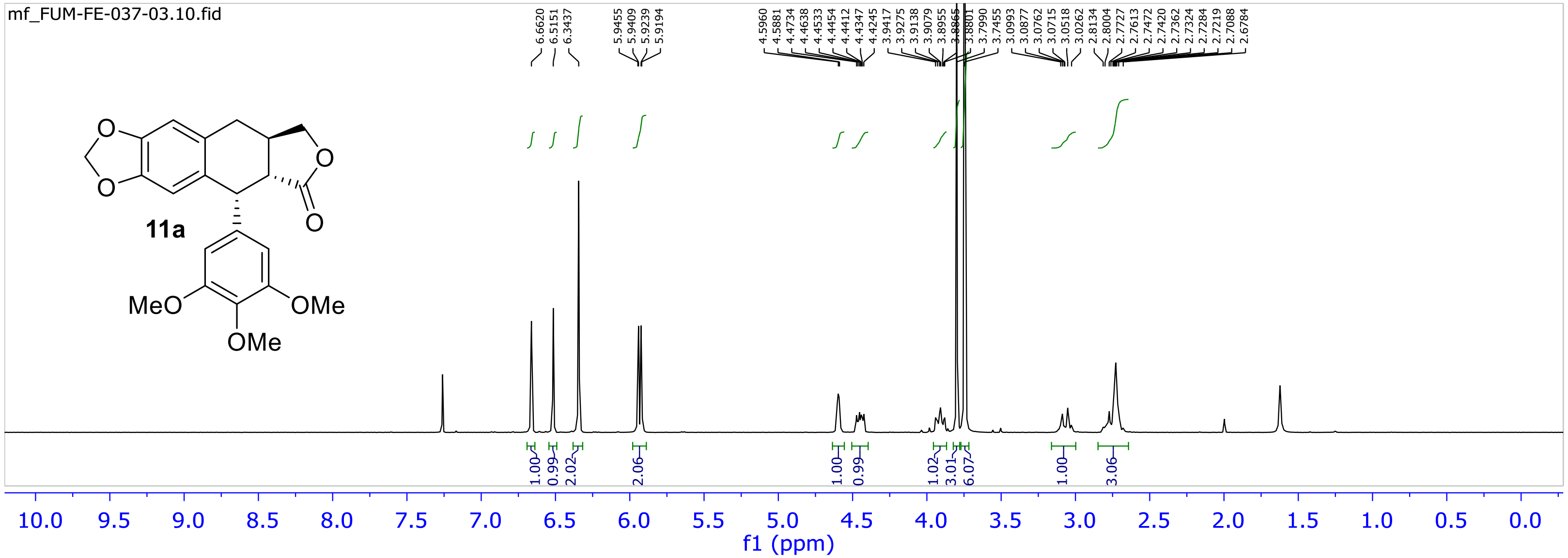

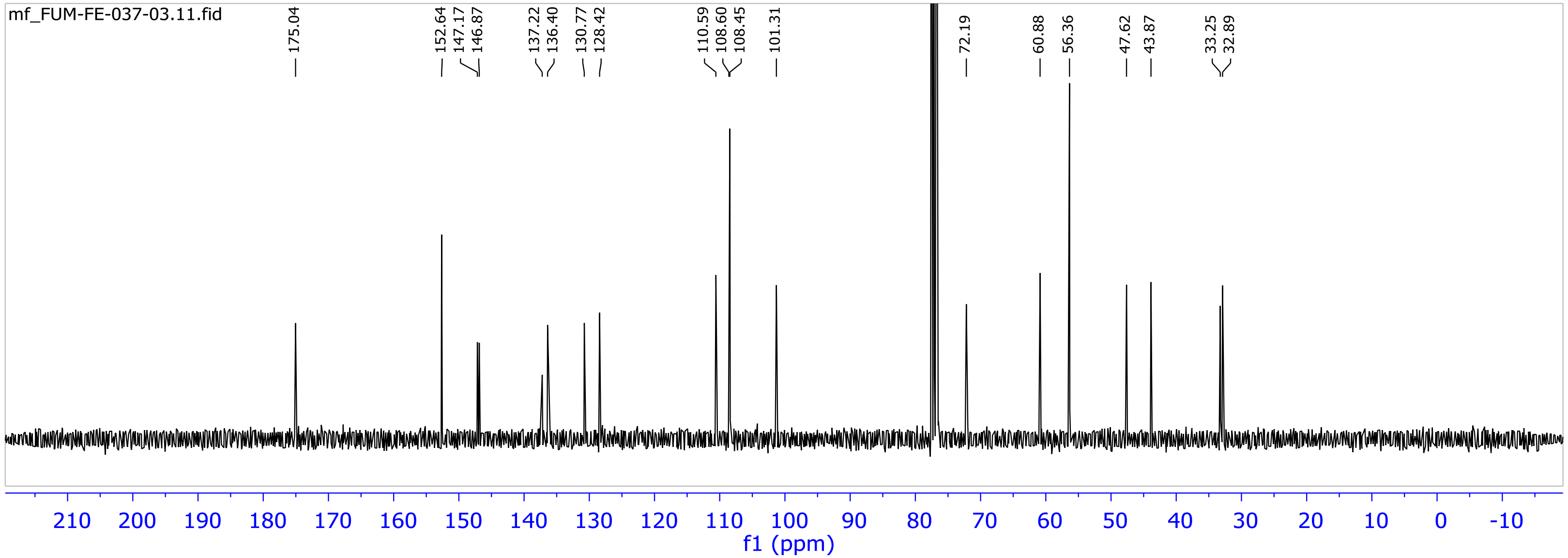

Isodeoxypodophyllotoxin (3).

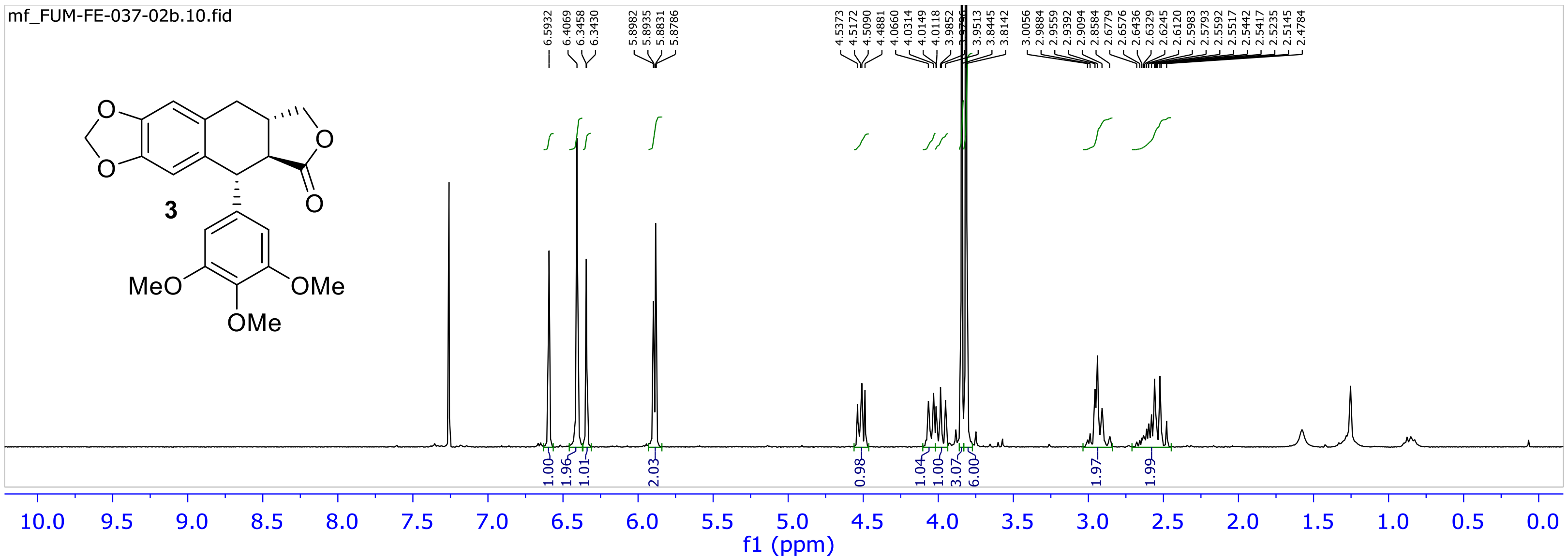

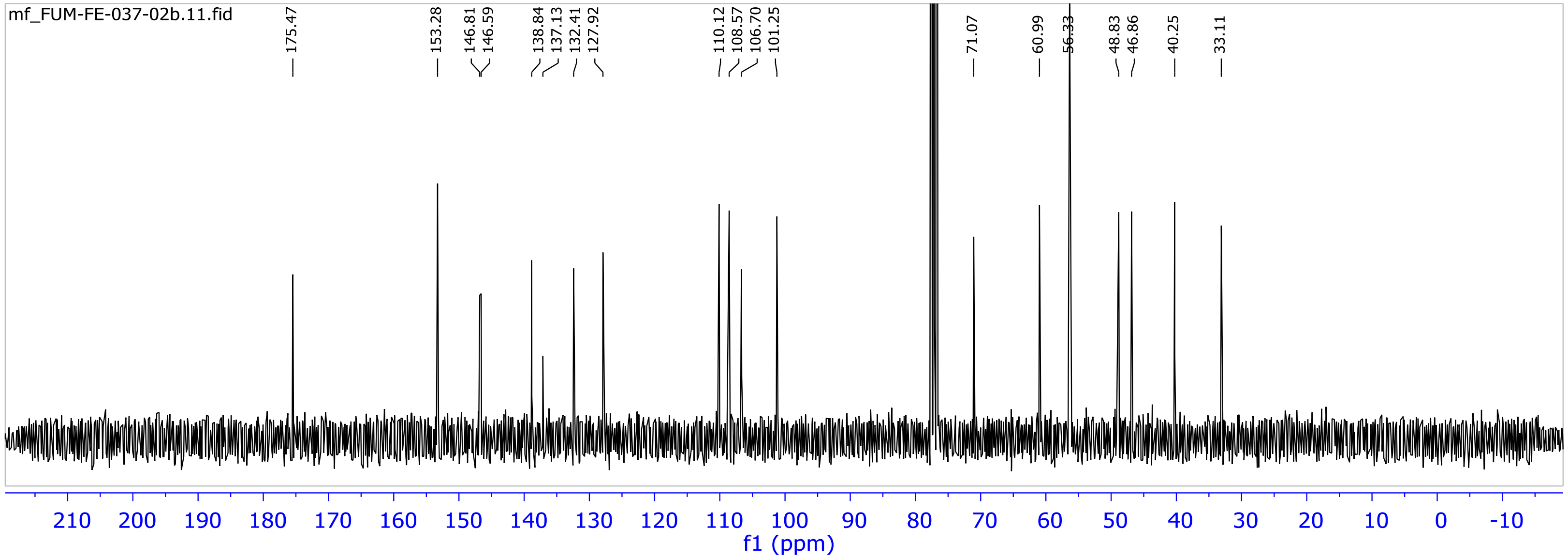

(3a*R*,4*S*,9*R*,9a*R*)-4-hydroxy-6,7-dimethoxy-9-(3,4,5-trimethoxyphenyl)-3a,4,9,9a-tetrahydronaphtho[2,3-c]furan-1(3*H*)-one (11c).

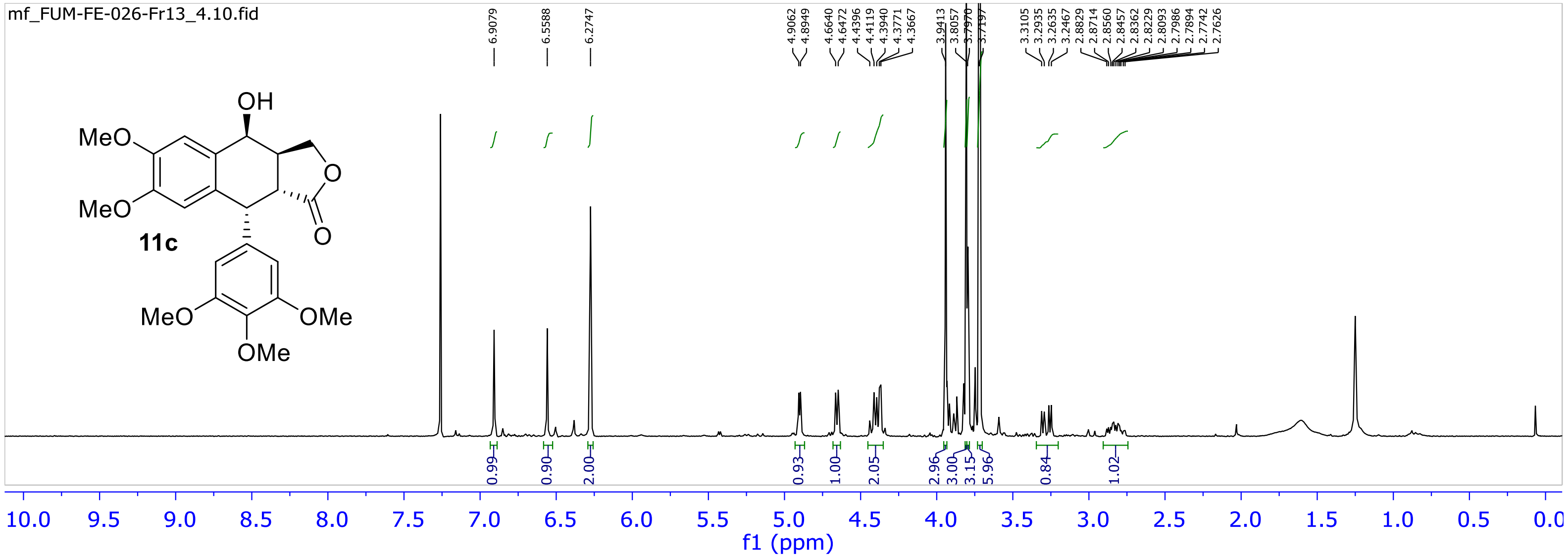

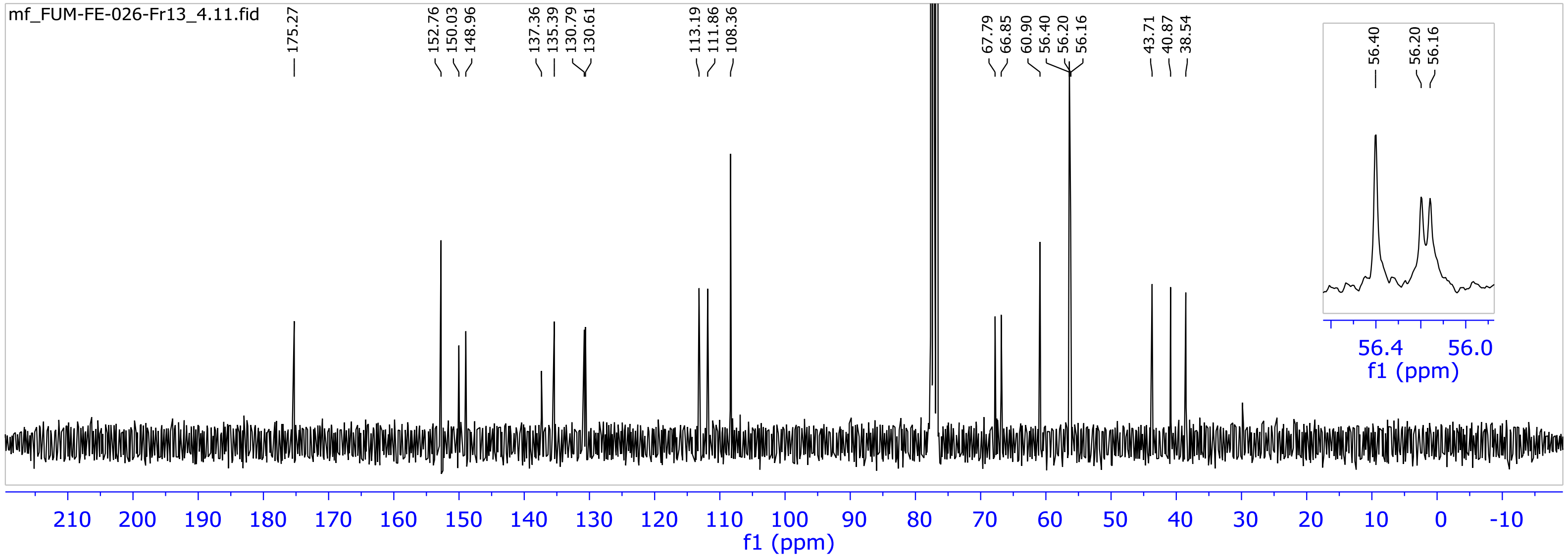

(3*S*,4*R*)-4-[(*S*)-(3,4-dimethoxyphenyl)(hydroxy)methyl]-3-[hydroxy(3,4,5-trimethoxyphenyl)methyl]dihydrofuran-2(3*H*)-one (12c).

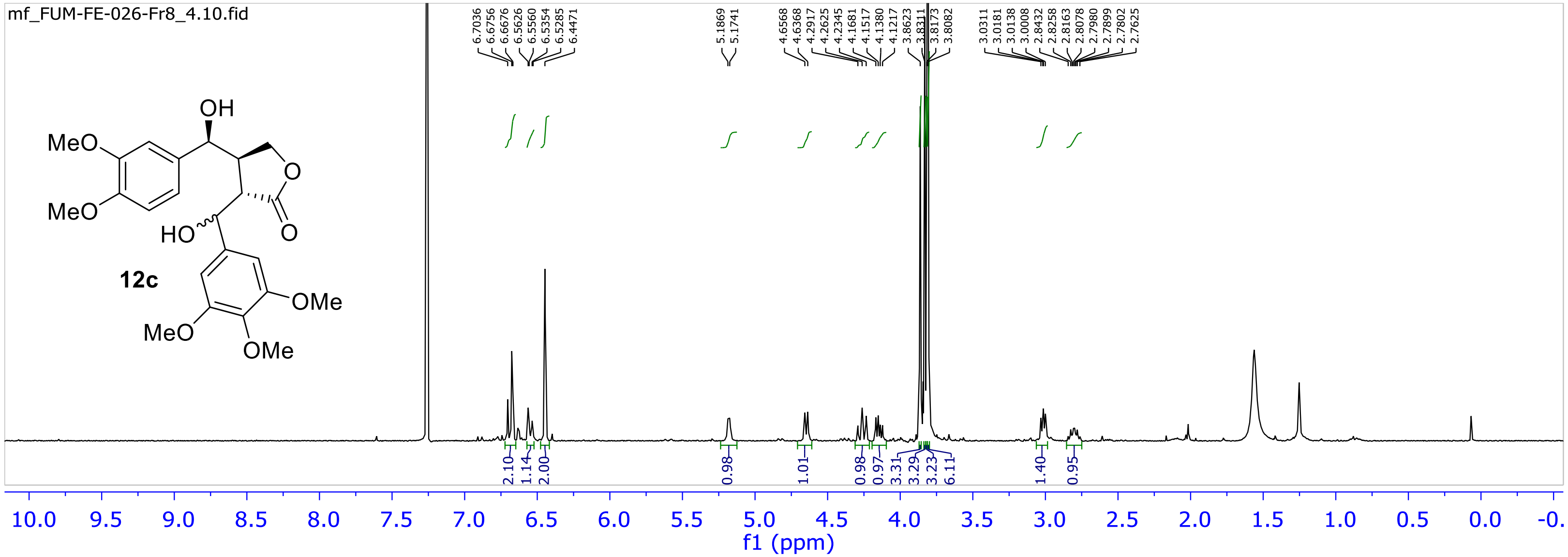

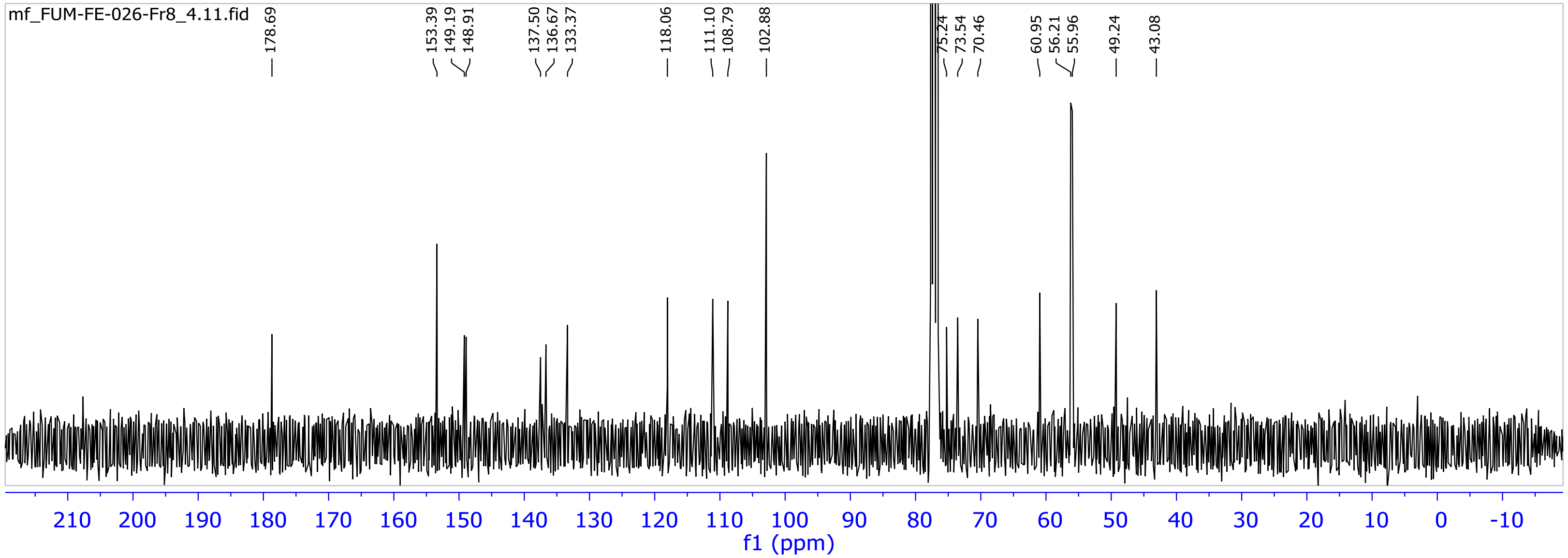

(3*S*,4*R*)-3-[Hydroxy(3,4,5-trimethoxyphenyl)methyl]-4-[(*S*)-hydroxy(phenyl)methyl]dihydrofuran-2(3*H*)-one (12e).

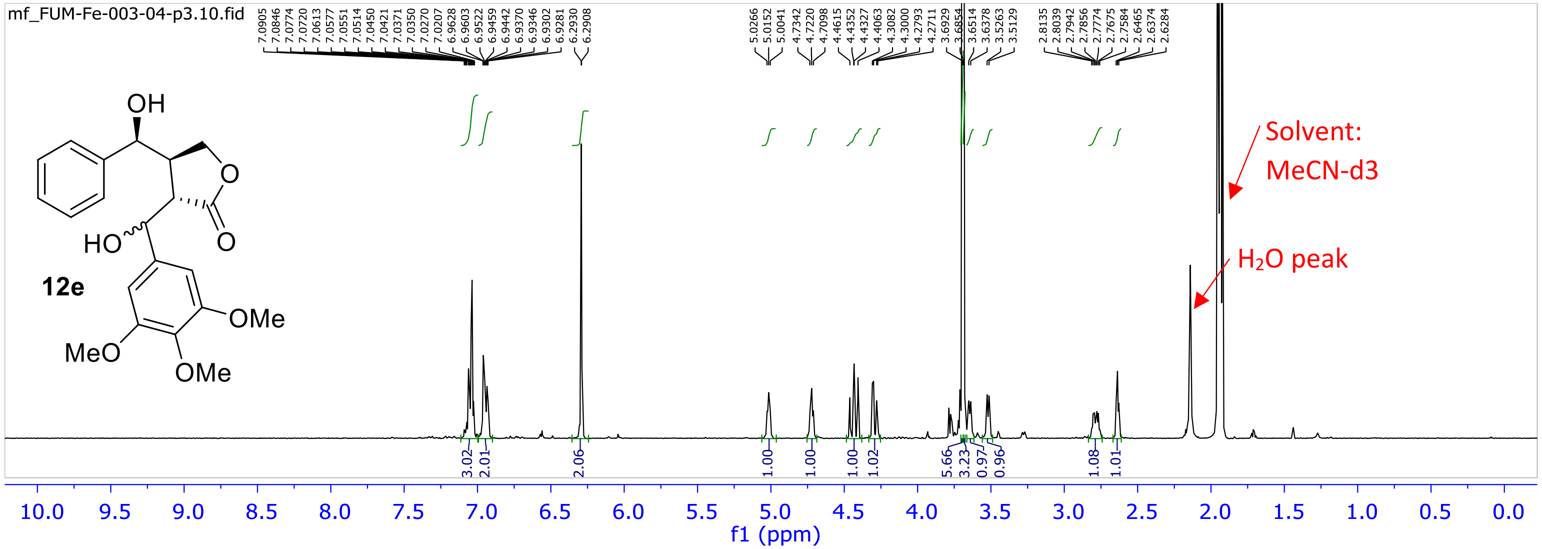

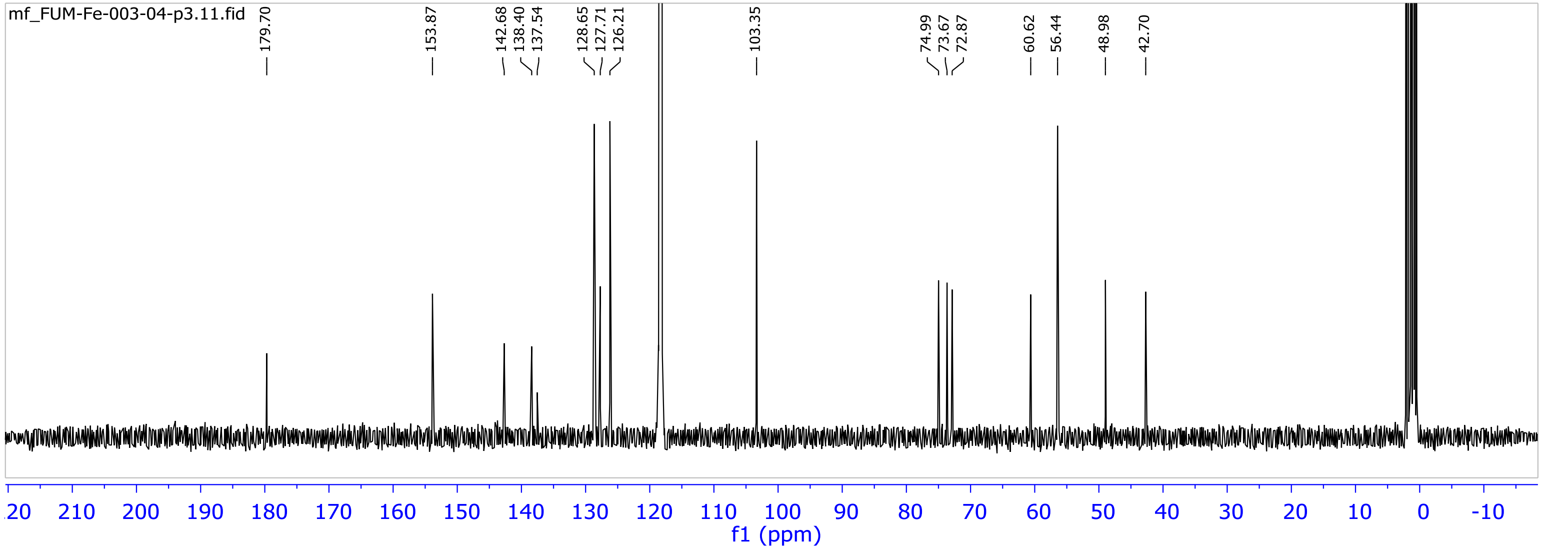

(3*S*,4*R*)-3-[Hydroxy(3,4,5-trimethoxyphenyl)methyl]-4-[(*S*)-hydroxy(naphthalen-2-yl)methyl]dihydrofuran-2(3*H*)-one (12f).

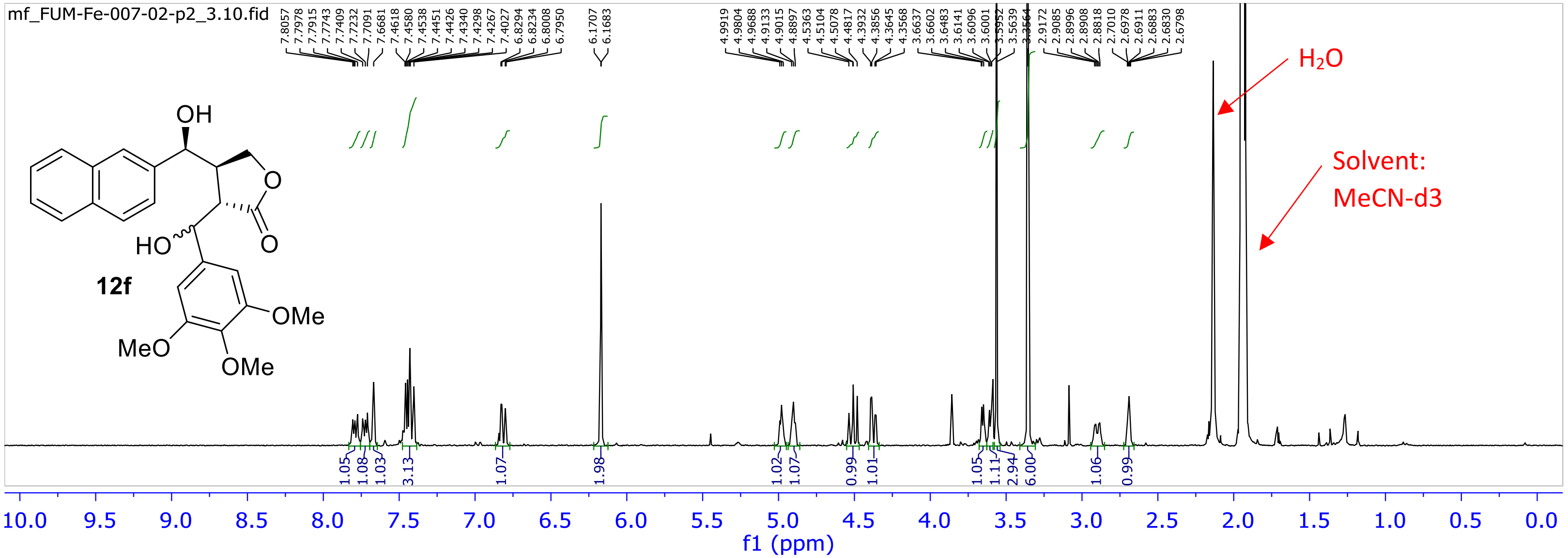

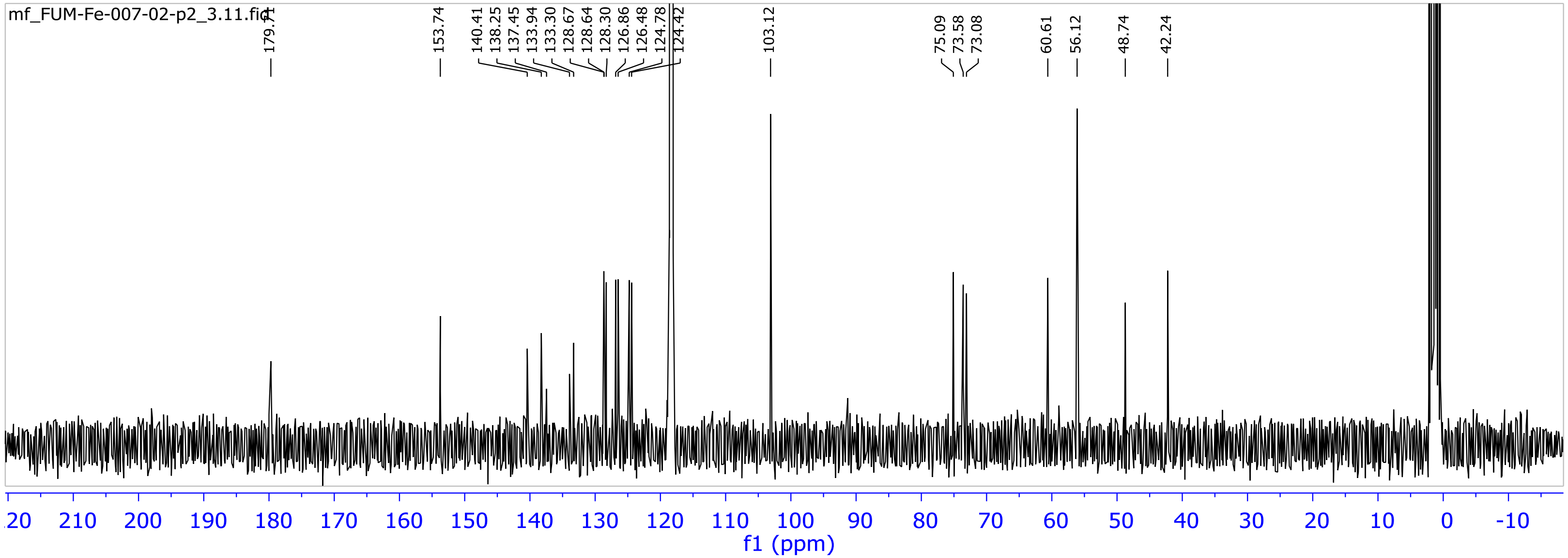

(3*S*,4*R*)-4-[(*S*)-(3-chlorophenyl)(hydroxy)methyl]-3-[hydroxy(3,4,5-trimethoxyphenyl)methyl]dihydrofuran-2(3*H*)-one (12g).

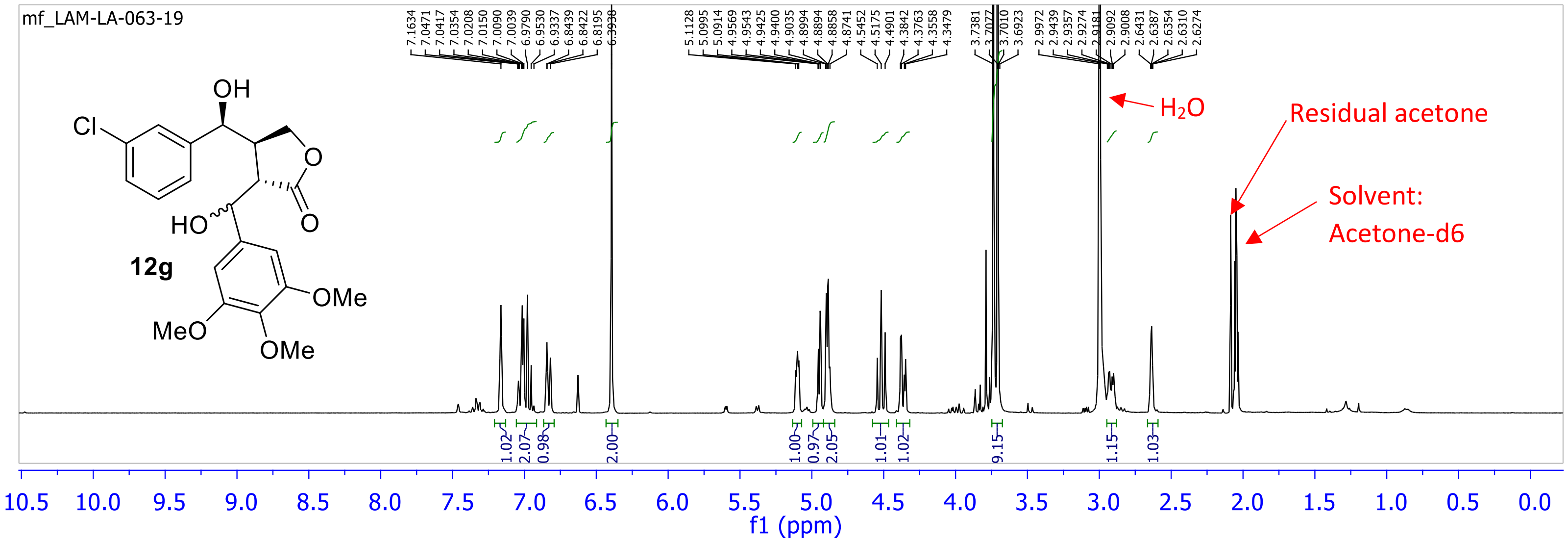

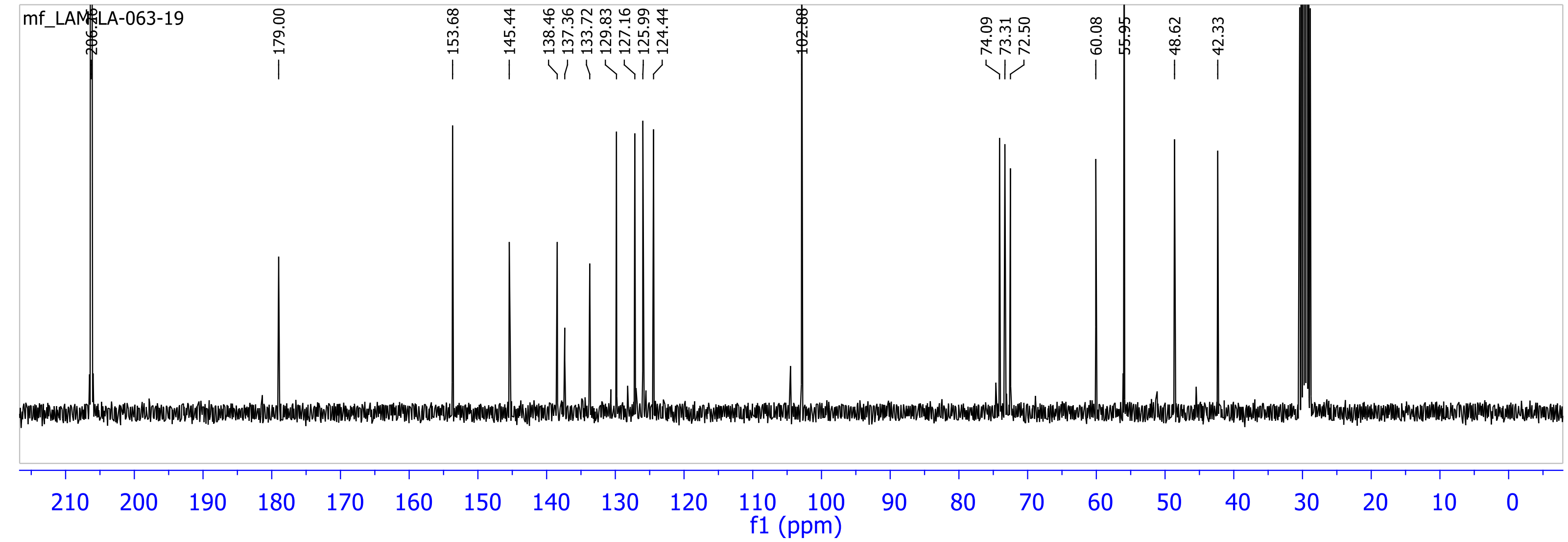

(3*S*,4*R*)-4-[(*S*)-(4-chlorophenyl)(hydroxy)methyl]-3-[hydroxy(3,4,5-trimethoxyphenyl)methyl]dihydrofuran-2(3*H*)-one (12h).

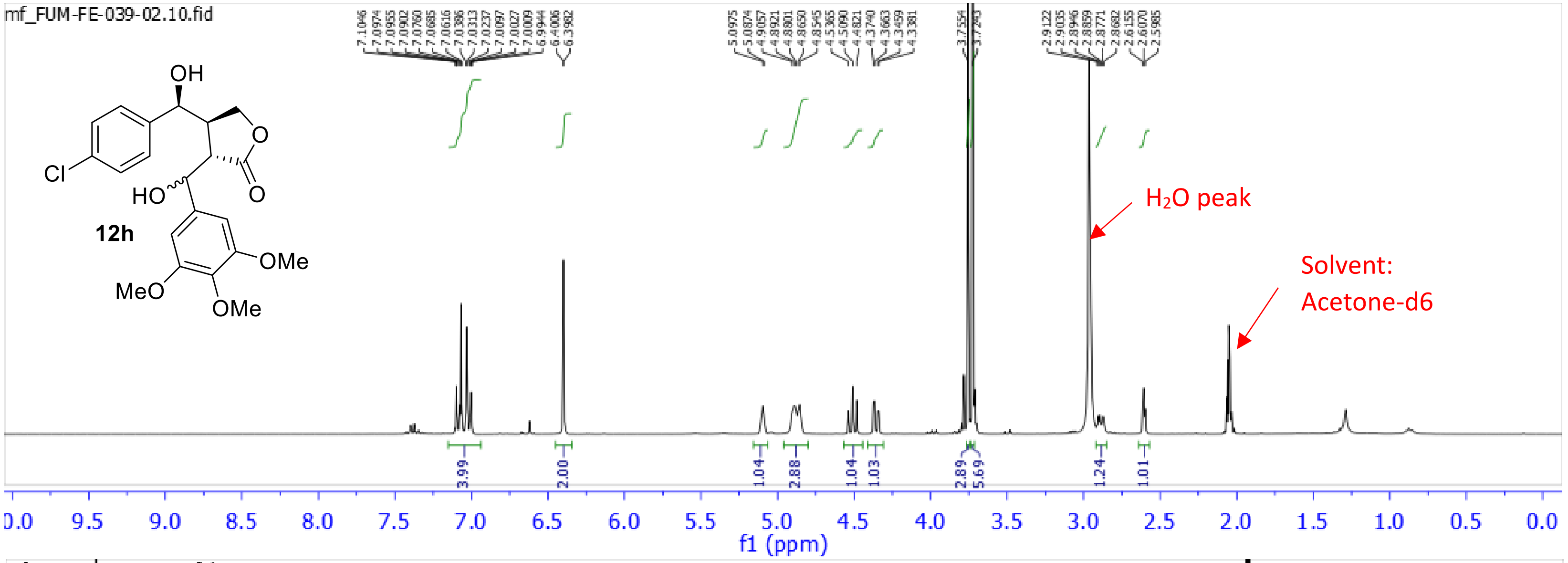

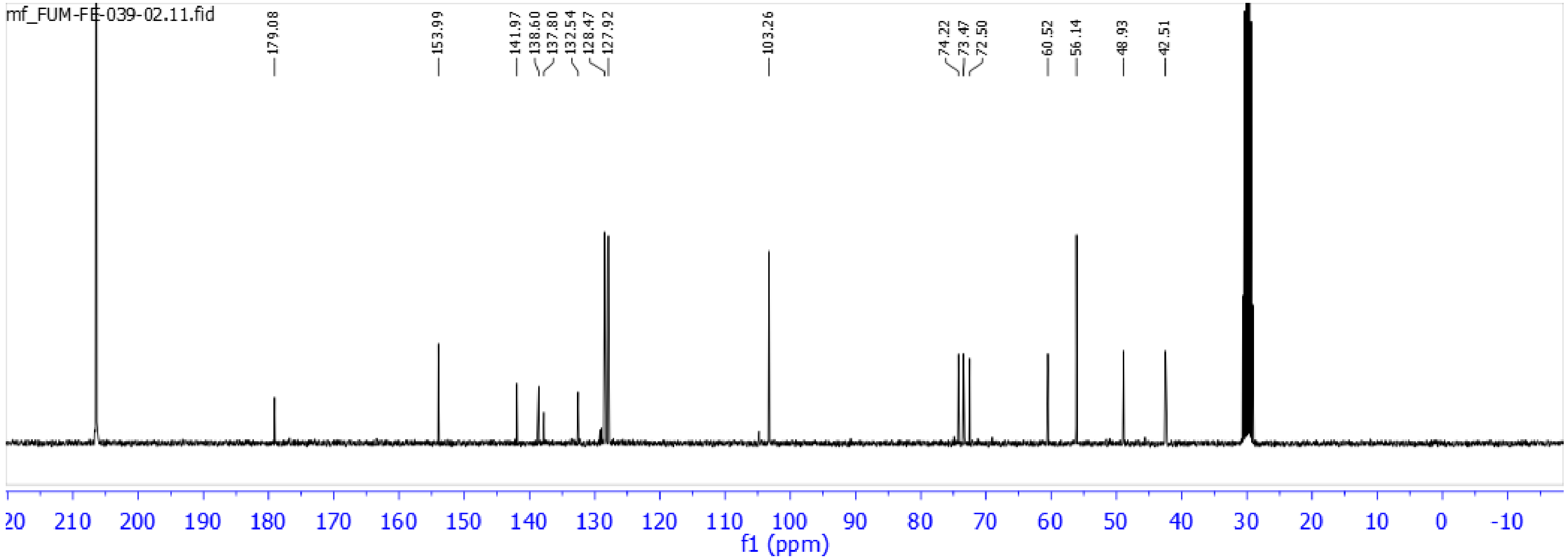

(3*S*,4*R*)-4-[(*S*)-(3-iodophenyl)(hydroxy)methyl]-3-[hydroxy(3,4,5-trimethoxyphenyl)methyl]dihydrofuran-2(3*H*)-one (12i).

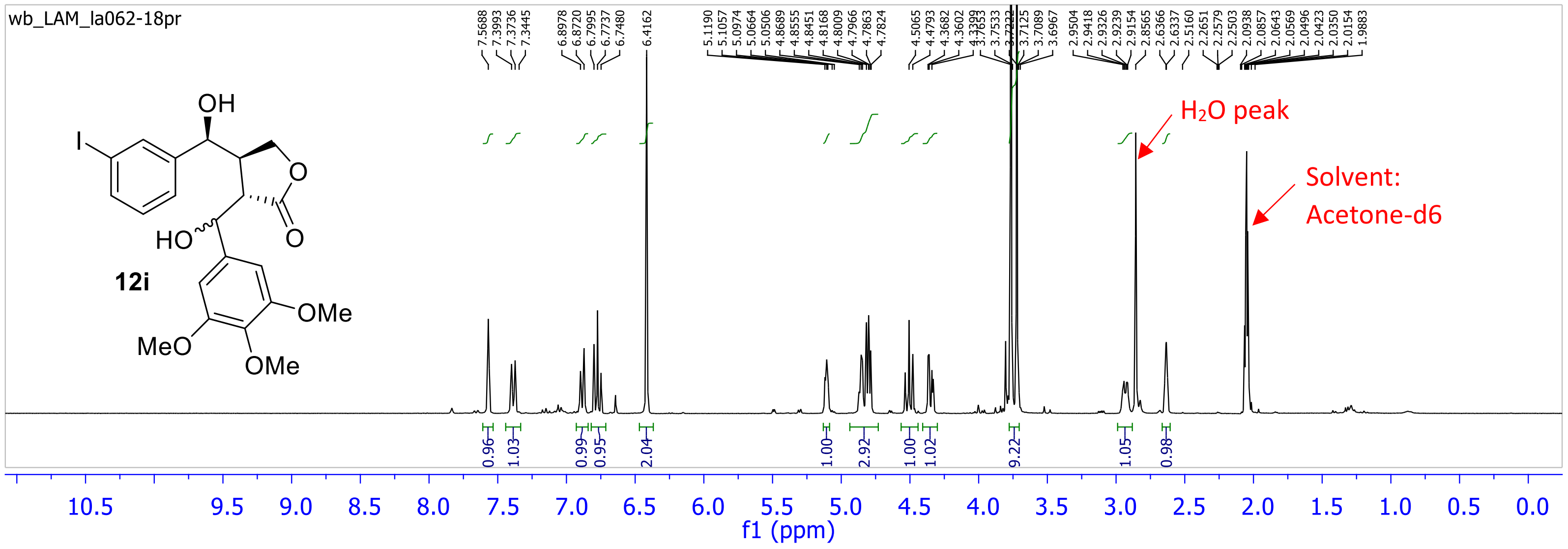

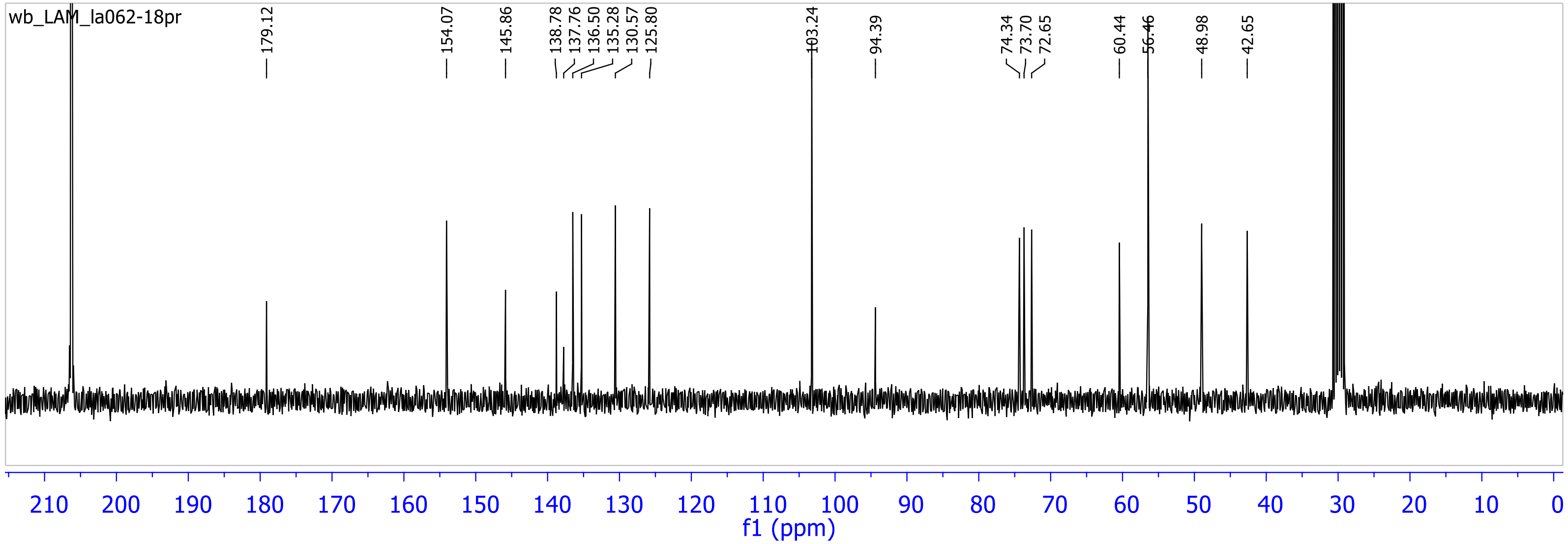

Methyl 4-((1*S*)-hydroxy{(3*R*,4*S*)-4-[hydroxy(3,4,5-trimethoxyphenyl)methyl]-5-oxotetrahydrofuran-3-yl}methyl)benzoate (12j).

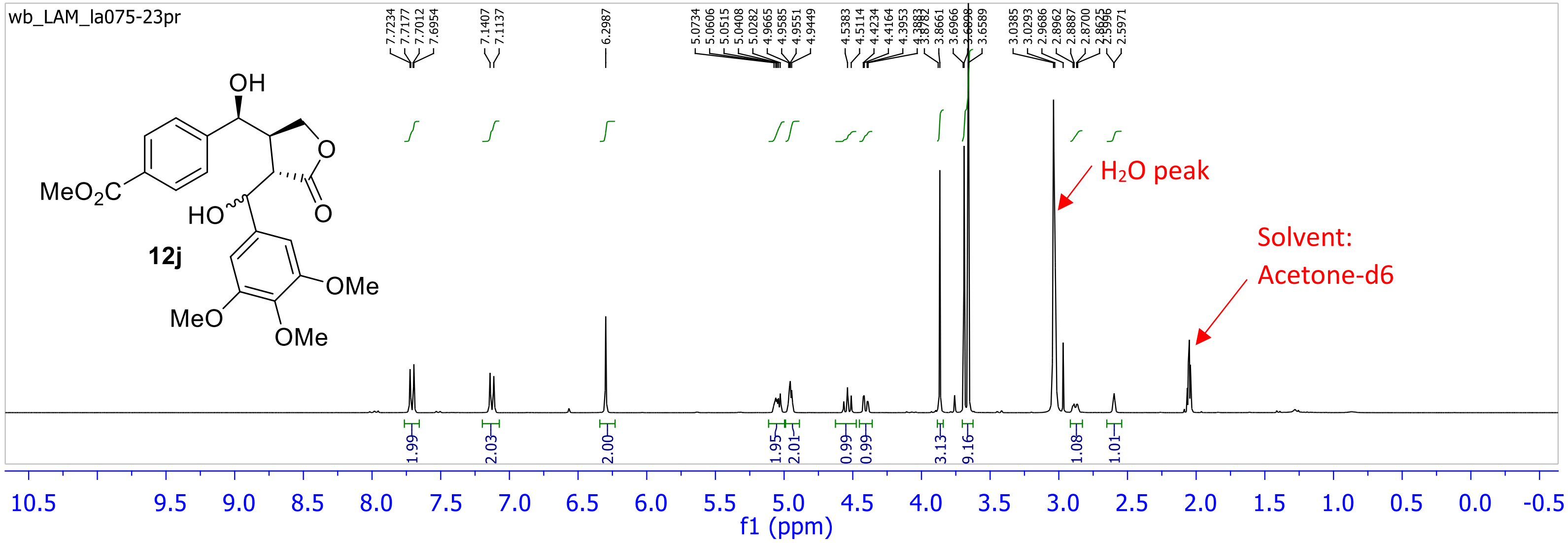

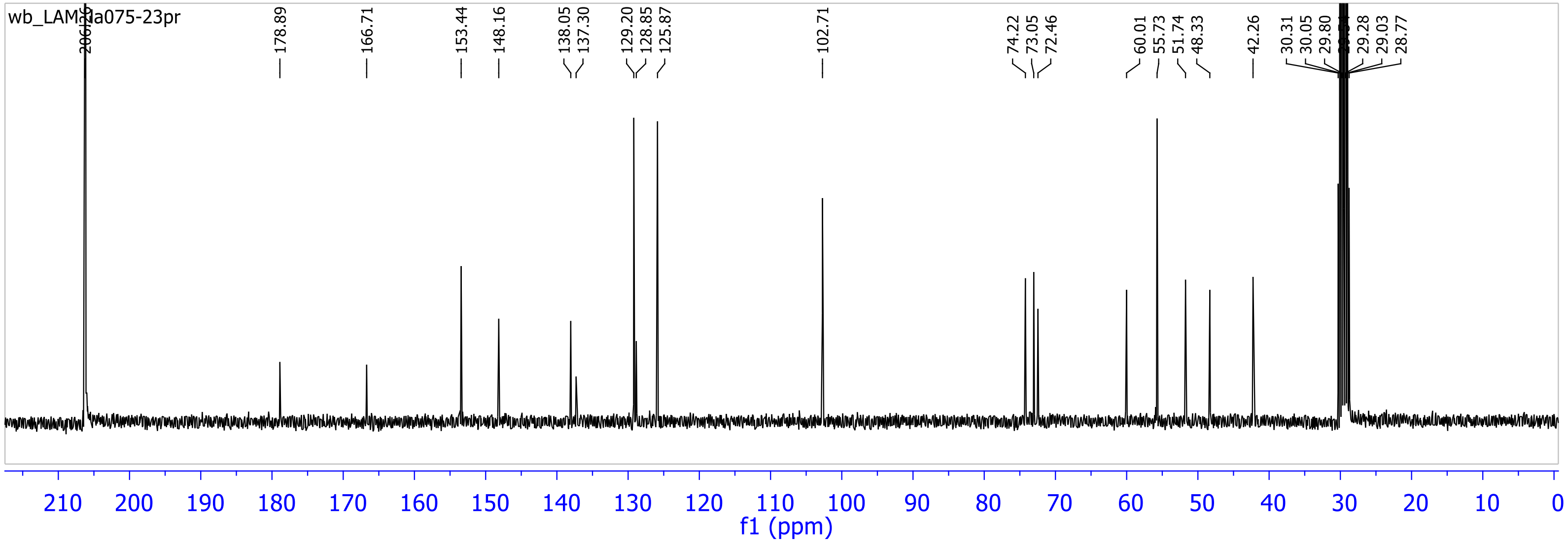

(3*S*,4*R*)-3-[hydroxy(3,4,5-trimethoxyphenyl)methyl]-4-[(*S*)-hydroxy(4-methoxyphenyl)methyl]dihydrofuran-2(3*H*)-one (12k).

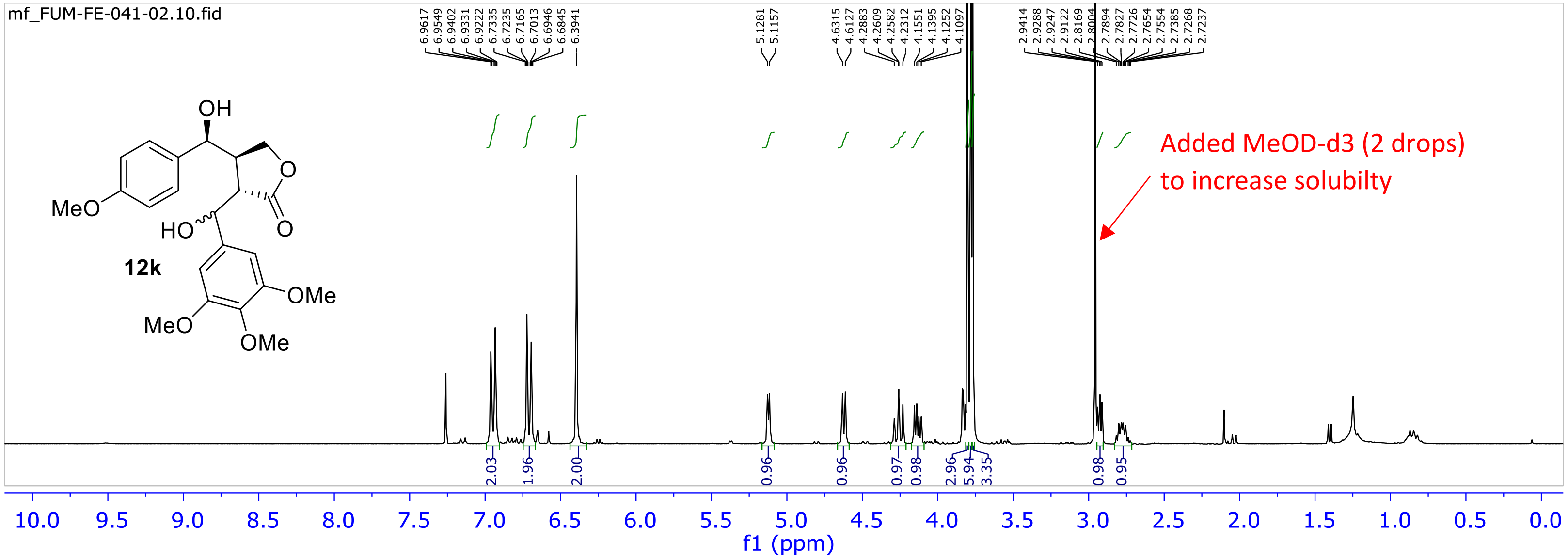

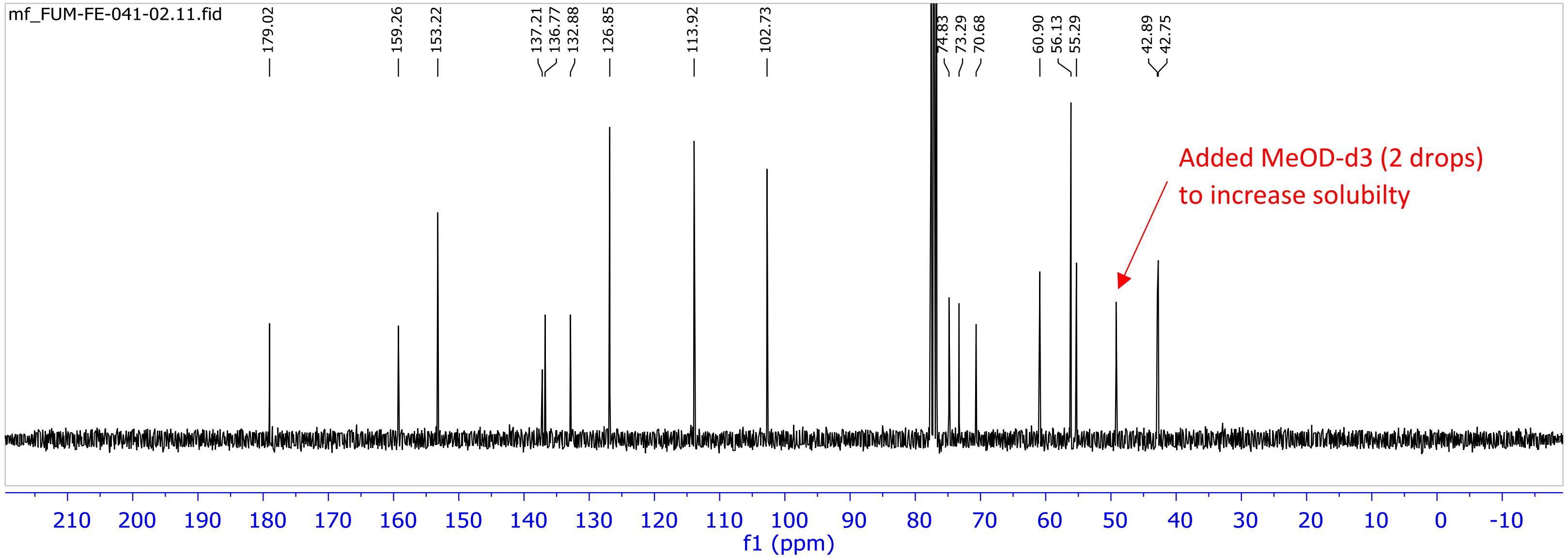

(3*S*,4*R*)-3-[hydroxy(3,4,5-trimethoxyphenyl)methyl]-4-[(*S*)-hydroxy(3-methoxyphenyl)methyl]dihydrofuran-2(3*H*)-one (11l).

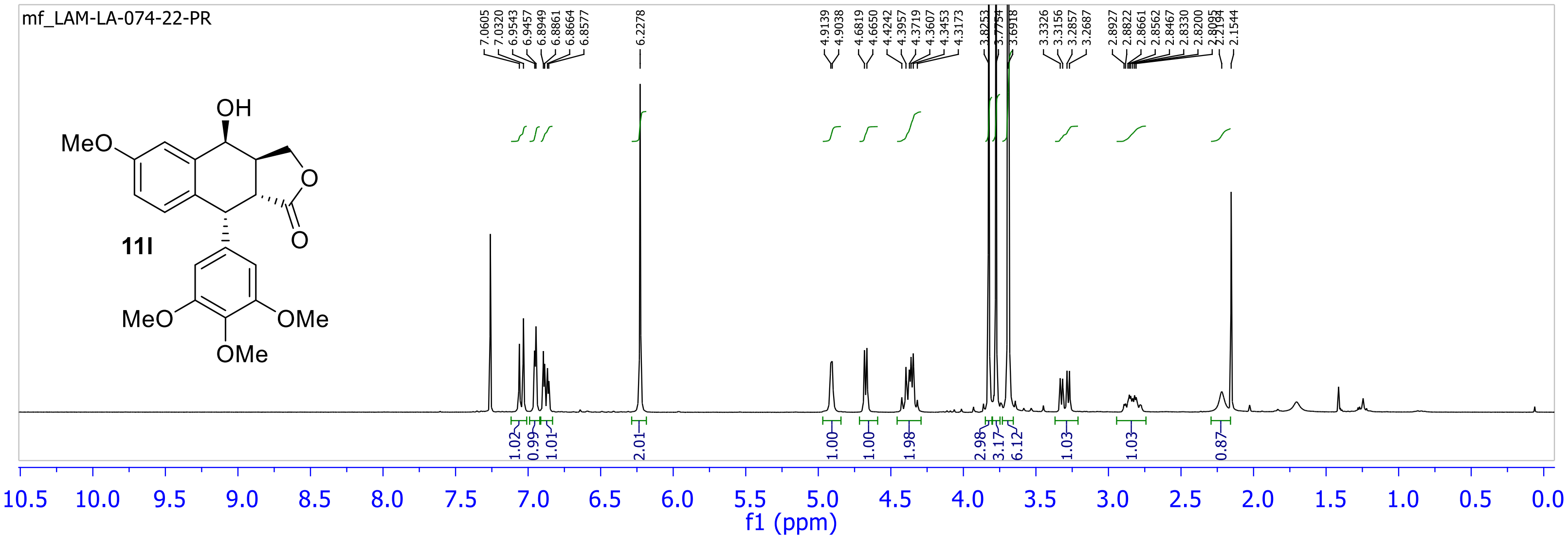

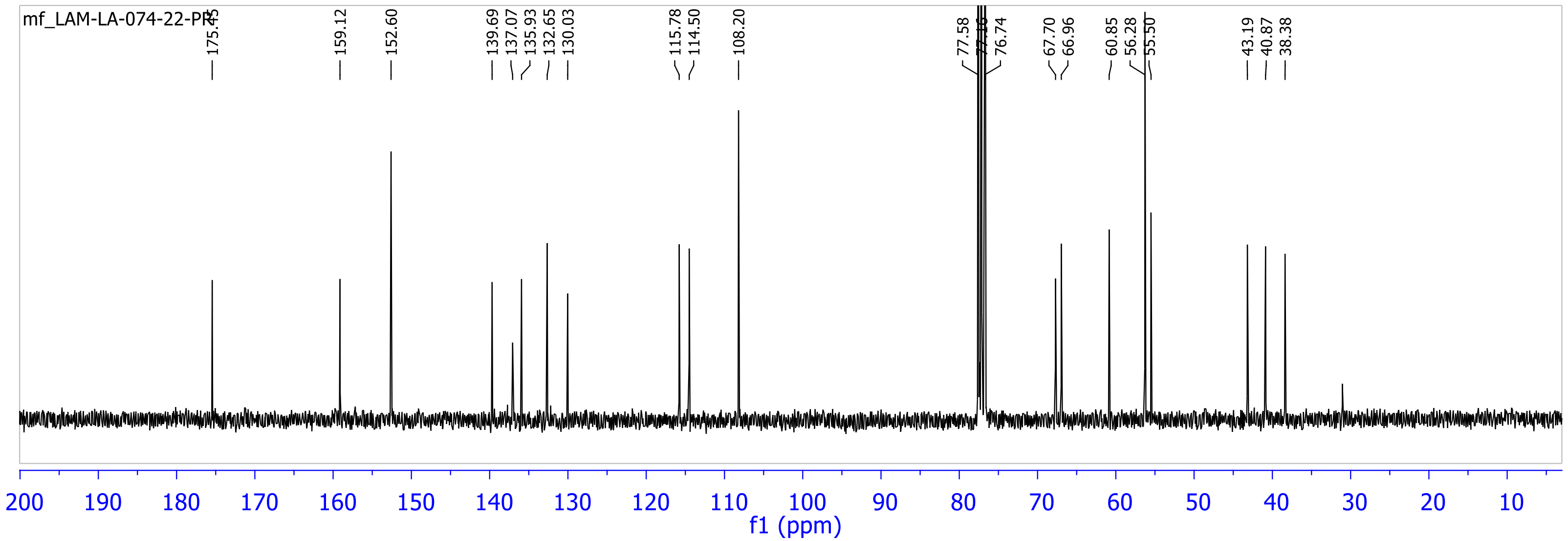

(3*S*,4*R*)-3-[hydroxy(3,4,5-trimethoxyphenyl)methyl]-4-[(*S*)-hydroxy(3-methoxyphenyl)methyl]dihydrofuran-2(3*H*)-one (12l).

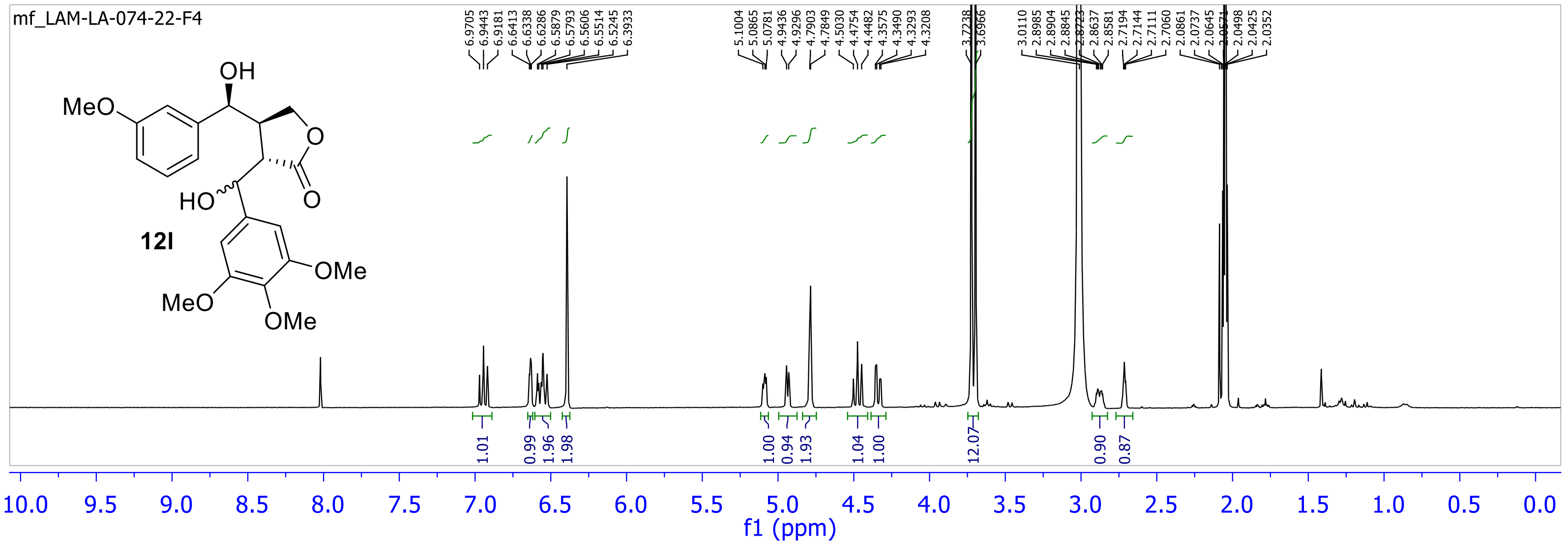

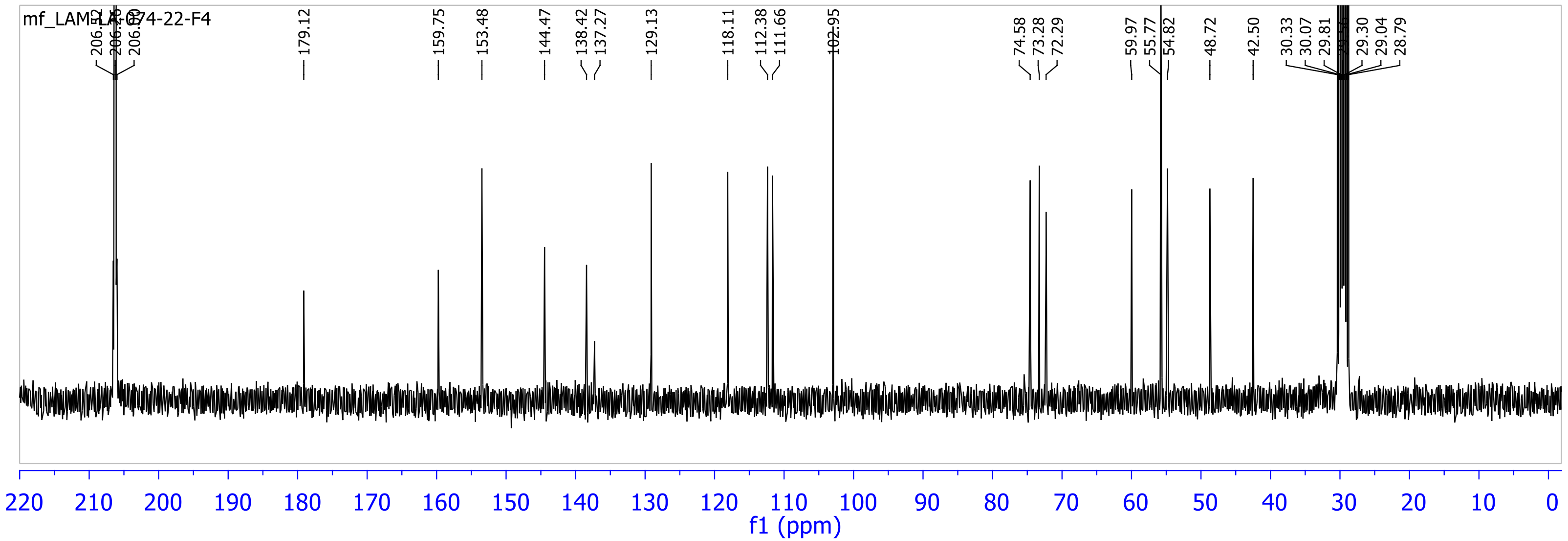

(3*S*,4*R*)-4-[(*S*)-(4-fluorophenyl)(hydroxy)methyl]-3-[hydroxy(3,4,5-trimethoxyphenyl)methyl]dihydrofuran-2(3*H*)-one (12m).

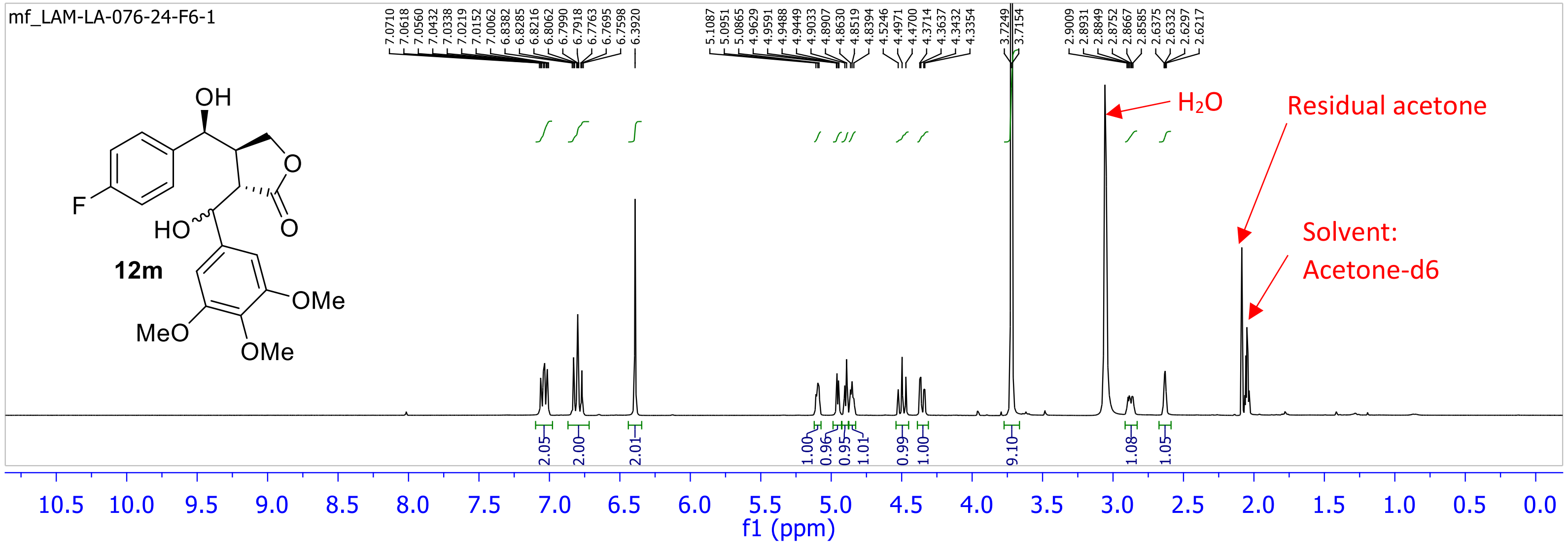

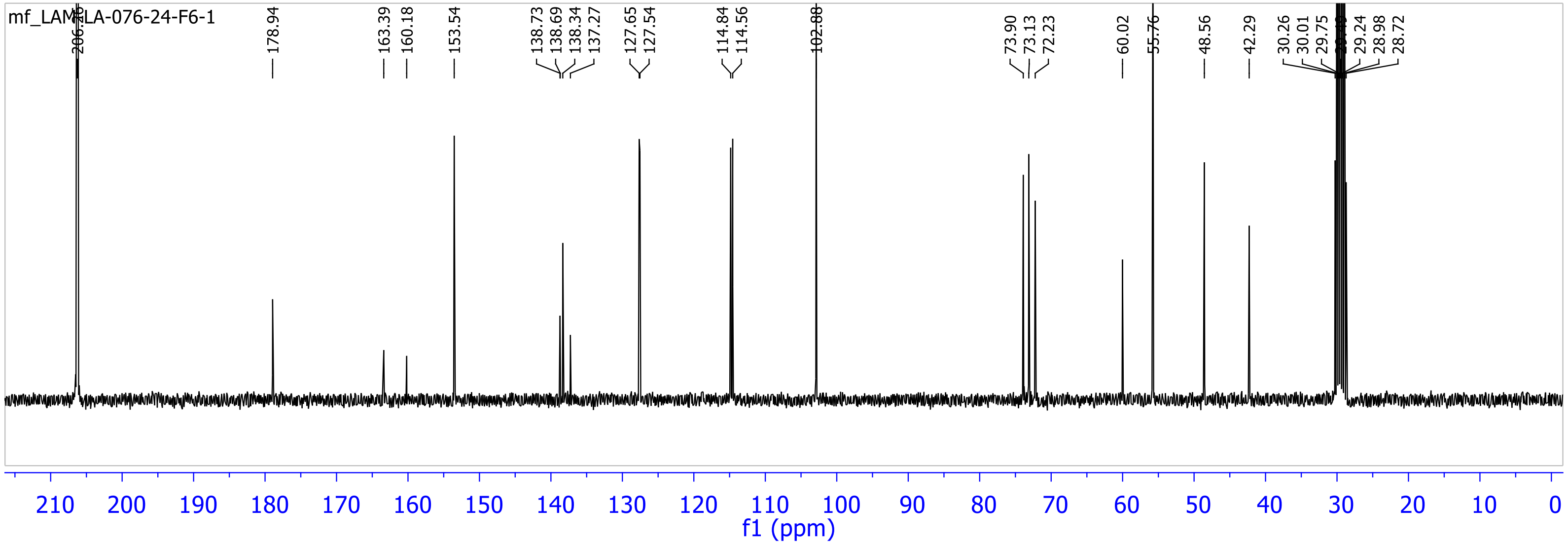

(3*S*,4*R*)-4-[(R)-(-Benzo[d][1,3]dioxol-5-yl(hydroxy)methyl]-3-[hydroxy(3,4,5-trimethoxyphenyl)methyl]dihydrofuran-2(3*H*)-one (12c).

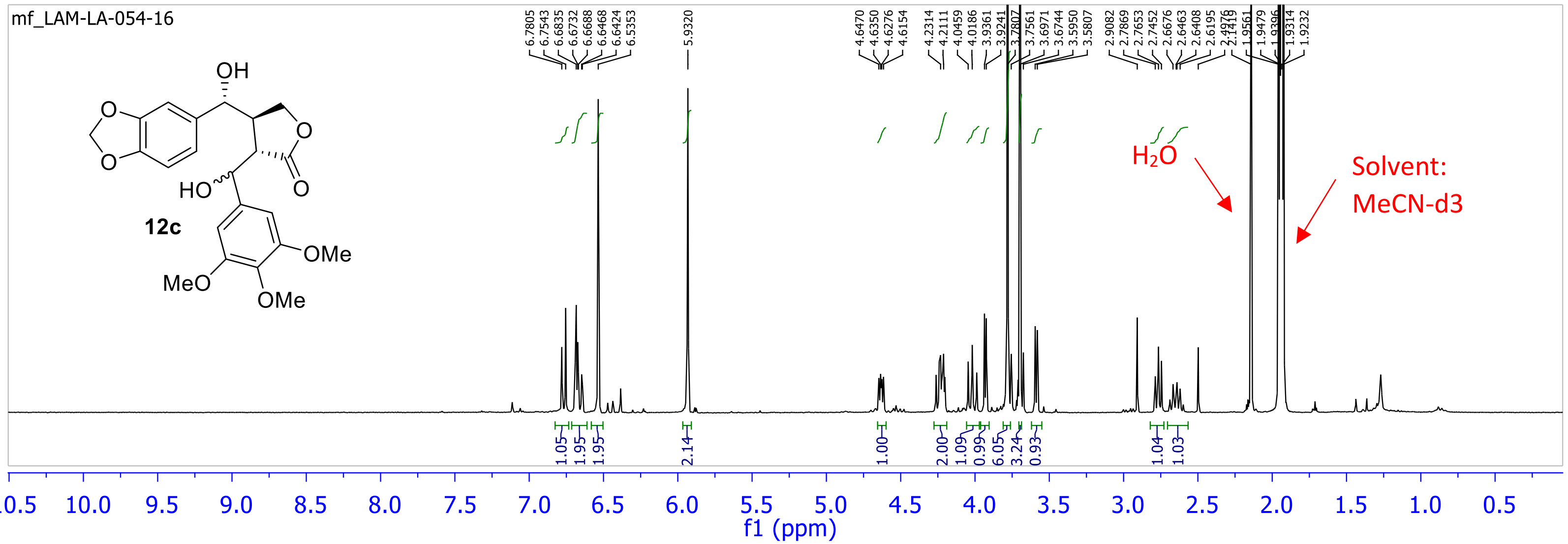

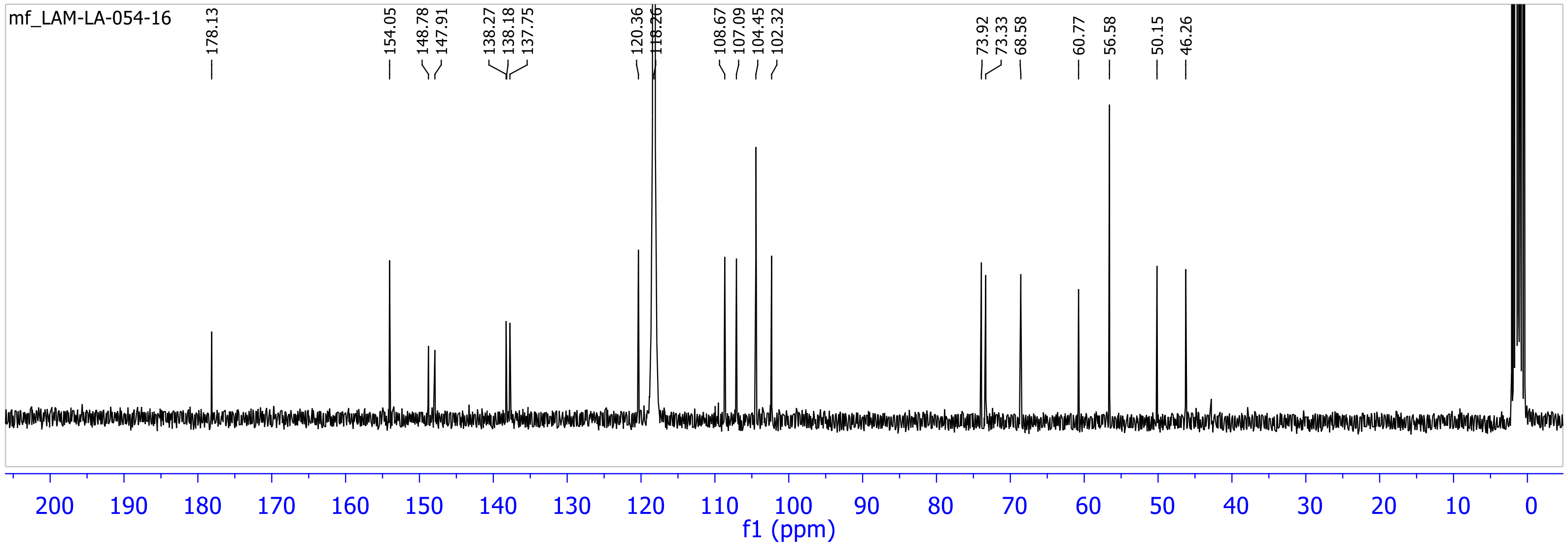

Podophyllotoxone (SI-42):

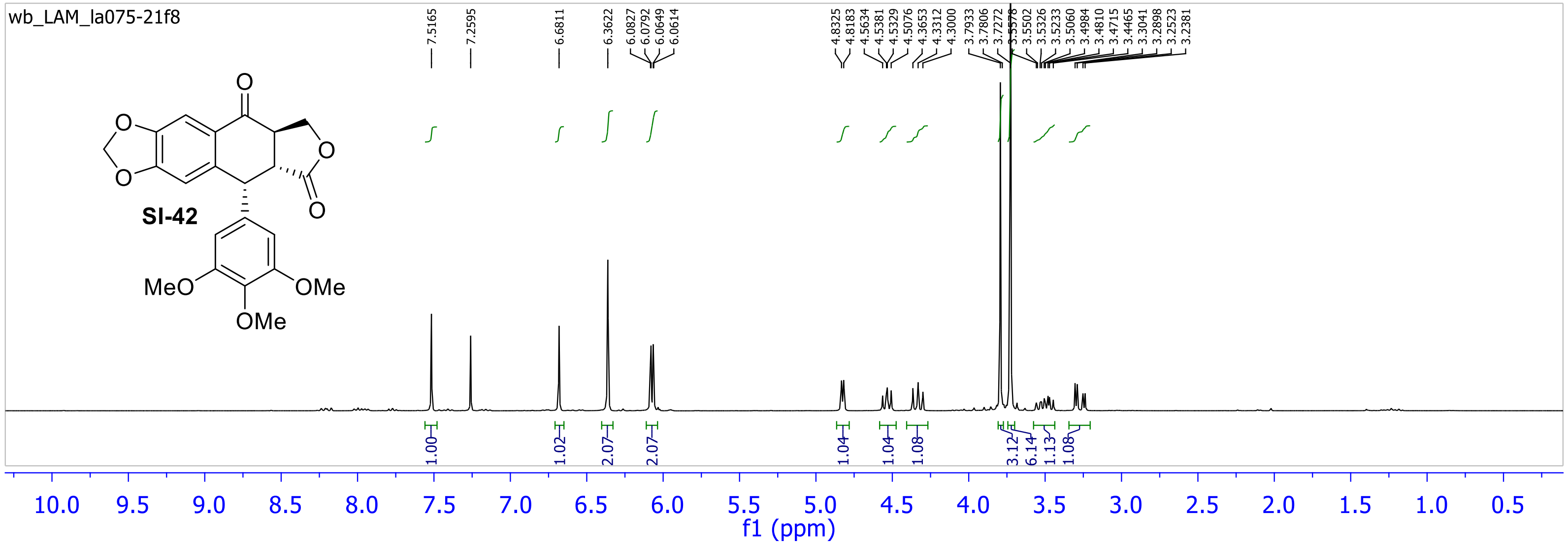

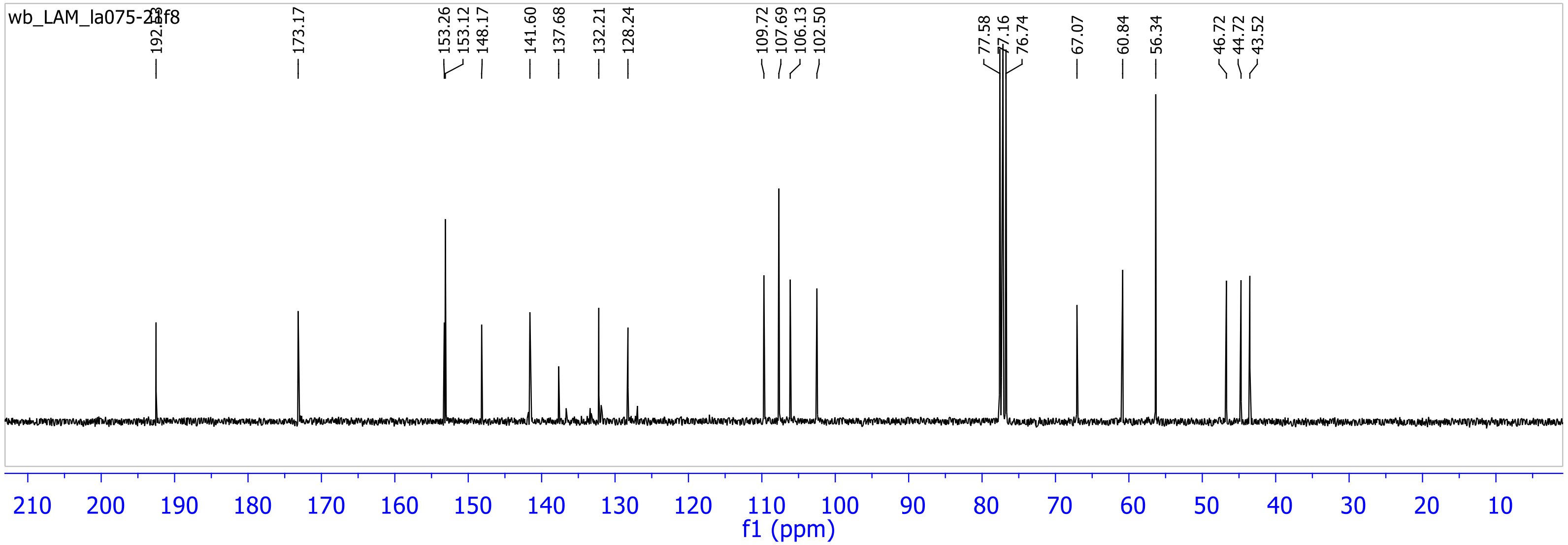

Podophyllotoxin (1):

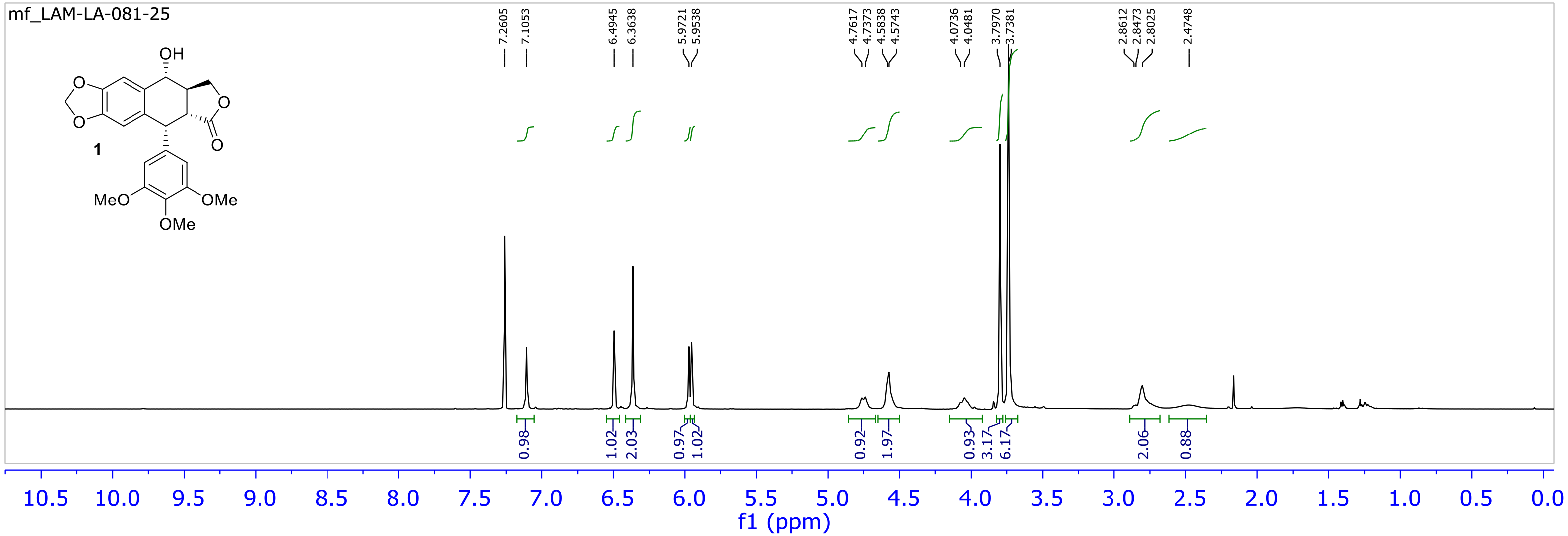

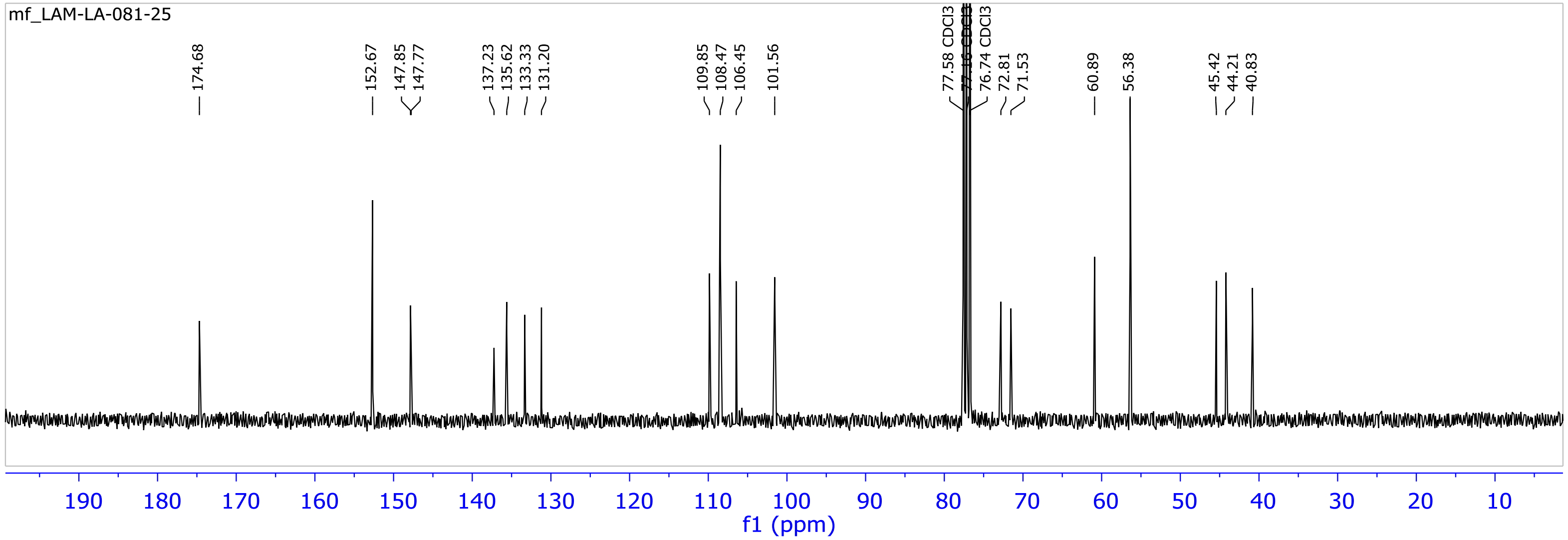

HPLC Chromatograms

epi-Podophyllotoxin (**11d**, 2 gram upscale experiment).

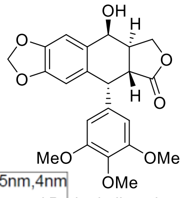

epi-Podophyllotoxin (**11d**)

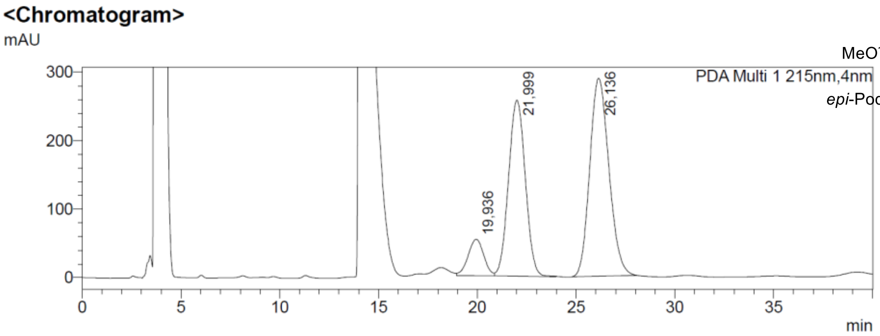

<Peak Table>

PDA Ch1 215nm

Peak#	Ret. Time	Name	Area	Height	Area%
1	19,936		2933813	53297	7,803
2	21,999		15171816	257057	40,353
3	26,136		19492539	288752	51,844
Total			37598167	599107	100,000

Crude reaction mixture

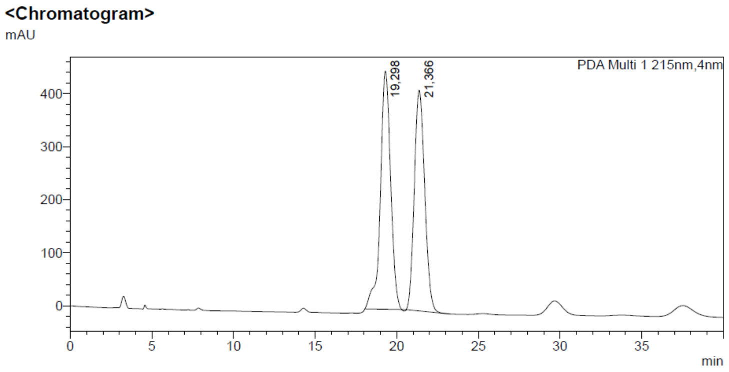

<Peak Table>

PDA Ch1 215nm

Peak#	Ret. Time	Area	Height	Area%
1	19,298	19628123	448861	51,544
2	21,366	18451998	415895	48,456
Total		38080121	864756	100,000

Racemic reference of substrate **2d**

<Chromatogram>

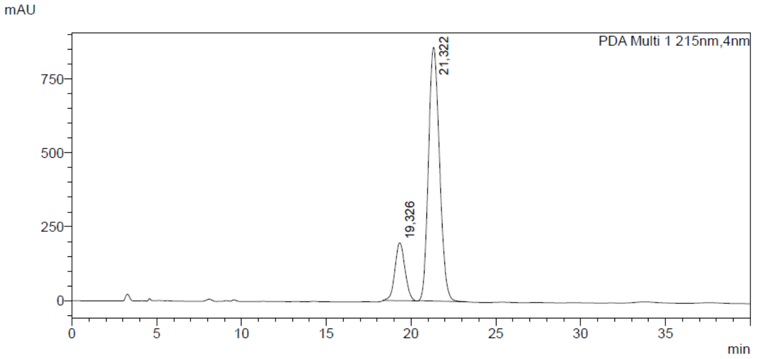

<Peak Table>

PDA Ch1 215nm

Peak#	Ret. Time	Area	Height	Area%
1	19,326	8062825	195572	17,165
2	21,322	38908678	857004	82,835
Total		46971504	1052576	100,000

Recovered substrate **2d**

<Chromatogram>

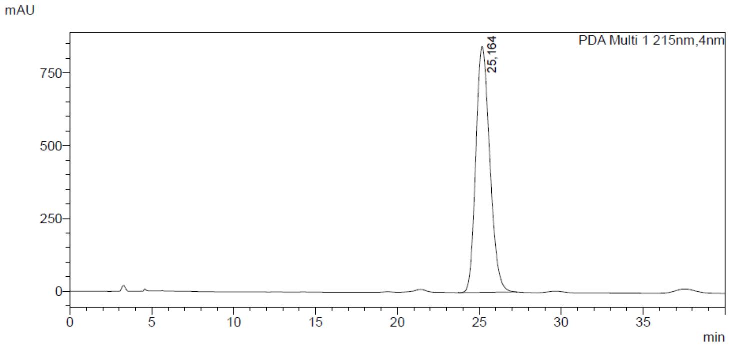

<Peak Table>

PDA Ch1 215nm

Peak#	Ret. Time	Area	Height	Area%
1	25,164	50179738	844366	100,000
Total		50179738	844366	100,000

Compound **11d**

Upscale of yatein (2).

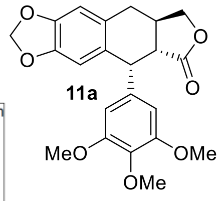

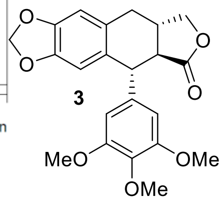

<Chromatogram>

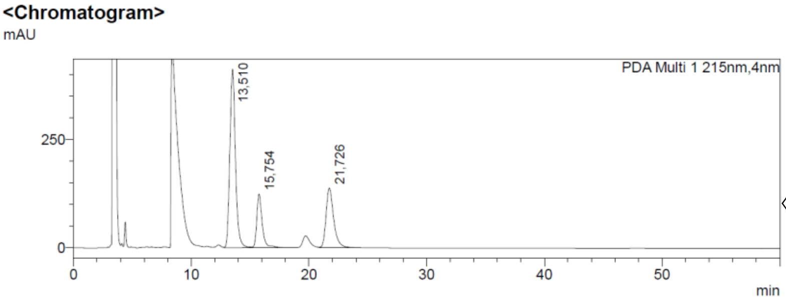

<Peak Table>

PDA Ch1 215nm

Peak#	Ret. Time	Name	Area	Height	Area%
1	13,510		13580316	411640	57,261
2	15,754		3954450	123566	16,674
3	21,726		6181656	137513	26,065
Total			23716422	672719	100,000

Crude reaction mixture

<Chromatogram>

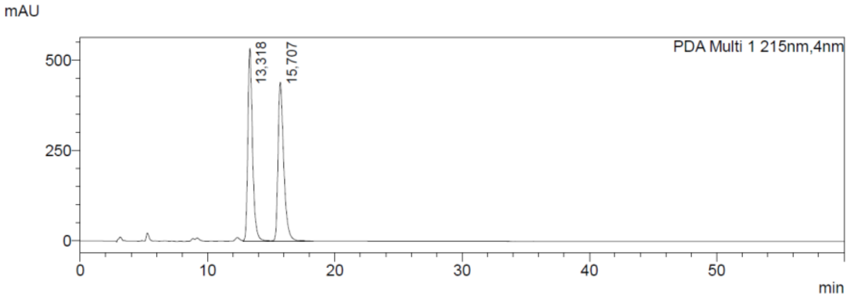

<Peak Table>

PDA Ch1 215nm

Peak#	Ret. Time	Name	Area	Height	Area%
1	13,318		13705736	533557	49,974
2	15,707		13720164	440127	50,026
Total			27425899	973684	100,000

Racemic reference of substrate **2a**

<Chromatogram>

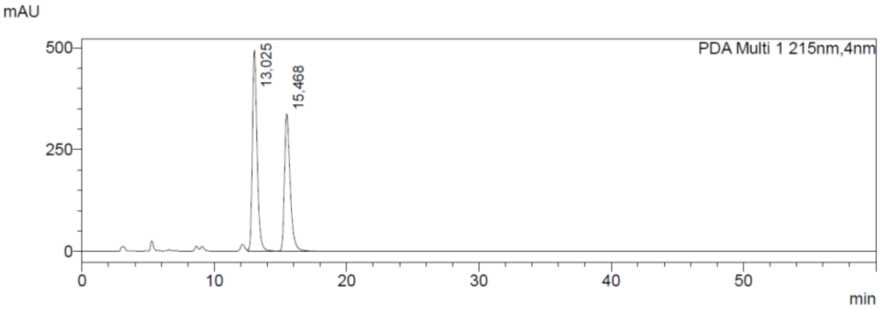

<Peak Table>

PDA Ch1 215nm

Peak#	Ret. Time	Name	Area	Height	Area%
1	13,025		12311061	493749	54,926
2	15,468		10102692	337427	45,074
Total			22413754	831176	100,000

Recovered substrate **2a**

<Chromatogram>

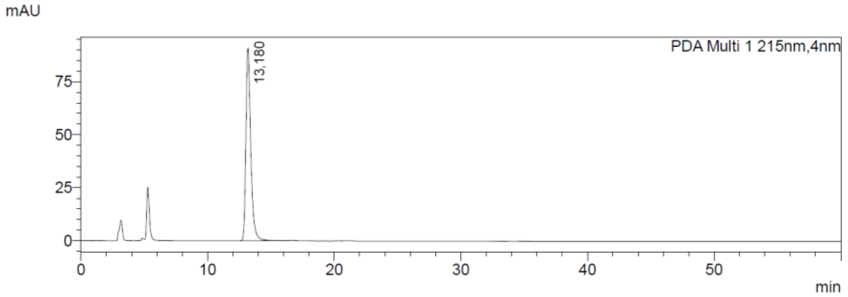

<Peak Table>

PDA Ch1 215nm

Peak#	Ret. Time	Name	Area	Height	Area%
1	13,180		2418696	90950	100,000
Total			2418696	90950	100,000

Deoxypodophyllotoxin (**11a**)

<Chromatogram>

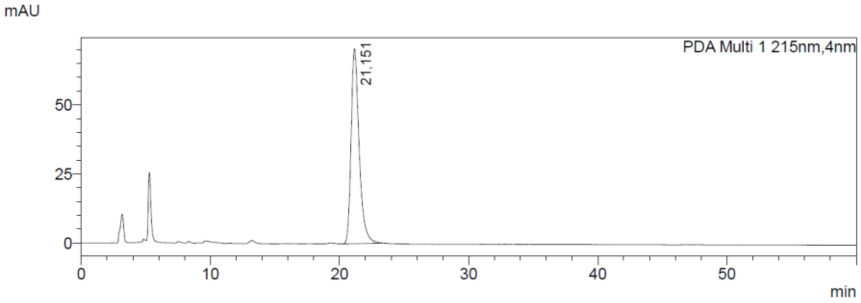

<Peak Table>

PDA Ch1 215nm

Peak#	Ret. Time	Name	Area	Height	Area%
1	21,151		3053049	70607	100,000
Total			3053049	70607	100,000

Isodeoxypodophyllotoxin (**3**)

Upscale of substrate **2c**.

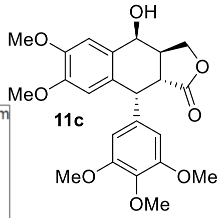

11c

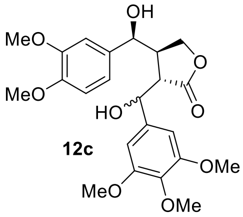

12c

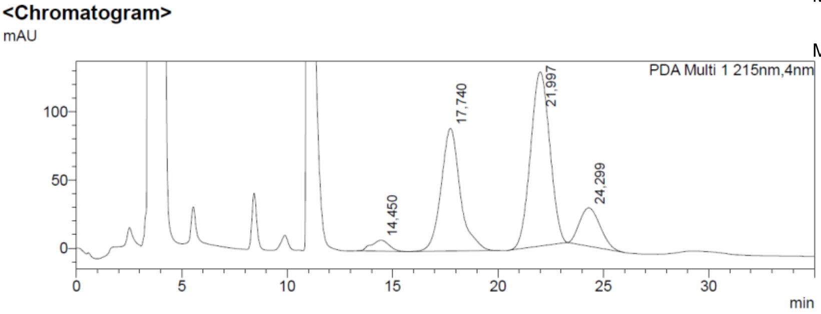

<Peak Table>
PDA Ch1 215nm

Peak#	Ret. Time	Name	Area	Height	Area%
1	14,450		469005	8043	2,897
2	17,740		5687073	89829	35,134
3	21,997		8118600	127566	50,155
4	24,299		1912290	28044	11,814
Total			16186968	253481	100,000

Crude reaction mixture

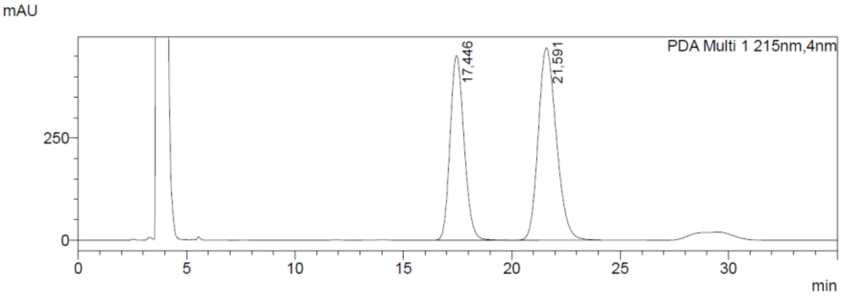

<Peak Table>
PDA Ch1 215nm

Peak#	Ret. Time	Name	Area	Height	Area%
1	17,446		20259195	452631	42,551
2	21,591		27352436	470781	57,449
Total			47611630	923412	100,000

Racemic reference of substrate **2c**

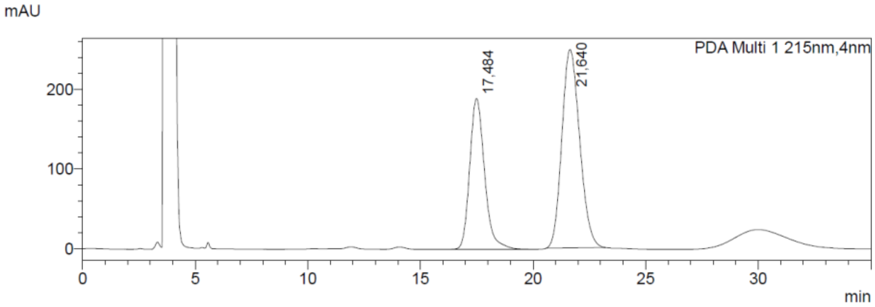

<Peak Table>
PDA Ch1 215nm

Peak#	Ret. Time	Name	Area	Height	Area%
1	17,484		8672539	189501	38,674
2	21,640		13752471	249446	61,326
Total			22425010	438946	100,000

Recovered substrate **2c**

<Chromatogram>

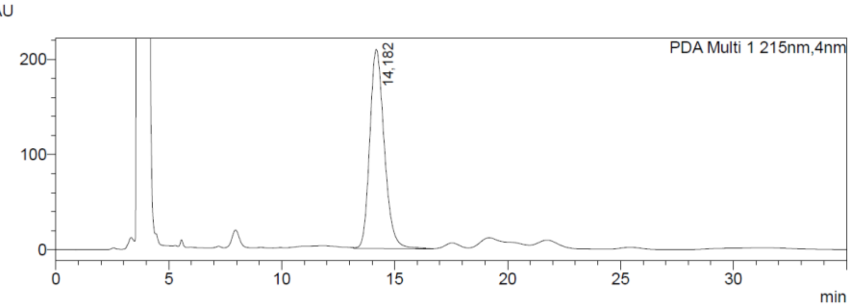

<Peak Table>

PDA Ch1 215nm

Peak#	Ret. Time	Name	Area	Height	Area%
1	14,182		9357106	209275	100,000
Total			9357106	209275	100,000

Product **11c**

<Chromatogram>

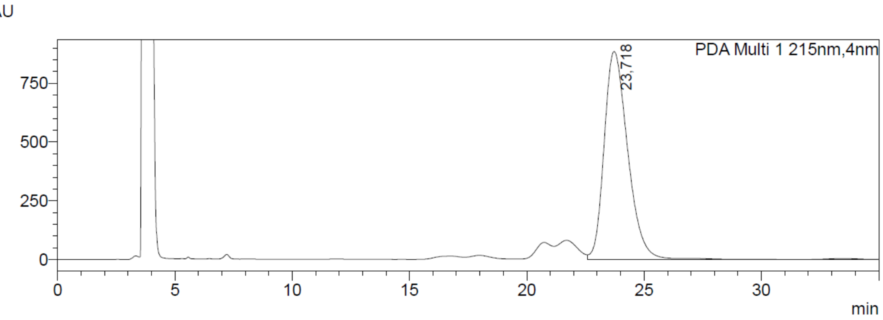

<Peak Table>

PDA Ch1 215nm

Peak#	Ret. Time	Name	Area	Height	Area%
1	23,718		62356207	885352	100,000
Total			62356207	885352	100,000

Product **12c**

(3*S*,4*R*)-3-[Hydroxy(3,4,5-trimethoxyphenyl)methyl]-4-[(*S*)-hydroxy(phenyl)methyl]dihydrofuran-2(3*H*)-one (**12e**).

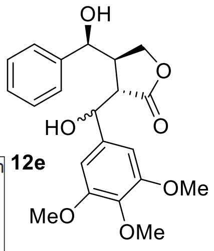

12e

<Chromatogram>

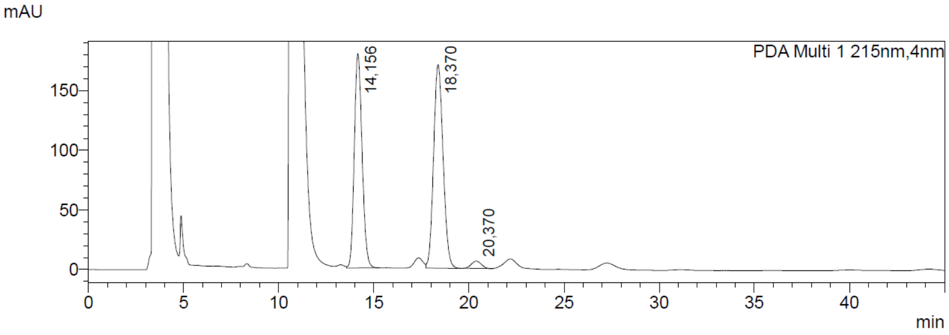

<Peak Table>

PDA Ch1 215nm

Peak#	Ret. Time	Name	Area	Height	Area%
1	14,156		5122339	179670	45,370
2	18,370		5944593	170629	52,654
3	20,370		223090	6162	1,976
Total			11290022	356461	100,000

Crude reaction mixture

<Chromatogram>

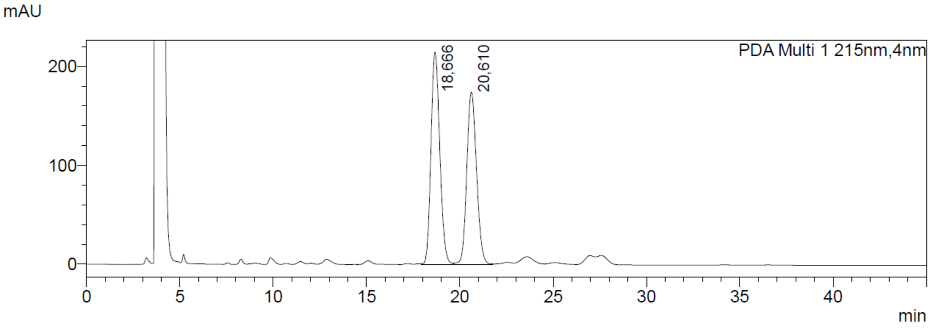

<Peak Table>

PDA Ch1 215nm

Peak#	Ret. Time	Name	Area	Height	Area%
1	18,666		7073410	214863	52,614
2	20,610		6370508	174507	47,386
Total			13443918	389371	100,000

Racemic reference of substrate **2e**

<Chromatogram>

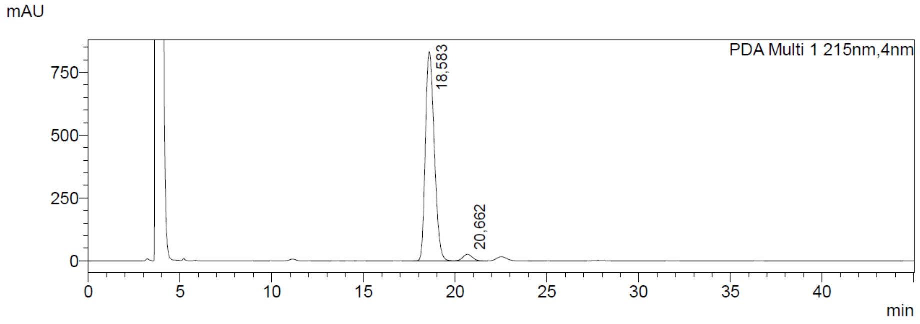

<Peak Table>

PDA Ch1 215nm

Peak#	Ret. Time	Name	Area	Height	Area%
1	18,583		27997281	832580	96,719
2	20,662		949761	26224	3,281
Total			28947042	858803	100,000

Recovered substrate **2e**

<Chromatogram>

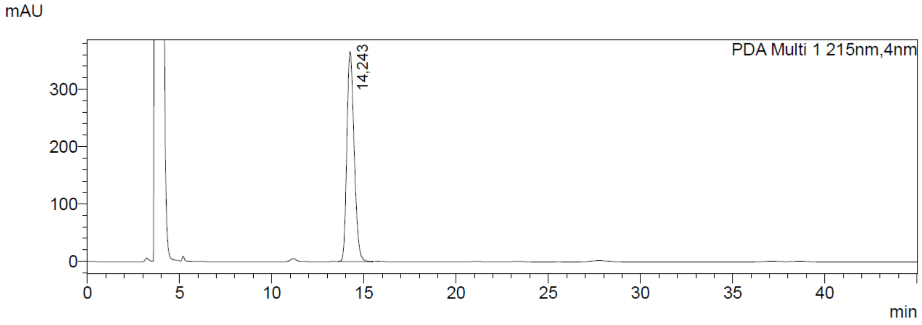

<Peak Table>

PDA Ch1 215nm

Peak#	Ret. Time	Name	Area	Height	Area%
1	14,243		10057215	365569	100,000
Total			10057215	365569	100,000

Product **12e**

(3*S*,4*R*)-3-[Hydroxy(3,4,5-trimethoxyphenyl)methyl]-4-[(*S*)-hydroxy(naphthalen-2-yl)methyl]dihydrofuran-2(3*H*)-one (**12f**).

No data for the crude reaction mixture is given due to overlap with of substrate 17 with the DMSO cosolvent.

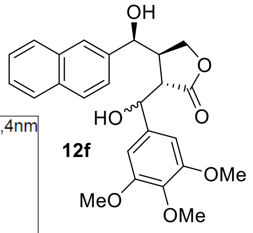

12f

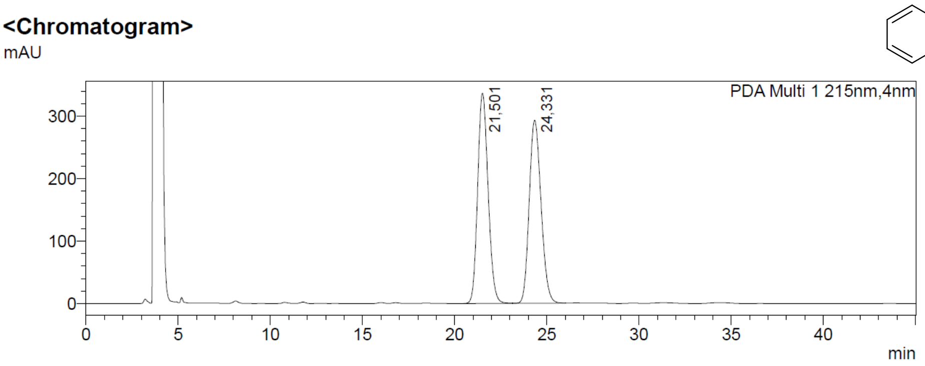

<Peak Table>

PDA Ch1 215nm

Peak#	Ret. Time	Name	Area	Height	Area%
1	21,501		13283820	336879	50,351
2	24,331		13098446	293183	49,649
Total			26382266	630061	100,000

Racemic reference of substrate **2f**

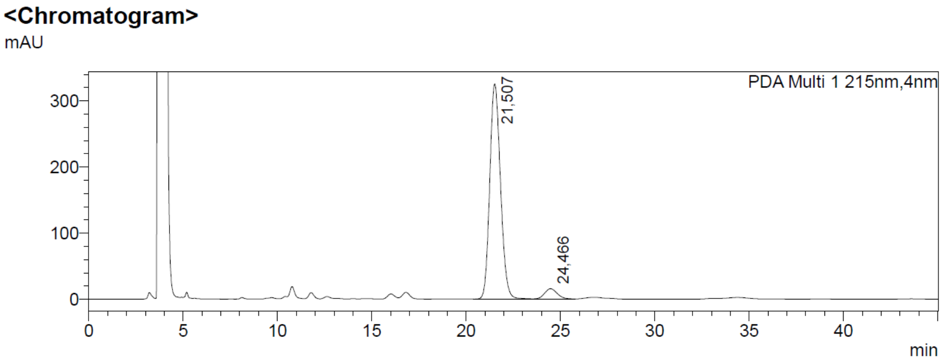

<Peak Table>

PDA Ch1 215nm

Peak#	Ret. Time	Name	Area	Height	Area%
1	21,507		12851780	325208	94,513
2	24,466		746148	15669	5,487
Total			13597928	340877	100,000

Recovered substrate **2f**

<Chromatogram>

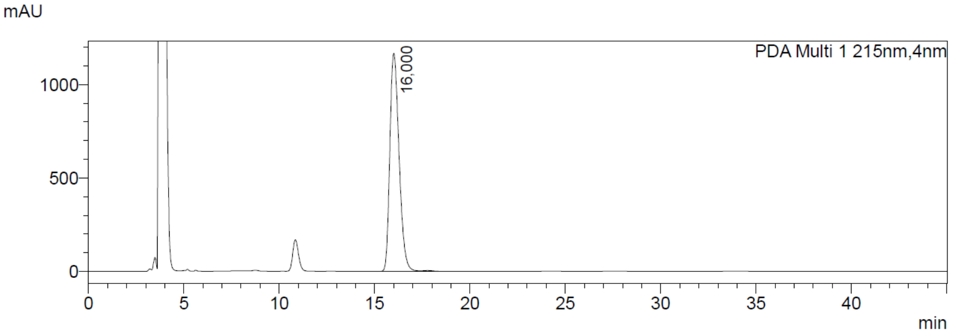

<Peak Table>

PDA Ch1 215nm

Peak#	Ret. Time	Name	Area	Height	Area%
1	16,000		39302837	1167082	100,000
Total			39302837	1167082	100,000

Product **12f**

(3*S*,4*R*)-4-[(*S*)-(3-chlorophenyl)(hydroxy)methyl]-3-[hydroxy(3,4,5-trimethoxyphenyl)methyl]dihydrofuran-2(3*H*)-one (**12g**).

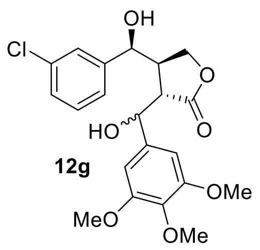

12g

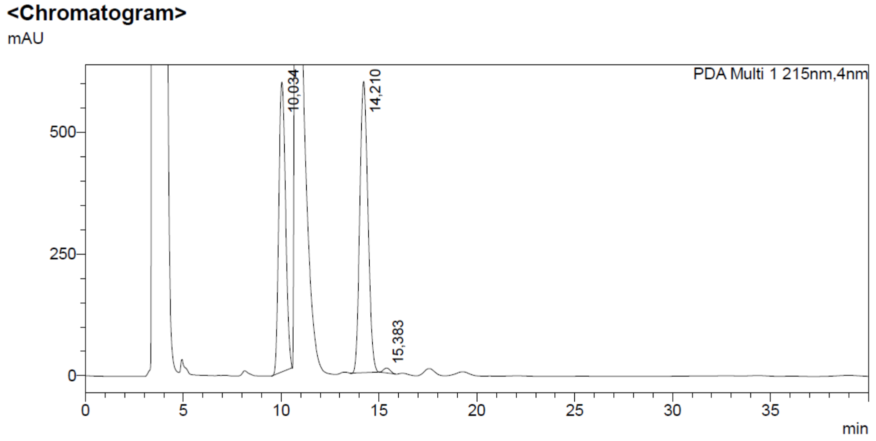

<Peak Table>

PDA Ch1 215nm

Peak#	Ret. Time	Area	Height	Area%
1	10,034	14294891	595461	44,961
2	14,210	17259501	598016	54,286
3	15,383	239514	9853	0,753
Total		31793906	1203330	100,000

Crude reaction mixture

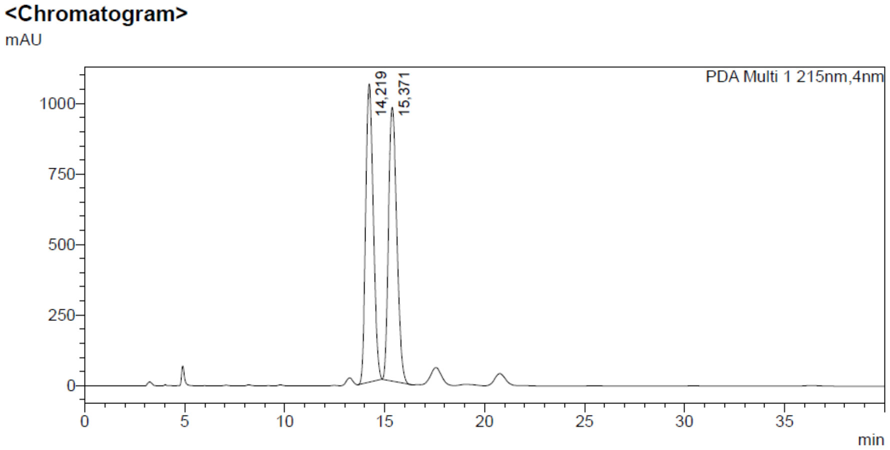

<Peak Table>

PDA Ch1 215nm

Peak#	Ret. Time	Area	Height	Area%
1	14,219	28017212	1055912	50,040
2	15,371	27972293	969649	49,960
Total		55989506	2025561	100,000

Racemic reference of substrate **2g**

<Chromatogram>

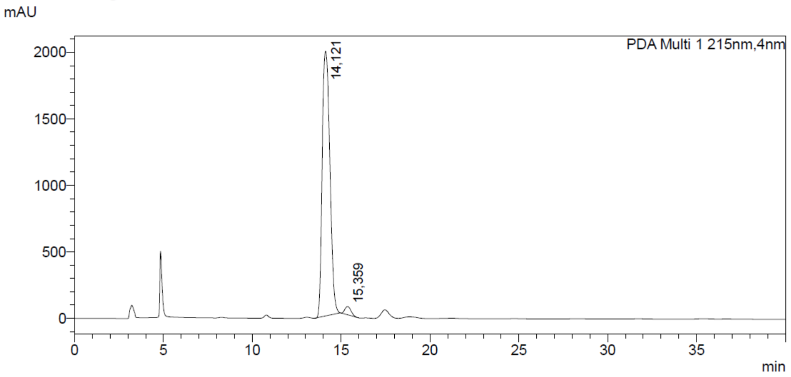

<Peak Table>

PDA Ch1 215nm

Peak#	Ret. Time	Area	Height	Area%
1	14,121	59810480	1991029	97,590
2	15,359	1476912	61391	2,410
Total		61287392	2052419	100,000

Recovered substrate **2g**

<Chromatogram>

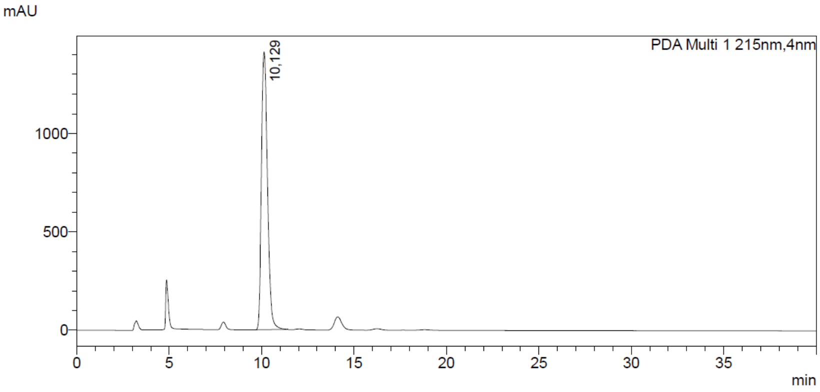

<Peak Table>

PDA Ch1 215nm

Peak#	Ret. Time	Area	Height	Area%
1	10,129	32245169	1411320	100,000
Total		32245169	1411320	100,000

Product **12g**

(3*S*,4*R*)-4-[(*S*)-(4-chlorophenyl)(hydroxy)methyl]-3-[hydroxy(3,4,5-trimethoxyphenyl)methyl]dihydrofuran-2(3*H*)-one (**12h**).

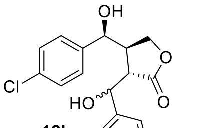

12h

<Chromatogram>

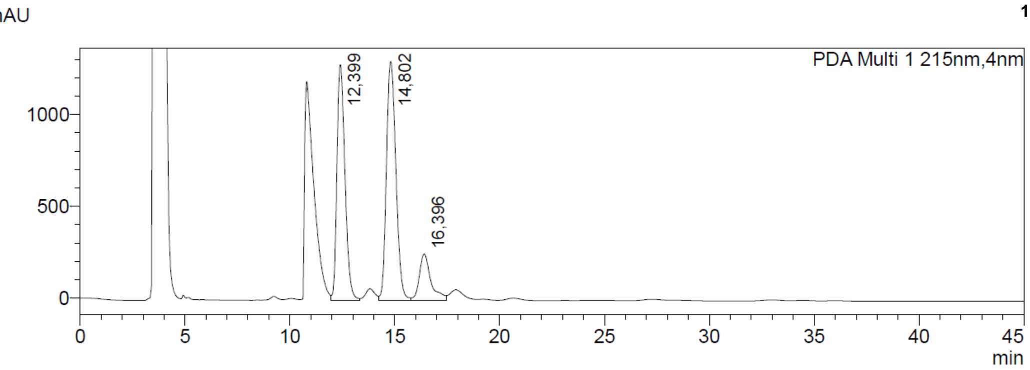

<Peak Table>

PDA Ch1 215nm

Peak#	Ret. Time	Name	Area	Height	Area%
1	12,399		34446800	1284598	40,361
2	14,802		41040117	1300772	48,086
3	16,396		9859570	254122	11,552
Total			85346487	2839492	100,000

Crude reaction mixture

<Chromatogram>

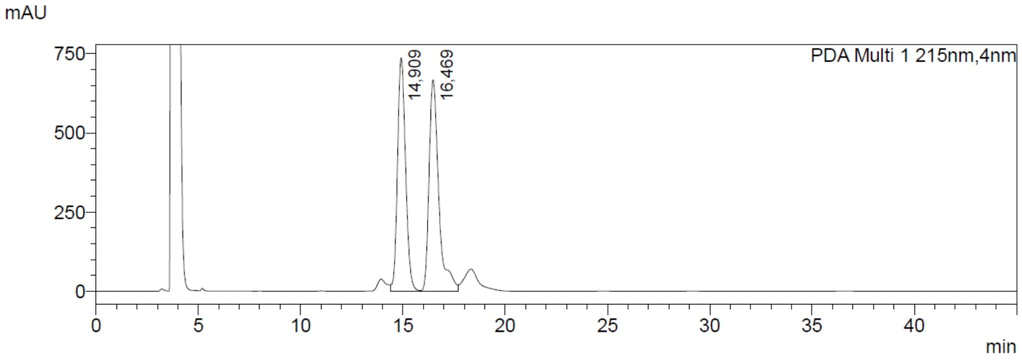

<Peak Table>

PDA Ch1 215nm

Peak#	Ret. Time	Name	Area	Height	Area%
1	14,909		20256331	737416	47,903
2	16,469		22029520	666901	52,097
Total			42285851	1404316	100,000

Racemic reference for substrate **2h**

<Chromatogram>

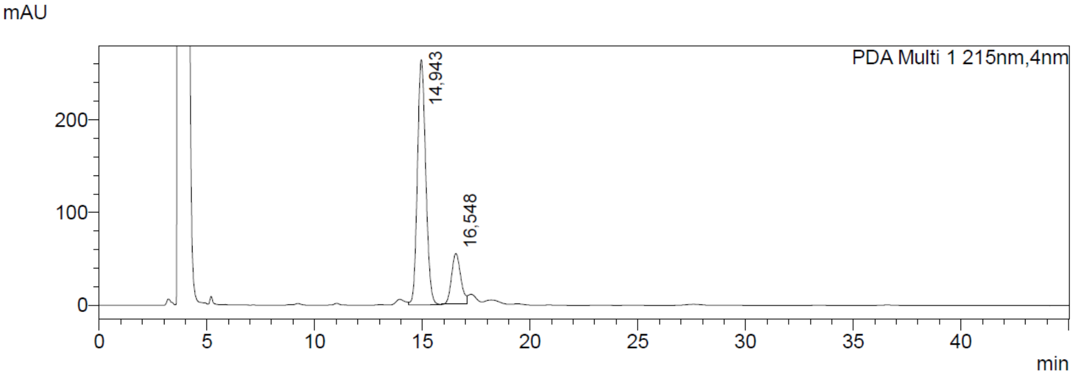

<Peak Table>

PDA Ch1 215nm

Peak#	Ret. Time	Name	Area	Height	Area%
1	14,943		7104557	264225	81,425
2	16,548		1620685	54716	18,575
Total			8725242	318940	100,000

Recovered substrate **2h**

<Chromatogram>

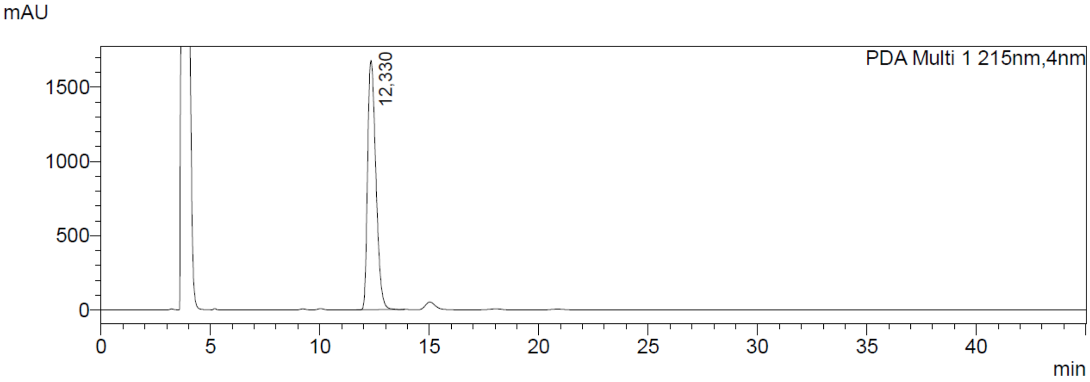

<Peak Table>

PDA Ch1 215nm

Peak#	Ret. Time	Name	Area	Height	Area%
1	12,330		44289409	1678793	100,000
Total			44289409	1678793	100,000

Product **12h**

(3S,4R)-4-[(S)-(3-iodophenyl)(hydroxy)methyl]-3-[hydroxy(3,4,5-trimethoxyphenyl)methyl]dihydrofuran-2(3*H*)-one (**12i**).

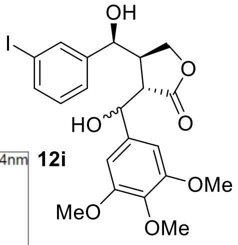

12i

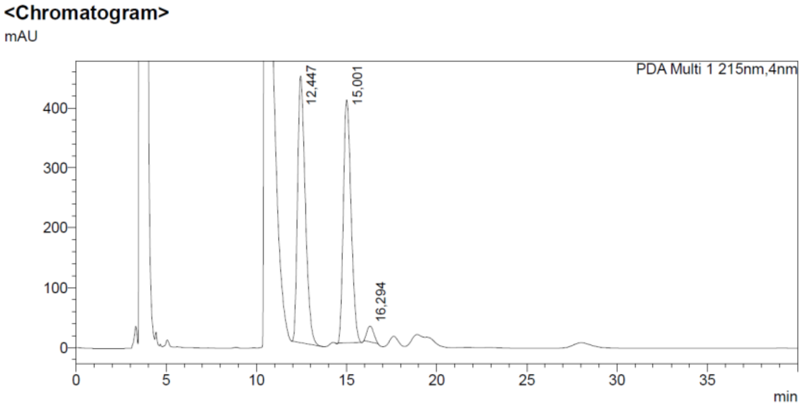

<Peak Table>

PDA Ch1 215nm

Peak#	Ret. Time	Area	Height	Area%
1	12,447	13201959	444539	50,854
2	15,001	12071943	405321	46,501
3	16,294	686748	26299	2,645
Total		25960650	876159	100,000

Crude reaction mixture

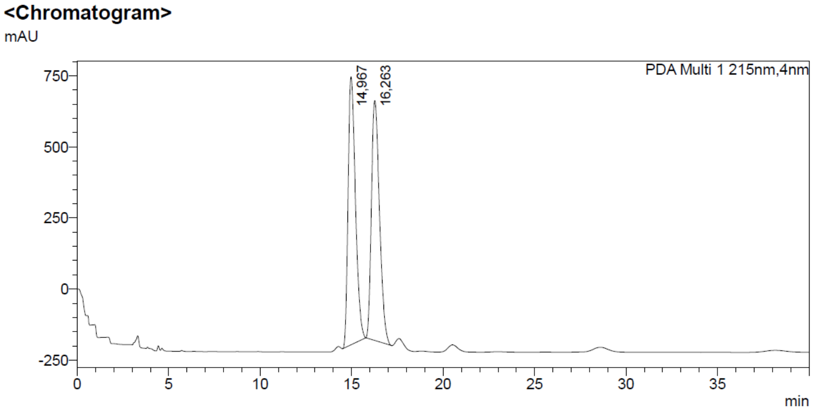

<Peak Table>

PDA Ch1 215nm

Peak#	Ret. Time	Area	Height	Area%
1	14,967	26347425	942876	50,419
2	16,263	25909829	844001	49,581
Total		52257255	1786877	100,000

Racemic reference for substrate **2i**

<Chromatogram>

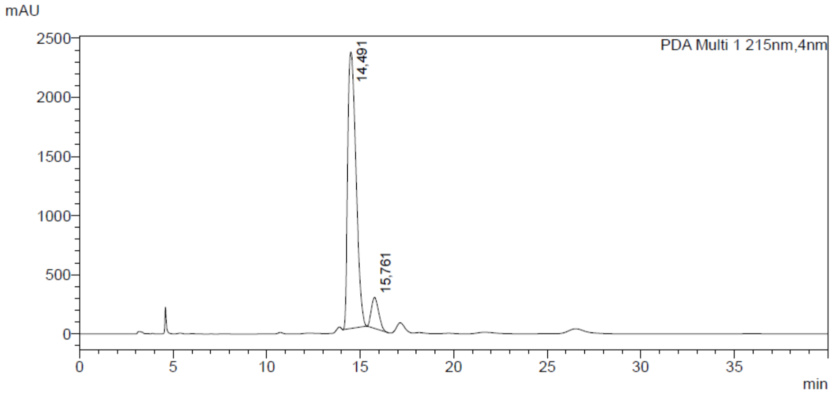

<Peak Table>

PDA Ch1 215nm

Peak#	Ret. Time	Area	Height	Area%
1	14,491	72135397	2339528	90,979
2	15,761	7152724	262152	9,021
Total		79288121	2601680	100,000

Recovered substrate **2i**

<Chromatogram>

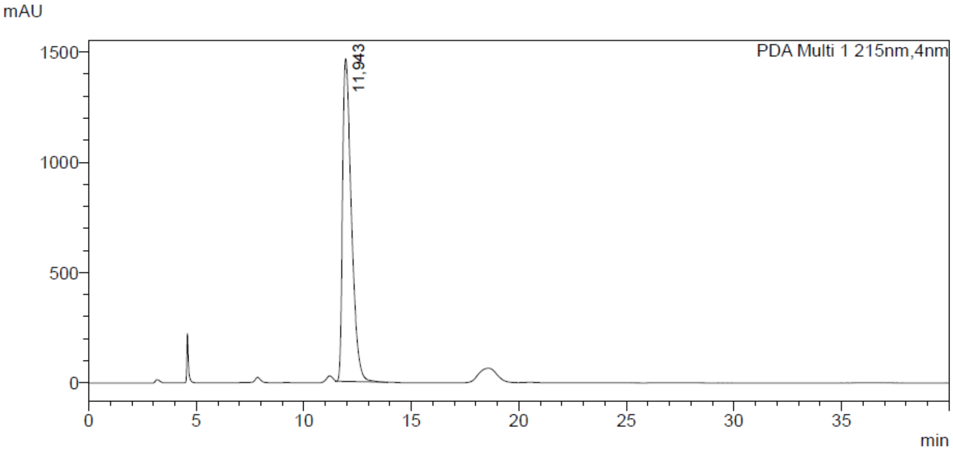

<Peak Table>

PDA Ch1 215nm

Peak#	Ret. Time	Area	Height	Area%
1	11,943	41823150	1463438	100,000
Total		41823150	1463438	100,000

Product **12i**

Methyl 4-((1*S*)-hydroxy{(3*R*,4*S*)-4-[hydroxy(3,4,5-trimethoxyphenyl)methyl]-5-oxotetrahydrofuran-3-yl}methyl)benzoate (**12j**).

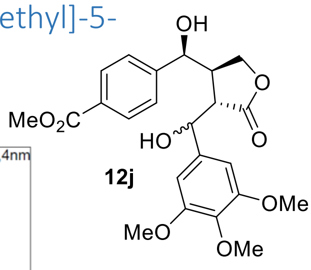

12j

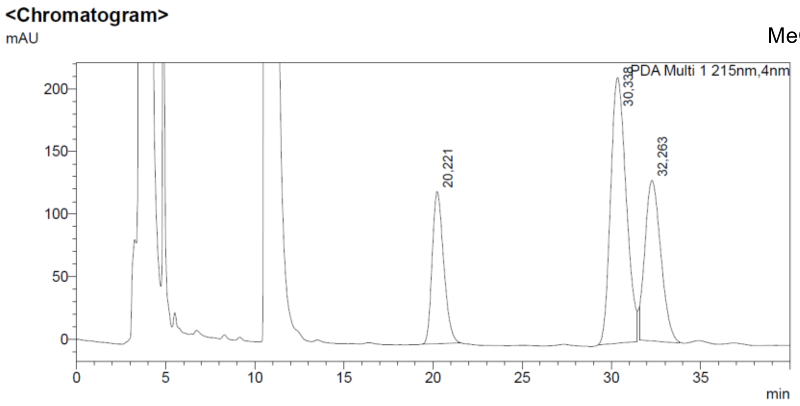

<Peak Table>

PDA Ch1 215nm

Peak#	Ret. Time	Area	Height	Area%
1	20,221	5453471	121338	20,758
2	30,338	12861273	212083	48,954
3	32,263	7957379	128247	30,288
Total		26272123	461669	100,000

Crude reaction mixture

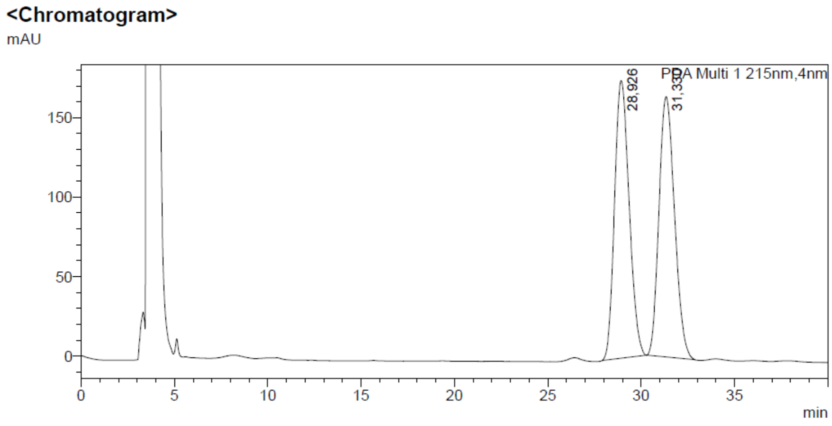

<Peak Table>

PDA Ch1 215nm

Peak#	Ret. Time	Area	Height	Area%
1	28,926	9490859	174722	49,980
2	31,330	9498499	163882	50,020
Total		18989358	338605	100,000

Racemic reference for substrate **2j**

<Chromatogram>

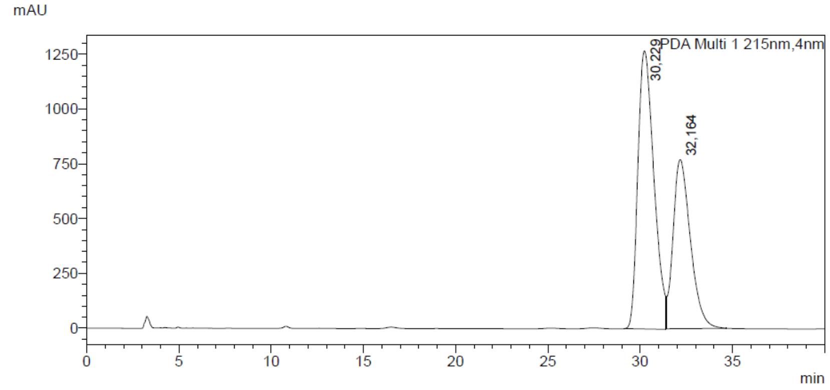

<Peak Table>

PDA Ch1 215nm

Peak#	Ret. Time	Area	Height	Area%
1	30,229	76639010	1267825	60,311
2	32,164	50433992	771387	39,689
Total		127073002	2039212	100,000

Recovered substrate **2j**

<Chromatogram>

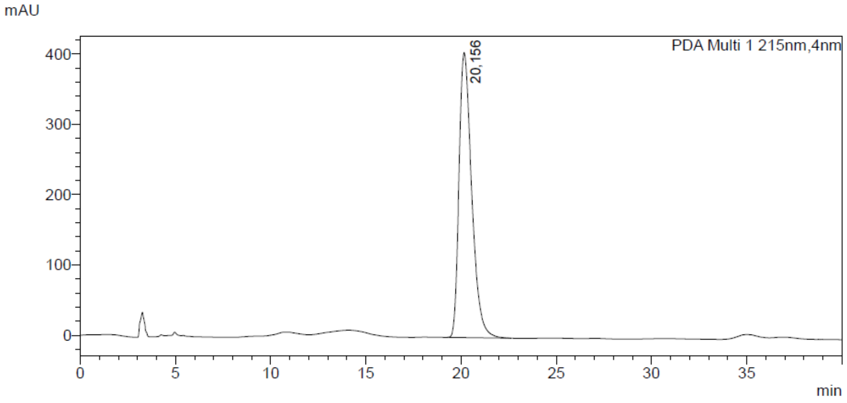

<Peak Table>

PDA Ch1 215nm

Peak#	Ret. Time	Area	Height	Area%
1	20,156	18760484	405863	100,000
Total		18760484	405863	100,000

Product **12j**

(3*S*,4*R*)-3-[hydroxy(3,4,5-trimethoxyphenyl)methyl]-4-[(*S*)-hydroxy(4-methoxyphenyl)methyl]dihydrofuran-2(3*H*)-one (**12k**).

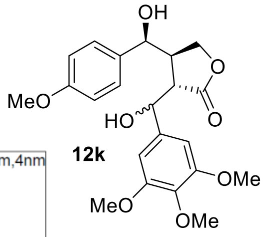

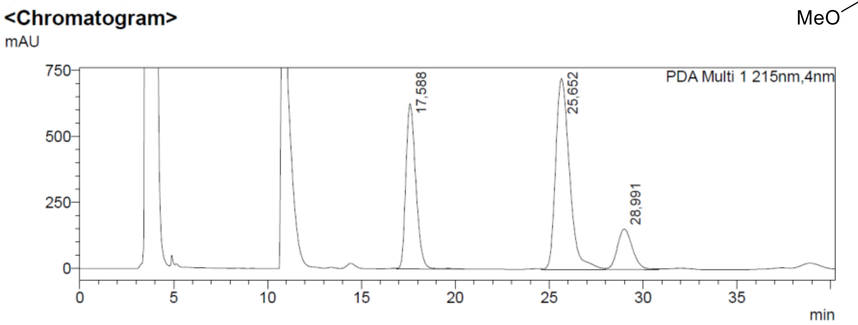

<Peak Table>

PDA Ch1 215nm

Peak#	Ret. Time	Name	Area	Height	Area%
1	17,588		23042548	625184	33,492
2	25,652		37242330	722934	54,131
3	28,991		8514866	152933	12,376
Total			68799744	1501051	100,000

Crude reaction mixture

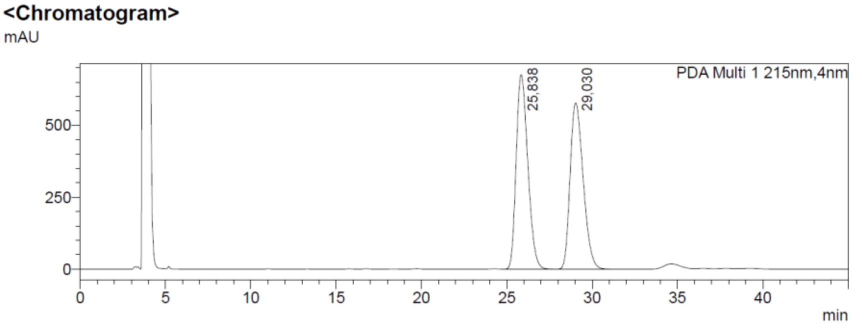

<Peak Table>

PDA Ch1 215nm

Peak#	Ret. Time	Name	Area	Height	Area%
1	25,838		32054582	675729	50,961
2	29,030		30845824	577721	49,039
Total			62900406	1253451	100,000

Racemic reference of substrate **2k**

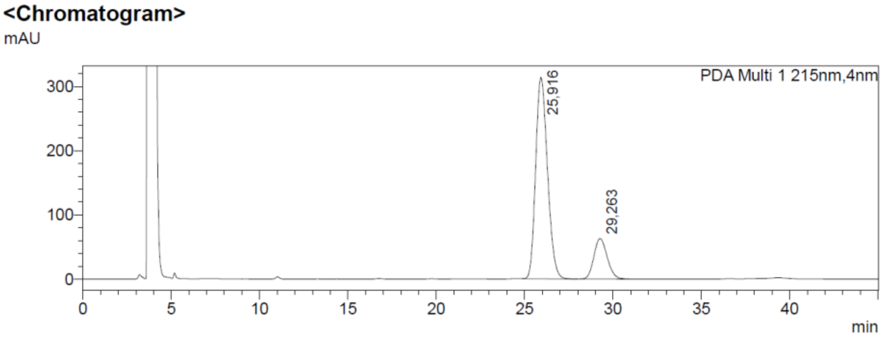

<Peak Table>

PDA Ch1 215nm

Peak#	Ret. Time	Name	Area	Height	Area%
1	25,916		14615347	313846	81,601
2	29,263		3295477	63061	18,399
Total			17910824	376907	100,000

Recovered substrate **2k**

<Chromatogram>

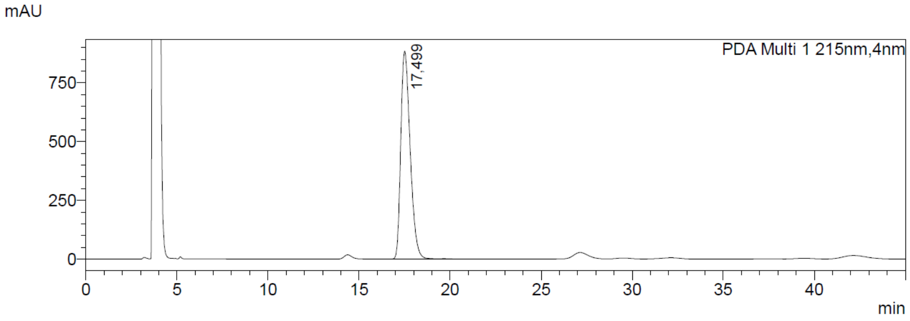

<Peak Table>

PDA Ch1 215nm

Peak#	Ret. Time	Name	Area	Height	Area%
1	17,499		31708164	883706	100,000
Total			31708164	883706	100,000

Product **12k**

(3*S*,4*R*)-3-[hydroxy(3,4,5-trimethoxyphenyl)methyl]-4-[(*S*)-hydroxy(3-methoxyphenyl)methyl]dihydrofuran-2(3*H*)-one (**11l**).

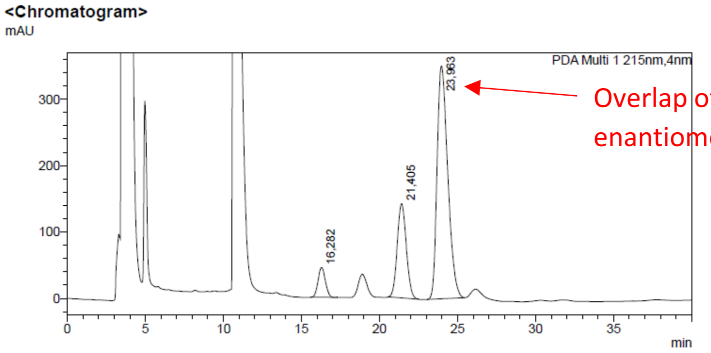

Overlap of product **11l** and one enantiomer of substrate **2l**

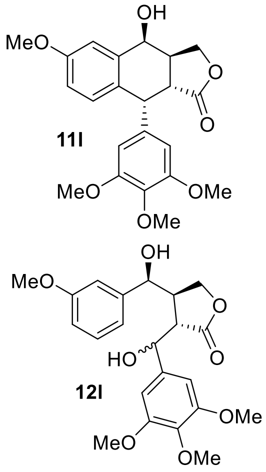

<Peak Table>

PDA Ch1 215nm

Peak#	Ret. Time	Area	Height	Area%
1	16,282	1463014	44845	6,124
2	21,405	5739790	142247	24,027
3	23,963	16686132	350698	69,849
Total		23888936	537790	100,000

Crude reaction mixture

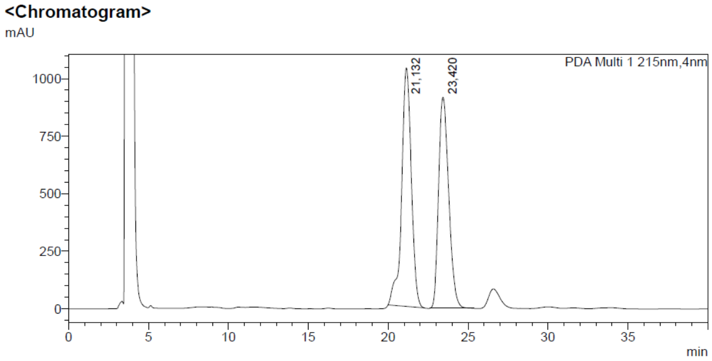

<Peak Table>

PDA Ch1 215nm

Peak#	Ret. Time	Area	Height	Area%
1	21,132	43757971	1036201	51,891
2	23,420	40568566	917384	48,109
Total		84326537	1953585	100,000

Racemic reference of substrate **2l**

<Chromatogram>

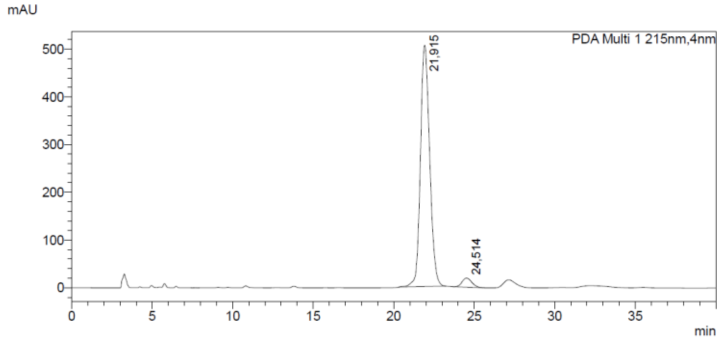

<Peak Table>

PDA Ch1 215nm

Peak#	Ret. Time	Area	Height	Area%
1	21,915	20224491	505445	96,156
2	24,514	808548	19127	3,844
Total		21033039	524572	100,000

Recovered substrate **2l**

<Chromatogram>

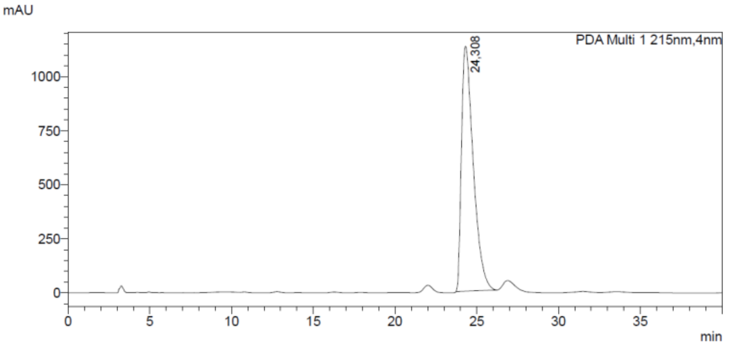

<Peak Table>

PDA Ch1 215nm

Peak#	Ret. Time	Area	Height	Area%
1	24,308	56811999	1132624	100,000
Total		56811999	1132624	100,000

Product **11l**

<Chromatogram>

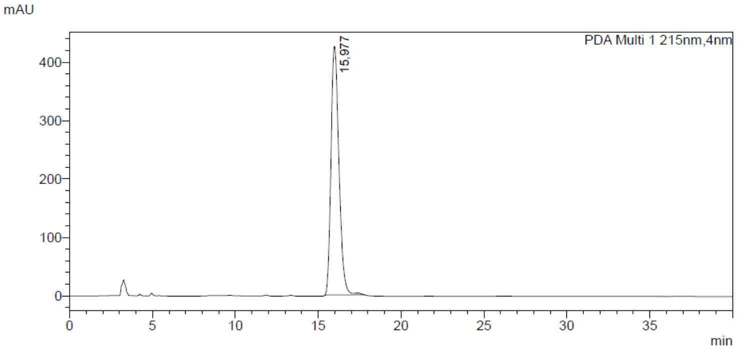

<Peak Table>

PDA Ch1 215nm

Peak#	Ret. Time	Area	Height	Area%
1	15,977	14234600	425716	100,000
Total		14234600	425716	100,000

Product **12l**

(3*S*,4*R*)-4-[(*S*)-(4-fluorophenyl)(hydroxy)methyl]-3-[hydroxy(3,4,5-trimethoxyphenyl)methyl]dihydrofuran-2(3*H*)-one (**12m**).

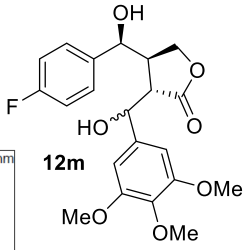

12m

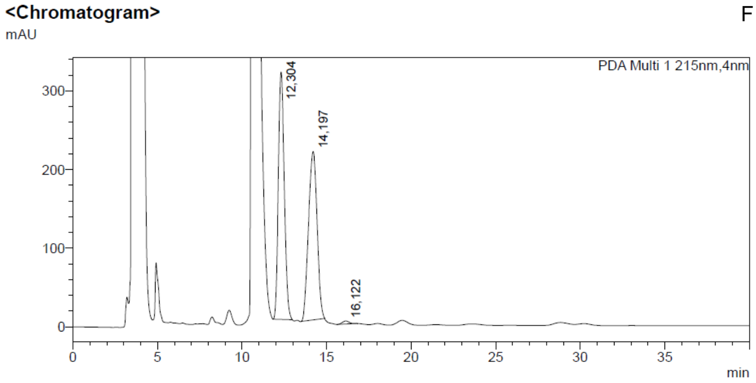

<Peak Table>

PDA Ch1 215nm

Peak#	Ret. Time	Area	Height	Area%
1	12,304	7700923	314841	50,081
2	14,197	7571785	214368	49,242
3	16,122	104092	3755	0,677
Total		15376801	532964	100,000

Crude reaction mixture

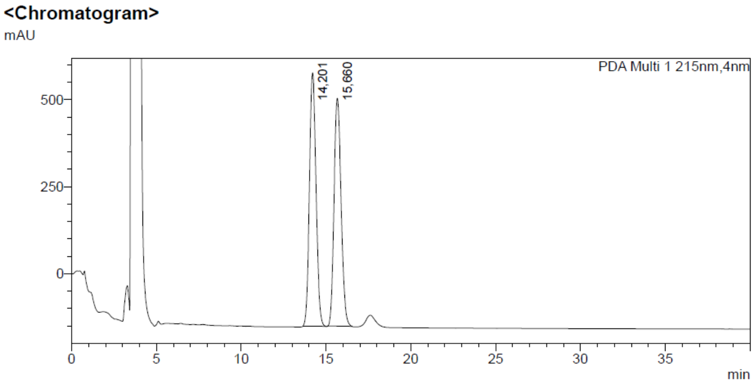

<Peak Table>

PDA Ch1 215nm

Peak#	Ret. Time	Area	Height	Area%
1	14,201	19674834	727595	50,906
2	15,660	18974658	654332	49,094
Total		38649492	1381927	100,000

Racemic reference of substrate **2m**

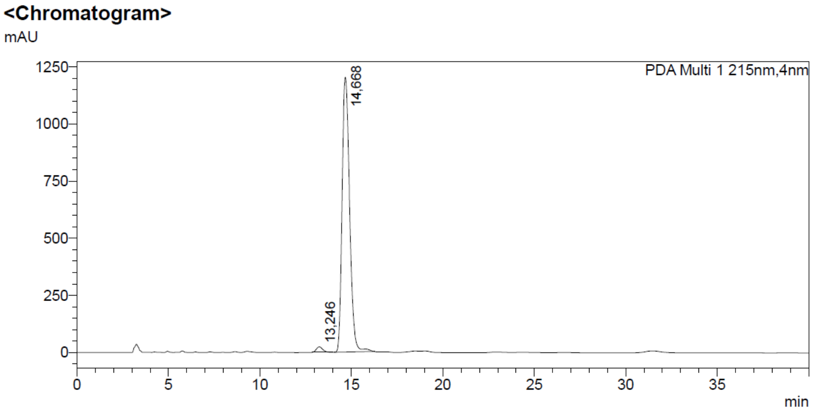

<Peak Table>

PDA Ch1 215nm

Peak#	Ret. Time	Area	Height	Area%
1	13,246	582372	23845	1,669
2	14,668	34311728	1202495	98,331
Total		34894100	1226340	100,000

Recovered substrate **2m**

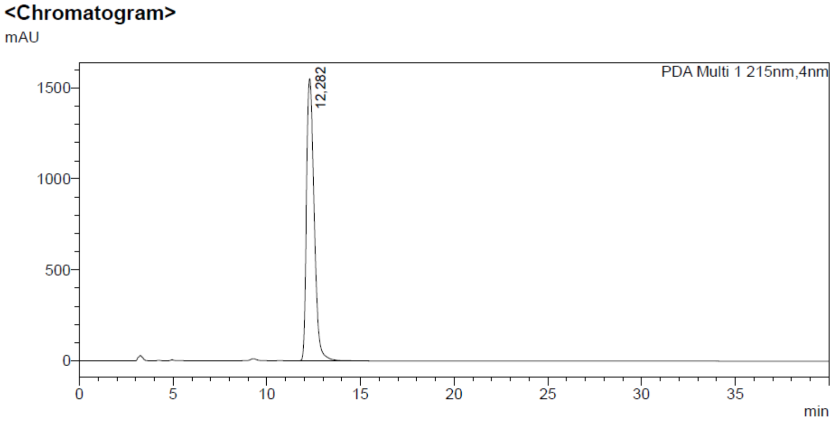

<Peak Table>

PDA Ch1 215nm

Peak#	Ret. Time	Height	Area	Area%
1	12,282	1551104	44150165	100,000
Total		1551104	44150165	100,000

Product **12m**

(3*S*,4*R*)-4-[(*R*)-(-Benzo[d][1,3]dioxol-5-yl(hydroxy)methyl]-3-[hydroxy(3,4,5-trimethoxyphenyl)methyl]dihydrofuran-2(3*H*)-one (Upscale Substrate **2b**).

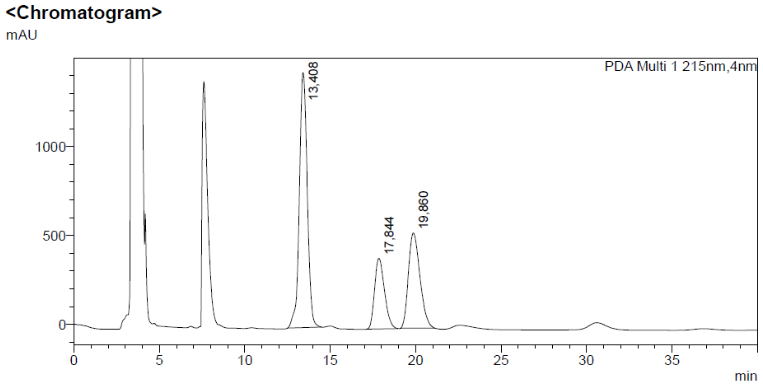

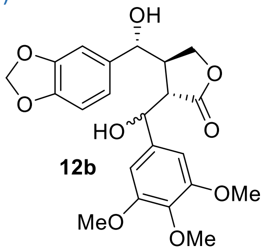

<Peak Table>

PDA Ch1 215nm

Peak#	Ret. Time	Area	Height	Area%
1	13,408	46137566	1434766	52,847
2	17,844	15993747	396914	18,319
3	19,860	25173432	536485	28,834
Total		87304745	2368165	100,000

Crude reaction mixture

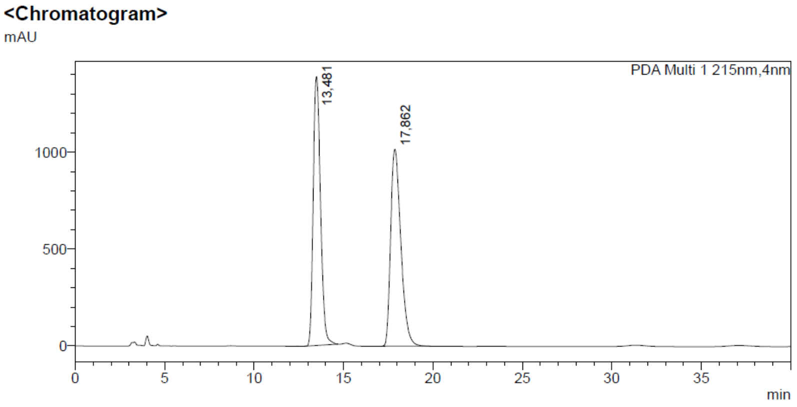

<Peak Table>

PDA Ch1 215nm

Peak#	Ret. Time	Area	Height	Area%
1	13,481	39270400	1387055	49,353
2	17,862	40299689	1016261	50,647
Total		79570089	2403316	100,000

Racemic reference of substrate **2b**

<Chromatogram>

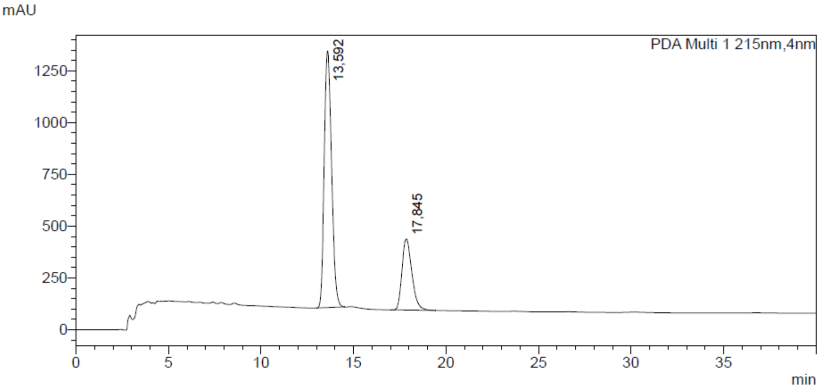

<Peak Table>

PDA Ch1 215nm

Peak#	Ret. Time	Area	Height	Area%
1	13,592	34352813	1239749	72,893
2	17,845	12775235	343733	27,107
Total		47128048	1583482	100,000

Recovered starting material **2b**

<Chromatogram>

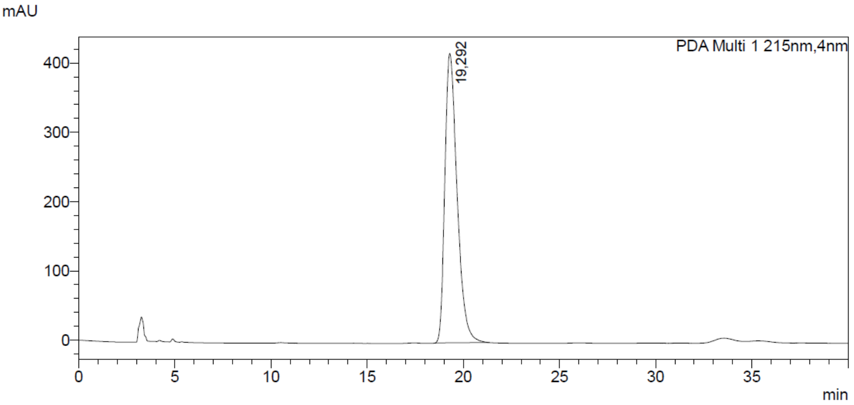

<Peak Table>

PDA Ch1 215nm

Peak#	Ret. Time	Area	Height	Area%
1	19,292	18474033	417855	100,000
Total		18474033	417855	100,000

Compound **12b**

Biotransformation of (3*R*,4*R*)-4-[(*S*)-hydroxy(4-iodophenyl)methyl]-3-(3,4,5-trimethoxybenzyl)dihydrofuran-2(3*H*)-one (**SI-1**).

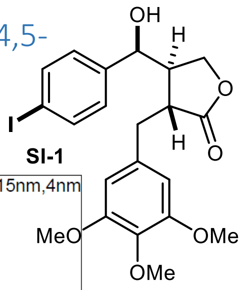

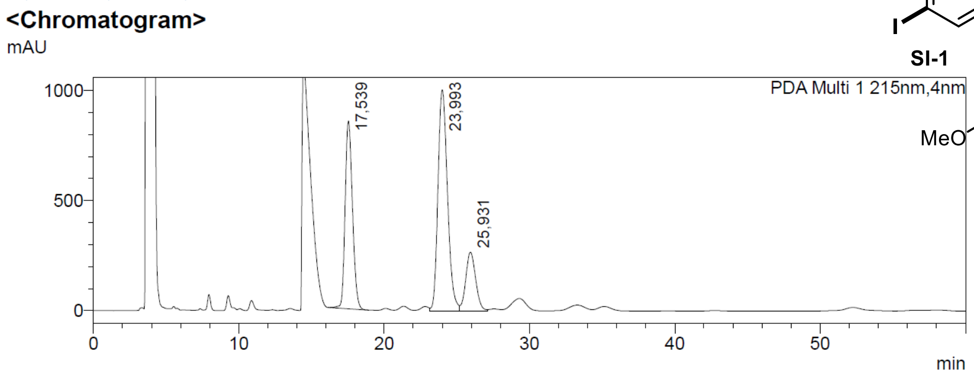

<Peak Table>

PDA Ch1 215nm

Peak#	Ret. Time	Name	Area	Height	Area%
1	17,539		29961979	853167	33,954
2	23,993		45670857	1003299	51,755
3	25,931		12610971	266531	14,291
Total			88243808	2122997	100,000

Crude reaction mixture

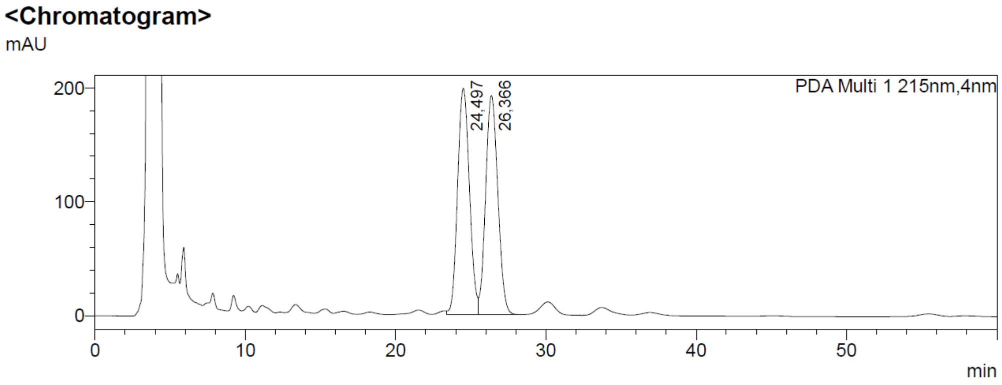

<Peak Table>

PDA Ch1 215nm

Peak#	Ret. Time	Name	Area	Height	Area%
1	24,497		10907975	198611	50,060
2	26,366		10881849	192232	49,940
Total			21789824	390843	100,000

Racemic reference of substrate **SI-1**

Control experiment for enzyme catalysis during the ring closure (see page 12 in this supporting information).

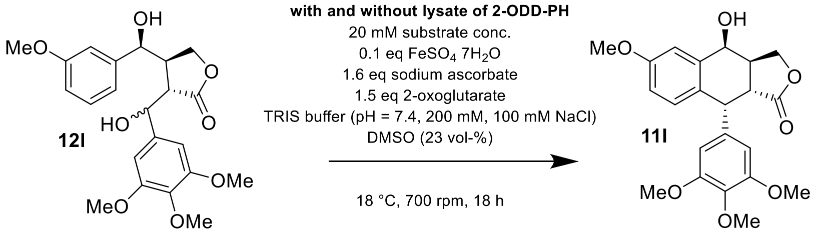

Reaction scheme

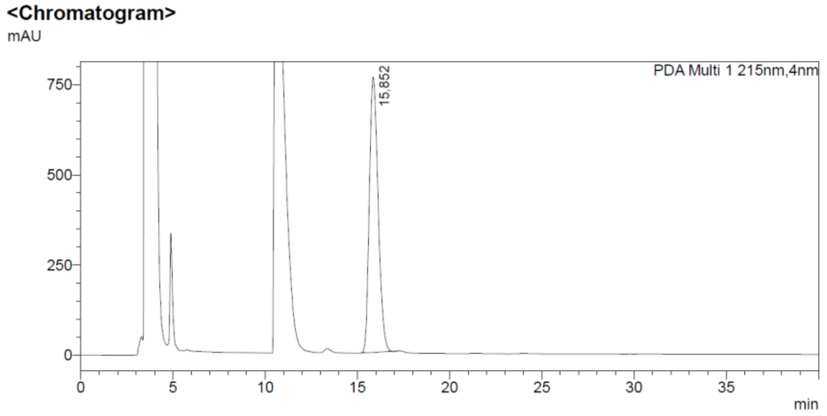

<Peak Table>

PDA Ch1 215nm

Peak#	Ret. Time	Area	Height	Area%
1	15,852	25284433	762640	100,000
Total		25284433	762640	100,000

Incubation of **12l** in the reaction mixture including 2-ODD-PH lysate

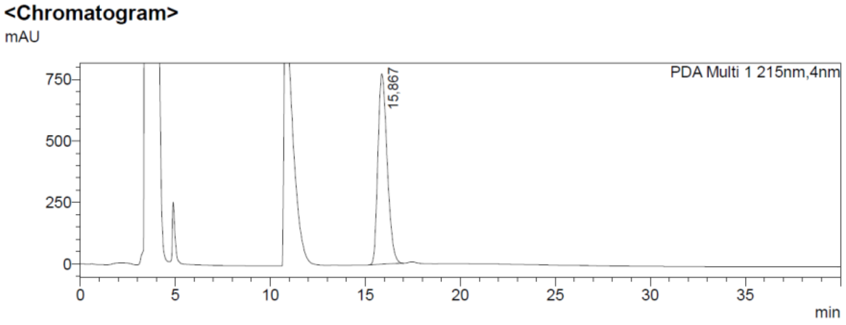

<Peak Table>

PDA Ch1 215nm

Peak#	Ret. Time	Name	Area	Height	Area%
1	15,867		26385660	773775	100,000
Total			26385660	773775	100,000

Incubation of **12l** in the reaction mixture excluding 2-ODD-PH lysate and using buffer instead

<Chromatogram>

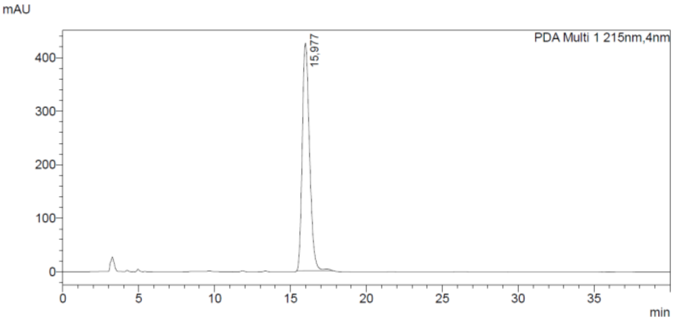

<Peak Table>

PDA Ch1 215nm

Peak#	Ret. Time	Area	Height	Area%
1	15,977	14234600	425716	100,000
Total		14234600	425716	100,000

Chromatogram of compound **12l** for comparison

<Chromatogram>

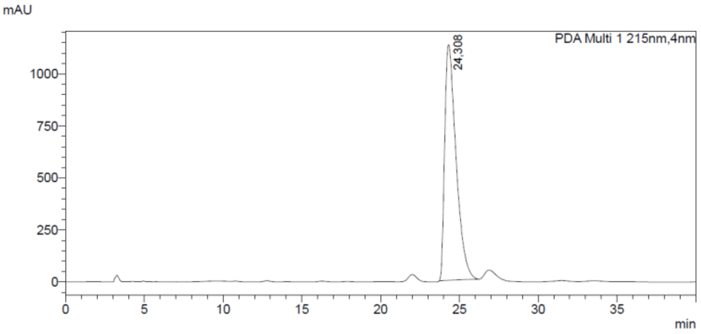

<Peak Table>

PDA Ch1 215nm

Peak#	Ret. Time	Area	Height	Area%
1	24,308	56811999	1132624	100,000
Total		56811999	1132624	100,000

Chromatogram of compound **11l** for comparison

Podophyllotoxin (1).

<Chromatogram>

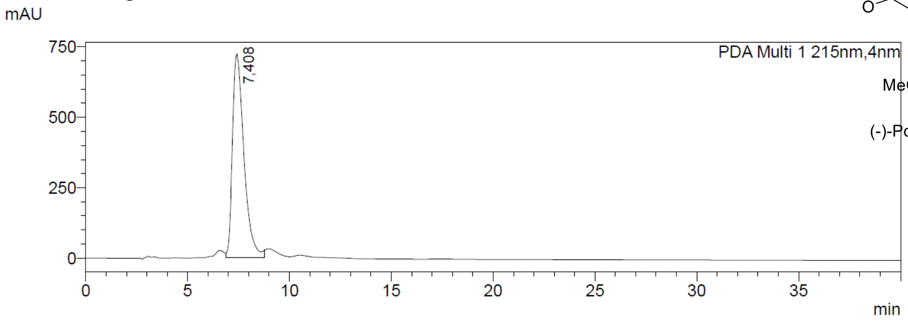

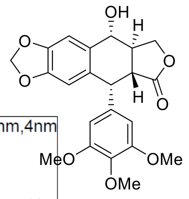

(-)-Podophyllotoxin
(**1**)

<Peak Table>

PDA Ch1 215nm

Peak#	Ret. Time	Name	Area	Height	Area%
1	7,408		28467254	723520	100,000
Total			28467254	723520	100,000

The retention times for the enantiomers are reported to be t_{ret}[(-)-podophyllotoxin] = 8.2 min, t_{ret}[(+)-podophyllotoxin] = 14.0 min.[16]

Calibration Curves for Table SI14b (see p. 18)

Table SI23. Calibration Curves for Table SI14b (see p. 18)

<table>
<tr><th rowspan="2">Substrate</th><th colspan="2">Calibration Curves[a]</th></tr>
<tr><th>Substrate</th><th>Product</th></tr>
<tr><td>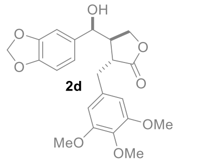</td><td>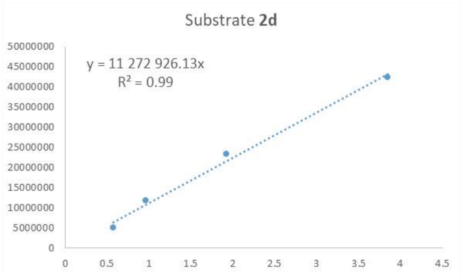</td><td>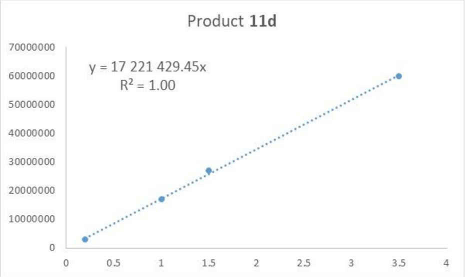</td></tr>
<tr><td>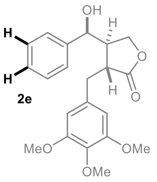</td><td>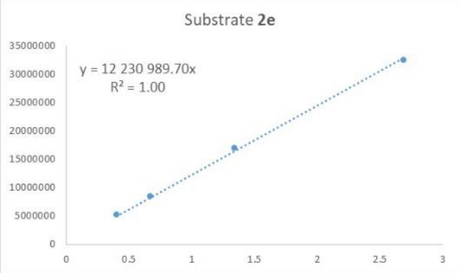</td><td>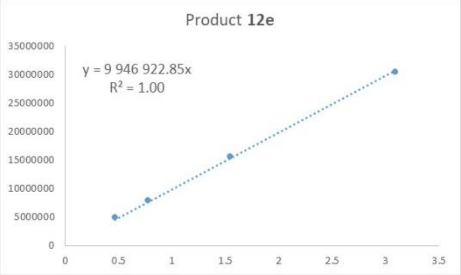</td></tr>
<tr><td>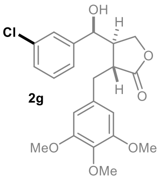</td><td>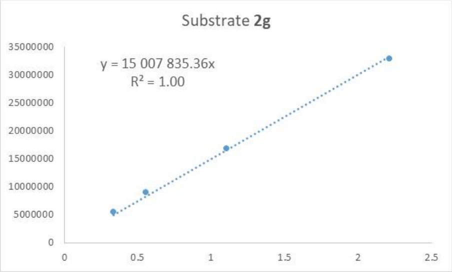</td><td>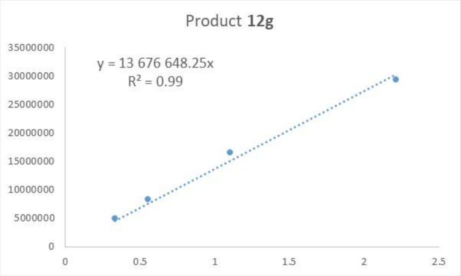</td></tr>
<tr><td>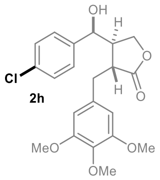</td><td>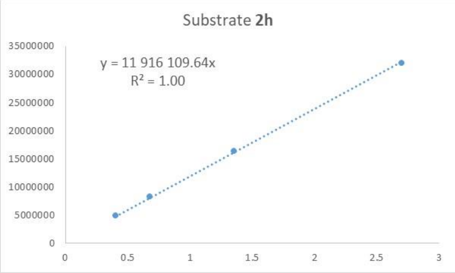</td><td>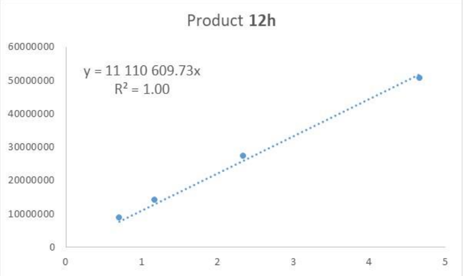</td></tr>
<tr><td>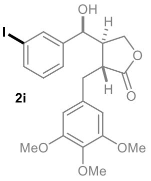</td><td>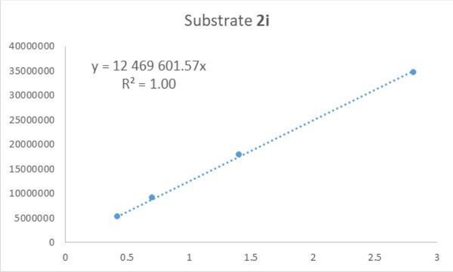</td><td>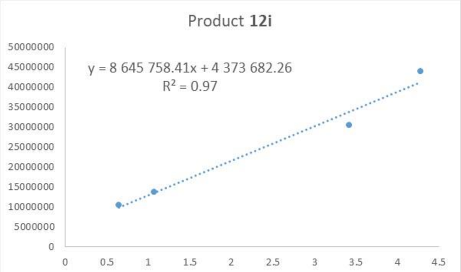</td></tr>
</table>

[a] x-axis: concentration [mmol L^{-1}]; y-axis: HPLC-UV absorption at 215 nm [ ]

Table SI23 – continued.

Substrate	Calibration Curves[a] *Substrate*	*Product*
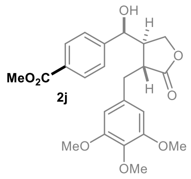 **2j**	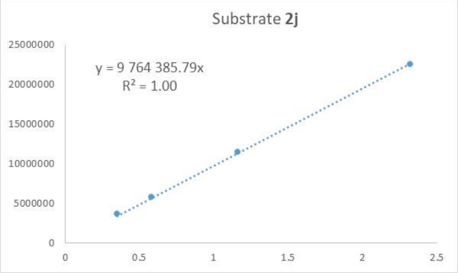	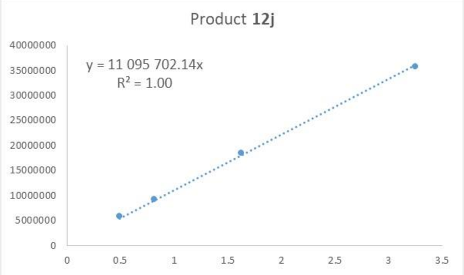
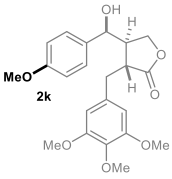 **2k**	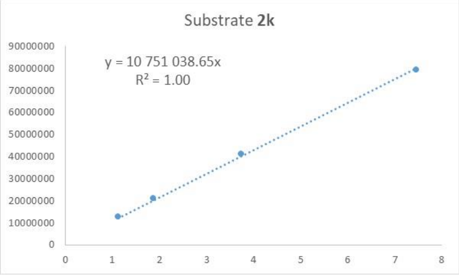	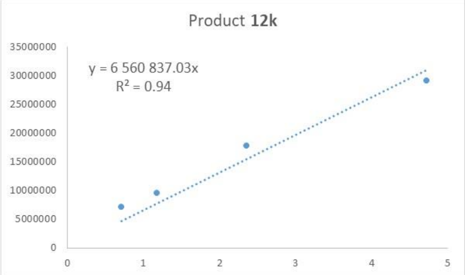
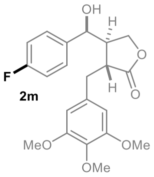 **2m**	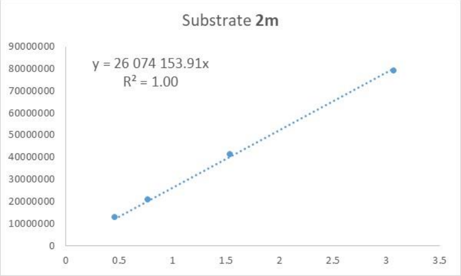	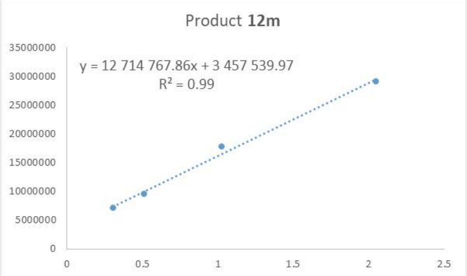

[a] x-axis: concentration [mmol L^{-1}]; y-axis: HPLC-UV absorption at 215 nm [ ]
